# Resistance and robustness of the global coral–symbiont network

**DOI:** 10.1002/ecy.2990

**Published:** 2020-02-14

**Authors:** Sara D. Williams, Mark R. Patterson

**Affiliations:** ^1^ Department of Marine and Environmental Sciences Marine Science Center Northeastern University Nahant Massachusetts 01908 USA; ^2^ Department of Civil and Environmental Engineering Northeastern University Boston Massachusetts 02115 USA

**Keywords:** coral reefs, coral bleaching, ecological network, ecological robustness, symbiosis, resistance, mutualistic network

## Abstract

Increasing ocean temperatures have widespread consequences for coral reefs, one of which is coral bleaching. We analyzed a global network of associations between coral species and Symbiodiniaceae for resistance to temperature stress and robustness to perturbations. Null networks were created by changing either the physiological parameters of the nodes or the structures of the networks. We developed a bleaching model in which each link, association, is given a weight based on temperature thresholds for specific host–symbiont pairs and links are removed as temperature increases. Resistance to temperature stress was determined from the response of the networks to the bleaching model. Ecological robustness, defined by how much perturbation is needed to decrease the number of nodes by 50%, was determined for multiple removal models that considered traits of the hosts, symbionts, and their associations. Network resistance to bleaching and robustness to perturbations differed from the null networks and varied across spatial scales, supporting that thermal tolerances, local association patterns, and environment play an important role in network persistence. Networks were more robust to attacks on associations than to attacks on species. Although the global network was fairly robust to random link removals, when links are removed according to the bleaching model, robustness decreases by about 20%. Specific environmental attacks, in the form of increasing temperatures, destabilize the global network of coral species and Symbiodiniaceae. On a global scale, the network was more robust to removals of links with susceptible Symbiodiniaceae than it was to removals of links with susceptible hosts. Thus, the symbionts convey more stability to the symbiosis than the hosts when the system is under an environmental attack. However, our results also provide evidence that the environment of the networks affects robustness to link perturbations. Our work shows that ecological resistance and robustness can be assessed through network analysis that considers specific biological traits and functional weaknesses. The global network of associations between corals and Symbiodiniaceae and its distribution of thermal tolerances are non‐random, and the evolution of this architecture has led to higher sensitivity to environmental perturbations.

## Introduction

The resistance of coral reefs to changing environmental conditions is a central theme of coral ecology as reefs continue to decline worldwide. Coral bleaching, the breakdown of the association between the coral host and its endosymbiotic algae, is a considerable force behind the deterioration of coral reefs (Hughes et al. 2018). Bleaching responses vary across species, individuals, and stress events (Loya et al. 2001, Baker 2003). Environmental factors drive bleaching patterns on a large scale (Nakamura and Van Woesik 2001), but the variation in bleaching response is attributed to the complex associations among coral hosts and their symbiotic algae, dinoflagellates in the family Symbiodiniaceae (LaJeunesse et al. 2008, 2018). Corals hosting specific symbionts (Rowan 1998, LaJeunesse 2001, Glynn et al. 2001, Toller et al. 2001, van Oppen et al. 2001), multiple symbiont types (Loh et al. 2001, Baker 2003), and diverse background symbiont populations (LaJeunesse 2002, Quigley et al. 2014, Ziegler et al. 2017) may be more resistant to thermal stress. These complex symbiotic associations can be analyzed as a network of coral species interacting with members of the family Symbiodiniaceae.

Network science conceptualizes complex systems as components, represented by nodes, connected to other components, by their interactions. Complex systems from social systems to ecological systems have been found to have heterogeneous or close to scale‐free structure, where the distribution of node connections, the degree distribution, follows a power law in which some nodes have a lot of links (the hubs) and most nodes have only a few links (Holme 2019). The structure and topology of complex systems determines their ability to withstand perturbation (Albert et al. 2000, Cohen et al. 2001, Allesina and Pascual 2007). Unlike random networks, scale‐free and heterogeneous networks are robust to random failures (node removals) and highly susceptible to targeted “attacks” on hubs (Albert et al. 2000). Susceptibility to attacks due to network structure has been found in food webs (Solé and Montoya 2001, Dunne et al. 2002) and mutualistic networks of plants and their pollinators (Bascompte and Jordano 2007). Typically, attacks on ecological networks are modeled as species extinctions, i.e., node removals. While the adaptive rewiring of links has been found to aggravate the effects of species loss (Gilljam et al. 2015), few studies have modeled attacks as interaction extinctions, or link removals (Valiente‐Banuet et al. 2015).

With the advent of ecoinformatics databases (e.g., GeoSymbio; Franklin et al. 2012) and finer resolution sequencing technologies, network analysis has been used to better understand associations among corals and Symbiodiniaceae. Fabina et al. (2012) analyzed a selected, well‐sampled portion of the GeoSymbio database and found the network to be sparse and significantly nested, key attributes thought to support network stability (Saavedra et al. 2011, Rohr et al. 2014). Fabina et al. (2013) modeled species loss effects on the robustness of the coral–symbiont network in Moorea, French Polynesia, and found that when Symbiodiniaceae nodes (designated ITS2 types) were removed based on clade‐level (currently viewed as genus level; LaJeunesse et al. 2018) thermal tolerance, the network was less robust than if Symbiodiniaceae were removed based on nutritional benefit. Network robustness also decreased when generalist (high‐degree nodes) and dominant symbiont types were removed (Fabina et al. 2013). Similarly, Ziegler et al. (2017) found that an abundance of rare background symbionts increased the robustness of a network of coral hosts and symbionts in the Red Sea, Sea of Oman, and the Persian/Arabian Gulf.

These initial analyses provide insight into the coral–symbiont network’s robustness to perturbations. However, two key aspects are yet to be explored. First, there is evidence for within‐genus differences in symbiont thermal tolerance (Ladner et al. 2012, Swain et al. 2017). Second, the removal conditions set by Fabina et al. (2013) ignore the contribution of the host to the coral’s ability to withstand thermal stress, when there is significant evidence supporting a combined host and symbiont, a holobiont, centric view of coral thermal tolerance (cf. Berkelmans and van Oppen 2006, Baird et al. 2008, Wooldridge 2014). Thus, a measure of combined resistance to temperature stress that incorporates more physiological and environmental data is needed for a better understanding of the coral–symbiont network. Although meta‐analyses of organismal physiology are common (Swain et al. 2016, 2017), network analyses of ecological stability have yet to regularly incorporate these data when exploring impacts of climate change on ecosystems. Coral bleaching can be considered as a perturbation on the network, so that measures of network robustness (Dunne et al. 2002, Yang et al. 2017) can be used to predict the association's stability under environmental stress. Our study further distinguishes resistance from robustness as a system‐specific measure. Resistance and robustness metrics are defined below in more detail; qualitatively, resistance measures performance of the network when under an environmental attack, while robustness measures network fragility when nodes or links are removed.

As the likelihood of annual coral bleaching events continues to increase in the coming decades (Hughes et al. 2018), a global approach to understanding the complex network and fragile relationship of coral species and their algal symbionts is needed from the cellular to the ecosystem level (Suggett and Smith 2019). We developed a network model for coral bleaching that uses exposure to elevated sea surface temperatures (NOAA Coral Reef Watch) and assigns thermal tolerances to both hosts (Swain et al. 2016) and symbionts (Swain et al. 2017) to determine the point of ecophysiological breakdown of the symbiosis under temperature stress on a global scale. The network of coral species and their associated Symbiodiniaceae was created using the GeoSymbio database (Franklin et al. 2012). We define the network’s resistance as the rise in temperature (°C) that increases the percentage of hosts bleached from 10% to 90% normalized by the maximum possible temperature excursion. We use the accepted metric of ecological robustness, i.e., the percentage of nodes (or in our case, also links) that must be removed to decrease the nodes remaining to 50% (Dunne et al. 2002) to quantify the effect of various network perturbations. Our model of coral bleaching, metric for network resistance to temperature stress, and ecological robustness metric allow us to answer the following questions: (1) How does network structure and the distribution of thermal tolerances affect resistance to temperature stress? (2) How does spatial scale, and thus local association patterns and environment impact network resistance to temperature stress and robustness to perturbations? (3) Is the network more robust to interaction or species removals? (4) Does environment, hosts, or symbionts convey more robustness to the network? We demonstrate that a network approach allows us to determine patterns of resistance to temperature stress and ecosystem robustness on global, ocean‐basin, and subregional scales for coral species and their symbiotic algae.

## Methods

### Data and network structure

Data from the GeoSymbio database (Franklin et al. 2012) were used to construct the global network of coral species and their associated Symbiodiniaceae (designated ITS2 types). Since the database was published in 2012, there has been much discussion over the usefulness of the ITS2 marker in characterizing Symbiodiniaceae intragenomic and intergenomic diversity (Hume et al. 2019). There is a lot of masked diversity within ITS2 phylotypes (Thornhill et al. 2014). A high level of host specificity and variation in thermal tolerances is unaccounted for when only using ITS2 symbiont designations (Thornhill et al. 2014, Hume et al. 2015, Smith et al. 2017). Theoretically, we can test the effects of greater host specificity on network structure and resistance to temperature stress by creating a network where the symbiont hubs are split into their degree number of nodes to represent many genetic variants (Appendix S1). However, this model does not yet have enough supporting data. Since an equivalent database to GeoSymbio that uses the updated ITS2‐type profiles designation (Hume et al. 2019) does not yet exist, we carried forth our analyses with the GeoSymbio data.

**Figure 1:**
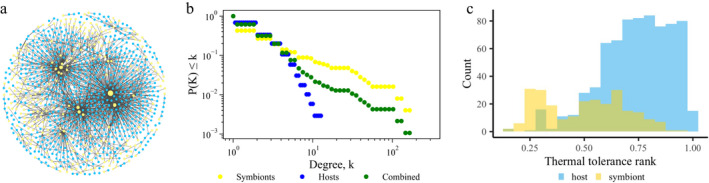
(a) Visualization of the global coral–symbiont network. Host nodes are in blue and symbiont nodes are in yellow. Size corresponds to degree. (b) Degree distribution, cumulative probability of nodes in the network having degree *k*, of the global network considering hosts and symbionts together (combined) and separately. (c) Distribution of thermal tolerances of host and symbiont nodes in the global network.

Data from the GeoSymbio database were filtered to contain only scleractinian corals (3,519 total records), in situ samples, non‐redundant Symbiodiniaceae ITS2 types, and samples with a known state‐region location (reclassified here as subregion). Once duplicate associations were removed, the data set consisted of 1,697 records of geographically unique coral–symbiont associations (Data S1), where each record is represented by a link in the global network (Table 1, Fig. 1a). Links connect a geographically distinct coral host node to its symbiont partner node, resulting in a total node count of 935 for the global network. Therefore, if a coral species was found in multiple subregions, then it is represented in the network by multiple nodes that correspond to each subregion location (Table 1). Including multiple nodes for host species based on geographic differences does not markedly change the structure of the network, and thus this network configuration was chosen to include the most environmental information (Appendix S1). In total, there are 685 host nodes, but only 362 coral species are represented in the network. In as much as the location datum is attached to the coral host node, designated Symbiodiniaceae ITS2 types are only represented by one node each in the network, regardless of whether or not they are found in multiple locations. There are 250 symbiont nodes representing Symbiodiniaceae from the genera *Symbiodinium* (formerly clade A), *Breviolum* (formerly clade B), *Cladocopium* (formerly clade C), and *Durusdinium* (formerly clade D). The global network has a truncated power law degree distribution (fit using the powerlaw package in Python 2.7; Appendix S1); thus, the average degree (〈*k*〉 = 3.63) is not typical of most nodes, and there are many specialists and few generalists (Fig. 1b). The three major network hubs are symbionts in *Cladocopium* that interact with more than 140 host nodes (Appendix S1: Table S2). Most hosts associate with fewer than 20 species of Symbiodiniaceae. The global network was divided into subsets by location to create 13 networks based on spatial scale (Table 1; Appendix S1: Table S1). A global network of coral–Symbiodiniaceae associations is necessary for studying the persistence of coral–symbiont associations on a global scale and provides a larger system to explore network analysis of this important symbiosis.

**Table 1:** Spatial division of natural networks nested within the global network.

Region and subregion	*T*_MMM (2005)_ (°C)	Hosts	Links
Pacific Ocean			
Great Barrier Reef	28.68	157	315
Central Pacific	27.60	30	147
Japan	28.54	58	84
Eastern Pacific	27.63	14	43
Western Pacific†	29.56	16	24
American Samoa†	29.87	2	11
Indian Ocean			
Phuket	30.38	140	404
Western Indian	28.97	109	272
Western Australia	28.78	20	55
Caribbean Sea			
Western Caribbean	29.72	52	122
Eastern Caribbean	29.63	40	106
Central Caribbean	29.94	31	75
Florida†	30.25	15	38
Gulf of Mexico†	30.54	1	1

Notes

There are three spatial scales: global, region (ocean‐basin), and subregion. Environmental data, mean monthly maximum sea surface temperatures from 2005 (*T*_MMM (2005)_,°C), are assigned to host nodes based on subregional location data. Every host node is a coral species found in a specific subregion, so that coral species found in multiple subregions are represented in the network as multiple nodes. Region‐scale natural networks (Pacific Ocean, Indian Ocean, and Caribbean Sea) contain all hosts, symbionts, and links from their respective subregions. Total number of nodes (*N*) and links (*L*) for the global and ocean‐basin networks, as well as a measure of their realized number of links, connectance (*C*, no. links/*Hosts* × *Symbionts*), are as follows: global (*N* = 935, *L* = 1697, *C* = 0.002); Pacific Ocean (*N* = 403, *L* = 624, *C* = 0.004); Indian Ocean (*N* = 343, *L* = 731, *C* = 0.006); Caribbean Sea (*N* = 220, *L* = 342, *C* = 0.007). Symbiodiniaceae ITS2 types are represented by 250 symbiont nodes in the global network. †Locations that had <40 links and were not used in calculations of resistance and robustness.

Nodes were assigned tolerance values ranging from 0 to 1 that represent how well a node can cope with temperature stress. These values were adapted from two meta‐analyses of coral and Symbiodiniaceae thermal tolerances (Swain et al. 2016, 2017). The taxon‐level Bleaching Resistance Index (BRI) metric developed by Swain et al. (2016) represents the fraction of bleached tissue that would be observed on a specific coral species during a heat stress event. All taxa found in Swain et al. (2016) were included in our analysis; however, our network includes more coral species than were examined by these authors. If a species was not listed in Swain et al. (2016), it was given a BRI value of a random number generated from the mean and standard deviation of its closest relative. Host node tolerances were calculated by dividing a species BRI by 100 to get a value within the range of 0–1 and then subtracting that value from 1, since the tolerance scheme used here is the opposite of Swain et al. (2016)’s BRI index (Fig. 1c). A lower value of our index indicates a coral is more susceptible to bleaching.

Swain et al. (2017) provides a framework for a consensus of Symbiodiniaceae thermal tolerance scores developed from rank‐aggregation methods. Their ranking scheme orders Symbiodiniaceae ITS2 phylotypes from 0 to 100, but the values are not indicative of total magnitude differences in thermal tolerance (Swain et al. 2017). Thus, we used a square‐root transformation of the rank scores to decrease the total difference in magnitude among the ranks. The square‐root transformation effectively narrows the scale of the ranks, although it may lead to an underestimate of the more tolerant symbionts. The square root of the rank was then divided by 10 to transform the values onto a 0–1 scale. To determine tolerances of the unlisted symbiont types, rank values were randomly drawn from the high, medium, and low thermal tolerance frequency distributions determined by Swain et al. (2017) in the relative proportions of the clades represented in those distributions (Appendix S1: Fig. S1). Thus, for each simulation of either the bleaching or different removal models described below, the symbiont tolerances varied within a set distribution (Fig. 1c). The node tolerances (dimensionless) are used as physiological parameters in the bleaching and removal models.

### Bleaching model

A weighted link‐removal model was developed to simulate coral bleaching on the network. Each link is given a weight based on how much temperature stress a certain coral–symbiont association can tolerate before the symbiotic interaction is broken. The weight serves as an association‐specific temperature threshold. The bleaching model ramps up environmental temperature and links are removed when their weight is exceeded. A coral species is considered bleached once it is isolated, i.e., it has no more links to alternative symbionts.

The weighting function is based on two important aspects of coral biology: (1) corals are predicted to start bleaching once temperatures rise over the mean monthly maximum sea surface temperature (Donner et al. 2005), and (2) there is an upper thermal limit for the coral–Symbiodiniaceae association (Jokiel and Coles 1990, Fitt et al. 2001)

(1) $$W_{h,s}=T_{\text{MMM}(2005)}+T_{\Delta }\left(\tau_{\text{symbiont}}+\tau_{\text{host}}\right)/2.$$

Our weighting function (*W*_h,s_) uses the average 2005 mean monthly maximum sea surface temperature (*T*_MMM(2005)_) of the subregional location of the host node (Table 1). Sea surface temperature has been shown to be a reliable predictor of bleaching (Heron et al. 2016) and is freely available from NOAA Coral Reef Watch. The last major global bleaching event within the time period of the data represented in GeoSymbio was in 2005 (Eakin et al. 2010), and thus is an appropriate time point for our model. The host (τ_host_) and symbiont (τ_symbiont_) tolerances are averaged to account for uncertainty of which partner is responsible for the breakdown of the symbiosis (Wooldridge 2014). *T*_Δ_ is the upper thermal limit for the mutualism interaction and was chosen to be 3.0°C (Jokiel and Coles 1990, Fitt et al. 2001). The averaged tolerance is multiplied by the upper thermal limit to give a measure of how much of the thermal limit a pair can tolerate.

Model simulations of the above bleaching model were coded as a function in Python (v2.7, Data S2). The bleaching model was applied to the natural networks (global, ocean basins, and subregions) and compared to four null networks created for each. These null networks are listed below and change either the thermal tolerance distribution or association structure of the networks to address the first objective of this research.

#### Shuffled tolerance

Tolerances of both node types were replaced with new values randomly drawn from within the original thermal tolerance distributions. Do specific thermal tolerances affect resistance?

#### Random tolerance

Tolerances of both node types were replaced with values randomly chosen from a uniform distribution. Does the thermal tolerance distribution found in nature affect resistance?

#### Random bipartite network degree conserved (RBDC)

The links of the network were randomized, but the degree distribution was held constant (NetworkX; Hagberg et al. 2008). The shuffled tolerances were used for this null network as well. Do specific interaction patterns affect resistance?

#### Random bipartite network not degree conserved (RBNDC)

The structure of the network was changed to that of a random bipartite network using a random graph generator (NetworkX; Hagberg et al. 2008) and the tolerances used were those of the shuffled tolerance null network. Does the interaction (network) structure affect resistance?

### Bleaching resistance metric

One hundred simulations of the bleaching model were run for each network: natural networks and the four null networks for each spatial scale. The bleaching model returns the percent of corals bleached as a function of temperature increase in degrees Celsius. The response curves are logistic or bi‐logistic functions. Logistic functions explain a range of biological and socio‐technical systems, and are often described by their characteristic time, the length of time required for the growth process to grow from 10% to 90% of the saturation level (Meyer 1994). This characteristic time can be used to describe the resistance of a system if we consider time to be any perturbation, temperature, in our case. Thus, we define resistance as the amount of temperature required for the network to go from 10% to 90% of hosts bleached normalized by the maximum temperature change for this range (3°C, *T*_Δ_ from model parameters). This definition of resistance is dimensionless, can be adapted for different stressors and complex systems, and closely aligns with the accepted concept of resistance as the ability of a system to withstand or resist a disturbance. The change in temperature between the 10% and 90% bleached values was calculated for each simulation. Randomization tests (described in detail in Appendix S2) were performed to determine statistical significance in differences among the resistance values of the natural and null networks and resistance values across spatial scales.

### Ecological robustness metric

Robustness of food webs to species loss has been quantified as the proportion of species removed that resulted in a total loss of some specified proportion of the species in the network (Dunne et al. 2002, Dunne and Williams 2009). Fabina et al. (2013) applied this metric to the bipartite network of coral species and their symbionts in Moorea, French Polynesia, by simulating local extinctions, but this analysis has not been applied on a global scale for a coral–symbiont network. Node removals from ecological networks represent a species removal, i.e., an extinction and previous robustness models have focused on removing specialist (low degree) or generalist (high degree) species (see Appendix S2 for these removal models on our networks). However, associations are likely to change or disappear on more ecologically relevant timescales. This is the case for corals, where the association between a coral host and its symbionts can disappear (i.e., coral bleaching) before either goes extinct. Link removals were considered here to remedy this neglected perturbation type. The following five removal models were simulated on the natural networks.

#### Random links

Links were removed from the network in a random order. This removal model serves as a baseline comparison for the other link removal models.

#### Bleaching

Links were removed according to the bleaching model (in order of low to high *W*_h,s_) described above.

#### Susceptible links

Links were removed from the network based on the tolerances of the connected nodes in order of low (susceptible) to high tolerances. Link tolerances were set to be the averaged tolerances of the nodes, which considers the combined tolerance of the holobiont or just the tolerance of the host or the symbiont node in order to explore which partner imparts more resistance to the symbiosis.

#### Random nodes

Nodes were removed randomly from the network. This node removal model serves as a baseline comparison for the other node removal models.

#### Susceptible nodes

Nodes were removed from the network in order of low to high tolerance.

Each removal model was coded in Python (v2.7, Data S2) and simulated on the networks 100 times. Although resistance directly simulates bleaching on the networks, robustness treats bleaching and the other removal models as a stepped perturbation and tracks the number of removals, as well as the remaining nodes. Robustness of the networks to the removals was determined by finding the *R*50 value, the number of nodes or links needed to be removed to decrease the number of nodes remaining to 50%, a common threshold value used to analyze the robustness of ecological networks (Dunne et al. 2002, Dunne and Williams 2009). *R*50 values were determined for the total network (host and symbiont nodes grouped; see Appendix S2 for additional host and symbiont robustness scenarios). Randomization tests (described in detail in Appendix S2) were performed to determine statistical significance in robustness values of the different removal models for each spatial scale.

## Results

### Bleaching model simulations and bleaching resistance

**Figure 2:**
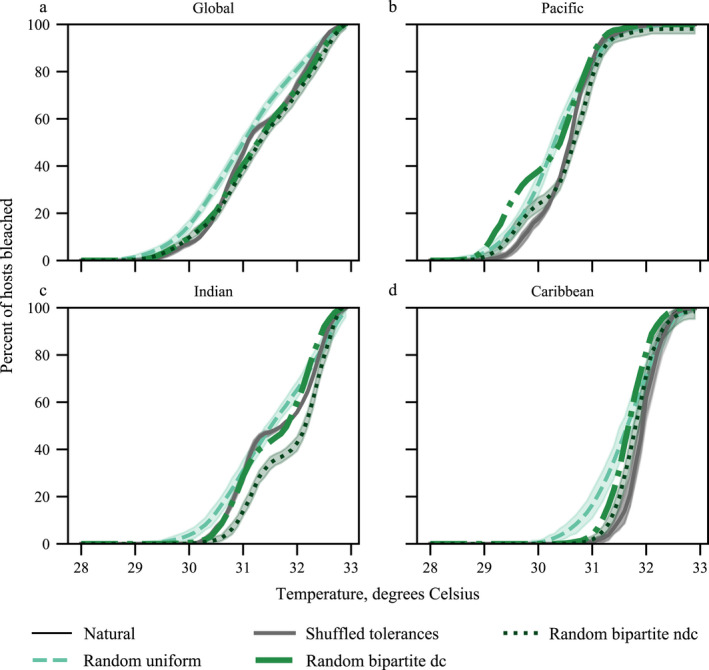
Percentage of hosts bleached as a function of each degree Celsius of temperature increase of the 100 simulations of the bleaching model (Eq. 1) on the (a) global, (b) Pacific Ocean, (c) Indian Ocean, and (d) Caribbean Sea networks and each of their associated null networks (dc, degree conserved; ndc, non‐degree conserved). Shaded regions are the 97% confidence intervals.

Simulations of the bleaching model result in response curves of the percentage of corals bleached as a function of temperature increase in degrees Celsius (Fig. 2). The weighting function explains the temperature separation of the different spatial networks, because each network has a bleaching temperature range based on its respective *T*_MMM(2005)_ values. The null networks all start bleaching at lower temperatures than their natural counterparts. The double logistic functions of the global, Pacific Ocean, and Indian Ocean networks’ response curves occur because of differences among *T*_MMM(2005)_ values of their subregions that result in intermediate saturation points.

**Figure 3:**
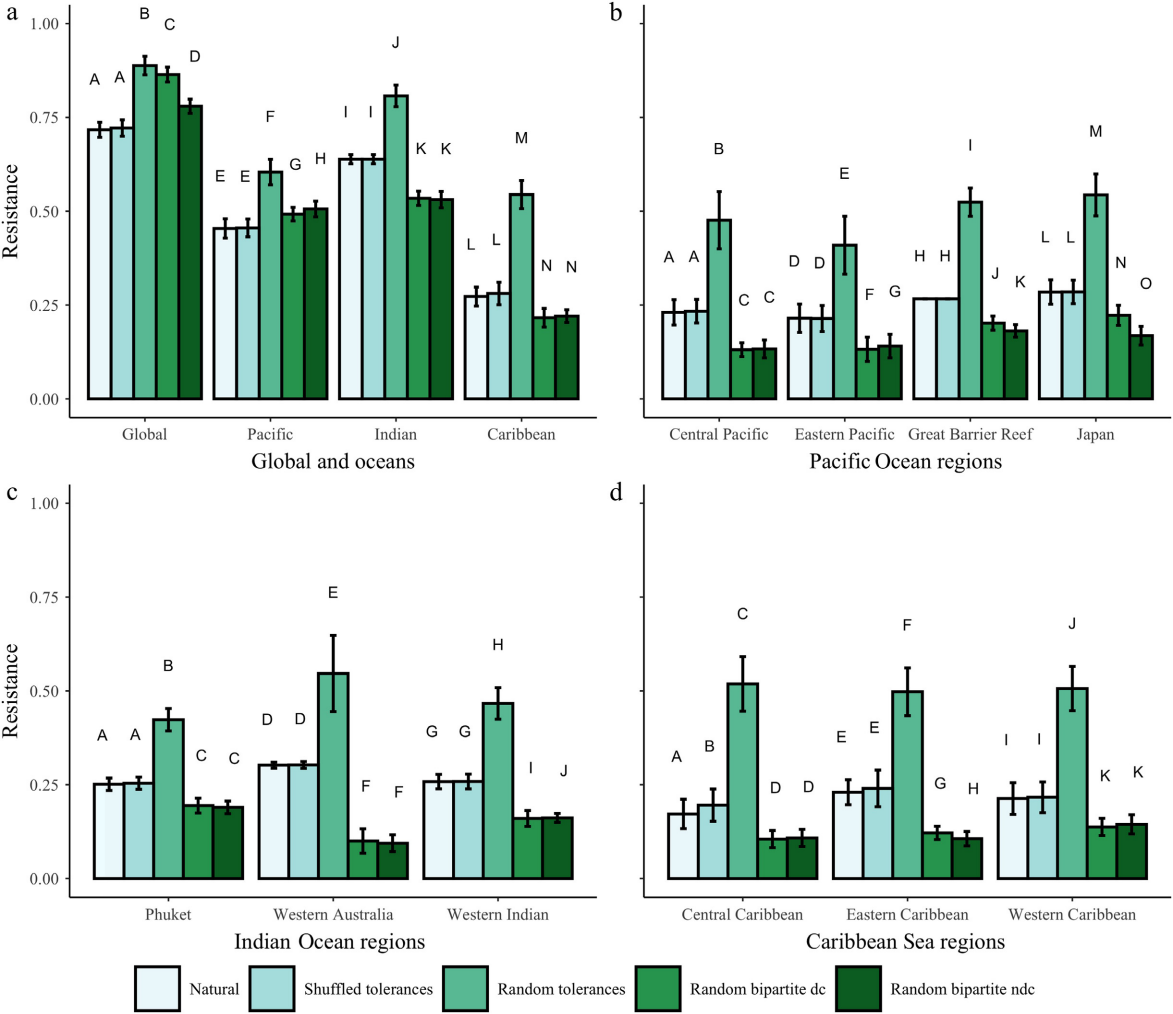
Resistance of the networks (all spatial scales and their associated nulls) to thermal stress calculated as the increase in temperature from 10% of hosts bleached to 90% of hosts bleached and then normalized by the maximum possible temperature change in this range (3°C, *T*_Δ_). Error bars represent the standard deviation of the mean resistance determined from the 100 simulations. Different letters above bars signify significant differences (*P* ≤ 0.05) determined by the randomization tests described in detail in Appendix S2.

Differences in resistance values for the natural networks and their nulls (Fig. 3) provide answers for how network structure and the distribution of thermal tolerances, as well as spatial scale, affect a coral–symbiont network’s resistance to temperature stress. Shuffling a network’s tolerances does not significantly affect its resistance to temperature stress regardless of spatial scale, except for the Central Caribbean network (*P* < 0.01, Fig. 3d). However, by changing the distribution of a network’s tolerances to that of a random uniform distribution, the network becomes more resistant to temperature stress. At all spatial scales, the random tolerance null network is the most resistant network. The natural distribution of thermal tolerances found on coral reefs makes them susceptible to temperature stress. Changing a network’s structure either by completely changing the degree distribution or just by rearranging links does increase a network’s resistance on larger spatial scales (global and Pacific, Fig. 3a), but this affect disappears at smaller spatial scales (Caribbean, Indian, and subregions, Fig. 3).

All natural networks have significantly different values of resistance (Fig. 3, Data S2), indicating that changes in network structure across scales and local environments affect the resistance of coral–symbiont associations. The global network has the highest resistance (*R* = 0.717 ± 0.020) of all the natural networks. At the ocean‐basin scale, the Indian Ocean is the most resistant network (*R* = 0.639 ± 0.012), followed by the Pacific Ocean (*R* = 0.454 ± 0.026) and the Caribbean Sea (*R* = 0.273 ± 0.025). Within the Pacific subregion networks, the Great Barrier Reef (*R* = 0.267 ± 0.000) and Japan (*R* = 0.285 ± 0.033) networks are more resistant than the Central (*R* = 0.231 ± 0.034) and Eastern Pacific (*R* = 0.215 ± 0.038) networks. The Western Australia (*R* = 0.302 ± 0.008) subregion is the most resistant network of the Indian Ocean subregions (Western Indian, *R* = 0.258 ± 0.019; Phuket, *R* = 0.251 ± 0.017). The Eastern (*R* = 0.233 ± 0.033) and Western (*R* = 0.213 ± 0.042) Caribbean networks are more resistant than the Central Caribbean network. (*R* = 0.172 ± 0.039).

### Ecological robustness

**Figure 4:**
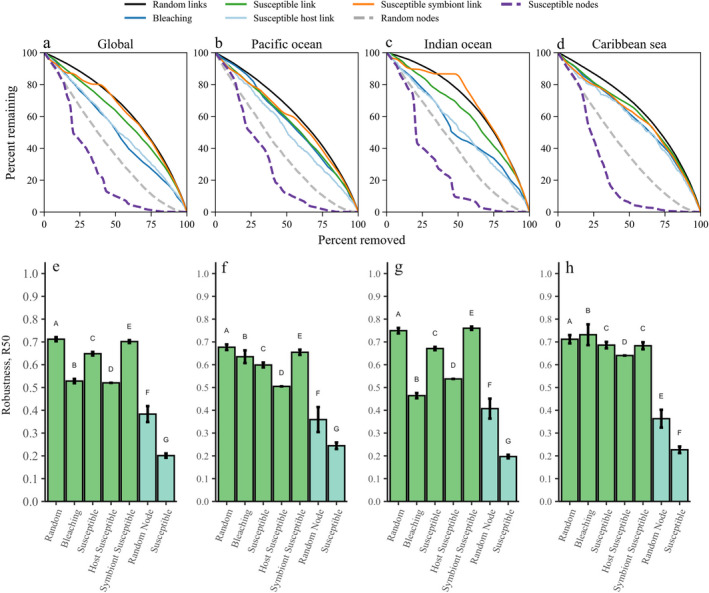
Results, percentage of nodes remaining as a function of percentage of nodes or links removed, of the removal models (mean curve of all 100 simulations) for the (a) global, (b) Pacific Ocean, (c) Indian Ocean, and (d) Caribbean Sea networks. Robustness (*R*50) is measured as the fraction of nodes or links removed needed to decrease the number of nodes in the network by one‐half, with error bars representing the standard deviation, for the (e) global, (f) Pacific Ocean, (g) Indian Ocean, and (h) Caribbean Sea networks. Different letters above bars signify significant differences (*P* ≤ 0.05) determined by the randomization tests described in detail in Appendix S2. Link‐removal models are colored green and node‐removal models are colored blue‐green.

The mean response curves of the 100 simulations of each removal type on the networks provide a visual understanding of robustness (Fig. 4a–d), and the quantitative metric, the *R*50, provides a direct comparative measure of ecological robustness to perturbations (Fig. 4e–h). *R*50 values for each removal type were determined for every spatial scale (Data S2); however, here we only present the robustness results of the global and ocean‐basin networks. Robustness results provide insight into how the networks respond to interaction (link) or species (node) removals and if the hosts or the symbionts convey more stability to the networks. A distinct separation between the networks’ responses to link and node removals are seen when robustness of all nodes remaining is determined. Across spatial scales, networks are significantly more robust to link removals than node removals (Data S2). For 50% of the global network to become isolated, on average 24.3% more links than nodes have to be removed.

The global network is significantly less (*P* < 0.01) robust to removals according to the bleaching model (*R*50 = 0.528 ± 0.009) than to links removed randomly (*R*50 = 0.710 ± 0.008). Thus, a system‐specific attack on the associations decreases robustness. When links are removed in order of averaged susceptible tolerances (which is essentially the bleaching model but without the influence of environmental temperature and a set upper limit), the robustness increases (R50 = 0.658 ± 0.008), so environmental factors decrease robustness on a global scale. The global network is significantly (*P* < 0.01) more robust to links removed in order of susceptible symbionts (*R*50 = 0.702 ± 0.006) than to links removed in order of host susceptibility (*R*50 = 0.520 ± 0.000). Thus, hosts are the weaker partner in the symbiosis on a global scale when considering their role in network robustness. The global network is significantly (*P* < 0.01) more robust to removing nodes randomly (*R*50 = 0.383 ± 0.035) than to removing nodes in order of susceptibility (*R*50 = 0.201 ± 0.010), again supporting the importance of incorporating ecophysiological data in studies of network stability.

Robustness to the different removal models varies across spatial scale, supporting a role for network structure in robustness to perturbations. Robustness tends to increase with increasing connectance (measure of number of realized connections, Table 1; Appendix S2, Data S2) when links are removed according to the bleaching model, though not significantly (Fig. 4e–h; Appendix S2). Of the global and ocean‐basin networks, the Caribbean network was most robust to removals by the bleaching model (*R*50 = 0.731 ± 0.046), followed by the Pacific Ocean network (*R*50 = 0.635 ± 0.028). Of this group, the Indian ocean network was the least robust to removals by the bleaching model (*R*50 = 0.464 ± 0.047). Although the robustness results of the Indian ocean network exactly align with those of the global network, the Pacific and Caribbean networks differ slightly. Both the Pacific and Caribbean networks are significantly more robust to removals by the bleaching model than to removing susceptible links. In the Pacific and Caribbean oceans, environmental factors may help stabilize the network to perturbations.

## Discussion

We developed a novel model to simulate bleaching on the global network of coral and Symbiodiniaceae associations using specific ecophysiological attributes. Our results indicate that the global network of coral species and Symbiodiniaceae associations is susceptible to perturbations that specifically take into account physiological and environmental data and that this susceptibility is, in part, due to the structure of the associations. As ecosystems continue to be threatened by climate change, modeling environmental stress on ecological networks that incorporate ecophysiological data will prove to be a powerful tool for understanding their stability. The networks studied here are of course limited by the data used to create them and do not account for possible temporal shifts in associations (Glynn et al. 2001, Jones et al. 2008, Sampayo et al. 2016) or greater symbiont diversity and host‐specificity that is masked by the use of ITS2 phylotypes (Thornhill et al. 2014, Hume et al. 2019). In our bleaching model, temperature acts as a press perturbation that puts stress on the system’s associations until they break. Our “stress‐test” approach is an effective first pass at modeling coral bleaching to understand resistance and robustness as what happens leading up to the collapse of the coral–symbiont network.

### Heterogeneous structure of the global network decreases its resistance to temperature stress

Our results show that when the connections of the natural global network are randomized, and thus no longer follow the truncated power‐law distribution of having a few hubs and many lowly connected nodes, these homogenous networks become more resistant to temperature stress under the bleaching model (Fig. 3a, RBNDC simulation). Even when the associations are just shuffled with the natural degree distribution conserved, the network’s resistance to temperature stress increases (Fig. 3a, RBDC simulation). This suggests that specific associations, not just the overall structure of the network increase susceptibility to link removals. Although these results are not seen for every location, the result that resistance varies across locations and spatial scales suggests that structure affects resistance. If we consider the bleaching model to be a targeted attack on this symbiosis network based on its ecophysiological properties, these results complement current theory of the resistance of heterogeneous networks to attacks (Albert et al. 2000, Solé and Montoya 2001). However, our bleaching model is a novel, targeted attack type. Previously, attacks have mostly been modeled as species extinctions. The bleaching model attacks the links of the network, the associations of the ecosystem, on an environmental front. When the network’s links are randomized, thus shifting the overall structure to become homogeneous (the RBNDC null network), the stress is more evenly distributed across the network resulting in a more resistant system. The natural networks are a mixed landscape of strong and weak contributors that are connected in such a way that makes them susceptible to temperature stress.

### The natural distribution of thermal tolerances makes the system less resistant

When the distribution of a coral–symbiont network’s tolerances is changed to that of a random uniform distribution, the network becomes more resistant to temperature stress. This is seen at all spatial scales (Fig. 3). We adapted thermal tolerances from two recent meta‐analyses (Swain et al. 2016, 2017) that are to date the most comprehensive ranking of thermal tolerances for coral species and their symbiotic algae. Large comparative experiments and theoretical work like Swain et al. (2016, 2017) are needed to drive the predictive power of network analyses that incorporate ecophysiological data. Network analyses and modeling provide the analytical toolbox for investigating large data sets like GeoSymbio, but as our results have shown, the ecophysiological data are an important part of the networks’ dynamics under stress.

Associations among coral species and their algal symbionts create non‐uniform patterns of thermal tolerance that make the complex system more sensitive to environmental perturbations. Corals have been shown to acclimate to increasing temperatures (Marshall and Baird 2000, Maynard et al. 2008). However, our results show that the overall distribution of thermal tolerances will have to shift to increase future bleaching resistance.

### Spatial scale and local environment affect network resistance and robustness

Only the global and Pacific Ocean networks are less resistant than the null networks that changed the structure of associations (RBDC and RBNDC). However, resistance and robustness of the natural networks vary with location and scale supporting the notion that resistance is a function of network structure since the number of nodes and links, as well as the connectance, of the networks varies across scales (Table 1; Appendix S1 and S2). Hughes et al. (2018) found that the western Atlantic had two to three times more bleaching events from 1980 to 2015 than the Pacific, Indian, or Australasia regions. The Western Atlantic also experienced regular bleaching sooner than the other regions (Hughes et al. 2018). Their findings corroborate our results showing that the Caribbean Sea was the least resistant network of the ocean‐basin networks (Fig. 3). Thus, network analysis and our bleaching model can serve as a predictor of the bleaching resistance of coral reefs on a global scale.

Elevated ocean temperature is the primary cause of mass bleaching and coral die‐offs (Jokiel and Coles 1990, Fitt et al. 2001) and sea surface temperature is a reliable predictor of coral bleaching (Heron et al. 2016). The difference in resistance across scale may also be a function of the *T*_MMM(2005)_ values attached to each host based on the subregion scale of its sampling location providing support for the influence of environment on coral bleaching. Additionally, the global network is more robust to removing links by susceptibility than to removing links by the bleaching model that incorporates temperature. Therefore, including environmental experience in the removal model decreases the system’s robustness to an environmental perturbation. However, other environmental factors like irradiance levels (Lesser et al. 1990) and water flow (Carpenter and Patterson 2007, Carpenter et al. 2010) affect the occurrence and severity of coral bleaching. Our bleaching model is the first of its kind to model an environmental perturbation as a breakdown of an interaction on a physiological level on a network. However, it is only a first pass at modeling coral bleaching on a network, as the use of more and finer resolution environmental parameters may add more predictive power.

### The network is more robust to link removals, unless they are a targeted environmental stressor

Link removals have mostly been ignored in studies of ecological robustness. In the case of coral bleaching, link removal is the most appropriate perturbation type for understanding the complex system under temperature stress. Across scales, networks of corals and their symbionts are more robust to link removals than to node removals. The links of the coral–symbiont network convey more stability to the network than individual nodes. However, the robustness of the global, Pacific Ocean, and Indian Ocean networks to link removals according to the bleaching model is less than that of the networks’ robustness to random removals (Fig. 4). These networks are vulnerable to a targeted environmental attack: bleaching. On ecological timescales, associations and interactions may play a more important role in ecosystem stability, as more often than not, environmental stressors will affect interaction patterns before eradicating entire species**.** Climate change is known to affect species associations and interactions by causing range shifts, behavioral changes, and impacting physiological performance (Doney et al. 2012). All of these happen on much shorter time scales than extinction. As organisms respond to climate change, the structure of ecological networks will first change by losing or shifting links, not by first losing nodes. Initial studies of ecological robustness may have underestimated system stability by only modeling node removals. The associations of the global coral–symbiont network lend the system stability until the links are under attack by a specific environmental stressor.

### Symbiodiniaceae increase the robustness of the coral–symbiont network

Although the main bleaching model averaged the influence of host and symbiont on the coral holobiont’s thermal tolerance, the results of the different removal models allow us to determine which partner conveys more stability to the network. The global and ocean‐basin networks are more robust to the removal of links with susceptible symbionts than to removals according to the bleaching model, susceptible averaged tolerances, and susceptible hosts (Fig. 4). These results would suggest that Symbiodiniaceae convey higher levels of stability to the network in areas where it is needed, i.e., the more highly connected, generalist symbionts have higher thermal tolerances. However, the highly generalist Symbiodiniaceae only have average thermal tolerances (Appendix S1: Table S2). Given the distribution of thermal tolerances and the degree distribution (Fig. 1), it is more likely that enough specialist symbionts have lower thermal tolerances than the key generalist symbionts. From a network science perspective, the coral hosts are the weak partner in this symbiosis when the coral holobiont is under attack by an environmental stressor.

### The possibility and limitations of applying “network triage” on coral reefs

Our results indicate that the associations of a complex ecological system convey more stability to the system than the individual species. This is an important finding when considering how to model the stability of ecosystems under global climate change. Even more so, it is an important finding for understanding how to manage and conserve threatened ecosystems, like coral reefs (National Academies of Sciences, Engineering, and Medicine 2019). Protecting key associations may be more important than protecting individual species. In the context of the global coral reef ecosystem, preserving key associations for network stability starts with a better understanding of coral–symbiont interactions on an ecophysiological level. Future network studies should narrow in on region‐scale collections that sample multiple individuals within a given taxon in a wide range of environmental conditions to allow mapping of the network on a finer scale with more node‐specific information. Within species and individuals, symbiont community composition follows gradients of environmental irradiance (Rowan and Knowlton 1995, Rowan et al. 1997) and temperature (Oliver and Palumbi 2009, Baumann et al. 2018). The GeoSymbio database used to map the networks for this study may not capture local variation of coral–symbiont associations well, and thus local network collections would be useful to coral reef managers in determining reef resistance. Local variability could be mapped through targeted collections to determine where the network of coral–symbiont associations is strongest and where more conservation efforts should be focused. Once the use of ITS2 type profiles is more widely adopted (c.f. Hume et al. 2019), additional network analyses including the higher levels of host specificity and genetic variation should be explored.

**Figure 5:**
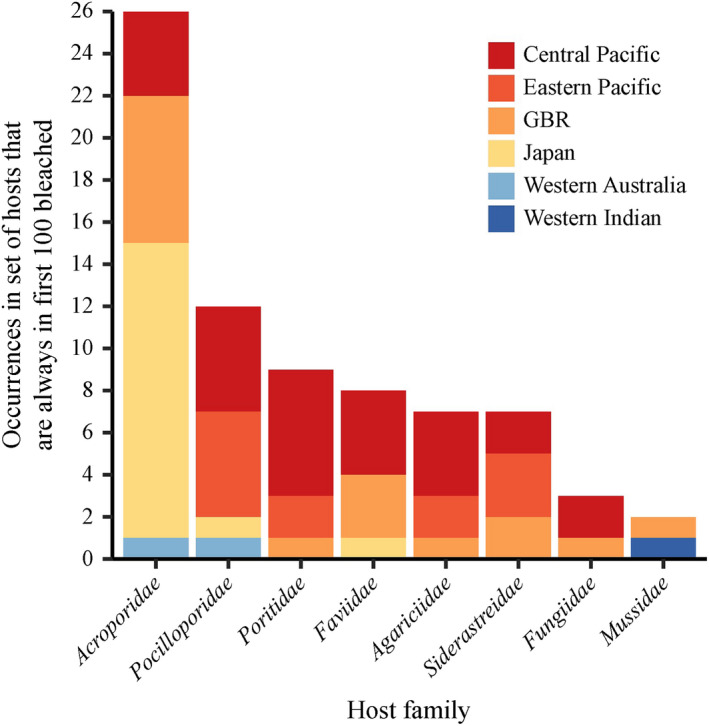
Occurrences of coral host family in the set of host nodes that are always in the first 100 bleached host nodes when the bleaching model is run on the global network of coral–symbiont associations. GBR, Great Barrier Reef.

Network analysis may be useful to managers of coral reef ecosystems in planning restoration efforts and managing other stressors that impact reefs. It can provide insight into what species are most at risk. We can consider a coral species to be very susceptible to bleaching if it appears within the first 100 bleached nodes when the bleaching model is run in multiple simulations. Fig. 5 shows the number of host node occurrences in the first 100 bleached host nodes of 13 coral families and where those host nodes were sampled. According to our bleaching model, the most susceptible coral families include Acroporidae, Pocilloporidae, Poritidae, Favidae, Agaricidae, and Siderastreidae (Fig. 5). Species from these families should be targeted for future studies of network stability. Our resistance model could help decide whether reintroducing or engineering certain coral‐algal network associations (van Oppen et al. 2015, National Academies of Sciences, Engineering, and Medicine 2019) would restabilize a collapsing network. Or it could also elucidate areas where management has neglected key associations that should be conserved. In theory, the distribution of thermal tolerances on a reef or the structure of coral–symbiont interaction patterns could be reconstructed to stabilize the network: a “network triage” approach to coral reef conservation. However, since our results show that environmental experience plays a large role in network robustness, the best conservation strategy is one that also tackles climate change.

The global network of coral species’ associations with Symbiodiniaceae and associated thermal thresholds is nonrandom, and this architecture leads to a higher sensitivity to environmental perturbations. Associations, not species, stabilize an ecosystem when it is perturbed, unless those associations are susceptible to a certain stressor, as is the case for coral reefs. Our novel resistance metric can be adapted for different environmental stressors and ecosystems. Resistance is just the first step in a complex response to environmental change. Resilience can be defined as a complex system’s ability to adjust activity when faced with disturbances or stress and recover to a functional state of persistence. Network models of resilience (Gao et al. 2016) are a rapidly developing field. The coral–symbiont network provides a prime test‐bed for resilience metrics, as symbiont populations within corals can shift during and after bleaching events (Glynn et al. 2001, Jones et al. 2008, Sampayo et al. 2016) and the change in interaction patterns may be an adaptive action to increase thermal tolerance (Buddemeier and Fautin 1993, Baker 2003). Future network analyses of resilience combined with our study of network resistance and robustness of global coral‐Symbiodiniaceae associations provide a new trajectory for the conservation of coral reefs under attack by climate change.

## Data Availability

Data are available on Zenodo: https://doi.org/10.5281/zenodo.3595582
