# Supplementary material for: Resistance and robustness of the global coral–symbiont network
Source: Ecology. 2020 Feb 14;101(5):e02990. doi: 10.1002/ecy.2990 (PMC7317464; doi:10.1002/ecy.2990)
Supplement: Supplementary file 1 [file ECY-101-e02990-s001.pdf]

Supporting Information. Williams, S.D., and M.R. Patterson. 2020. Resistance and robustness of the global coral–symbiont network. Ecology.

Appendix S1: Supplementary tables and figures for the network dataset. Description of the degree distribution fitting procedure, the host-specific exploratory network model, and the generalized global network model.

Table S1: Descriptive statistics for each network.

Table S2: Degree and tolerance values for the network hubs.

Table S3: Highest degree nodes of the global network, host-specific network, and generalized global network.

Table S4: Degree distribution fit results.

Figure S1: Distribution of symbiont thermal tolerances adapted from Swain et al. (2017).

Figure S2: Response of the host-specific networks to the bleaching model.

Figure S3: Cumulative degree distributions of the global combined, host, symbionts, generalized, and host-specific networks.

Spatial scale	All Nodes	Hosts	Symbionts	Links	Average Degree	Connectance (Links/HS)	Web Asymmetry $\frac{hosts-symbionts}{hosts+symbionts}$	$T_{MMM(2005)}$ (°C)
Global	*935*	*685*	*250*	*1697*	*3.63*	*0.010*	*0.465*	-
**Pacific**	403	277	126	624	3.1	*0.018*	0.375	-
Great Barrier Reef	205	157	48	315	3.07	*0.042*	0.532	28.68
Central Pacific	97	30	67	147	3.03	*0.073*	-0.381	27.60
Japan	75	58	17	84	2.24	*0.085*	0.547	28.54
Eastern Pacific	27	14	13	43	3.19	*0.236*	0.037	27.63
Western Pacific*	37	16	21	24	-	-	-	29.56
American Samoa*	13	2	11	11	-	-	-	29.87
**Indian**	343	269	74	731	4.26	*0.037*	0.569	-
Phuket	169	140	29	404	4.78	*0.100*	0.657	30.38
Western Indian	144	109	35	272	3.78	*0.071*	0.515	28.97
Western Australia	48	20	28	55	2.29	*0.098*	-0.167	28.78
**Caribbean**	220	139	81	342	3.11	*0.030*	0.264	-
Western Caribbean	93	52	41	122	2.62	*0.057*	0.118	29.72
Eastern Caribbean	80	40	40	106	2.65	*0.066*	0	29.63
Central Caribbean	67	31	36	75	2.24	*0.067*	-0.075	29.94
Florida*	29	15	14	38	-	-	-	30.25
Gulf of Mexico*	2	1	1	1	-	-	-	30.54

TABLE S1. Descriptive statistics for each spatial scale network used in the resistance and robustness models. Spatial scales with (*) had less than 40 links and were not used as their own networks in the resistance and robustness models. Spatial scales in bold denote the global and main oceans. 'All Nodes' gives the size of the network, while 'Hosts' and 'Symbionts' are the number of corals and Symbiodiniaceae in the network, respectively. 'Links' is the number of associations in the network. 'Average Degree' is the average number of associations per node in the network. 'Connectance' (links/hosts*symbionts) is a measure of the realized number of associations out of all possible associations in the network. 'Web Asymmetry'((hosts-symbionts) / (hosts+symbionts)), identifies the relative abundance of corals compared to symbionts in the network; a positive web asymmetry indicates more hosts than symbionts.

Symbiont Type	Number of Associations	Thermal Tolerance
C3	335	0.5097
C1	165	0.4660
C3u	142	0.4685
D1	111	0.6620
D1a	110	0.4190

TABLE S2. Symbiodiniaceae ITS2 phylotypes in the global network with more than 100 associations (degree) and their thermal tolerances ($\tau_{symbiont}$) used in Eq. 1.

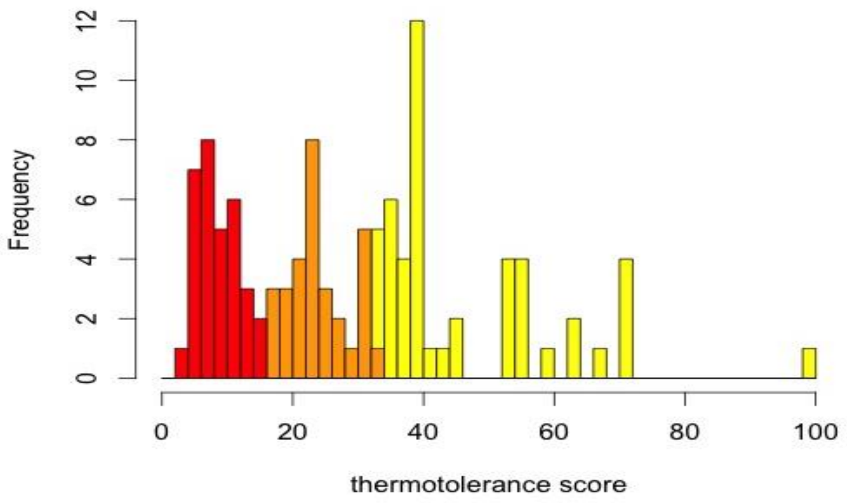

FIGURE S1. Frequency distribution of *Symbiodinium* thermal tolerance scores adapted from Swain et al. (2017); distribution is colored by tolerance range, red is highly susceptible, orange is medium tolerance, and yellow is high tolerance. Swain et al. (2017) provides a framework for a consensus of *Symbiodinium* thermotolerance ranks developed from rank-aggregation methods. Their ranking scheme orders *Symbiodinium* phylotypes from 0-100, but the rank values are not indicative of total magnitude differences in thermotolerance. To determine tolerances of the unlisted symbiont types in our network, rank values were randomly drawn from the high, medium, and low thermal tolerance frequency distributions in the relative proportions of the clades represented in those distributions. Thus, for each simulation of either the bleaching or different removal models described below, the symbiont tolerances varied within a set distribution (Figure 1D). Code for the symbiont thermal tolerances is in Data S2.

HOST-SPECIFIC EXPLORATORY NETWORK MODEL

We tested the effect of greater host-specificity on the structure of the network and thus the resistance to temperature stress by creating a new exploratory model of host-specificity. In this host-specific network the C3, C1, B1, and D1 symbiont nodes were split into their respective degree numbers of new nodes to simulate the most host-specific version of those hubs. The tolerances for these now split-hubs were assigned from a random uniform distribution (0-1) to account for greater variation, and these were regenerated for each model simulation.

We note that this is very much an exploratory model due to the following qualifications: 1) we don’t know what proportion of host species really do have a specific genetic variant of the symbiont hubs, 2) we don’t know exactly how many symbiont ITS2 types are actually many different genetic variants, and 3) we don’t have specific thermal tolerance values for these genetic variants. These same qualifications are why we decided not to make a more complicated model for this host-specific network.

The degree distribution of the host-specific network is shown in Figure S3, and it is still heterogeneous (best fit by a truncated power law) as supported by Table S4. We ran the bleaching model on this host specific network (Figure S2). Visually, there is not a difference between the original networks and the host-specific network, thus we did not continue with the rest of the resistance and robustness analyses.

The bleaching model removes links and tracks the percent of bleached hosts– the number of links and hosts do not change by splitting the symbiont hubs. The only thing really changing in the bleaching model with the host-specific network is the tolerance values of the split hub nodes. The tolerance values of these hub nodes were already about 0.5 which would be the most likely value under the uniform distribution, so even with a few of them being much lower or much higher, the tolerances of these split nodes would not really vary far from the original value. Thus, we end up with a bleaching response similar to the original networks.

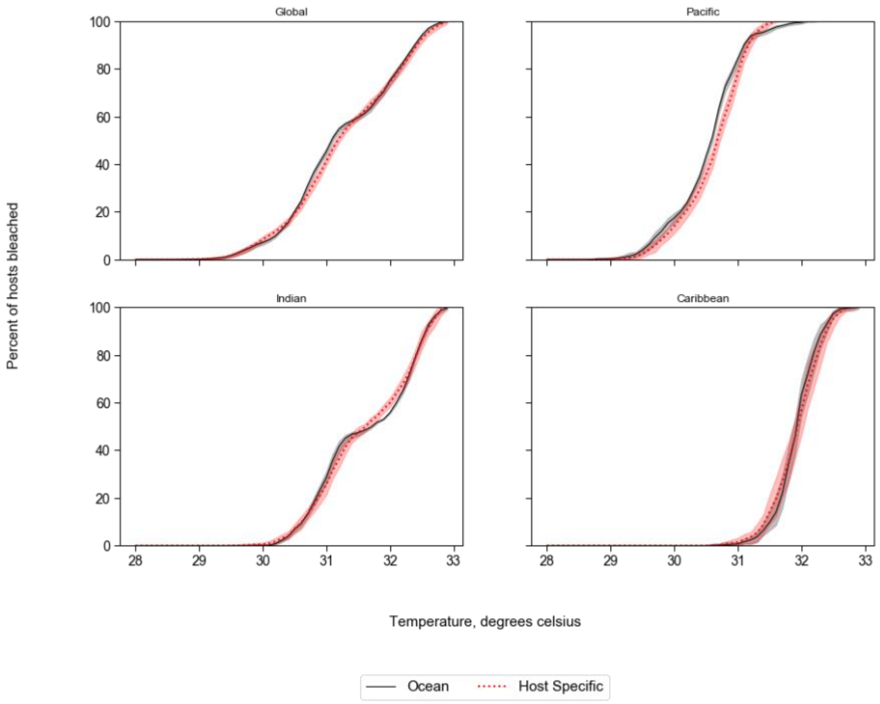

Figure S2. Results of the bleaching model on the original networks and the host-specific networks at the global, Pacific Ocean, Indian Ocean, and Caribbean Sea scales.

THE GENERALIZED GLOBAL NETWORK

To test if the structure is an artefact of the network construction where hosts are included as multiple nodes when they are found in different subregions, we created a generalized global network where both host species and symbiont types were only included once. This generalized network no longer contains environmental or geographical information. Instead, it represents all potential associations among coral hosts and their symbionts on a global scale regardless of any spatial influence on association patterns. This generalized network that is not geographically restricted has a heterogeneous degree distribution that is best fit by a truncated power law (Figure S3 and Table S4). Additionally, the symbiont hubs of this generalized network have degrees on the same order of magnitude as their original global network counterparts (Table S3). Since the global network still has a heterogeneous structure when it is in this generalized form, we argue that its structure is not an artefact of the node inclusion parameters, and thus is not trivial.

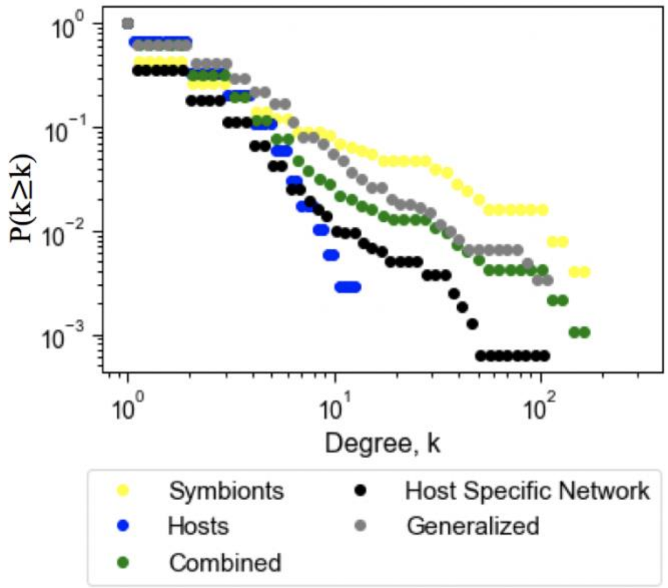

Figure S3. The cumulative degree distributions of the global network, symbionts of the global network, hosts of the global network, host-specific global network, and the generalized global network.

Original Global Network		Generalized Global Network		Host Specific Global Network	
C3	335	C3	208	C3u	142
C1	165	C1	118	D1a	110
C3u	142	C3u	114	C15	47
D1	111	D1a	88	C21	43
D1a	110	D1	85	C3h	39
B1	53	C21	41	C1b	36
C15	47	C3h	39	C3z	35
C21	43	C3z	33	C1c	28
C3h	39	C15	30	C101	28
C1b	36	C1b	30	*Montipora capitata*	18

Table S3. Top 10 highest degree nodes and their degree for three versions of the global network. Note that all major hubs have the same order of magnitude degree

FITTING DEGREE DISTRIBUTIONS

We used the function ‘fit.distribution_compare’ from the ‘powerlaw’ package (Alstott et al., 2014) in Python to determine the best fit for the degree distributions (Table S4). The loglikelihood ratio between two candidate distributions is positive if it is the first candidate and negative if the data is more likely the second distribution. The significance value for the direction is given by p. We tested power law, truncated power law, and exponential functions for the fit comparisons.

Network/node type	Fit comparisons	Loglikelihood ratio	Significance value
Original Global	Power law vs. **truncated**	-2.037	0.044*
	Exponential vs **truncated**	-315.806	0.001**
Global Hosts	Power law vs. truncated	-0.066	0.716
	Exponential vs truncated	-0.989	0.437
Global Symbionts	Power law vs. truncated	-0.212	0.515
	Exponential vs **truncated**	-96.132	3.032e-6***
Generalized Global Network	Power law vs. truncated	-0.032	0.801
	Exponential vs **truncated**	-76.557	0.001**
Host-specific global network	Power law vs. **truncated**	-2.460	0.027*
	Exponential vs **truncated**	-147.330	0.010*
Pacific Ocean Network	Power law vs. truncated	-0.091	0.670
	Exponential vs **truncated**	-20.727	0.017*
Indian Ocean Network	Power law vs. truncated	-1.073	0.143
	Exponential vs **truncated**	-155.247	0.002**
Caribbean Sea Network	Power law vs. truncated	-0.195	0.533
	Exponential vs **truncated**	-15.192	0.046*

Table S4. Results of statistical tests of fits to the degree distributions. Significant differences are noted by * and best fit distributions are bolded for each network/node type. If the fits are not significantly different, the distribution indicated by the loglikelihood value is italicized.

Literature Cited

Alstott J, Bullmore E, Plenz D. (2014). Powerlaw: A Python Package for Analysis of Heavy Tailed Distributions. PLoS ONE 9(1): e85777. doi:10.1371/ journal.pone.0085777

Swain T. D., Chandler J., Backman V., Marcelino L. (2017). Consensus thermotolerance ranking for 110 *Symbiodinium* phylotypes: an exemplar utilization of a novel iterative partial-rank aggregation tool with broad application potential. Functional Ecology. doi: 10.1111/1365-2435.12694
