# Supplementary material for: Resistance and robustness of the global coral–symbiont network
Source: Ecology. 2020 Feb 14;101(5):e02990. doi: 10.1002/ecy.2990 (PMC7317464; doi:10.1002/ecy.2990)
Supplement: Supplementary file 2 [file ECY-101-e02990-s002.pdf]

Supporting Information. Williams, S.D., and M.R. Patterson. 2020. Resistance and robustness of the global coral–symbiont network. Ecology.

Appendix S2: Detailed description of the statistics (randomization tests), high and low degree removal model robustness results, relationship between robustness and connectance, and a note on robustness separately determined for hosts and symbionts (host and symbiont robustness scenarios).

Table S1: Results of Kendal coefficient of rank correlation test of robustness and connectance.

Figure S1: Robustness results of the global and major ocean basins including the degree removals.

Figure S2: Results of the robustness removal model simulations on the global network split by robustness scenario (total, host, symbiont).

RANDOMIZATION TESTS

Values of resistance or robustness (R50) for two groups were first compared using a Two-way ANOVA (Vegan package, R) to obtain the F-statistic. A null distribution of F-statistics was created by randomizing the two groups (shuffled labels and values) 1000 times. The P-value was then taken to be the number of randomizations that generated a F-statistic greater than the obtained F-statistic divided by the number of simulations. P-values were adjusted to account for multiple comparisons using the Holm correction. R Code for a general case of the randomization test is in Data S2. All randomization test results are located in Data S2, see Metadata S2 for file descriptions.

HIGH AND LOW DEGREE REMOVAL MODELS FOR ROBUSTNESS

Nodes were removed from the network according to their degree. Nodes were removed in order of both high and low degree. Nodes with the same degree were randomized in each simulation of the removal model so that the order of node removals was varied in each simulation but still followed the degree removal rule.

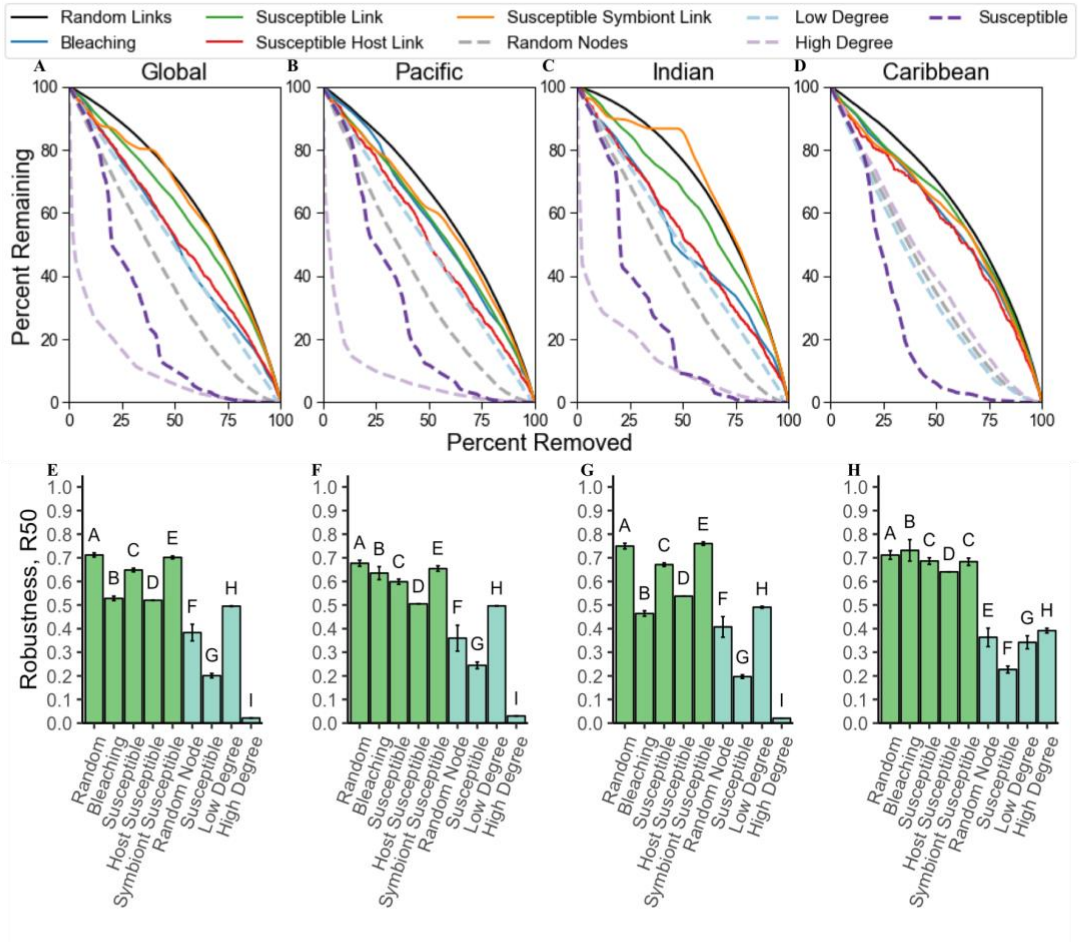

Figure S1. Results, percent of nodes remaining as a function of percent nodes or links removed, of the removal models (mean curve of all 100 simulations) for the global (A), Pacific (B), Indian (C), and Caribbean (D) networks. Robustness (R50) is measured as the fraction of nodes or links

removed needed to decrease the number of nodes in the network by half, with error bars representing the standard deviation for the global (E), Pacific (F), Indian (G), and Caribbean (H) networks. Letters above the error bars signify significant differences determined by the randomization tests described in detail above. Link-removal models are colored green and node-removal models are colored blue-green.

According with other studies on the robustness of networks to node removals (Albert et al. 2000, Dunne et al. 2002), removing low degree nodes results in the highest R50 value of the node removals (0.495 ± 0.001) and removing high degree nodes results in the lowest R50 value of the node removals (0.021 ± 0.000).

RELATIONSHIP BETWEEN ROBUSTNESS AND CONNECTANCE

Dunne et al. (2002) found that robustness (R50 values, described in main text) of food webs significantly increases with connectance (links/species^2) of the networks when nodes were removed in order of most connected nodes, most connected nodes with no basal deletions, and randomly. Robustness decreased with connectance when least connected nodes were removed, though not significantly. The authors determined the relation between robustness and connectance by using simple linear regressions.

The connectance of the networks studied here range from 0.01 to 0.236 and do not have a normal distribution. Thus, we used a nonparametric test for association, Kendall's coefficient of rank (τ) correlation test. The test was performed in R using the cor.test(method = "Kendall") function. Significant correlations are bolded and were determined using a Bonferroni-corrected value of P = 0.0056 (0.05/9). Code for this statistical test is in Data S2.

Removal Model	τ	P
Random links	0.143	0.518
Bleaching	0.473	0.019
Susceptible links	0.209	0.331
Susceptible host links	0.231	0.279
Susceptible symbiont links	-0.077	0.747
Random nodes	0.033	0.915
Susceptible nodes	0.231	0.279
Low degree nodes	-0.407	0.047
**High degree nodes**	**0.582**	**0.003**

TABLE S1. Results of Kendall coefficient of rank correlation test to determine the relationship between network robustness (R50 value) and connectance (#links/(#hosts*#symbionts).

Only the relationship of robustness under the high degree removal model with increasing connectance is significant. As connectance increases, robustness to nodes removed in order of low to high degree decreases, though not significantly.

HOST AND SYMBIONT ROBUSTNESS SCENARIOS

R50 values can be determined separately for the total network (all nodes considered, and all nodes removed), hosts (just coral host nodes considered and only symbiont nodes were removed in the node removal models), and symbiont nodes (just symbiont nodes were considered and only host nodes were removed in the node removal models).

In the host (Figure S1 B&E) and symbiont (Figure S1 C&F) robustness scenarios, the same result patterns as the total robustness scenario are seen when links are removed. Hosts and symbionts are significantly (hosts $P < 0.001$; symbionts $P < 0.001$; Data S2) more robust when links are removed randomly (Host R50 = 0.721±0.008; Symbiont R50=0.677±0.027) than when links are removed by bleaching (Host R50 = 0.520±0.008; Symbiont R50=0.541±0.007). Within the node removals on the host and symbiont robustness scenarios, the result patterns are similar to those in the total robustness scenario, but the magnitude of the R50 values increase. When host or symbiont robustness are considered separately, they are more robust to node removals (host scenario $P = 0.002$; symbiont scenario $P < 0.001$; Data S2).

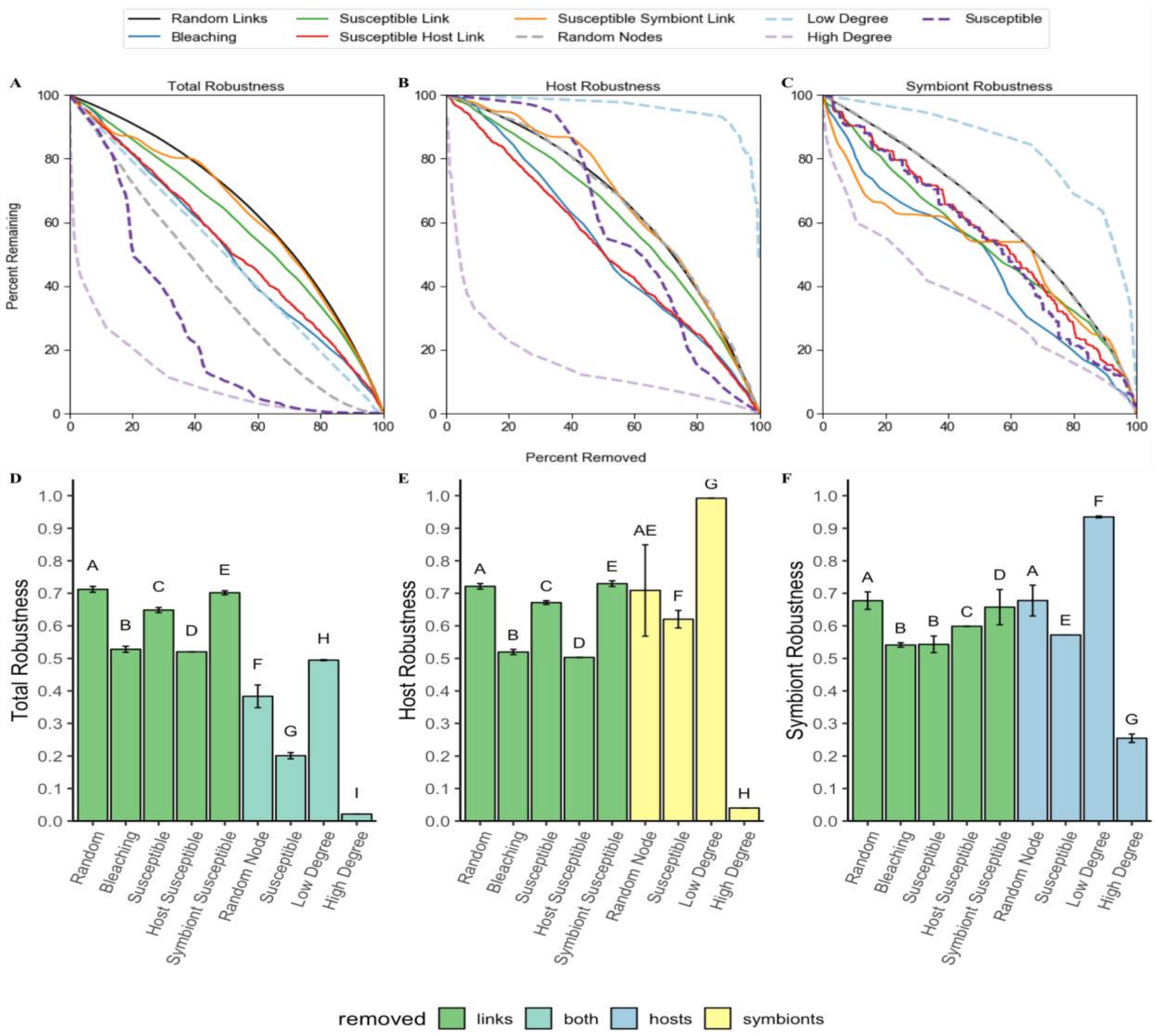

FIGURE S2. Results, percent of nodes remaining as a function of percent nodes or links removed, of the removal models (mean curve of all 100 simulations) for the Total (A), Host (B), and Symbiont (C) robustness scenarios of the global network. Robustness (R50), measured as the fraction of nodes or links removed needed to decrease the number of nodes in the network by half, values with error bars representing the standard deviation for the Total (A), Host (B), and Symbiont (C) robustness scenarios of the global network. Letters above the error bars signify significant differences determined by the randomization tests described in detail in Appendix S2.

Literature Cited

Albert, R., H. Jeong, and A-L. Barabási. 2000. Error and attack tolerance of complex networks. Nature 406:378–382.

Dunne J. A., R. J. Williams, and N. D. Martinez. 2002. Network structure and biodiversity loss in food webs: robustness increases with connectance. Ecology Letters 5:558–567.
