# Supplementary material for: Resistance and robustness of the global coral–symbiont network
Source: Ecology. 2020 Feb 14;101(5):e02990. doi: 10.1002/ecy.2990 (PMC7317464; doi:10.1002/ecy.2990)
Supplement: Supplementary file 4 [file ECY-101-e02990-s004.zip › DataS2/BleachingModel_Resistance_Py27.pdf]

Bleaching Removal Model and Resistance

A weighted link removal model to simulate coral bleaching on networks of coral species and Symbiodineacea ITS2 types (In article text, EQ 1). Each link is given a weight based on how much temperatures stress a certain coral-symbiont association can tolerate before the symbiotic interaction is broken. The weight serves as an association-specific temperature threshold. The bleaching model ramps up environmental temperature and links are removed when their weight is exceeded, and a coral species is considered bleached once it is isolated, i.e., it has no more links to symbionts. The bleaching model was applied to the natural networks (global, ocean-basins, and subregions) and compared to four null networks created for each. These null networks are listed below and change either the thermal tolerance distribution or association structure of the networks to address the first objective of this research.

In [1]:

```
import os #to use os.chdir
#path = "" #change to your specific path
path = "/Users/saradellwilliams/Dropbox/Williams_Suppmat_obj2/Updated8_2017/Code/Python/Inputs/"
os.chdir( path ) #set directory
%pwd #output current directory
```

Out[1]:

```
u'/Users/saradellwilliams/Dropbox/Williams_Suppmat_obj2/Updated8_2017/Code/Python/Inputs'
```

In [2]:

```
import sys
print (sys.version)
```

```
2.7.16 |Anaconda, Inc.| (default, Mar 14 2019, 16:24:02)
[GCC 4.2.1 Compatible Clang 4.0.1 (tags/RELEASE_401/final)]
```

In [3]:

```
#import all of the packages that you will need to run the rest of the
code
import numpy as np
import networkx as nx
import matplotlib.pyplot as plt
import matplotlib
import scipy.cluster.hierarchy as hierarchy
import math
from pylab import figure
import pandas as pd
from scipy.optimize import curve_fit
import scipy as scipy
from scipy import stats
%matplotlib inline
# Font preferences:
matplotlib.rc('xtick', labelsize=14)
matplotlib.rc('ytick', labelsize=14)
matplotlib.rc('font', **{'family':'sans-serif','sans-serif':['Arial']}
)
matplotlib.rc('font', size=16)
matplotlib.rc('xtick.major', size=6, width=1)
matplotlib.rc('xtick.minor', size=3, width=1)
matplotlib.rc('ytick.major', size=6, width=1)
matplotlib.rc('ytick.minor', size=3, width=1)
matplotlib.rc('axes', linewidth=1)
```

My custom functions for creating networks from csvs, calculating network stats, the bleaching model, and running multiple simulations of the bleaching model

In [4]:

```
def mygraph(hosts,edges,tols): #this function creates a graph object f
rom nodes,edges, and missing tolerances files

    x=nx.Graph() #create empty graph
    #get all the data imported
    hostnodes = pd.read_csv(hosts)
    symbnodesgood=pd.read_csv('Global_symbiontgood_nodes.csv') #the sy
mbionts that had tolerances listed in Swain et al. 2016a
    symbnodesrest=pd.read_csv('Global_symbiontbad_nodes.csv') #the one
s that didn't

    fittols=pd.read_csv(tols)
    #combine the tolerance file with the symbionts that need tolerance
s
    symbnodesrest['tols']=fittols['tolerance']
    #Add nodes into the graph with their attributes
    for row in hostnodes.iterrows():
        x.add_node(row[1][0], ocean=row[1][1], name=row[1][2],type=row
[1][3],genetic=row[1][4], tolerance=row[1][5])
```

```
    for row in symbnodesgood.iterrows():
        x.add_node(row[1][0], name=row[1][1],type=row[1][2],genetic=ro
w[1][3], tolerance=row[1][4])
    for row in symbnodesrest.iterrows():
        x.add_node(row[1][0], name=row[1][1],type=row[1][2],genetic=ro
w[1][3], tolerance=row[1][4])

    #now for the edges
    edges = pd.read_csv(edges,header=None) #ordered by ocean and then
by region in alphabetical order
    edge_list=[] #an empty list of edges
    thresh_list=[] #an empty list of thresholds
    for row in edges.iterrows():
        s=row[1][0] #symbiont ID is in first column
        h=row[1][1] #Host ID is n second column
        MMM=row[1][2] #third column is the mean monthly max temperatur
e
        #get the tolerance values from the node attributes
        symb_tol=x.node[s]['tolerance']
        host_tol=x.node[h]['tolerance']
        #calculate the threshold for each edge based on node pairs
        threshvalue=(MMM+(1.5*(symb_tol+host_tol)))
        thresh_list.append(threshvalue)
        #update the edge list with a weight determined by thresholds
        edge_list.append((s,h,{'weight':threshvalue}))

    x.add_edges_from(edge_list) #add edges to the graph object
    x.remove_nodes_from(nx.isolates(x)) #there's a few nodes that dont
actually have edges, and this was easier than going back through the o
riginal excel

    return x

def getstats(x): #this function calculates key network statistics for
a graph

    xN = x.number_of_nodes() #number of nodes
    xL = x.number_of_edges() #number of edges
    C = sorted(nx.connected_components(x), key=lambda c: len(c), rever
se=True)
    C = [len(c) for c in C] ,#components
    degrees = x.degree().values() #list of degrees
    kmin = min(degrees) #min degree
    kmax = max(degrees) #max degree
    #used for graphing degree distribution
    bin_edges = np.logspace(np.log10(kmin), np.log10(kmax), num=0.2*km
ax)
    density, _ = np.histogram(degrees, bins=bin_edges, density=True)
    return xN, xL, x, C, degrees, kmin, kmax, bin_edges, density,_

#Here is the bleaching model: takes a graph object and returns the siz
e of the largest connected component,
#the number of nodes removed, and the list of isolated hosts at each t
```

```
emperature step
def bleaching(G):
    #create a bunch of empty lists
    N_g1= []
    nodesremoved=[]
    iso=[]

    for i in xrange(0,50): #over the range of the temperature steps, d
o the following:
        G1=G.copy() #as a precaution, copy the graph so that you dont
actually change the original one
        T=28+0.1*i #for each step, the change of T is 0.1
        for j in G1.edges(): #look over the edges
            if G1.edge[j[0]][j[1]]['weight']<=T: #when T exceeds the t
hreshold, remove link
                G1.remove_edge(j[0],j[1])
        C=nx.connected_components(G1) #recalculate the connected compo
nents
        isolated_nodes=0 #Calculate the isolated nodes
        for m in C: #look over the components
            if len(m)==1: #if the component is just 1 node
                if m[0]<=730: #dont count the symbiont, just add the h
ost nodes whose ID#s go up to 730
                    isolated_nodes=isolated_nodes+1 #update the isolat
ed nodes size
        iso.append(isolated_nodes) #update isolated host nodes list
        N_g1.append(max(map(len, nx.connected_components(G1)))) #recal
culate the giant component size
        isolates=nx.isolates(G1) #all the isolated nodes
        G1.remove_nodes_from(isolates) #remove the isolated nodes from
the network
        nodesremoved.append(len(isolates)) #total number of nodes remo
ved, including symbionts


    return N_g1,nodesremoved, iso
#run multiple simulations of the bleaching model
def Mult_sims_bleaching(sims,hosts,edges):
    results_isos=np.zeros((50,sims)) #need a blank matrix of colnum=si
ms and row num=number of temperature steps which is 50
    results_nodes=np.zeros((50,sims))
    results_GC=np.zeros((50,sims))
    for i in xrange(0,sims): #for all the sims do the following
        tols="trial" + str(i+1) + ".csv" #call the right tolerance fil
e
        graph=mygraph(hosts,edges,tols) #make network
        GC, nodes, iso = bleaching(graph) #run bleaching model
        results_isos[:,i]=iso #update the matrix
        results_nodes[:,i]=nodes
        results_GC[:,i]=GC
    return results_isos, results_nodes, results_GC
```

Let's take a look at the Global Network

In [5]:
```
globnet=mygraph("Global_host_nodes.csv","Global_edges.csv","trial1.csv
")
#get network info
G=globnet
xN, xL, x, C, degrees, kmin, kmax, bin_edges, density,_ =getstats(G)

print xN, "nodes"
print xL, "links"
print kmin, "min degree"
print kmax, "max degree"
```

```
935 nodes
1697 links
1 min degree
335 max degree
```

Now run 100 Simulations of the bleaching model

In [12]:
```
numsims=100
alltheisos,allremoved,allGC=Mult_sims_bleaching(numsims,"Global_host_n
odes.csv","Global_edges.csv")
alltheisos_caribbean,allremoved_C,allGC_C=Mult_sims_bleaching(numsims,
'Global_host_nodes.csv','Caribbean_edges.csv')
alltheisos_indian,allremoved_I,allGC_I=Mult_sims_bleaching(numsims,'Gl
obal_host_nodes.csv','Indian_edges.csv')
alltheisos_pacific,allremoved_P,allGC_P=Mult_sims_bleaching(numsims,"G
lobal_host_nodes.csv","Pacific_edges.csv")

alltheisos_cc,allremoved_cc,allGC_cc=Mult_sims_bleaching(100,'Global_h
ost_nodes.csv','Central_Caribbean_edges.csv')
alltheisos_cp,allremoved_cp,allGC_cp=Mult_sims_bleaching(100,'Global_h
ost_nodes.csv','Central_Pacific_edges.csv')
alltheisos_ec,allremoved_ec,allGC_ec=Mult_sims_bleaching(100,'Global_h
ost_nodes.csv','Eastern_Caribbean_edges.csv')
alltheisos_ep,allremoved_ep,allGC_ep=Mult_sims_bleaching(100,'Global_h
ost_nodes.csv','Eastern_Pacific_edges.csv')
alltheisos_gbr,allremoved_gbr,allGC_gbr=Mult_sims_bleaching(100,'Globa
l_host_nodes.csv','GBR_edges.csv')
alltheisos_j,allremoved_j,allGC_j=Mult_sims_bleaching(100,'Global_host
_nodes.csv','Japan_edges.csv')
alltheisos_ph,allremoved_ph,allGC_ph=Mult_sims_bleaching(100,'Global_h
ost_nodes.csv','Phuket_edges.csv')
alltheisos_wa,allremoved_wa,allGC_wa=Mult_sims_bleaching(100,'Global_h
ost_nodes.csv','Western_Australia_edges.csv')
alltheisos_wc,allremoved_wc,allGC_wc=Mult_sims_bleaching(100,'Global_h
ost_nodes.csv','Western_Caribbean_edges.csv')
alltheisos_wi,allremoved_wi,allGC_wi=Mult_sims_bleaching(100,'Global_h
ost_nodes.csv','Western_Indian_edges.csv')
```

Custom Functions for determining the mean of the bleaching results, the confidence interval, and getting the temperature steps for plotting

In [13]:
```
def mymean(isos):
    totalhost=max(isos[:,1])
    newiso = (isos/totalhost)*100
    mean=np.mean(newiso,axis=1)
    return mean

def myconfint(isos,high,low): #use 97,3
    totalhost=max(isos[:,1])
    newiso = (isos/totalhost)*100
    high_int=np.percentile(newiso,high,1)
    low_int=np.percentile(newiso,low,1)
    return high_int, low_int

def mytemp(init,stepfinal): #use 28,50
    Temp=[]
    for i in xrange(0,stepfinal):
        T=init+0.1*i
        Temp.append(T)
    return Temp
temp=mytemp(28,50)
```

Null Models:

Random Symbionts:

The symbionts come from the 3 thermal distributions with equal probability. Uses 100 rtrial.csv files from inputs.

Function that creates a graph with tolerance files specified:

In [14]:
```
def mygraph_spectols(hosts,edges,symbtols,hosttols): #this function cr
eates a graph object from nodes,edges, and missing tolerances files

    x=nx.Graph() #create empty graph
    #get all the data imported
    hostnodes = pd.read_csv(hosts)
    symbnodesgood=pd.read_csv('Global_symbiontgood_nodes.csv')
    symbnodesrest=pd.read_csv('Global_symbiontbad_nodes.csv')

    fittols=pd.read_csv(symbtols)
    shuffletols=pd.read_csv(hosttols)
    #combine the tolerance file with the symbionts that need tolerance
s
    symbnodesrest['tols']=fittols['tolerance']
    hostnodes['tolerance']=shuffletols['shuffled']
    #Add nodes into the graph with their attributes
    for row in hostnodes.iterrows():
        x.add_node(row[1][0], ocean=row[1][1], name=row[1][2],type=row
[1][3],genetic=row[1][4], tolerance=row[1][5])
    for row in symbnodesgood.iterrows():
        x.add_node(row[1][0], name=row[1][1],type=row[1][2],genetic=ro
w[1][3], tolerance=row[1][4])
    for row in symbnodesrest.iterrows():
        x.add_node(row[1][0], name=row[1][1],type=row[1][2],genetic=ro
w[1][3], tolerance=row[1][4])

    #now for the edges
    edges = pd.read_csv(edges,header=None)
    edge_list=[] #an empty list of edges
    thresh_list=[] #an empty list of thresholds
    for row in edges.iterrows():
        s=row[1][0] #symbiont ID is in first column
        h=row[1][1] #Host ID is n second column
        MMM=row[1][2] #third column is the mean monthly max temperatur
e
        #get the tolerance values from the node attributes
        symb_tol=x.node[s]['tolerance']
        host_tol=x.node[h]['tolerance']
        #calculate the threshold for each edge based on node pairs
        threshvalue=(MMM)+(1.5*(symb_tol+host_tol))
        thresh_list.append(threshvalue)
        #update the edge list with a weight determined by thresholds
        edge_list.append((s,h,{'weight':threshvalue}))

    x.add_edges_from(edge_list) #add edges to the graph object
    x.remove_nodes_from(nx.isolates(x)) #there's a few nodes that dont
actually have edges, and this was easier than going back through the o
riginal excel

    return x
```

Funtion to create the null network with random tolerances from a random uniform distribution:

In [15]:
```
def myrandgraph(nodes,edges): #this function creates a graph object fr
om nodes,edges, and missing tolerances files
    #AND assigns tolerances from a random uniform distribution

    x=nx.Graph() #create empty graph
    #get all the data imported
    nodes = pd.read_csv(nodes)

    #Add nodes into the graph with their attributes
    for row in nodes.iterrows():
        x.add_node(row[1][0], ocean=row[1][1], name=row[1][2],type=row
[1][3],genetic=row[1][4])

    #now for the edges
    edges = pd.read_csv(edges,header=None)
    edge_list=[] #an empty list of edges
    thresh_list=[] #an empty list of thresholds
    for row in edges.iterrows():
        s=row[1][0] #symbiont ID is in first column
        h=row[1][1] #Host ID is n second column
        MMM=row[1][2] #third column is the mean monthly max temperatur
e
        #get the tolerance values from the node attributes
        symb_tol=np.random.random()
        host_tol=np.random.random()
        #calculate the threshold for each edge based on node pairs
        threshvalue=(MMM)+(1.5*(symb_tol+host_tol))
        thresh_list.append(threshvalue)
        #update the edge list with a weight determined by thresholds
        edge_list.append((s,h,{'weight':threshvalue}))

    x.add_edges_from(edge_list) #add edges to the graph object
    x.remove_nodes_from(nx.isolates(x)) #there's a few nodes that dont
actually have edges, and this was easier than going back through the o
riginal excel

    return x
```

The following function simulates bleaching on the random tolerance null networks 100 times and does it for the shuffled symbionts, hosts, hosts and symbionts, or the random uniform model.

In [16]:

```
def Mult_sims_random_bleaching(sims,hosts,edges,shuffletype): #combine it all!!!
    results_isos=np.zeros((50,sims)) #need a blank matrix of colnum=sims and row num=number of temperature steps which is 60
    results_nodes=np.zeros((50,sims))
    results_GC=np.zeros((50,sims))
    if shuffletype=="Symbionts":
        for i in xrange(0,sims): #for all the sims do the following
            symbtols="rtrial" + str(i+1) + ".csv" #call the right tolerance file
            hosttols="hosttolerances_init.csv"
            graph=mygraph_spectols(hosts,edges,symbtols,hosttols) #make graph
            meh, nah, iso = bleaching(graph) #run bleaching model
            results_isos[:,i]=iso #update the giant ass matrix
            results_nodes[:,i]=nah
            results_GC[:,i]=meh
    if shuffletype=="Hosts_and_Symbionts":
        for i in xrange(0,sims): #for all the sims do the following
            symbtols="rtrial" + str(i+1) + ".csv" #call the right tolerance file
            hosttols="hosttolerances"+str(i+1)+".csv"
            graph=mygraph_spectols(hosts,edges,symbtols,hosttols) #make graph
            meh, nah, iso = bleaching(graph) #run bleaching model
            results_isos[:,i]=iso #update the giant ass matrix
            results_nodes[:,i]=nah
            results_GC[:,i]=meh
    if shuffletype=="Hosts":
        for i in xrange(0,sims): #for all the sims do the following
            symbtols="trial" + str(i+1) + ".csv" #call the right tolerance file
            hosttols="hosttolerances"+str(i+1)+".csv"
            graph=mygraph_spectols(hosts,edges,symbtols,hosttols) #make graph
            meh, nah, iso = bleaching(graph) #run bleaching model
            results_isos[:,i]=iso #update the giant ass matrix
            results_nodes[:,i]=nah
            results_GC[:,i]=meh
    if shuffletype=="Random_uniform":
        for i in xrange(0,sims):
            graph=myrandgraph(hosts,edges) #make graph, which randomizes tolerances
            meh, nah, iso = bleaching(graph) #run bleaching model
            results_isos[:,i]=iso #update the results
            results_nodes[:,i]=nah
            results_GC[:,i]=meh
    return results_isos, results_nodes, results_GC
```

The random bipartite null networks, both degree conserving (DC) and non-degree conserving (NDC).

The next two functions get the degree sequence of the hosts and symbiont nodes separately:

In [17]:
```
def hostseq(test):
    degrees = test.degree().values()
    d=nx.degree(test)
    nx.set_node_attributes(test,'degree',d)
    hostdegs=np.zeros((1,731))

    for node in test.nodes():
        if test.node[node]['type']==0 :
                hostdegs[0,node]=test.node[node]['degree']
    hostdegs=hostdegs[hostdegs!=0]
    host_seq=tuple(hostdegs)
    new=[]
    for c in xrange(0,len(host_seq)):
        ugh=host_seq[c]
        why=int(ugh)
        new.append(why)
    host_seq=tuple(new)
    return (host_seq)

def symbseq(test):
    degrees = test.degree().values()
    d=nx.degree(test)
    nx.set_node_attributes(test,'degree',d)
    symbdegs=np.zeros((1,982))
    for node in test.nodes():
        if test.node[node]['type']==1 :
                symbdegs[0,node]=test.node[node]['degree']
    symbdegs=symbdegs[symbdegs!=0]
    symb_seq=tuple(symbdegs)
    new=[]

    for c in xrange(0,len(symb_seq)):
        ugh=symb_seq[c]
        why=int(ugh)
        new.append(why)
    symb_seq=tuple(new)
    return (symb_seq)
```

This function creates the random bipartite null networks (DC and NDC) from the original network:

```
In [18]: def get_biparnull_nets(nodes,edges, symbtols, hosttols,nulltype):
    graph=mygraph(nodes,edges,symbtols) #makes the graph so that you c
an get the hostseq and symbseq
    host_seq=hostseq(graph) #gets degree sequence of hosts
    symb_seq=symbseq(graph) #gets degree sequence of symbionts
    G=nx.Graph()
    #choose which model to run
    if nulltype=="dc":
        x=nx.bipartite_configuration_model(host_seq,symb_seq)
        hostnodes=nx.bipartite.sets(x)[0]
        hostnodes=list(hostnodes)
        symbnodes=nx.bipartite.sets(x)[1]
        symbnodes=list(symbnodes)
    if nulltype=="ndc":
        x=nx.bipartite_gnmk_random_graph(len(host_seq), len(symb_seq),
graph.number_of_edges(), seed=None, directed=False)
        hostnodes=x.nodes()[0:len(host_seq)]
        symbnodes=x.nodes()[len(host_seq):len(x.nodes())]


    symbtols=pd.read_csv(symbtols)
    hosttols=pd.read_csv(hosttols)
    symbtols=symbtols[0:len(symbnodes)]
    hosttols=hosttols[0:len(hostnodes)]
    hosttols['ID']=hostnodes
    symbtols['ID']=symbnodes

    for row in hosttols.iterrows():
        G.add_node(row[1][1], tolerance=row[1][0])
    for row in symbtols.iterrows():
        G.add_node(row[1][1], tolerance=row[1][0])

    edgesMMM = pd.read_csv(edges,header=None)
    edges = x.edges()
    edge_list=[]
    thresh_list=[]
    for i in xrange(0,len(edges)):
        MMM=edgesMMM[2][i]
        s=edges[i][1]
        h=edges[i][0]
        symb_tol=G.node[s]['tolerance']
        host_tol=G.node[h]['tolerance']
        #calculate the threshold for each edge based on node pairs
        threshvalue=(MMM)+(1.5*(symb_tol+host_tol))
        thresh_list.append(threshvalue)
        edge_list.append((s,h,{'weight':threshvalue}))

    G.add_edges_from(edge_list) #add edges to the graph object
    G.remove_nodes_from(nx.isolates(G))
    return G
```

Function to simulate the bleaching model on the random bipartite null networks 100 times:

In [19]:
```
def Mult_sims_randbipar_bleaching(sims,nodes,edges,nulltype):
    results_isos=np.zeros((50,sims))
    results_nodes=np.zeros((50,sims))
    results_GC=np.zeros((50,sims))

    for i in xrange(0,sims): #for all the sims do the following
        #make new random network each time
        G=get_biparnull_nets(nodes,edges,"symbionttolerances_init.csv"
,"hosttolerances_init.csv",nulltype)
        meh, nah, iso = bleaching(G) #run bleaching model
        results_isos[:,i]=iso #update the giant matrix
        results_nodes[:,i]=nah
        results_GC[:,i]=meh
    return results_isos, results_nodes, results_GC
```

In [20]:
```
def null_networks_model_results(hostnodes,edges):
    sims=100
    #get all the nulls done that change tolerance
    isos_hostssymbsrand,removed_hostssymbsrand,GC_hostssymbsrand=Mult_
sims_random_bleaching(100,hostnodes,edges,"Hosts_and_Symbionts")
    isos_rand,removed_rand,GC_rand=Mult_sims_random_bleaching(100,host
nodes,edges,"Random_uniform")
    #get nulls that change structure
    results_isos_ndc, results_nodes_ndc, results_GC_ndc=Mult_sims_rand
bipar_bleaching(sims,hostnodes,edges,"ndc")
    results_isos_dc, results_nodes_dc, results_GC_dc=Mult_sims_randbip
ar_bleaching(sims,hostnodes,edges,"dc")


    return isos_hostssymbsrand,removed_hostssymbsrand,GC_hostssymbsran
d, isos_rand,removed_rand,GC_rand, results_isos_ndc, results_nodes_ndc
, results_GC_ndc,results_isos_dc, results_nodes_dc, results_GC_dc
```

In [21]:
```
Gisos_hostssymbsrand,Gremoved_hostssymbsrand,GGC_hostssymbsrand, Gisos
_rand,Gremoved_rand,GGC_rand, Gresults_isos_ndc, Gresults_nodes_ndc, G
results_GC_ndc, Gresults_isos_dc, Gresults_nodes_dc, Gresults_GC_dc =
null_networks_model_results("Global_host_nodes.csv","Global_edges.csv"
)


Pisos_hostssymbsrand,Premoved_hostssymbsrand,PGC_hostssymbsrand, Pisos
_rand,Premoved_rand,PGC_rand, Presults_isos_ndc, Presults_nodes_ndc, P
results_GC_ndc, Presults_isos_dc, Presults_nodes_dc, Presults_GC_dc =
null_networks_model_results("Global_host_nodes.csv","Pacific_edges.csv
")
Cisos_hostssymbsrand,Cremoved_hostssymbsrand,CGC_hostssymbsrand, Cisos
_rand,Cremoved_rand,CGC_rand, Cresults_isos_ndc, Cresults_nodes_ndc, C
results_GC_ndc, Cresults_isos_dc, Cresults_nodes_dc, Cresults_GC_dc =
```

```
null_networks_model_results("Global_host_nodes.csv","Caribbean_edges.c
sv")


CCisos_hostssymbsrand,CCremoved_hostssymbsrand,CCGC_hostssymbsrand, CC
isos_rand,CCremoved_rand,CCGC_rand, CCresults_isos_ndc, CCresults_node
s_ndc, CCresults_GC_ndc, CCresults_isos_dc, CCresults_nodes_dc, CCresu
lts_GC_dc = null_networks_model_results("Global_host_nodes.csv","Centr
al_Caribbean_edges.csv")
CPisos_hostssymbsrand,CPremoved_hostssymbsrand,CPGC_hostssymbsrand, CP
isos_rand,CPremoved_rand,CPGC_rand, CPresults_isos_ndc, CPresults_node
s_ndc, CPresults_GC_ndc, CPresults_isos_dc, CPresults_nodes_dc, CPresu
lts_GC_dc = null_networks_model_results("Global_host_nodes.csv","Centr
al_Pacific_edges.csv")
ECisos_hostssymbsrand,ECremoved_hostssymbsrand,ECGC_hostssymbsrand, EC
isos_rand,ECremoved_rand,ECGC_rand, ECresults_isos_ndc, ECresults_node
s_ndc, ECresults_GC_ndc, ECresults_isos_dc, ECresults_nodes_dc, ECresu
lts_GC_dc = null_networks_model_results("Global_host_nodes.csv","Easte
rn_Caribbean_edges.csv")
EPisos_hostssymbsrand,EPremoved_hostssymbsrand,EPGC_hostssymbsrand, EP
isos_rand,EPremoved_rand,EPGC_rand, EPresults_isos_ndc, EPresults_node
s_ndc, EPresults_GC_ndc, EPresults_isos_dc, EPresults_nodes_dc, EPresu
lts_GC_dc = null_networks_model_results("Global_host_nodes.csv","Easte
rn_Pacific_edges.csv")
GBRisos_hostssymbsrand,GBRremoved_hostssymbsrand,GBRGC_hostssymbsrand,
GBRisos_rand,GBRremoved_rand,GBRGC_rand, GBRresults_isos_ndc, GBRresul
ts_nodes_ndc, GBRresults_GC_ndc, GBRresults_isos_dc, GBRresults_nodes_
dc, GBRresults_GC_dc = null_networks_model_results("Global_host_nodes.
csv","GBR_edges.csv")
Jisos_hostssymbsrand,Jremoved_hostssymbsrand,JGC_hostssymbsrand, Jisos
_rand,Jremoved_rand,JGC_rand, Jresults_isos_ndc, Jresults_nodes_ndc, J
results_GC_ndc, Jresults_isos_dc, Jresults_nodes_dc, Jresults_GC_dc =
null_networks_model_results("Global_host_nodes.csv","Japan_edges.csv")
PHisos_hostssymbsrand,PHremoved_hostssymbsrand,PHGC_hostssymbsrand, PH
isos_rand,PHremoved_rand,PHGC_rand, PHresults_isos_ndc, PHresults_node
s_ndc, PHresults_GC_ndc, PHresults_isos_dc, PHresults_nodes_dc, PHresu
lts_GC_dc = null_networks_model_results("Global_host_nodes.csv","Phuke
t_edges.csv")
WAisos_hostssymbsrand,WAremoved_hostssymbsrand,WAGC_hostssymbsrand, WA
isos_rand,WAremoved_rand,WAGC_rand, WAresults_isos_ndc, WAresults_node
s_ndc, WAresults_GC_ndc, WAresults_isos_dc, WAresults_nodes_dc, WAresu
lts_GC_dc = null_networks_model_results("Global_host_nodes.csv","Weste
rn_Australia_edges.csv")
WCisos_hostssymbsrand,WCremoved_hostssymbsrand,WCGC_hostssymbsrand, WC
isos_rand,WCremoved_rand,WCGC_rand, WCresults_isos_ndc, WCresults_node
s_ndc, WCresults_GC_ndc, WCresults_isos_dc, WCresults_nodes_dc, WCresu
lts_GC_dc = null_networks_model_results("Global_host_nodes.csv","Weste
rn_Caribbean_edges.csv")
WIisos_hostssymbsrand,WIremoved_hostssymbsrand,WIGC_hostssymbsrand, WI
isos_rand,WIremoved_rand,WIGC_rand, WIresults_isos_ndc, WIresults_node
s_ndc, WIresults_GC_ndc, WIresults_isos_dc, WIresults_nodes_dc, WIresu
lts_GC_dc = null_networks_model_results("Global_host_nodes.csv","Weste
rn_Indian_edges.csv")
```

In [22]:
```
Iisos_hostssymbsrand,Iremoved_hostssymbsrand,IGC_hostssymbsrand, Iisos
_rand,Iremoved_rand,IGC_rand, Iresults_isos_ndc, Iresults_nodes_ndc, I
results_GC_ndc, Iresults_isos_dc, Iresults_nodes_dc, Iresults_GC_dc =
null_networks_model_results("Global_host_nodes.csv","Indian_edges.csv"
)

PHisos_hostssymbsrand,PHremoved_hostssymbsrand,PHGC_hostssymbsrand, PH
isos_rand,PHremoved_rand,PHGC_rand, PHresults_isos_ndc, PHresults_node
s_ndc, PHresults_GC_ndc, PHresults_isos_dc, PHresults_nodes_dc, PHresu
lts_GC_dc = null_networks_model_results("Global_host_nodes.csv","Phuke
t_edges.csv")
```

Need a function to calculate Resistance

We define resistance as the amount of temperature required for the network to go from 10 to 90 percent of hosts bleached normalized by the maximum temperature change for this range (3).

In [27]:
```
def myresistance(isos,edges):
    G=mygraph("Global_host_nodes.csv",edges,"trial1.csv")
    N, L, G, C, degrees, kmin, kmax, bin_edges, density,_ =getstats(G)
    numHosts=len(hostseq(G))
    percent_isos=(isos/numHosts)*100
    Temp=mytemp(28,50)

    temp_10=[] #get the 10% temperature
    jtrack=0
    for j in xrange(0,100): #for each simulation
        for i in xrange(0,49): #for each temp increase step
            if j>jtrack: #so that dont get multiple values per simulat
ion
                if percent_isos[i,j]<=10:
                    if percent_isos[i+1,j]>= 10:
                        temp_10.append(Temp[i])
                        jtrack=j

    temp_90=[] #get the 90% temperature
    ktrack=0
    for k in xrange(0,100): #for each simulation
        for i in xrange(0,49): #for each temp increase step
            if k>ktrack: #so that dont get multiple values per simulat
ion
                if percent_isos[i,k]<=90:
                    if percent_isos[i+1,k]>= 90:
                        temp_90.append(Temp[i])
                        ktrack=k
    #if less than 90% of the corals were bleached at the end of the bl
eaching simulation, then it wont count a temp_90
    #need to add a max temp reading for that missing value. This only
happenned 1x in the indian ocean and 2x in the phuket ocean both in th
e random null model
    if len(temp_90)<99:
        missing=99-len(temp_90)
        for i in xrange(0,missing):
            temp_90.append(Temp[49])

    dtemp=[(x1 - x2)/3 for (x1, x2) in zip(temp_90, temp_10)]


    return temp_10,temp_90,dtemp
```

In [28]:

```
def myresistanceall(net_isos,isos_hostssymbsrand,isos_rand,isos_ndc,is
os_dc,edges):
    net_temp_10, net_temp_90,net_dtemp=myresistance(net_isos,edges)
    hsrand_temp_10, hsrand_temp_90,hsrand_dtemp=myresistance(isos_host
ssymbsrand,edges)
    rand_temp_10, rand_temp_90,rand_dtemp=myresistance(isos_rand,edges
)
    rbndc_temp_10, rbndc_temp_90,rbndc_dtemp=myresistance(isos_ndc,edg
es)
    rbdc_temp_10, rbdc_temp_90,rbdc_dtemp=myresistance(isos_dc,edges)


    a=np.column_stack((net_temp_10, net_temp_90,net_dtemp,hsrand_temp_
10, hsrand_temp_90,hsrand_dtemp,rand_temp_10, rand_temp_90,rand_dtemp,
rbndc_temp_10, rbndc_temp_90,rbndc_dtemp,rbdc_temp_10, rbdc_temp_90,rb
dc_dtemp))
    df=pd.DataFrame(a)
    df.columns=["net_temp_10", "net_temp_90","net_dtemp","hsrand_temp_
10", "hsrand_temp_90","hsrand_dtemp","rand_temp_10", "rand_temp_90","r
and_dtemp","rbndc_temp_10", "rbndc_temp_90","rbndc_dtemp","rbdc_temp_1
0", "rbdc_temp_90","rbdc_dtemp"]
    return df
```

In [31]:

```
g=myresistanceall(alltheisos,Gisos_hostssymbsrand,Gisos_rand,Gresults_isos_ndc,Gresults_isos_dc,"Global_edges.csv")


Carib=myresistanceall(alltheisos_caribbean,Cisos_hostssymbsrand,Cisos_rand,Cresults_isos_ndc,Cresults_isos_dc,"Caribbean_edges.csv")

Pacific=myresistanceall(alltheisos_pacific,Pisos_hostssymbsrand,Pisos_rand,Presults_isos_ndc,Presults_isos_dc,"Pacific_edges.csv")



CC=myresistanceall(alltheisos_cc,CCisos_hostssymbsrand,CCisos_rand,CCresults_isos_ndc,CCresults_isos_dc,"Central_Caribbean_edges.csv")

CP=myresistanceall(alltheisos_cp,CPisos_hostssymbsrand,CPisos_rand,CPresults_isos_ndc,CPresults_isos_dc,"Central_Pacific_edges.csv")


EC=myresistanceall(alltheisos_ec,ECisos_hostssymbsrand,ECisos_rand,ECresults_isos_ndc,ECresults_isos_dc,"Eastern_Caribbean_edges.csv")

EP=myresistanceall(alltheisos_ep,EPisos_hostssymbsrand,EPisos_rand,EPresults_isos_ndc,EPresults_isos_dc,"Eastern_Pacific_edges.csv")


GBR=myresistanceall(alltheisos_gbr,GBRisos_hostssymbsrand,GBRisos_rand,GBRresults_isos_ndc,GBRresults_isos_dc,"GBR_edges.csv")


J=myresistanceall(alltheisos_j,Jisos_hostssymbsrand,Jisos_rand,Jresults_isos_ndc,Jresults_isos_dc,"Japan_edges.csv")


WA=myresistanceall(alltheisos_wa,WAisos_hostssymbsrand,WAisos_rand,WAresults_isos_ndc,WAresults_isos_dc,"Western_Australia_edges.csv")


WC=myresistanceall(alltheisos_wc,WCisos_hostssymbsrand,WCisos_rand,WCresults_isos_ndc,WCresults_isos_dc,"Western_Caribbean_edges.csv")

WI=myresistanceall(alltheisos_wi,WIisos_hostssymbsrand,WIisos_rand,WIresults_isos_ndc,WIresults_isos_dc,"Western_Indian_edges.csv")

Indian=myresistanceall(alltheisos_indian,Iisos_hostssymbsrand,Iisos_rand,Iresults_isos_dc,Iresults_isos_dc,"Indian_edges.csv")

PH=myresistanceall(alltheisos_ph,PHisos_hostssymbsrand,PHisos_rand,PHresults_isos_ndc,PHresults_isos_dc,"Phuket_edges.csv")
```

In [34]:
```
def myresistanceall_summary(net_isos,isos_hostssymbsrand,isos_rand,iso
s_ndc,isos_dc,edges):
    net_temp_10, net_temp_90,net_dtemp=myresistance(net_isos,edges)
    hsrand_temp_10, hsrand_temp_90,hsrand_dtemp=myresistance(isos_host
ssymbsrand,edges)
    rand_temp_10, rand_temp_90,rand_dtemp=myresistance(isos_rand,edges
)
    rbndc_temp_10, rbndc_temp_90,rbndc_dtemp=myresistance(isos_ndc,edg
es)
    rbdc_temp_10, rbdc_temp_90,rbdc_dtemp=myresistance(isos_dc,edges)

    mean_net_dtemp=np.mean(net_dtemp)
    std_net_dtemp=np.std(net_dtemp)

    mean_hsrand_dtemp=np.mean(hsrand_dtemp)
    std_hsrand_dtemp=np.std(hsrand_dtemp)

    mean_rand_dtemp=np.mean(rand_dtemp)
    std_rand_dtemp=np.std(rand_dtemp)

    mean_rbndc_dtemp=np.mean(rbndc_dtemp)
    std_rbndc_dtemp=np.std(rbndc_dtemp)

    mean_rbdc_dtemp=np.mean(rbdc_dtemp)
    std_rbdc_dtemp=np.std(rbdc_dtemp)

    a=np.column_stack((mean_net_dtemp,std_net_dtemp,mean_hsrand_dtemp,
std_hsrand_dtemp,mean_rand_dtemp,std_rand_dtemp,mean_rbndc_dtemp,std_r
bndc_dtemp,mean_rbdc_dtemp,std_rbdc_dtemp))
    df=pd.DataFrame(a)
    df.columns=["mean_net_dtemp","std_net_dtemp","mean_hsrand_dtemp","
std_hsrand_dtemp","mean_rand_dtemp","std_rand_dtemp","mean_rbndc_dtemp
","std_rbndc_dtemp","mean_rbdc_dtemp","std_rbdc_dtemp"]
    return df
```

In [35]:
```
glob=myresistanceall_summary(alltheisos,Gisos_hostssymbsrand,Gisos_ran
d,Gresults_isos_ndc,Gresults_isos_dc,"Global_edges.csv")
glob
```

Out[35]:

	mean_net_dtemp	std_net_dtemp	mean_hsrand_dtemp	std_hsrand_dtemp	mean_rand_dtemp
**0**	0.717172	0.020303	0.721886	0.021795	0.89124[illegible]

In [23]:
```
def mynullsandnetsplot(name,net_isos,randuniform_isos,symbhostsrand_is
os,randbipar_dc_isos,randbipar_ndc_isos, legend,ax):

    Temp=mytemp(28,50)
    net_mean=mymean(net_isos)
    net_high=myconfint(net_isos,97,3)[0]
    net_low=myconfint(net_isos,97,3)[1]
    randuniform_mean=mymean(randuniform_isos)
```

```
    randuniform_high=myconfint(randuniform_isos,97,3)[0]
    randuniform_low=myconfint(randuniform_isos,97,3)[1]
    symbhostsrand_mean=mymean(symbhostsrand_isos)
    symbhostsrand_high=myconfint(symbhostsrand_isos,97,3)[0]
    symbhostsrand_low=myconfint(symbhostsrand_isos,97,3)[1]
    randbipar_dc_mean=mymean(randbipar_dc_isos)
    randbipar_dc_high=myconfint(randbipar_dc_isos,97,3)[0]
    randbipar_dc_low=myconfint(randbipar_dc_isos,97,3)[1]
    randbipar_ndc_mean=mymean(randbipar_ndc_isos)
    randbipar_ndc_high=myconfint(randbipar_ndc_isos,97,3)[0]
    randbipar_ndc_low=myconfint(randbipar_ndc_isos,97,3)[1]

    #plot!
    fig = plt.figure(figsize=(8, 6))
    ax.plot(Temp,net_mean,color='black',label='Ocean',linestyle='-',linewidth=1)
    ax.fill_between(Temp,net_high,net_low,color='grey',alpha=0.5)

    ax.plot(Temp,randuniform_mean,color='#66c2a4',linestyle='--',linewidth=2, label='Random Uniform')
    ax.fill_between(Temp,randuniform_high,randuniform_low,color='#66c2a4',alpha=0.25)

    ax.plot(Temp,symbhostsrand_mean,color='dimgray',linestyle='-',linewidth=2, label='Shuffled Tolerances')
    ax.fill_between(Temp,symbhostsrand_high,symbhostsrand_low,color='dimgray',alpha=0.25)

    ax.plot(Temp,randbipar_dc_mean,color='#238b45',linestyle='-.',linewidth=3, label='Random Bipartite DC')
    plt.fill_between(Temp,randbipar_dc_high,randbipar_dc_low,color='#238b45',alpha=0.25)

    ax.plot(Temp,randbipar_ndc_mean,color='#00441b',linestyle=':',linewidth=2, label='Random Bipartite NDC')
    ax.fill_between(Temp,randbipar_ndc_high,randbipar_ndc_low,color='#00441b',alpha=0.25)


    ax.set_ylim(0,100)
    #ax.set_xlabel('Temperature degrees celsius')
    #ax.set_ylabel('Percent of Hosts Bleached')

    ax.set_title(name)
    #ax.patch.set_facecolor('#cccccc')


    plt.close()
    return fig
```

In [24]:

```
legend="upper left"

fig,((ax1,ax2),(ax3,ax4))=plt.subplots(nrows=2,ncols=2,sharex='col', s
harey='row',figsize=(8,8))
mynullsandnetsplot("Pacific",alltheisos_pacific,Pisos_rand,Pisos_hosts
symbsrand,Presults_isos_dc,Presults_isos_ndc, 'upper left',ax2)
mynullsandnetsplot("Global",alltheisos,Gisos_rand,Gisos_hostssymbsrand
,Gresults_isos_dc,Gresults_isos_ndc, 'upper left',ax1)
mynullsandnetsplot("Indian",alltheisos_indian,Iisos_rand,Iisos_hostssy
mbsrand,Iresults_isos_dc,Iresults_isos_ndc, 'upper left',ax3)
mynullsandnetsplot("Caribbean",alltheisos_caribbean,Cisos_rand,Cisos_h
ostssymbsrand,Cresults_isos_dc,Cresults_isos_ndc, 'upper left',ax4)
fig.text(0.5, 0.01, 'Temperature, degrees celsius', ha='center')
fig.text(0.01, 0.5, 'Percent of hosts bleached', va='center', rotation
='vertical')
legend=plt.legend( loc = 'upper center', bbox_to_anchor = (0.5, -0.05)
,ncol=4,
            bbox_transform = plt.gcf().transFigure ,fontsize=15)
#legend.get_frame().set_facecolor('#cccccc')
```

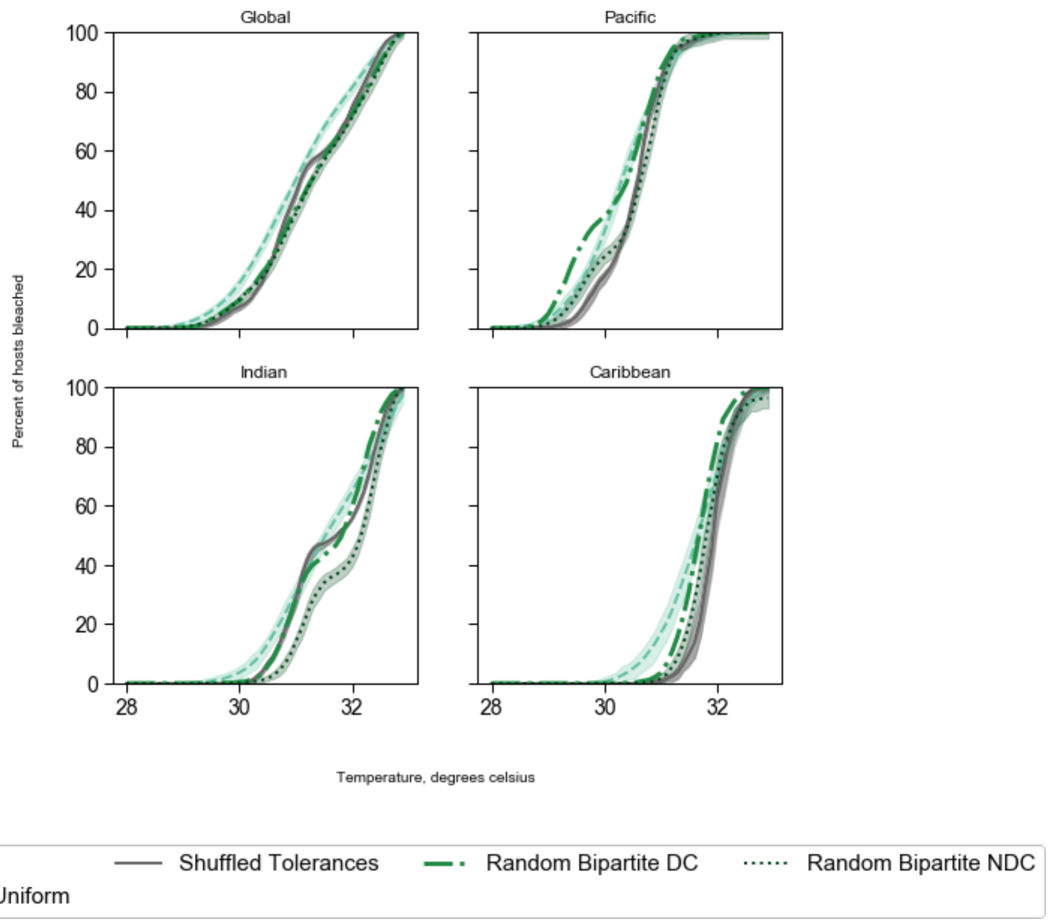

Addressing Host Specificity

In R, I created new nodes for the symbiont hubs, so that the hub is now its degree number of nodes each with only 1 link to a specific host. The tolerances for these split hub nodes are taken from a uniform distribution to account for variation in thermal tolerances.

The hubs that were replaced by specific nodes were C3, C1, B1, and D1.

In [6]:

```
def mygraph_hostspecific(hosts,edges,tols): #this function creates a g
raph object from nodes,edges, and missing tolerances files

    x=nx.Graph() #create empty graph
    #get all the data imported
    hostnodes = pd.read_csv(hosts)
    symbnodesgood=pd.read_csv('Global_symbiontgood_nodes.csv') #the sy
mbionts that had tolerances listed in Swain et al. 2016a
    symbnodesrest=pd.read_csv('Global_symbiontbad_nodes.csv') #the one
s that didn't
    symbnodesspecific=pd.read_csv('HostSpecific_symbnodes.csv')

    fittols=pd.read_csv(tols)
    #combine the tolerance file with the symbionts that need tolerance
s
    symbnodesrest['tols']=fittols['tolerance']
    #Add nodes into the graph with their attributes
    for row in hostnodes.iterrows():
        x.add_node(row[1][0], ocean=row[1][1], name=row[1][2],type=row
[1][3],genetic=row[1][4], tolerance=row[1][5])
    for row in symbnodesgood.iterrows():
        x.add_node(row[1][0], name=row[1][1],type=row[1][2],genetic=ro
w[1][3], tolerance=row[1][4])
    for row in symbnodesrest.iterrows():
        x.add_node(row[1][0], name=row[1][1],type=row[1][2],genetic=ro
w[1][3], tolerance=row[1][4])
    #add host specific
    for row in symbnodesspecific.iterrows():
        x.add_node(row[1][0], name=row[1][1],type=row[1][2],genetic=ro
w[1][3], tolerance=np.random.random())

    #now for the edges
    edges = pd.read_csv(edges,header=None) #ordered by ocean and then
by region in alphabetical order
    edge_list=[] #an empty list of edges
    thresh_list=[] #an empty list of thresholds
    for row in edges.iterrows():
        s=row[1][0] #symbiont ID is in first column
        h=row[1][1] #Host ID is in second column
        MMM=row[1][2] #third column is the mean monthly max temperatur
e
        #get the tolerance values from the node attributes
```

```
        symb_tol=x.node[s]['tolerance']
        host_tol=x.node[h]['tolerance']
        #calculate the threshold for each edge based on node pairs
        threshvalue=(MMM+(1.5*(symb_tol+host_tol)))
        thresh_list.append(threshvalue)
        #update the edge list with a weight determined by thresholds
        edge_list.append((s,h,{'weight':threshvalue}))

    x.add_edges_from(edge_list) #add edges to the graph object
    x.remove_nodes_from(nx.isolates(x)) #there's a few nodes that dont
actually have edges, and this was easier than going back through the o
riginal excel

    return x

def HS_Mult_sims_bleaching(sims,hosts,edges):
    results_isos=np.zeros((50,sims)) #need a blank matrix of colnum=si
ms and row num=number of temperature steps which is 50
    results_nodes=np.zeros((50,sims))
    results_GC=np.zeros((50,sims))
    for i in xrange(0,sims): #for all the sims do the following
        tols="trial" + str(i+1) + ".csv" #call the right tolerance fil
e
        graph=mygraph_hostspecific(hosts,edges,tols) #make network
        GC, nodes, iso = bleaching(graph) #run bleaching model
        results_isos[:,i]=iso #update the matrix
        results_nodes[:,i]=nodes
        results_GC[:,i]=GC
    return results_isos, results_nodes, results_GC

def myresistanceHS(isos,edges):
    G=mygraph_hostspecific("Global_host_nodes.csv",edges,"trial1.csv")
    N, L, G, C, degrees, kmin, kmax, bin_edges, density,_ =getstats(G)
    numHosts=len(hostseq(G))
    percent_isos=(isos/numHosts)*100
    Temp=mytemp(28,50)

    temp_10=[] #get the 10% temperature
    jtrack=0
    for j in xrange(0,100): #for each simulation
        for i in xrange(0,49): #for each temp increase step
            if j>jtrack: #so that dont get multiple values per simulat
ion
                if percent_isos[i,j]<=10:
                    if percent_isos[i+1,j]>= 10:
                        temp_10.append(Temp[i])
                        jtrack=j

    temp_90=[] #get the 90% temperature
    ktrack=0
    for k in xrange(0,100): #for each simulation
        for i in xrange(0,49): #for each temp increase step
            if k>ktrack: #so that dont get multiple values per simulat
```

```
ion
                if percent_isos[i,k]<=90:
                    if percent_isos[i+1,k]>= 90:
                        temp_90.append(Temp[i])
                        ktrack=k
    #if less than 90% of the corals were bleached at the end of the bl
eaching simulation, then it wont count a temp_90
    #need to add a max temp reading for that missing value. This only
happenned 1x in the indian ocean and 2x in the phuket ocean both in th
e random null model
    if len(temp_90)<99:
        missing=99-len(temp_90)
        for i in xrange(0,missing):
            temp_90.append(Temp[49])

    dtemp=[(x1 - x2)/3 for (x1, x2) in zip(temp_90, temp_10)]


    return temp_10,temp_90,dtemp
```

In [7]:

```
G_hostspec=mygraph_hostspecific("Global_host_nodes.csv","HostSpecific_
Globaledges.csv","trial1.csv")

hsxN, hsxL, hsx, hsC, hsdegrees, hskmin, hskmax, hsbin_edges, hsdensit
y,_ =getstats(G_hostspec)


print hsxN, "nodes"
print hsxL, "links"
print hskmin, "min degree"
print hskmax, "max degree"
```

```
1595 nodes
1697 links
1 min degree
142 max degree
```

In [10]:

```
numsims=100
alltheisosHS,allremovedHS,allGCHS=HS_Mult_sims_bleaching(numsims,"Glob
al_host_nodes.csv","HostSpecific_Globaledges.csv")
```

In [11]:
```
alltheisos_caribbeanHS,allremoved_CHS,allGC_CHS=HS_Mult_sims_bleaching
(numsims,'Global_host_nodes.csv','Caribbean_HSedges.csv')
alltheisos_indianHS,allremoved_IHS,allGC_IHS=HS_Mult_sims_bleaching(nu
msims,'Global_host_nodes.csv','Indian_HSedges.csv')
alltheisos_pacificHS,allremoved_PHS,allGC_PHS=HS_Mult_sims_bleaching(n
umsims,"Global_host_nodes.csv","Pacific_HSedges.csv")

alltheisos_ccHS,allremoved_ccHS,allGC_ccHS=HS_Mult_sims_bleaching(100,
'Global_host_nodes.csv','Central_Caribbean_HSedges.csv')
alltheisos_cpHS,allremoved_cpHS,allGC_cpHS=HS_Mult_sims_bleaching(100,
'Global_host_nodes.csv','Central_Pacific_HSedges.csv')
alltheisos_ecHS,allremoved_ecHS,allGC_ecHS=HS_Mult_sims_bleaching(100,
'Global_host_nodes.csv','Eastern_Caribbean_HSedges.csv')
alltheisos_epHS,allremoved_epHS,allGC_epHS=HS_Mult_sims_bleaching(100,
'Global_host_nodes.csv','Eastern_Pacific_HSedges.csv')
alltheisos_gbrHS,allremoved_gbrHS,allGC_gbrHS=HS_Mult_sims_bleaching(1
00,'Global_host_nodes.csv','Great_Barrier_Reef_HSedges.csv')
alltheisos_jHS,allremoved_jHS,allGC_jHS=HS_Mult_sims_bleaching(100,'Gl
obal_host_nodes.csv','Japan_HSedges.csv')
alltheisos_phHS,allremoved_phHS,allGC_phHS=HS_Mult_sims_bleaching(100,
'Global_host_nodes.csv','Phuket_HSedges.csv')
alltheisos_waHS,allremoved_waHS,allGC_waHS=HS_Mult_sims_bleaching(100,
'Global_host_nodes.csv','Western_Australia_HSedges.csv')
alltheisos_wcHS,allremoved_wcHS,allGC_wcHS=HS_Mult_sims_bleaching(100,
'Global_host_nodes.csv','Western_Caribbean_HSedges.csv')
alltheisos_wiHS,allremoved_wiHS,allGC_wiHS=HS_Mult_sims_bleaching(100,
'Global_host_nodes.csv','Western_Indian_HSedges.csv')
```

In [48]:
```
def mynullsandnetsplot_andHS(name,net_isos,randuniform_isos,symbhostsr
and_isos,randbipar_dc_isos,randbipar_ndc_isos,HSisos, legend,ax):

    Temp=mytemp(28,50)
    net_mean=mymean(net_isos)
    net_high=myconfint(net_isos,97,3)[0]
    net_low=myconfint(net_isos,97,3)[1]
    randuniform_mean=mymean(randuniform_isos)
    randuniform_high=myconfint(randuniform_isos,97,3)[0]
    randuniform_low=myconfint(randuniform_isos,97,3)[1]
    symbhostsrand_mean=mymean(symbhostsrand_isos)
    symbhostsrand_high=myconfint(symbhostsrand_isos,97,3)[0]
    symbhostsrand_low=myconfint(symbhostsrand_isos,97,3)[1]
    randbipar_dc_mean=mymean(randbipar_dc_isos)
    randbipar_dc_high=myconfint(randbipar_dc_isos,97,3)[0]
    randbipar_dc_low=myconfint(randbipar_dc_isos,97,3)[1]
    randbipar_ndc_mean=mymean(randbipar_ndc_isos)
    randbipar_ndc_high=myconfint(randbipar_ndc_isos,97,3)[0]
    randbipar_ndc_low=myconfint(randbipar_ndc_isos,97,3)[1]

    HS_mean=mymean(HSisos)
    HS_high=myconfint(HSisos,97,3)[0]
    HS_low=myconfint(HSisos,97,3)[1]
```

```
    #plot!
    fig = plt.figure(figsize=(8, 6))
    ax.plot(Temp,net_mean,color='black',label='Ocean',linestyle='-',li
newidth=1)
    ax.fill_between(Temp,net_high,net_low,color='grey',alpha=0.5)

    #ax.plot(Temp,randuniform_mean,color='#66c2a4',linestyle='--',line
width=2, label='Random Uniform')
    #ax.fill_between(Temp,randuniform_high,randuniform_low,color='#66c
2a4',alpha=0.25)

    #ax.plot(Temp,symbhostsrand_mean,color='dimgray',linestyle='-',lin
ewidth=2, label='Shuffled Tolerances')
    #ax.fill_between(Temp,symbhostsrand_high,symbhostsrand_low,color='
dimgray',alpha=0.25)

    #ax.plot(Temp,randbipar_dc_mean,color='#238b45',linestyle='-.',lin
ewidth=3, label='Random Bipartite DC')
    #plt.fill_between(Temp,randbipar_dc_high,randbipar_dc_low,color='#
238b45',alpha=0.25)

    #ax.plot(Temp,randbipar_ndc_mean,color='#00441b',linestyle=':',lin
ewidth=2, label='Random Bipartite NDC')
    #ax.fill_between(Temp,randbipar_ndc_high,randbipar_ndc_low,color='
#00441b',alpha=0.25)

    ax.plot(Temp,HS_mean,color='red',linestyle=':',linewidth=2, label=
'Host Specific')
    ax.fill_between(Temp,HS_high,HS_low,color='red',alpha=0.25)



    ax.set_ylim(0,100)
    #ax.set_xlabel('Temperature degrees celsius',fontsize=15)
    #ax.set_ylabel('Percent of Hosts Bleached',fontsize=15)

    ax.set_title(name)
    #ax.patch.set_facecolor('#cccccc')


    plt.close()
    return fig
```

In [49]:
```
legend="upper left"

fig,((ax1,ax2),(ax3,ax4))=plt.subplots(nrows=2,ncols=2,sharex='col', s
harey='row',figsize=(14,10))
mynullsandnetsplot_andHS("Pacific",alltheisos_pacific,Pisos_rand,Pisos
_hostssymbsrand,Presults_isos_dc,Presults_isos_ndc,alltheisos_pacificH
S, 'upper left',ax2)
mynullsandnetsplot_andHS("Global",alltheisos,Gisos_rand,Gisos_hostssym
bsrand,Gresults_isos_dc,Gresults_isos_ndc,alltheisosHS, 'upper left',a
x1)
mynullsandnetsplot_andHS("Indian",alltheisos_indian,Iisos_rand,Iisos_h
ostssymbsrand,Iresults_isos_dc,Iresults_isos_ndc,alltheisos_indianHS,
'upper left',ax3)
mynullsandnetsplot_andHS("Caribbean",alltheisos_caribbean,Cisos_rand,C
isos_hostssymbsrand,Cresults_isos_dc,Cresults_isos_ndc, alltheisos_car
ibbeanHS,'upper left',ax4)
fig.text(0.5, 0.01, 'Temperature, degrees celsius', ha='center',fontsi
ze=15)
fig.text(0.01, 0.5, 'Percent of hosts bleached', va='center', rotation
='vertical',fontsize=15)
legend=plt.legend( loc = 'upper center', bbox_to_anchor = (0.5, -0.05)
,ncol=4,
            bbox_transform = plt.gcf().transFigure ,fontsize=15)
#legend.get_frame().set_facecolor('#cccccc')
```

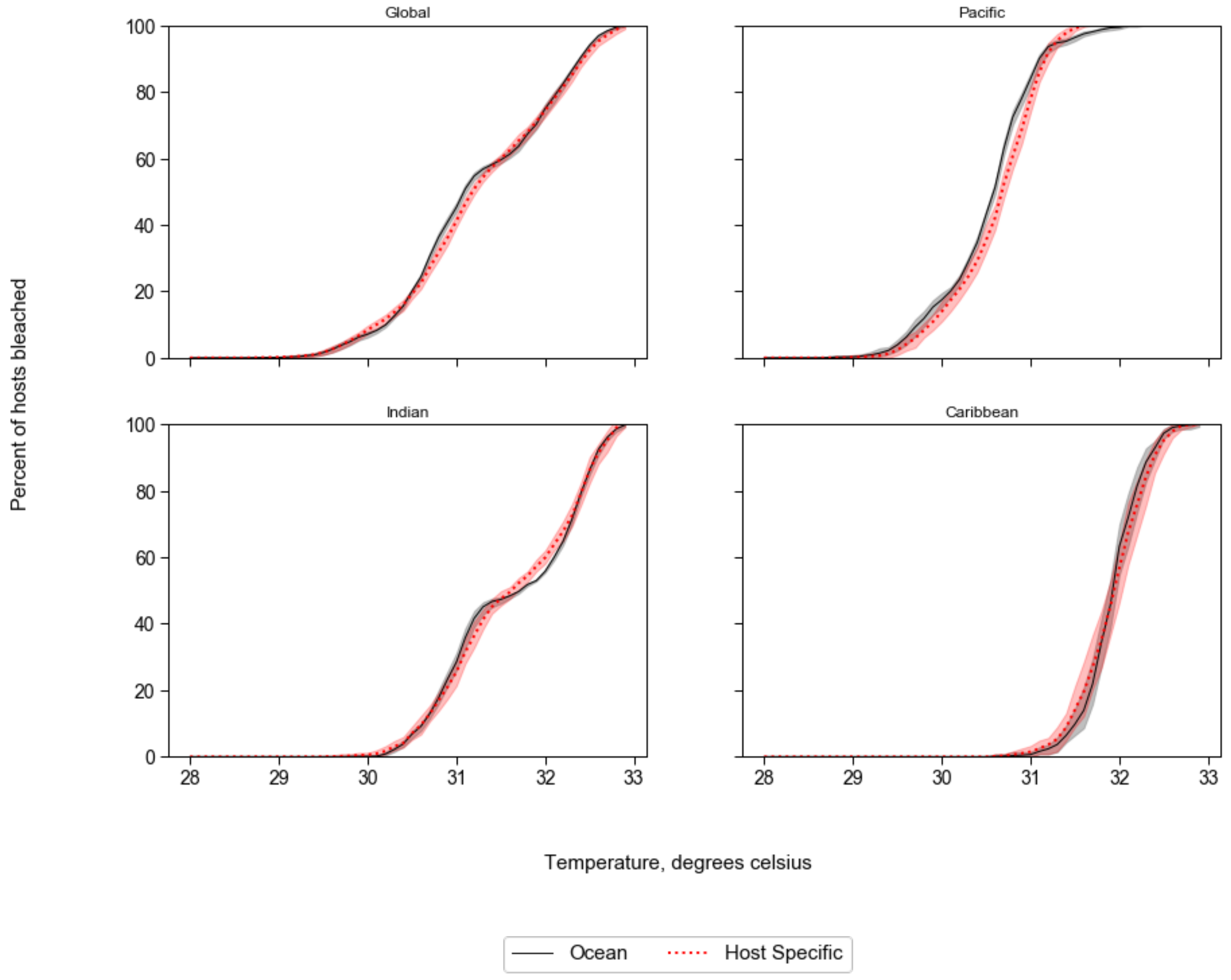

Now let's get the resistance values to export:

In [50]:
```
temp_10_Ghs,temp_90_Ghs,dtemp_Ghs=myresistanceHS(alltheisosHS,"HostSpecific_Globaledges.csv")
temp_10_Ihs,temp_90_Ihs,dtemp_Ihs=myresistanceHS(alltheisos_indianHS,'Indian_HSedges.csv')
temp_10_Chs,temp_90_Chs,dtemp_Chs=myresistanceHS(alltheisos_caribbeanHS,"Caribbean_HSedges.csv")
temp_10_Phs,temp_90_Phs,dtemp_Phs=myresistanceHS(alltheisos_pacificHS,"Pacific_HSedges.csv")

temp_10_cchs,temp_90_cchs,dtemp_cchs=myresistanceHS(alltheisos_ccHS,"Central_Caribbean_HSedges.csv")
temp_10_cphs,temp_90_cphs,dtemp_cphs=myresistanceHS(alltheisos_cpHS,"Central_Pacific_HSedges.csv")
temp_10_wchs,temp_90_wchs,dtemp_wchs=myresistanceHS(alltheisos_wcHS,"Western_Caribbean_HSedges.csv")
temp_10_echs,temp_90_echs,dtemp_echs=myresistanceHS(alltheisos_ecHS,"Eastern_Caribbean_HSedges.csv")
temp_10_gbrhs,temp_90_gbrhs,dtemp_gbrhs=myresistanceHS(alltheisos_gbrHS,"Great_Barrier_Reef_HSedges.csv")
temp_10_phhs,temp_90_phhs,dtemp_phhs=myresistanceHS(alltheisos_phHS,"Phuket_HSedges.csv")
temp_10_jhs,temp_90_jhs,dtemp_jhs=myresistanceHS(alltheisos_jHS,"Japan_HSedges.csv")
temp_10_wahs,temp_90_wahs,dtemp_wahs=myresistanceHS(alltheisos_waHS,"Western_Australia_HSedges.csv")
temp_10_wihs,temp_90_wihs,dtemp_wihs=myresistanceHS(alltheisos_wiHS,"Western_indian_HSedges.csv")
```

In [53]:
```
a=np.column_stack((dtemp_Ghs,dtemp_Ihs,dtemp_Chs,dtemp_Phs,dtemp_cchs,dtemp_cphs,dtemp_wchs,dtemp_echs,dtemp_gbrhs,dtemp_phhs,dtemp_jhs,dtemp_wahs,dtemp_wihs))
df=pd.DataFrame(a)
df.columns=["dtemp_Ghs","dtemp_Ihs","dtemp_Chs","dtemp_Phs","dtemp_cchs","dtemp_cphs","dtemp_wchs","dtemp_echs","dtemp_gbrhs","dtemp_phhs","dtemp_jhs","dtemp_wahs","dtemp_wihs"]
```

A generalized global network

I took out all spatial aspects of the network. Hosts and symbionts have the same inclusion parameters, and the network represents all possible combinations of symbionts and species on a global scale regardless of spatial restrictions. However, this means that environmental temperature cannot be included. Thus, the weight is now just the average of the host and symbiont tolerance.

In [8]:

```
def mygraph_general(hosts,edges,tols): #this function creates a graph object from nodes,edges, and missing tolerances files

    x=nx.Graph() #create empty graph
    #get all the data imported
    hostnodes = pd.read_csv(hosts)
    symbnodesgood=pd.read_csv('Global_symbiontgood_nodes.csv') #the symbionts that had tolerances listed in Swain et al. 2016a
    symbnodesrest=pd.read_csv('Global_symbiontbad_nodes.csv') #the ones that didn't

    fittols=pd.read_csv(tols)
    #combine the tolerance file with the symbionts that need tolerances
    symbnodesrest['tols']=fittols['tolerance']
    #Add nodes into the graph with their attributes
    for row in hostnodes.iterrows():
        x.add_node(row[1][0], name=row[1][1],type=row[1][2], tolerance=row[1][3])
    for row in symbnodesgood.iterrows():
        x.add_node(row[1][0], name=row[1][1],type=row[1][2],genetic=row[1][3], tolerance=row[1][4])
    for row in symbnodesrest.iterrows():
        x.add_node(row[1][0], name=row[1][1],type=row[1][2],genetic=row[1][3], tolerance=row[1][4])

    #now for the edges
    edges = pd.read_csv(edges,header=None) #ordered by ocean and then by region in alphabetical order
    edge_list=[] #an empty list of edges
    thresh_list=[] #an empty list of thresholds
    for row in edges.iterrows():
        s=row[1][0] #symbiont ID is in first column
        h=row[1][1] #Host ID is n second column
        #get the tolerance values from the node attributes
        symb_tol=x.node[s]['tolerance']
        host_tol=x.node[h]['tolerance']
        #calculate the threshold for each edge based on node pairs
        threshvalue=((0.5*(symb_tol+host_tol)))
        thresh_list.append(threshvalue)
        #update the edge list with a weight determined by thresholds
        edge_list.append((s,h,{'weight':threshvalue}))

    x.add_edges_from(edge_list) #add edges to the graph object
    x.remove_nodes_from(nx.isolates(x)) #there's a few nodes that dont actually have edges, and this was easier than going back through the original excel

    return x
```

In [9]:
```
Ggen=mygraph_general("generalized_global_hostnodes.csv","generalized_g
lobal_edges.csv","trial1.csv")
```

In [10]:
```
xN, xL, x, C, degrees, kmin, kmax, bin_edges, density,_ =getstats(Ggen
)

print xN, "nodes"
print xL, "links"
print kmin, "min degree"
print kmax, "max degree"
```

```
606 nodes
1322 links
1 min degree
208 max degree
```

In [12]:
```
import powerlaw
globnet=mygraph("Global_host_nodes.csv","Global_edges.csv","trial1.csv
")
#get network info
G=Ggen #Switch for different networks
xN, xL, x, C, degrees, kmin, kmax, bin_edges, density,_ =getstats(G)


degs=np.array(degrees)
#get fit
fit = powerlaw.Fit(degs, discrete=True)
print fit.distribution_compare('power_law', 'truncated_power_law'), "p
ower law vs truncated"
print fit.distribution_compare('exponential', 'truncated_power_law'),
"exponential vs truncated"
print fit.distribution_compare('stretched_exponential', 'truncated_pow
er_law'), "stretched exponential vs truncated"
print fit.distribution_compare('lognormal', 'truncated_power_law'), "l
ognormal vs truncated"
```

```
Calculating best minimal value for power law fit
Assuming nested distributions

(-0.031637798715239196, 0.8013916837556763) power law vs truncated
(-76.55744711194367, 0.0012158303665313528) exponential vs truncated
(-1.0489230048061882, 0.32871291634116073) stretched exponential vs
truncated
(-0.057775139471285986, 0.34747679095269157) lognormal vs truncated
```

In [13]:
```
def get_degree_distribution_binning(data):
    # Implement a function that from a vector of data (e.g. the degree
s of your nodes)
    # create nb bins either using linear binning (log=False, the defau
lt value) or by using log binning in base 10
    # (log=True).
    # Your function must return you vectors:
    #   xs: the the midpoint of each bin
    #   ys: the height of each bin

    # Your code
    degrees=data
    kmax=max(degrees)
    kmin=min(degrees)
    if kmin<1:
        kmin=1
    N=len(degrees)
    Num=50
    #bin_edges = np.logspace(np.log10(kmin), np.log10(kmax), num=Num)
#xs
    bin_edges=np.linspace(kmin,kmax,num=Num) #so now using linear
    density, _ = np.histogram(degrees, bins=bin_edges, density=True) #
ys
    #now need to get stuff for log log
    logbin_edges=np.logspace(np.log10(kmin),np.log10(kmax),num=Num) #s
o now using linear
    logdensity, _ = np.histogram(degrees, bins=logbin_edges, density=T
rue) #ys
    binwidth=np.diff(logbin_edges)
    prob=(binwidth*logdensity)
    cummulative=np.cumsum(prob[::-1])[::-1]
    return bin_edges, density,_, cummulative, logbin_edges
```

In [16]:
```
xN, xL, x, C, degrees, kmin, kmax, bin_edges, density,_ =getstats(glob
net)

bin_edges,density,_,cummulative,logbin_edges=get_degree_distribution_b
inning(degrees)
```

In [18]:
```
def hostseq(test):
    degrees = test.degree().values()
    d=nx.degree(test)
    nx.set_node_attributes(test,'degree',d)
    hostdegs=np.zeros((1,731))

    for node in test.nodes():
        if test.node[node]['type']==0 :
                hostdegs[0,node]=test.node[node]['degree']
    hostdegs=hostdegs[hostdegs!=0]
    host_seq=tuple(hostdegs)
    new=[]
    for c in xrange(0,len(host_seq)):
        ugh=host_seq[c]
        why=int(ugh)
        new.append(why)
    host_seq=tuple(new)
    return (host_seq)

def symbseq(test):
    degrees = test.degree().values()
    d=nx.degree(test)
    nx.set_node_attributes(test,'degree',d)
    symbdegs=np.zeros((1,982))
    for node in test.nodes():
        if test.node[node]['type']==1 :
                symbdegs[0,node]=test.node[node]['degree']
    symbdegs=symbdegs[symbdegs!=0]
    symb_seq=tuple(symbdegs)
    new=[]

    for c in xrange(0,len(symb_seq)):
        ugh=symb_seq[c]
        why=int(ugh)
        new.append(why)
    symb_seq=tuple(new)
    return (symb_seq)

hostdegs=hostseq(globnet)
symbdegs=symbseq(globnet)
```

In [19]:
```
Hbin_edges,Hdensity,_,Hcummulative,Hlogbin_edges=get_degree_distributi
on_binning(hostdegs)

Sbin_edges,Sdensity,_,Scummulative,Slogbin_edges=get_degree_distributi
on_binning(symbdegs)

G_hostspec=mygraph_hostspecific("Global_host_nodes.csv","HostSpecific_
Globaledges.csv","trial1.csv")
hsxN, hsxL, hsx, hsC, hsdegrees, hskmin, hskmax, hsbin_edges, hsdensit
y,_ =getstats(G_hostspec)
HSbin_edges,HSdensity,_,HScummulative,HSlogbin_edges=get_degree_distri
bution_binning(hsdegrees)

genxN, genxL, genx, genC, gendegrees, genkmin, genkmax, genbin_edges,
gendensity,_ =getstats(Ggen)
genbin_edges,gendensity,_,gencummulative,genlogbin_edges=get_degree_di
stribution_binning(gendegrees)

plt.loglog(Slogbin_edges[:-1], Scummulative, marker='o', linestyle='no
ne', color='yellow', label="Symbionts")
plt.loglog(Hlogbin_edges[:-1], Hcummulative, marker='o', linestyle='no
ne', color='blue', label="Hosts")
plt.loglog(logbin_edges[:-1], cummulative, marker='o', linestyle='none
', color='green', label="Combined")
plt.loglog(HSlogbin_edges[:-1], HScummulative, marker='o', linestyle='
none', color='black', label="Host Specific Network")
plt.loglog(genlogbin_edges[:-1], gencummulative, marker='o', linestyle
='none', color='grey', label="Generalized")



plt.xlabel("Degree, k")
plt.ylabel( u'P(K) ≤ k')
legend=plt.legend( loc = 'upper center', bbox_to_anchor = (0.5, -0.05)
,ncol=2,
            bbox_transform = plt.gcf().transFigure ,fontsize=15)
```

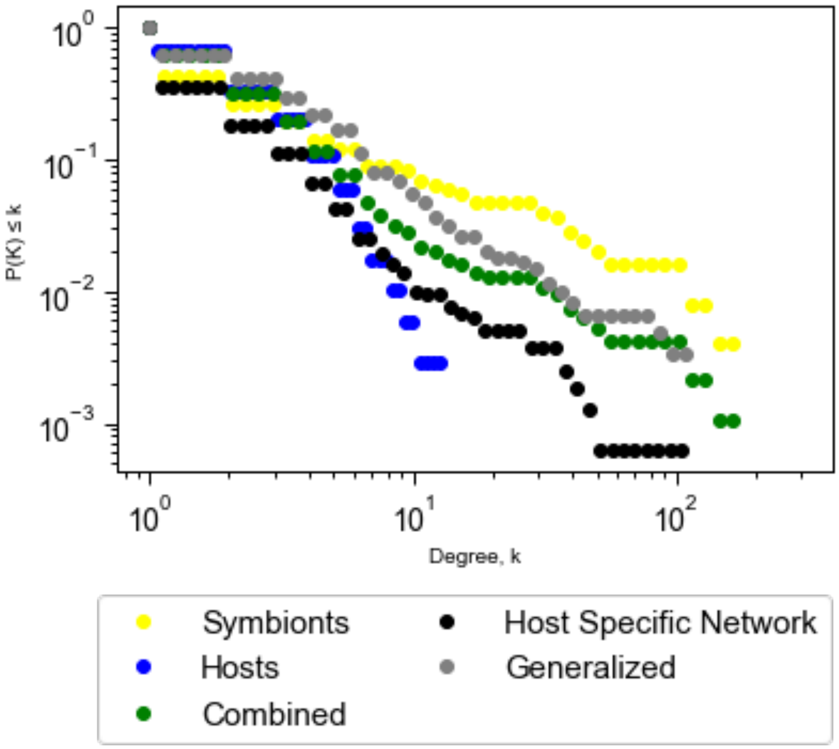

10^0
10^{-1}
10^{-2}
10^{-3}
P(K) ≤ k
10^0
10^1
10^2
Degree, k
Symbionts
Hosts
Combined
Host Specific Network
Generalized

In [ ]:
