# Supplementary material for: Resistance and robustness of the global coral–symbiont network
Source: Ecology. 2020 Feb 14;101(5):e02990. doi: 10.1002/ecy.2990 (PMC7317464; doi:10.1002/ecy.2990)
Supplement: Supplementary file 4 [file ECY-101-e02990-s004.zip › DataS2/RemovalModels_Robustness_py27.pdf]

Robustness of the global network of coral species and their associated algal symbionts

Robustness of food webs to species loss has been quantified as the proportion of species removed that resulted in a total loss of some specified proportion (50% is a common threshold) of the species (Dunne & Williams, 2009). Fabina et al. (2013) applied this measure to the bipartite network of coral species and their symbionts in Moorea by simulating local extinctions, but this analysis has not been applied on a global scale, and has only been applied to specific removal cases. Node removals from ecological networks represent a species “extinction.” However, interactions are likely to change or disappear on more ecologically relevant timescales. Thus, link removals need to be considered in ecological robustness analyses. The R50 value is the amount of nodes or links needed to be removed to decrease the number of (total, host, or symbiont) nodes remaining to 50%

In [1]:
```
#Check version of python used
import sys
print sys.version
```

```
2.7.16 |Anaconda, Inc.| (default, Mar 14 2019, 16:24:02)
[GCC 4.2.1 Compatible Clang 4.0.1 (tags/RELEASE_401/final)]
```

In [2]:
```
#Set the file directory
import os #to use os.chdir
#path = "" #change to your specific path
os.chdir( path ) #set directory
%pwd #output current directory
```

Out[2]:
```
u'/Users/saradellwilliams/Dropbox/Williams_Suppmat_obj2/Updated8_201
7/Code/Python/Inputs'
```

```
In [3]: #import all of the packages that you will need to run the rest of the
code
import numpy as np
import networkx as nx
import matplotlib.pyplot as plt
import matplotlib
import scipy.cluster.hierarchy as hierarchy
import math
from pylab import figure
import pandas as pd
from scipy.optimize import curve_fit
import scipy as scipy
from scipy import stats
from operator import itemgetter, attrgetter
import random
%matplotlib inline
# change fonts and formatting options
matplotlib.rc('xtick', labelsize=14)
matplotlib.rc('ytick', labelsize=14)
matplotlib.rc('font', **{'family':'sans-serif','sans-serif':['Arial']}
)
matplotlib.rc('font', size=16)
matplotlib.rc('xtick.major', size=6, width=1)
matplotlib.rc('xtick.minor', size=3, width=1)
matplotlib.rc('ytick.major', size=6, width=1)
matplotlib.rc('ytick.minor', size=3, width=1)
matplotlib.rc('axes', linewidth=1)
```

First, define the common functions I use.

```
In [4]: def mygraph(hosts,edges,tols): #this function creates a graph object f
rom nodes,edges, and missing tolerances files

    x=nx.Graph() #create empty graph
    #get all the data imported
    hostnodes = pd.read_csv(hosts)
    symbnodesgood=pd.read_csv('Global_symbiontgood_nodes.csv') #the sy
mbionts that had tolerances listed in Swain et al. 2016a
    symbnodesrest=pd.read_csv('Global_symbiontbad_nodes.csv') #the one
s that didn't

    fittols=pd.read_csv(tols)
    #combine the tolerance file with the symbionts that need tolerance
s
    symbnodesrest['tols']=fittols['tolerance']
    #Add nodes into the graph with their attributes
    for row in hostnodes.iterrows():
        x.add_node(row[1][0], ocean=row[1][1], name=row[1][2],type=row
[1][3],genetic=row[1][4], tolerance=row[1][5])
    for row in symbnodesgood.iterrows():
```

```
        x.add_node(row[1][0], name=row[1][1],type=row[1][2],genetic=ro
w[1][3], tolerance=row[1][4])
    for row in symbnodesrest.iterrows():
        x.add_node(row[1][0], name=row[1][1],type=row[1][2],genetic=ro
w[1][3], tolerance=row[1][4])

    #now for the edges
    edges = pd.read_csv(edges,header=None) #ordered by ocean and then
by region in alphabetical order
    edge_list=[] #an empty list of edges
    thresh_list=[] #an empty list of thresholds
    for row in edges.iterrows():
        s=row[1][0] #symbiont ID is in first column
        h=row[1][1] #Host ID is n second column
        MMM=row[1][2] #third column is the mean monthly max temperatur
e
        #get the tolerance values from the node attributes
        symb_tol=x.node[s]['tolerance']
        host_tol=x.node[h]['tolerance']
        #calculate the threshold for each edge based on node pairs
        threshvalue=(MMM+(1.5*(symb_tol+host_tol)))
        thresh_list.append(threshvalue)
        #update the edge list with a weight determined by thresholds
        edge_list.append((s,h,{'weight':threshvalue}))

    x.add_edges_from(edge_list) #add edges to the graph object
    x.remove_nodes_from(list(nx.isolates(x))) #there's a few nodes tha
t dont actually have edges, and this was easier than going back throug
h the original excel

    return x,edge_list

#The main Bleaching model function:
def bleaching(G):
    #create a bunch of empty lists
    N_g1= []
    nodesremoved=[]
    iso=[]

    for i in xrange(0,50): #over the range of the temperature steps, d
o the following:
        G1=G.copy() #as a precaution, copy the graph so that you dont
actually change the original one
        T=28+0.1*i #for each step, the change of T is 0.1
        for j in G1.edges(): #look over the edges
            if G1.edge[j[0]][j[1]]['weight']<=T: #when T exceeds the t
hreshold, remove link
                G1.remove_edge(j[0],j[1])
        C=nx.connected_components(G1) #recalculate the connected compo
nents
        isolated_nodes=0 #Calculate the isolated nodes
        for m in C: #look over the components
            if len(m)==1: #if the component is just 1 node
```

```
                if m[0]<=730: #dont count the symbiont, just add the host nodes whose ID#s go up to 730
                    isolated_nodes=isolated_nodes+1 #update the isolated nodes size
        iso.append(isolated_nodes) #update isolated host nodes list
        N_g1.append(max(map(len, nx.connected_components(G1)))) #recalculate the giant component size
        isolates=nx.isolates(G1) #all the isolated nodes
        G1.remove_nodes_from(isolates) #remove the isolated nodes from the network
        nodesremoved.append(len(isolates)) #total number of nodes removed, including symbionts
    return N_g1,nodesremoved, iso

#Run multiple simulations of Bleaching model
def Mult_sims_bleaching(sims,hosts,edges):
    results_isos=np.zeros((50,sims)) #need a blank matrix of colnum=sims and row num=number of temperature steps which is 50
    results_nodes=np.zeros((50,sims))
    results_GC=np.zeros((50,sims))
    for i in xrange(0,sims): #for all the sims do the following
        tols="trial" + str(i+1) + ".csv" #call the right tolerance file
        graph=mygraph(hosts,edges,tols) #make network
        GC, nodes, iso = bleaching(graph) #run bleaching model
        results_isos[:,i]=iso #update the matrix
        results_nodes[:,i]=nodes
        results_GC[:,i]=GC
    return results_isos, results_nodes, results_GC

#get the degree sequence for the hosts
def hostseq(test):
    degrees = test.degree().values()
    d=nx.degree(test)
    nx.set_node_attributes(test,'degree',d)
    hostdegs=np.zeros((1,731))

    for node in test.nodes():
        if test.node[node]['type']==0 :
                hostdegs[0,node]=test.node[node]['degree']
    hostdegs=hostdegs[hostdegs!=0]
    host_seq=tuple(hostdegs)
    new=[]
    for c in xrange(0,len(host_seq)):
        ugh=host_seq[c]
        why=int(ugh)
        new.append(why)
    host_seq=tuple(new)
    return (host_seq)

#get the degree sequence for the symbionts
def symbseq(test):
    degrees = test.degree().values()
```

```
    d=nx.degree(test)
    nx.set_node_attributes(test,'degree',d)
    symbdegs=np.zeros((1,982))
    for node in test.nodes():
        if test.node[node]['type']==1 :
                symbdegs[0,node]=test.node[node]['degree']
    symbdegs=symbdegs[symbdegs!=0]
    symb_seq=tuple(symbdegs)
    new=[]

    for c in xrange(0,len(symb_seq)):
        ugh=symb_seq[c]
        why=int(ugh)
        new.append(why)
    symb_seq=tuple(new)
    return (symb_seq)

#Make the Random Bipartite Not Degree Conserved null network model (NO
T USED)
def get_biparnull_ndc(edges):
    nodes="Global_host_nodes.csv"
    hosttols="hosttolerances_init.csv"
    symbtols="symbionttolerances_init.csv"
    graph,edgelist=mygraph(nodes,edges,symbtols) #makes the graph so t
hat you can get the hostseq and symbseq
    host_seq=hostseq(graph) #gets degree sequence of hosts
    symb_seq=symbseq(graph) #gets degree sequence of symbionts
    G=nx.Graph()
    #choose which model to run

    x=nx.bipartite_gnmk_random_graph(len(host_seq), len(symb_seq), gra
ph.number_of_edges(), seed=None, directed=False)
    hostnodes=x.nodes()[0:len(host_seq)]
    symbnodes=x.nodes()[len(host_seq):len(x.nodes())]

    symbtols=pd.read_csv(symbtols)
    hosttols=pd.read_csv(hosttols)
    symbtols=symbtols[0:len(symbnodes)]
    hosttols=hosttols[0:len(hostnodes)]
    hosttols['ID']=hostnodes
    symbtols['ID']=symbnodes

    for row in hosttols.iterrows():
        G.add_node(row[1][1], tolerance=row[1][0])
    for row in symbtols.iterrows():
        G.add_node(row[1][1], tolerance=row[1][0])

    edgesMMM = pd.read_csv(edges,header=None)
    edges = x.edges()
    edge_list=[]
    thresh_list=[]
    for i in xrange(0,len(edges)):
        MMM=edgesMMM[2][i]
```

```
        s=edges[i][1]
        h=edges[i][0]
        symb_tol=G.node[s]['tolerance']
        host_tol=G.node[h]['tolerance']
        #calculate the threshold for each edge based on node pairs
        threshvalue=(MMM)+(1.5*(symb_tol+host_tol))
        thresh_list.append(threshvalue)
        edge_list.append((s,h,{'weight':threshvalue}))

    G.add_edges_from(edge_list) #add edges to the graph object

    return G
```

Lists of names for use in running and saving results

In [5]:
```
network_edges=["Global_edges.csv",
'Caribbean_edges.csv',
'Indian_edges.csv',
"Pacific_edges.csv",
'Central_Caribbean_edges.csv',
'Central_Pacific_edges.csv',
'Eastern_Caribbean_edges.csv',
'Eastern_Pacific_edges.csv',
'GBR_edges.csv',
'Japan_edges.csv',
'Phuket_edges.csv',
'Western_Australia_edges.csv',
'Western_Caribbean_edges.csv',
'Western_Indian_edges.csv']
abbrevs=['G','C','I','P','cc','cp','ec','ep','gbr','j','ph','wa','wc',
'wi']
```

Let's start with link removals.

- Random link removal
- Bleaching link removal
- Link tolerance removal

Random link removal: Shuffles the order of the edgelist and removes edges in order of their place in the edgelist

In [7]:
```
def removeRandomLink(G,edges,nettype):

    G.remove_nodes_from(nx.isolates(G)) #get rid of any isolated nodes
```

```
, that could exist in a RBNDC generated graph
    edge_list=G.edges(data=True) #get edgelist
    random.shuffle(edge_list) #shuffle the edgelist

    #get blank lists set up fro results
    N_g = []
    iso_h=[]
    iso_s=[]
    total_isos=[]

    #get number of symbionts and hosts in the network for use if doing
a null model
    F,hedge_list=mygraph("Global_host_nodes.csv",edges,"symbionttolera
nces_init.csv")
    num_hosts=len(hostseq(F))
    num_symbs=len(symbseq(F))

    if nettype=="null":
        h=num_hosts
    else:
        h=730

    #get the initial sizes of things
    N_g.append(max(map(len, nx.connected_components(G))))
    iso_h.append(0)
    iso_s.append(0)
    total_isos.append(0)

    #start removing links
    while len(edge_list) > 1:
        a=edge_list[0][0]
        b=edge_list[0][1]
        edge_list.remove(edge_list[0]) #remove the link at the top of
the list from the edgelist
        G.remove_edge(a,b) #remove the link from the graph

        N_g.append(max(map(len, nx.connected_components(G)))) #update
the size of the giant component
        C=nx.connected_components(G) #recalculate the connected compon
ents
        #start the isolated counts at 0
        isolated_hosts=0
        isolated_symbs=0
        for m in C: #look over the components
            if len(m)==1: #if the component is just 1 node
                if m[0]<=h:
                    isolated_hosts=isolated_hosts+1 #update the isolat
ed host nodes size
                else:
                    isolated_symbs=isolated_symbs+1
        iso_h.append(isolated_hosts) #update isolated host nodes list
        iso_s.append(isolated_symbs)
        total_isos.append(isolated_hosts+isolated_symbs)
```

```
    if len(N_g)<len(F.edges()):
        x=len(F.edges())-len(N_g)
        for i in xrange(0,x):
            N_g.append(0)
    if len(iso_h)<len(F.edges()):
        y=len(F.edges())-len(iso_h)
        for i in xrange(0,y):
            iso_h.append(num_hosts)
    if len(iso_s)<len(F.edges()):
        z=len(F.edges())-len(iso_s)
        for i in xrange(0,z):
            iso_s.append(num_symbs)
    if len(total_isos)<len(F.edges()):
        a=len(F.edges())-len(total_isos)
        for i in xrange(0,a):
            total_isos.append(len(F.nodes()))

    return N_g, iso_h, iso_s, total_isos

# Now for the multiple simulations functions

def MultSimsRemovals_RL(edges,nettype):
    sims=100
    hosts="Global_host_nodes.csv"
    #setup results arrays
    G1,edge_list=mygraph(hosts,edges,"symbionttolerances_init.csv")
    length=len(G1.edges())
    results_isos_s=np.zeros((length,sims))
    results_isos_h=np.zeros((length,sims))
    results_isos_total=np.zeros((length,sims))
    results_GC=np.zeros((length,sims))

    if nettype=="net":
        for i in xrange(0,sims): #for all the sims do the following
            tols="trial" + str(i+1) + ".csv" #call the right tolerance
file
            G,edge_list=mygraph(hosts,edges,tols) #make network
            N_g, iso_h, iso_s, total_isos=removeRandomLink(G,edges,net
type)
            results_GC[:,i]=N_g
            results_isos_h[:,i]=iso_h
            results_isos_s[:,i]=iso_s
            results_isos_total[:,i]=total_isos

    if nettype=="null":
        for i in xrange(0,sims): #for all the sims do the following
            G=get_biparnull_ndc(edges) #make network
            N_g, iso_h, iso_s, total_isos=removeRandomLink(G,edges,net
type)
            results_GC[:,i]=N_g
            results_isos_h[:,i]=iso_h
            results_isos_s[:,i]=iso_s
```

```
            results_isos_total[:,i]=total_isos

    return results_GC, results_isos_h, results_isos_s, results_isos_to
tal

#R50 function

def R50_RL(edges,nettype):
    results_GC, iso_h, iso_s, iso_total=MultSimsRemovals_RL(edges,nett
ype)
    G,edge_list=mygraph("Global_host_nodes.csv",edges,"symbionttoleran
ces_init.csv")
    numHosts=len(hostseq(G))
    numSymbs=len(symbseq(G))
    numNodes=len(G.nodes())
    totedges=len(G.edges())
    #print totedges, "totedges"

    GC=results_GC
    GCstart=(GC[0,:])
    GCfrac=GC/GCstart
    meanGCfrac=np.mean(GCfrac,axis=1)

    percHostsRemoved=(iso_h/numHosts)*100
    percHostsRemaining=100-percHostsRemoved
    meanPHR=np.mean(percHostsRemaining,axis=1)
    #print np.shape(percHostsRemaining)

    percSymbsRemoved=(iso_s/numSymbs)*100
    percSymbsRemaining=100-percSymbsRemoved
    meanPSR=np.mean(percSymbsRemaining,axis=1)
    #print np.shape(percSymbsRemaining)

    percNodesRemoved=(iso_total/numNodes)*100
    percNodesRemaining=100-percNodesRemoved
    meanPNR=np.mean(percNodesRemaining,axis=1)
    #print np.shape(percNodesRemaining)
    #print percNodesRemaining

    LR=list(xrange(0,len(iso_total)))
    LR=np.array(LR,dtype='float')
    percLR=(LR/totedges)*100
    numremovals=len(iso_total)
    #print numremovals, "numremovals"

    LR_H50=[] #get hosts R50 values
    jtrack=0 #set initial J
    for j in xrange(0,100): #for each simulation do the following
        for i in xrange(1,numremovals): #for each of the removals do t
he following
            if j>jtrack: #so that dont get multiple values per simulat
ion
                if percHostsRemaining[i,j]<=50: #if the pHR of the rem
```

```
oval step is <=50
                    if percHostsRemaining[i-1,j]>=50: #and if the pHR
predecessor (previous removal step) was >=50
                        LR_H50.append(i) #the R50 values for this simu
lations is this removal step which
                                    #corresponds to how many links hav
e been removed so far
                        jtrack=j #update j

    LR_S50=[] #get the symbionts R50 values
    jtrack=0
    for j in xrange(0,100):
        for i in xrange(1,numremovals):
            if j>jtrack:
                if percSymbsRemaining[i,j]<=50:
                    if percSymbsRemaining[i-1,j]>=50:
                        LR_S50.append(i)
                        jtrack=j
    LR_N50=[]
    jtrack=0
    for j in xrange(0,100):
        for i in xrange(1,numremovals):
            if j>jtrack:
                if percNodesRemaining[i,j]<=50:
                    if percNodesRemaining[i-1,j]>=50:
                        LR_N50.append(i)
                        jtrack=j

    #print len(LR_N50), "NR50 length"
    #print len(LR_H50), "HR50 length"
    #print len(LR_S50), "SR50 length"
    percLR_SR50=list(np.divide(LR_S50,totedges,dtype=float))
    percLR_HR50=list(np.divide(LR_H50,totedges,dtype=float))
    percLR_NR50=list(np.divide(LR_N50,totedges,dtype=float))
    meanR50symbs=np.mean(percLR_SR50)
    meanR50hosts=np.mean(percLR_HR50)
    meanR50total=np.mean(percLR_NR50)
    stdR50symbs=np.std(percLR_SR50)
    stdR50hosts=np.std(percLR_HR50)
    stdR50total=np.std(percLR_NR50)
    return(meanGCfrac,meanPHR,meanPSR,meanPNR,percLR,percLR_HR50,percL
R_SR50,percLR_NR50,meanR50hosts,stdR50hosts,meanR50symbs,stdR50symbs,
meanR50total,stdR50total)
```

Run the Random Link Removals

In [8]:
```
#NOW the natural Networks
for i in xrange(0,14):
    meanGCfrac,meanPHR,meanPSR,meanPNR,percLR,percLR_HR50,percLR_SR50,
percLR_NR50,meanR50hosts,stdR50hosts,meanR50symbs,stdR50symbs, meanR50
total,stdR50total=R50_RL(network_edges[i],"net")
    globals()['RL_mGCfrac_%s' % abbrevs[i]]=meanGCfrac
    globals()['RL_mpHR_%s' % abbrevs[i]]=meanPHR
    globals()['RL_mpSR_%s' % abbrevs[i]]=meanPSR
    globals()['RL_mpNR_%s' % abbrevs[i]]=meanPNR
    globals()['RL_pLR_%s' % abbrevs[i]]=percLR
    globals()['RL_R50H_%s' % abbrevs[i]]=percLR_HR50 #misleading becau
se it is the fraction not percent value
    globals()['RL_R50S_%s' % abbrevs[i]]=percLR_SR50
    globals()['RL_R50N_%s' % abbrevs[i]]=percLR_NR50
    globals()['RL_mR50H_%s' % abbrevs[i]]=meanR50hosts
    globals()['RL_mR50S_%s' % abbrevs[i]]=meanR50symbs
    globals()['RL_mR50N_%s' % abbrevs[i]]=meanR50total
    globals()['RL_std50H_%s' % abbrevs[i]]=stdR50hosts
    globals()['RL_std50S_%s' % abbrevs[i]]=stdR50symbs
    globals()['RL_std50N_%s' % abbrevs[i]]=stdR50total
```

In [9]:
```
### saves the mean R50s and their stdevs

meanR50H_all=[]
stdR50H_all=[]
meanR50S_all=[]
stdR50S_all=[]
meanR50N_all=[]
stdR50N_all=[]

for i in xrange(0,14):
    meanR50H_all.append(globals()['RL_mR50H_%s' % abbrevs[i]])
    meanR50S_all.append(globals()['RL_mR50S_%s' % abbrevs[i]])
    stdR50H_all.append(globals()['RL_std50H_%s' % abbrevs[i]])
    stdR50S_all.append(globals()['RL_std50S_%s' % abbrevs[i]])
    meanR50N_all.append(globals()['RL_mR50N_%s' % abbrevs[i]])
    stdR50N_all.append(globals()['RL_std50N_%s' % abbrevs[i]])


meanR50H_all=np.array(meanR50H_all)
stdR50H_all=np.array(stdR50H_all)
meanR50S_all=np.array(meanR50S_all)
stdR50S_all=np.array(stdR50S_all)
meanR50N_all=np.array(meanR50N_all)
stdR50N_all=np.array(stdR50N_all)

a=np.column_stack([abbrevs,meanR50H_all,stdR50H_all,meanR50S_all,stdR5
0S_all,meanR50N_all,stdR50N_all])

df = pd.DataFrame(a)
df.columns=["spatial","meanR50H","stdR50H","meanR50S","stdR50S","meanR
50N","stdR50N"]
```

Bleaching Link Removal: Uses the bleaching removal method of removing links but also tracks links removed at the same time

In [10]:
```
def bleaching_w_links(G,edges,nettype):

    G.remove_nodes_from(nx.isolates(G)) #get rid of isolates already p
resent
    #create a bunch of empty lists for results
    N_g1= []
    nodesremoved=[]
    iso_hosts=[]
    iso_symbs=[]
    linksremoved=[]

    #get number of symbionts and hosts in the network for use if doing
a null model
```

```
    F,hedge_list=mygraph("Global_host_nodes.csv",edges,"symbionttolerances_init.csv")
    num_hosts=len(hostseq(F))
    num_symbs=len(symbseq(F))

    if nettype=="null":
        h=num_hosts
    else:
        h=730

    for i in xrange(0,50): #over the range of the temperature steps, do the following:
        G1=G.copy() #as a precaution, copy the graph so that you dont actually change the original one
        T=28+0.1*i #for each step, the change of T is 0.1
        links=0
        for j in G1.edges(): #look over the edges
            if G1.edge[j[0]][j[1]]['weight']<=T: #when T exceeds the threshold, remove link
                G1.remove_edge(j[0],j[1])
                links=links+1 #add to the number of links removed
        C=nx.connected_components(G1) #recalculate the connected components
        isolated_nodes=0 #Calculate the isolated nodes
        for m in C: #look over the components
            if len(m)==1: #if the component is just 1 node
                if m[0]<=h: #dont count the symbiont, just add the host nodes whose ID#s go up to 730
                    isolated_nodes=isolated_nodes+1 #update the isolated nodes size
        iso_hosts.append(isolated_nodes) #update isolated host nodes list
        N_g1.append(max(map(len, nx.connected_components(G1)))) #recalculate the giant component size
        isolates=nx.isolates(G1) #all the isolated nodes
        G1.remove_nodes_from(isolates) #remove the isolated nodes from the network
        nodesremoved.append(len(isolates)) #total number of nodes removed, including symbionts
        linksremoved.append(links)
        total_isos=nodesremoved
    for j in xrange(0,50):
        x=nodesremoved[j]-iso_hosts[j]
        iso_symbs.append(x)
    return N_g1,total_isos, iso_hosts, iso_symbs, linksremoved




def MultSimsRemovals_BleachLinks(edges,nettype):
    #G,edge_list=mygraph(hosts,edges,"symbionttolerances_init.csv")
```

```
    sims=100
    length=50
    results_isos_h=np.zeros((length,sims)) #need a blank matrix of col
num=sims and row num=number of temperature steps which is 50
    results_isos_s=np.zeros((length,sims))
    results_GC=np.zeros((length,sims))
    results_links=np.zeros((length,sims))
    results_isos_total=np.zeros((length,sims))

    if nettype=="null":
        for i in xrange(0,sims):
            G=get_biparnull_ndc(edges) #make network
            N_g1,total_isos, iso_hosts, iso_symbs, linksremoved = blea
ching_w_links(G,edges,nettype)
            results_isos_h[:,i]=iso_hosts
            results_GC[:,i]=N_g1
            results_isos_s[:,i]=iso_symbs
            results_links[:,i]=linksremoved
            results_isos_total[:,i]=total_isos

    if nettype=="net":
        for i in xrange(0,sims):
            tols="trial" + str(i+1) + ".csv" #call the right tolerance
file
            G,edge_list=mygraph("Global_host_nodes.csv",edges,tols) #m
ake network
            N_g1,total_isos, iso_hosts, iso_symbs, linksremoved = blea
ching_w_links(G,edges,nettype)
            results_isos_h[:,i]=iso_hosts
            results_GC[:,i]=N_g1
            results_isos_s[:,i]=iso_symbs
            results_links[:,i]=linksremoved
            results_isos_total[:,i]=total_isos

    return results_GC, results_isos_h, results_isos_s, results_isos_to
tal, results_links


def R50_bleach(edges,nettype):
    results_GC, results_isos_h, results_isos_s, results_isos_total, re
sults_links=MultSimsRemovals_BleachLinks(edges,nettype)

    G,edge_list=mygraph("Global_host_nodes.csv",edges,"symbionttoleran
ces_init.csv")
    numHosts=len(hostseq(G))
    numSymbs=len(symbseq(G))
    totedges=len(G.edges())
    numNodes=len(G.nodes())

    GC=results_GC
    GCstart=(GC[0,:])
    GCfrac=GC/GCstart
    meanGCfrac=np.mean(GCfrac,axis=1)
```

```
    percHostsRemoved=(results_isos_h/numHosts)*100
    percHostsRemaining=100-percHostsRemoved
    meanPHR=np.mean(percHostsRemaining,axis=1)

    percSymbsRemoved=(results_isos_s/numSymbs)*100
    percSymbsRemaining=100-percSymbsRemoved
    meanPSR=np.mean(percSymbsRemaining,axis=1)

    percNodesRemoved=(results_isos_total/numNodes)*100
    percNodesRemaining=100-percNodesRemoved
    meanPNR=np.mean(percNodesRemaining,axis=1)

    percLR=(results_links/totedges)*100
    numremovals=50

    LR_H50=[] #get hosts R50 values
    jtrack=0 #set initial J
    for j in xrange(0,100): #for each simulation do the following
        for i in xrange(1,numremovals): #for each of the removals do t
he following
            if j>jtrack: #so that dont get multiple values per simulat
ion
                if percHostsRemaining[i,j]<=50: #if the pHR of the rem
oval step is <=50
                    if percHostsRemaining[i-1,j]>=50: #and if the pHR
predecessor (previous removal step) was >=50
                        LR_H50.append(percLR[i,j])
                        jtrack=j #update j

    LR_S50=[] #get the symbionts R50 values
    jtrack=0
    for j in xrange(0,100):
        for i in xrange(1,numremovals):
            if j>jtrack:
                if percSymbsRemaining[i,j]<=50:
                    if percSymbsRemaining[i-1,j]>=50:
                        LR_S50.append(percLR[i,j])
                        jtrack=j
    LR_N50=[]
    jtrack=0
    for j in xrange(0,100):
        for i in xrange(1,numremovals):
            if j>jtrack:
                if percNodesRemaining[i,j]<=50:
                    if percNodesRemaining[i-1,j]>=50:
                        LR_N50.append(percLR[i,j])
                        jtrack=j


    percLR_SR50=list(LR_S50)
    percLR_HR50=list(LR_H50)
    percLR_NR50=list(LR_N50)
```

```
    meanR50symbs=np.mean(percLR_SR50)
    meanR50hosts=np.mean(percLR_HR50)
    meanR50total=np.mean(percLR_NR50)
    stdR50symbs=np.std(percLR_SR50)
    stdR50hosts=np.std(percLR_HR50)
    stdR50total=np.std(percLR_NR50)
    return(meanGCfrac,meanPHR,meanPSR,meanPNR,percLR,percLR_HR50,percL
R_SR50,percLR_NR50,meanR50hosts,stdR50hosts,meanR50symbs,stdR50symbs,
meanR50total,stdR50total)
```

Run the bleaching removals and save results

In [11]:
```
for i in xrange(0,14):
    meanGCfrac,meanPHR,meanPSR,meanPNR,percLR,percLR_HR50,percLR_SR50,
percLR_NR50,meanR50hosts,stdR50hosts,meanR50symbs,stdR50symbs, meanR50
total,stdR50total=R50_bleach(network_edges[i],"net")
    globals()['bleach_mGCfrac_%s' % abbrevs[i]]=meanGCfrac
    globals()['bleach_mpHR_%s' % abbrevs[i]]=meanPHR
    globals()['bleach_mpSR_%s' % abbrevs[i]]=meanPSR
    globals()['bleach_mpNR_%s' % abbrevs[i]]=meanPNR
    globals()['bleach_pLR_%s' % abbrevs[i]]=percLR
    globals()['bleach_R50H_%s' % abbrevs[i]]=percLR_HR50 #misleading b
ecause it is the fraction not percent value
    globals()['bleach_R50S_%s' % abbrevs[i]]=percLR_SR50
    globals()['bleach_R50N_%s' % abbrevs[i]]=percLR_NR50
    globals()['bleach_mR50H_%s' % abbrevs[i]]=meanR50hosts
    globals()['bleach_mR50S_%s' % abbrevs[i]]=meanR50symbs
    globals()['bleach_mR50N_%s' % abbrevs[i]]=meanR50total
    globals()['bleach_std50H_%s' % abbrevs[i]]=stdR50hosts
    globals()['bleach_std50S_%s' % abbrevs[i]]=stdR50symbs
    globals()['bleach_std50N_%s' % abbrevs[i]]=stdR50total
```

In [12]:
```
### save means and stdevs

meanR50H_bleach_all=[]
stdR50H_bleach_all=[]
meanR50S_bleach_all=[]
stdR50S_bleach_all=[]
meanR50N_bleach_all=[]
stdR50N_bleach_all=[]

for i in xrange(0,14):
    meanR50H_bleach_all.append(globals()['bleach_mR50H_%s' % abbrevs[i
]])
    meanR50S_bleach_all.append(globals()['bleach_mR50S_%s' % abbrevs[i
]])
    stdR50H_bleach_all.append(globals()['bleach_std50H_%s' % abbrevs[i
]])
    stdR50S_bleach_all.append(globals()['bleach_std50S_%s' % abbrevs[i
]])
    meanR50N_bleach_all.append(globals()['bleach_mR50N_%s' % abbrevs[i
]])
    stdR50N_bleach_all.append(globals()['bleach_std50N_%s' % abbrevs[i
]])


meanR50H_bleach_all=np.array(meanR50H_bleach_all)
stdR50H_bleach_all=np.array(stdR50H_bleach_all)
meanR50S_bleach_all=np.array(meanR50S_bleach_all)
stdR50S_bleach_all=np.array(stdR50S_bleach_all)
meanR50N_bleach_all=np.array(meanR50N_bleach_all)
stdR50N_bleach_all=np.array(stdR50N_bleach_all)

a=np.column_stack([abbrevs,meanR50H_bleach_all,stdR50H_bleach_all,mean
R50S_bleach_all,stdR50S_bleach_all,meanR50N_bleach_all,stdR50N_bleach_
all])

df = pd.DataFrame(a)
df.columns=["spatial","meanR50H","stdR50H","meanR50S","stdR50S","meanR
50N","stdR50N"]
```

Remove by Link tolerance functions: remove links by average tolerance, host tol only or symbiont tol only

In [13]:
```
def mygraphsanstemps(hosts,edges,tols,tol_who): #this function creates
a graph object from nodes,edges, and missing tolerances files

    x=nx.Graph() #create empty graph
    #get all the data imported
    hostnodes = pd.read_csv(hosts)
    symbnodesgood=pd.read_csv('Global_symbiontgood_nodes.csv') #the sy
mbionts that had tolerances listed in Swain et al. 2016a
```

```
    symbnodesrest=pd.read_csv('Global_symbiontbad_nodes.csv') #the one
s that didn't

    fittols=pd.read_csv(tols)
    #combine the tolerance file with the symbionts that need tolerance
s
    symbnodesrest['tols']=fittols['tolerance']
    #Add nodes into the graph with their attributes
    for row in hostnodes.iterrows():
        x.add_node(row[1][0], ocean=row[1][1], name=row[1][2],type=row
[1][3],genetic=row[1][4], tolerance=row[1][5])
    for row in symbnodesgood.iterrows():
        x.add_node(row[1][0], name=row[1][1],type=row[1][2],genetic=ro
w[1][3], tolerance=row[1][4])
    for row in symbnodesrest.iterrows():
        x.add_node(row[1][0], name=row[1][1],type=row[1][2],genetic=ro
w[1][3], tolerance=row[1][4])

    #now for the edges
    edges = pd.read_csv(edges,header=None) #ordered by ocean and then
by region in alphabetical order
    edge_list=[] #an empty list of edges
    thresh_list=[] #an empty list of thresholds
    for row in edges.iterrows():
        s=row[1][0] #symbiont ID is in first column
        h=row[1][1] #Host ID is n second column
        #MMM=row[1][2] #third column is the mean monthly max temperatu
re
        #get the tolerance values from the node attributes
        symb_tol=x.node[s]['tolerance']
        host_tol=x.node[h]['tolerance']
        #calculate the threshold for each edge based on node pairs
        if tol_who=="both":
            threshvalue=0.5*(symb_tol+host_tol)
        if tol_who=="hosts":
            threshvalue=host_tol
        if tol_who=="symbionts":
            threshvalue=symb_tol
        thresh_list.append(threshvalue)
        #update the edge list with a weight determined by thresholds
        edge_list.append((s,h,{'weight':threshvalue}))

    x.add_edges_from(edge_list) #add edges to the graph object
    x.remove_nodes_from(nx.isolates(x)) #there's a few nodes that dont
actually have edges, and this was easier than going back through the o
riginal excel

    return x,edge_list


def removelinkbytolerance_net(G,edges,direction):
    #this will sort the edges by their weights
    G.remove_nodes_from(nx.isolates(G))
```

```
    edge_list=G.edges(data=True)
    targets=sorted(edge_list,key=lambda G: G[2]['weight'], reverse=dir
ection)
    #print targets
    N_g = []
    iso_h=[]
    iso_s=[]
    total_isos=[]

    F,hedge_list=mygraph("Global_host_nodes.csv",edges,"symbionttolera
nces_init.csv")
    num_hosts=len(hostseq(F))
    num_symbs=len(symbseq(F))

    h=730

    #get the initial sizes of things
    N_g.append(max(map(len, nx.connected_components(G))))
    iso_h.append(0)
    iso_s.append(0)
    total_isos.append(0)

    while len(targets) > 2:
        a=targets[0][0]
        b=targets[0][1]
        targets.remove(targets[0])
        G.remove_edge(a,b)
        #G.remove_node(targets[0])

        N_g.append(max(map(len, nx.connected_components(G))))
        C=nx.connected_components(G) #recalculate the connected compon
ents
        isolated_hosts=0
        isolated_symbs=0
        for m in C: #look over the components
            if len(m)==1: #if the component is just 1 node
                if m[0]<=h: #dont count the symbiont, just add the hos
t nodes whose ID#s go up to 730
                    isolated_hosts=isolated_hosts+1 #update the isolat
ed host nodes size
                else:
                    isolated_symbs=isolated_symbs+1
        iso_h.append(isolated_hosts) #update isolated host nodes list
        iso_s.append(isolated_symbs)
        total_isos.append(isolated_hosts+isolated_symbs)

    if len(N_g)<len(F.edges()):
        x=len(F.edges())-len(N_g)
        for i in xrange(0,x):
            N_g.append(0)
    if len(iso_h)<len(F.edges()):
        y=len(F.edges())-len(iso_h)
        for i in xrange(0,y):
```

```
            iso_h.append(num_hosts)
    if len(iso_s)<len(F.edges()):
        z=len(F.edges())-len(iso_s)
        for i in xrange(0,z):
            iso_s.append(num_symbs)
    if len(total_isos)<len(F.edges()):
        a=len(F.edges())-len(total_isos)
        for i in xrange(0,a):
            total_isos.append(len(F.nodes()))


    return N_g, iso_h, iso_s, total_isos


def MultSimsRemovals_LT(edges,direction,tol_who):
    sims=100
    hosts="Global_host_nodes.csv"
    #setup results arrays
    G1,edge_list=mygraph(hosts,edges,"symbionttolerances_init.csv")
    length=len(G1.edges())
    results_isos_s=np.zeros((length,sims))
    results_isos_h=np.zeros((length,sims))
    results_isos_total=np.zeros((length,sims))
    results_GC=np.zeros((length,sims))

    for i in xrange(0,sims): #for all the sims do the following
        tols="trial" + str(i+1) + ".csv" #call the right tolerance fil
e
        G,edge_list=mygraphsanstemps(hosts,edges,tols,tol_who) #make n
etwork
        N_g, iso_h, iso_s, total_isos=removelinkbytolerance_net(G,edge
s,direction)
        results_GC[:,i]=N_g
        results_isos_h[:,i]=iso_h
        results_isos_s[:,i]=iso_s
        results_isos_total[:,i]=total_isos



    return results_GC, results_isos_h, results_isos_s, results_isos_to
tal



def R50_LT(edges, direction, tol_who):
    results_GC, iso_h, iso_s, iso_total=MultSimsRemovals_LT(edges,dire
ction,tol_who)
    G,edge_list=mygraph("Global_host_nodes.csv",edges,"symbiontoleran
ces_init.csv")
    numHosts=len(hostseq(G))
    numSymbs=len(symbseq(G))
    numNodes=len(G.nodes())
    totedges=len(G.edges())
```

```
    #print totedges, "totedges"

    GC=results_GC
    GCstart=(GC[0,:])
    GCfrac=GC/GCstart
    meanGCfrac=np.mean(GCfrac,axis=1)

    percHostsRemoved=(iso_h/numHosts)*100
    percHostsRemaining=100-percHostsRemoved
    meanPHR=np.mean(percHostsRemaining,axis=1)
    #print np.shape(percHostsRemaining)

    percSymbsRemoved=(iso_s/numSymbs)*100
    percSymbsRemaining=100-percSymbsRemoved
    meanPSR=np.mean(percSymbsRemaining,axis=1)
    #print np.shape(percSymbsRemaining)

    percNodesRemoved=(iso_total/numNodes)*100
    percNodesRemaining=100-percNodesRemoved
    meanPNR=np.mean(percNodesRemaining,axis=1)
    #print np.shape(percNodesRemaining)
    #print percNodesRemaining

    LR=list(xrange(0,len(iso_total)))
    LR=np.array(LR,dtype='float')
    percLR=(LR/totedges)*100
    numremovals=len(iso_total)
    #print numremovals, "numremovals"

    LR_H50=[] #get hosts R50 values
    jtrack=0 #set initial J
    for j in xrange(0,100): #for each simulation do the following
        for i in xrange(1,numremovals): #for each of the removals do t
he following
            if j>jtrack: #so that dont get multiple values per simulat
ion
                if percHostsRemaining[i,j]<=50: #if the pHR of the rem
oval step is <=50
                    if percHostsRemaining[i-1,j]>=50: #and if the pHR
predecessor (previous removal step) was >=50
                        LR_H50.append(i) #the R50 values for this simu
lations is this removal step which
                                    #corresponds to how many links hav
e been removed so far
                        jtrack=j #update j

    LR_S50=[] #get the symbionts R50 values
    jtrack=0
    for j in xrange(0,100):
        for i in xrange(1,numremovals):
            if j>jtrack:
                if percSymbsRemaining[i,j]<=50:
                    if percSymbsRemaining[i-1,j]>=50:
```

```
                        LR_S50.append(i)
                        jtrack=j
    LR_N50=[]
    jtrack=0
    for j in xrange(0,100):
        for i in xrange(1,numremovals):
            if j>jtrack:
                if percNodesRemaining[i,j]<=50:
                    if percNodesRemaining[i-1,j]>=50:
                        LR_N50.append(i)
                        jtrack=j

    #print len(LR_N50), "NR50 length"
    #print len(LR_H50), "HR50 length"
    #print len(LR_S50), "SR50 length"
    percLR_SR50=list(np.divide(LR_S50,totedges,dtype=float))
    percLR_HR50=list(np.divide(LR_H50,totedges,dtype=float))
    percLR_NR50=list(np.divide(LR_N50,totedges,dtype=float))
    meanR50symbs=np.mean(percLR_SR50)
    meanR50hosts=np.mean(percLR_HR50)
    meanR50total=np.mean(percLR_NR50)
    stdR50symbs=np.std(percLR_SR50)
    stdR50hosts=np.std(percLR_HR50)
    stdR50total=np.std(percLR_NR50)
    return(meanGCfrac,meanPHR,meanPSR,meanPNR,percLR,percLR_HR50,percL
R_SR50,percLR_NR50,meanR50hosts,stdR50hosts,meanR50symbs,stdR50symbs,
meanR50total,stdR50total)
```

In [15]:

```
##RUN ALL THE THINGS.
length=14
tol_who="both"
direction=True
for i in xrange(0,length):
    meanGCfrac,meanPHR,meanPSR,meanPNR,percLR,percLR_HR50,percLR_SR50,
percLR_NR50,meanR50hosts,stdR50hosts,meanR50symbs,stdR50symbs, meanR50
total,stdR50total=R50_LT(network_edges[i],direction,tol_who)
    globals()['LT_BH_mGCfrac_%s' % abbrevs[i]]=meanGCfrac
    globals()['LT_BH_mpHR_%s' % abbrevs[i]]=meanPHR
    globals()['LT_BH_mpSR_%s' % abbrevs[i]]=meanPSR
    globals()['LT_BH_mpNR_%s' % abbrevs[i]]=meanPNR
    globals()['LT_BH_pLR_%s' % abbrevs[i]]=percLR
    globals()['LT_BH_R50H_%s' % abbrevs[i]]=percLR_HR50 #misleading be
cause it is the fraction not percent value
    globals()['LT_BH_R50S_%s' % abbrevs[i]]=percLR_SR50
    globals()['LT_BH_R50N_%s' % abbrevs[i]]=percLR_NR50
    globals()['LT_BH_mR50H_%s' % abbrevs[i]]=meanR50hosts
    globals()['LT_BH_mR50S_%s' % abbrevs[i]]=meanR50symbs
    globals()['LT_BH_mR50N_%s' % abbrevs[i]]=meanR50total
    globals()['LT_BH_std50H_%s' % abbrevs[i]]=stdR50hosts
    globals()['LT_BH_std50S_%s' % abbrevs[i]]=stdR50symbs
    globals()['LT_BH_std50N_%s' % abbrevs[i]]=stdR50total
tol_who="both"
direction=False
```

```
for i in xrange(0,length):
    meanGCfrac,meanPHR,meanPSR,meanPNR,percLR,percLR_HR50,percLR_SR50,
percLR_NR50,meanR50hosts,stdR50hosts,meanR50symbs,stdR50symbs, meanR50
total,stdR50total=R50_LT(network_edges[i],direction,tol_who)
    globals()['LT_BL_mGCfrac_%s' % abbrevs[i]]=meanGCfrac
    globals()['LT_BL_mpHR_%s' % abbrevs[i]]=meanPHR
    globals()['LT_BL_mpSR_%s' % abbrevs[i]]=meanPSR
    globals()['LT_BL_mpNR_%s' % abbrevs[i]]=meanPNR
    globals()['LT_BL_pLR_%s' % abbrevs[i]]=percLR
    globals()['LT_BL_R50H_%s' % abbrevs[i]]=percLR_HR50 #misleading be
cause it is the fraction not percent value
    globals()['LT_BL_R50S_%s' % abbrevs[i]]=percLR_SR50
    globals()['LT_BL_R50N_%s' % abbrevs[i]]=percLR_NR50
    globals()['LT_BL_mR50H_%s' % abbrevs[i]]=meanR50hosts
    globals()['LT_BL_mR50S_%s' % abbrevs[i]]=meanR50symbs
    globals()['LT_BL_mR50N_%s' % abbrevs[i]]=meanR50total
    globals()['LT_BL_std50H_%s' % abbrevs[i]]=stdR50hosts
    globals()['LT_BL_std50S_%s' % abbrevs[i]]=stdR50symbs
    globals()['LT_BL_std50N_%s' % abbrevs[i]]=stdR50total



tol_who="symbionts"
direction=True
for i in xrange(0,length):
    meanGCfrac,meanPHR,meanPSR,meanPNR,percLR,percLR_HR50,percLR_SR50,
percLR_NR50,meanR50hosts,stdR50hosts,meanR50symbs,stdR50symbs, meanR50
total,stdR50total=R50_LT(network_edges[i],direction,tol_who)
    globals()['LT_SH_mGCfrac_%s' % abbrevs[i]]=meanGCfrac
    globals()['LT_SH_mpHR_%s' % abbrevs[i]]=meanPHR
    globals()['LT_SH_mpSR_%s' % abbrevs[i]]=meanPSR
    globals()['LT_SH_mpNR_%s' % abbrevs[i]]=meanPNR
    globals()['LT_SH_pLR_%s' % abbrevs[i]]=percLR
    globals()['LT_SH_R50H_%s' % abbrevs[i]]=percLR_HR50 #misleading be
cause it is the fraction not percent value
    globals()['LT_SH_R50S_%s' % abbrevs[i]]=percLR_SR50
    globals()['LT_SH_R50N_%s' % abbrevs[i]]=percLR_NR50
    globals()['LT_SH_mR50H_%s' % abbrevs[i]]=meanR50hosts
    globals()['LT_SH_mR50S_%s' % abbrevs[i]]=meanR50symbs
    globals()['LT_SH_mR50N_%s' % abbrevs[i]]=meanR50total
    globals()['LT_SH_std50H_%s' % abbrevs[i]]=stdR50hosts
    globals()['LT_SH_std50S_%s' % abbrevs[i]]=stdR50symbs
    globals()['LT_SH_std50N_%s' % abbrevs[i]]=stdR50total
tol_who="symbionts"
direction=False
for i in xrange(0,length):
    meanGCfrac,meanPHR,meanPSR,meanPNR,percLR,percLR_HR50,percLR_SR50,
percLR_NR50,meanR50hosts,stdR50hosts,meanR50symbs,stdR50symbs, meanR50
total,stdR50total=R50_LT(network_edges[i],direction,tol_who)
    globals()['LT_SL_mGCfrac_%s' % abbrevs[i]]=meanGCfrac
    globals()['LT_SL_mpHR_%s' % abbrevs[i]]=meanPHR
    globals()['LT_SL_mpSR_%s' % abbrevs[i]]=meanPSR
    globals()['LT_SL_mpNR_%s' % abbrevs[i]]=meanPNR
```

```
    globals()['LT_SL_pLR_%s' % abbrevs[i]]=percLR
    globals()['LT_SL_R50H_%s' % abbrevs[i]]=percLR_HR50 #misleading be
cause it is the fraction not percent value
    globals()['LT_SL_R50S_%s' % abbrevs[i]]=percLR_SR50
    globals()['LT_SL_R50N_%s' % abbrevs[i]]=percLR_NR50
    globals()['LT_SL_mR50H_%s' % abbrevs[i]]=meanR50hosts
    globals()['LT_SL_mR50S_%s' % abbrevs[i]]=meanR50symbs
    globals()['LT_SL_mR50N_%s' % abbrevs[i]]=meanR50total
    globals()['LT_SL_std50H_%s' % abbrevs[i]]=stdR50hosts
    globals()['LT_SL_std50S_%s' % abbrevs[i]]=stdR50symbs
    globals()['LT_SL_std50N_%s' % abbrevs[i]]=stdR50total




tol_who="hosts"
direction=True
for i in xrange(0,length):
    meanGCfrac,meanPHR,meanPSR,meanPNR,percLR,percLR_HR50,percLR_SR50,
percLR_NR50,meanR50hosts,stdR50hosts,meanR50symbs,stdR50symbs, meanR50
total,stdR50total=R50_LT(network_edges[i],direction,tol_who)
    globals()['LT_HH_mGCfrac_%s' % abbrevs[i]]=meanGCfrac
    globals()['LT_HH_mpHR_%s' % abbrevs[i]]=meanPHR
    globals()['LT_HH_mpSR_%s' % abbrevs[i]]=meanPSR
    globals()['LT_HH_mpNR_%s' % abbrevs[i]]=meanPNR
    globals()['LT_HH_pLR_%s' % abbrevs[i]]=percLR
    globals()['LT_HH_R50H_%s' % abbrevs[i]]=percLR_HR50 #misleading be
cause it is the fraction not percent value
    globals()['LT_HH_R50S_%s' % abbrevs[i]]=percLR_SR50
    globals()['LT_HH_R50N_%s' % abbrevs[i]]=percLR_NR50
    globals()['LT_HH_mR50H_%s' % abbrevs[i]]=meanR50hosts
    globals()['LT_HH_mR50S_%s' % abbrevs[i]]=meanR50symbs
    globals()['LT_HH_mR50N_%s' % abbrevs[i]]=meanR50total
    globals()['LT_HH_std50H_%s' % abbrevs[i]]=stdR50hosts
    globals()['LT_HH_std50S_%s' % abbrevs[i]]=stdR50symbs
    globals()['LT_HH_std50N_%s' % abbrevs[i]]=stdR50total
tol_who="hosts"
direction=False
for i in xrange(0,length):
    meanGCfrac,meanPHR,meanPSR,meanPNR,percLR,percLR_HR50,percLR_SR50,
percLR_NR50,meanR50hosts,stdR50hosts,meanR50symbs,stdR50symbs, meanR50
total,stdR50total=R50_LT(network_edges[i],direction,tol_who)
    globals()['LT_HL_mGCfrac_%s' % abbrevs[i]]=meanGCfrac
    globals()['LT_HL_mpHR_%s' % abbrevs[i]]=meanPHR
    globals()['LT_HL_mpSR_%s' % abbrevs[i]]=meanPSR
    globals()['LT_HL_mpNR_%s' % abbrevs[i]]=meanPNR
    globals()['LT_HL_pLR_%s' % abbrevs[i]]=percLR
    globals()['LT_HL_R50H_%s' % abbrevs[i]]=percLR_HR50 #misleading be
cause it is the fraction not percent value
    globals()['LT_HL_R50S_%s' % abbrevs[i]]=percLR_SR50
    globals()['LT_HL_R50N_%s' % abbrevs[i]]=percLR_NR50
    globals()['LT_HL_mR50H_%s' % abbrevs[i]]=meanR50hosts
    globals()['LT_HL_mR50S_%s' % abbrevs[i]]=meanR50symbs
```

```
    globals()['LT_HL_mR50N_%s' % abbrevs[i]]=meanR50total
    globals()['LT_HL_std50H_%s' % abbrevs[i]]=stdR50hosts
    globals()['LT_HL_std50S_%s' % abbrevs[i]]=stdR50symbs
    globals()['LT_HL_std50N_%s' % abbrevs[i]]=stdR50total
```

In [16]:

```
### Save the tolerance removed mean R50s and stdevs

meanR50H_all=[]
stdR50H_all=[]
meanR50S_all=[]
stdR50S_all=[]
meanR50N_all=[]
stdR50N_all=[]

for i in xrange(0,14):
    meanR50H_all.append(globals()['LT_BH_mR50H_%s' % abbrevs[i]])
    meanR50S_all.append(globals()['LT_BH_mR50S_%s' % abbrevs[i]])
    stdR50H_all.append(globals()['LT_BH_std50H_%s' % abbrevs[i]])
    stdR50S_all.append(globals()['LT_BH_std50S_%s' % abbrevs[i]])
    meanR50N_all.append(globals()['LT_BH_mR50N_%s' % abbrevs[i]])
    stdR50N_all.append(globals()['LT_BH_std50N_%s' % abbrevs[i]])


meanR50H_all=np.array(meanR50H_all)
stdR50H_all=np.array(stdR50H_all)
meanR50S_all=np.array(meanR50S_all)
stdR50S_all=np.array(stdR50S_all)
meanR50N_all=np.array(meanR50N_all)
stdR50N_all=np.array(stdR50N_all)

a=np.column_stack([abbrevs,meanR50H_all,stdR50H_all,meanR50S_all,stdR5
0S_all,meanR50N_all,stdR50N_all])

df = pd.DataFrame(a)
df.columns=["spatial","meanR50H","stdR50H","meanR50S","stdR50S","meanR
50N","stdR50N"]



meanR50H_all=[]
stdR50H_all=[]
meanR50S_all=[]
stdR50S_all=[]
meanR50N_all=[]
stdR50N_all=[]

for i in xrange(0,14):
    meanR50H_all.append(globals()['LT_BL_mR50H_%s' % abbrevs[i]])
    meanR50S_all.append(globals()['LT_BL_mR50S_%s' % abbrevs[i]])
    stdR50H_all.append(globals()['LT_BL_std50H_%s' % abbrevs[i]])
    stdR50S_all.append(globals()['LT_BL_std50S_%s' % abbrevs[i]])
    meanR50N_all.append(globals()['LT_BL_mR50N_%s' % abbrevs[i]])
    stdR50N_all.append(globals()['LT_BL_std50N_%s' % abbrevs[i]])
```

```
meanR50H_all=np.array(meanR50H_all)
stdR50H_all=np.array(stdR50H_all)
meanR50S_all=np.array(meanR50S_all)
stdR50S_all=np.array(stdR50S_all)
meanR50N_all=np.array(meanR50N_all)
stdR50N_all=np.array(stdR50N_all)

a=np.column_stack([abbrevs,meanR50H_all,stdR50H_all,meanR50S_all,stdR5
0S_all,meanR50N_all,stdR50N_all])

df = pd.DataFrame(a)
df.columns=["spatial","meanR50H","stdR50H","meanR50S","stdR50S","meanR
50N","stdR50N"]



meanR50H_all=[]
stdR50H_all=[]
meanR50S_all=[]
stdR50S_all=[]
meanR50N_all=[]
stdR50N_all=[]

for i in xrange(0,14):
    meanR50H_all.append(globals()['LT_SH_mR50H_%s' % abbrevs[i]])
    meanR50S_all.append(globals()['LT_SH_mR50S_%s' % abbrevs[i]])
    stdR50H_all.append(globals()['LT_SH_std50H_%s' % abbrevs[i]])
    stdR50S_all.append(globals()['LT_SH_std50S_%s' % abbrevs[i]])
    meanR50N_all.append(globals()['LT_SH_mR50N_%s' % abbrevs[i]])
    stdR50N_all.append(globals()['LT_SH_std50N_%s' % abbrevs[i]])


meanR50H_all=np.array(meanR50H_all)
stdR50H_all=np.array(stdR50H_all)
meanR50S_all=np.array(meanR50S_all)
stdR50S_all=np.array(stdR50S_all)
meanR50N_all=np.array(meanR50N_all)
stdR50N_all=np.array(stdR50N_all)

a=np.column_stack([abbrevs,meanR50H_all,stdR50H_all,meanR50S_all,stdR5
0S_all,meanR50N_all,stdR50N_all])

df = pd.DataFrame(a)
df.columns=["spatial","meanR50H","stdR50H","meanR50S","stdR50S","meanR
50N","stdR50N"]



meanR50H_all=[]
stdR50H_all=[]
meanR50S_all=[]
```

```
stdR50S_all=[]
meanR50N_all=[]
stdR50N_all=[]

for i in xrange(0,14):
    meanR50H_all.append(globals()['LT_SL_mR50H_%s' % abbrevs[i]])
    meanR50S_all.append(globals()['LT_SL_mR50S_%s' % abbrevs[i]])
    stdR50H_all.append(globals()['LT_SL_std50H_%s' % abbrevs[i]])
    stdR50S_all.append(globals()['LT_SL_std50S_%s' % abbrevs[i]])
    meanR50N_all.append(globals()['LT_SL_mR50N_%s' % abbrevs[i]])
    stdR50N_all.append(globals()['LT_SL_std50N_%s' % abbrevs[i]])


meanR50H_all=np.array(meanR50H_all)
stdR50H_all=np.array(stdR50H_all)
meanR50S_all=np.array(meanR50S_all)
stdR50S_all=np.array(stdR50S_all)
meanR50N_all=np.array(meanR50N_all)
stdR50N_all=np.array(stdR50N_all)

a=np.column_stack([abbrevs,meanR50H_all,stdR50H_all,meanR50S_all,stdR5
0S_all,meanR50N_all,stdR50N_all])

df = pd.DataFrame(a)
df.columns=["spatial","meanR50H","stdR50H","meanR50S","stdR50S","meanR
50N","stdR50N"]



meanR50H_all=[]
stdR50H_all=[]
meanR50S_all=[]
stdR50S_all=[]
meanR50N_all=[]
stdR50N_all=[]

for i in xrange(0,14):
    meanR50H_all.append(globals()['LT_HH_mR50H_%s' % abbrevs[i]])
    meanR50S_all.append(globals()['LT_HH_mR50S_%s' % abbrevs[i]])
    stdR50H_all.append(globals()['LT_HH_std50H_%s' % abbrevs[i]])
    stdR50S_all.append(globals()['LT_HH_std50S_%s' % abbrevs[i]])
    meanR50N_all.append(globals()['LT_HH_mR50N_%s' % abbrevs[i]])
    stdR50N_all.append(globals()['LT_HH_std50N_%s' % abbrevs[i]])


meanR50H_all=np.array(meanR50H_all)
stdR50H_all=np.array(stdR50H_all)
meanR50S_all=np.array(meanR50S_all)
stdR50S_all=np.array(stdR50S_all)
meanR50N_all=np.array(meanR50N_all)
stdR50N_all=np.array(stdR50N_all)

a=np.column_stack([abbrevs,meanR50H_all,stdR50H_all,meanR50S_all,stdR5
```

```
0S_all,meanR50N_all,stdR50N_all])

df = pd.DataFrame(a)
df.columns=["spatial","meanR50H","stdR50H","meanR50S","stdR50S","meanR
50N","stdR50N"]



meanR50H_all=[]
stdR50H_all=[]
meanR50S_all=[]
stdR50S_all=[]
meanR50N_all=[]
stdR50N_all=[]

for i in xrange(0,14):
    meanR50H_all.append(globals()['LT_HL_mR50H_%s' % abbrevs[i]])
    meanR50S_all.append(globals()['LT_HL_mR50S_%s' % abbrevs[i]])
    stdR50H_all.append(globals()['LT_HL_std50H_%s' % abbrevs[i]])
    stdR50S_all.append(globals()['LT_HL_std50S_%s' % abbrevs[i]])
    meanR50N_all.append(globals()['LT_HL_mR50N_%s' % abbrevs[i]])
    stdR50N_all.append(globals()['LT_HL_std50N_%s' % abbrevs[i]])


meanR50H_all=np.array(meanR50H_all)
stdR50H_all=np.array(stdR50H_all)
meanR50S_all=np.array(meanR50S_all)
stdR50S_all=np.array(stdR50S_all)
meanR50N_all=np.array(meanR50N_all)
stdR50N_all=np.array(stdR50N_all)

a=np.column_stack([abbrevs,meanR50H_all,stdR50H_all,meanR50S_all,stdR5
0S_all,meanR50N_all,stdR50N_all])

df = pd.DataFrame(a)
df.columns=["spatial","meanR50H","stdR50H","meanR50S","stdR50S","meanR
50N","stdR50N"]
```

Now for the node removal models!

- Random removals
- Degree based removals
- tolerance based removals

Random Removal: randomly shuffles the order of the node list and removes nodes from the top of the list until there are just two nodes left.

In [17]:
```
def removeRandomNodes(G, who,nettype,edges):
```

```
    G.remove_nodes_from(nx.isolates(G)) #get rid of isolates already p
resent
    node_list=G.nodes() #get list of nodes
    #make some empty lists for the results
    N_g = []
    iso_h=[]
    iso_s=[]
    rem_h=[]
    rem_s=[]
    targets_hosts=[]
    targets_symbs=[]

    random.shuffle(node_list) #shuffle the node list
    targets=node_list #make nodelist the target

    #get some info about the original graph
    F,hedge_list=mygraph("Global_host_nodes.csv",edges,"symbionttolera
nces_init.csv")
    num_hosts=len(hostseq(F))
    num_symbs=len(symbseq(F))
    #set the number of hosts
    if nettype=="null":
        h=num_hosts
        s=num_symbs
    else:
        h=730

    #split the targets into symbionts and hosts for when removing just
those
    for t in targets:
        if t<=h:
            targets_hosts.append(t)
        else:
            targets_symbs.append(t)
    if who=='both':
        targets=targets
    if who=='symbionts':
        targets=targets_symbs
    if who=='hosts':
        targets=targets_hosts
    #set initial removed
    removed_hosts=0
    removed_symbs=0

    #start the removal
    while len(targets) > 2:
        G.remove_node(targets[0]) #remove the top of the target list n
ode from the graph

        if targets[0] <=h:
            removed_hosts=removed_hosts+1 #updated removed hosts list
        else:
            removed_symbs=removed_symbs+1 #update removed symbs list
```

```
        N_g.append(max(map(len, nx.connected_components(G)))) #update the giant component size
        targets.remove(targets[0]) #remove the target node from the target list

        #now get the isolated hosts and total isolates
        C=nx.connected_components(G) #recalculate the connected components
        #set the initial number of isolated nodes
        isolated_hosts=0
        isolated_symbs=0
        for m in C: #look over the components
            if len(m)==1: #if the component is just 1 node
                if m[0]<=h: #dont count the symbiont, just add the host nodes whose ID#s go up to 730
                    isolated_hosts=isolated_hosts+1 #update the isolated host nodes size

                else:
                    isolated_symbs=isolated_symbs+1
        #update all the results list
        iso_h.append(isolated_hosts)
        iso_s.append(isolated_symbs)
        rem_h.append(removed_hosts)
        rem_s.append(removed_symbs)
    #ok so the isos just track who was isolated due to a removal, and the removed tracks who was removed
    #need to combine into a total_removed category, and this is where we can get the total removed of both
    a=iso_h
    b=rem_h
    totrem_h = [x+y for x,y in zip(a, b)]
    a=iso_s
    b=rem_s
    totrem_s = [x+y for x,y in zip(a, b)]
    totrem_both = [x+y for x,y in zip(totrem_s,totrem_h)]
    numremovals=list(xrange(1,len(totrem_both)+1))
    if len(numremovals)<len(F.nodes()):
        a=len(F.nodes())-len(numremovals)
        numremovals2=numremovals
        for i in xrange(0,a):
            totrem_h.append(totrem_h[len(numremovals)-1])
            totrem_s.append(totrem_s[len(numremovals)-1])
            totrem_both.append(totrem_both[len(numremovals)-1])
            N_g.append(N_g[len(numremovals)-1])
            numremovals2.append(numremovals[len(numremovals)-1])
    return N_g, iso_h, iso_s, totrem_h, totrem_s, totrem_both,numremovals2
```

Degree removal: remove nodes by degree, can remove both, just hosts, or just symbionts AND can sort by high or low degree

In [18]:

```
#Need the mydegsort function to sort the degrees so that each simulati
on the order of degrees removed in the same degree class is random.
def mydegsort(G,direction):
    degrees=G.degree().values()
    degrand=[x+random.random() for x in degrees]
    nodes=G.nodes()
    rands={z[0]:list(z[1:]) for z in zip(nodes,degrand)}
    for node in G.nodes():
        x=random.random()
        nx.set_node_attributes(G,'rand',rands)
    degswrand=nx.get_node_attributes(G,'rand')
    targets=sorted(degswrand,key=degswrand.__getitem__,reverse=directi
on)
    return targets

def removebydegree(G,direction, who, nettype, edges):
    #set up the blanks
    G.remove_nodes_from(nx.isolates(G)) #get rid of isolates already p
resent
    N_g = []
    iso_h=[]
    iso_s=[]
    rem_s=[]
    rem_h=[]
    #get some info about the original graph
    F,hedge_list=mygraph("Global_host_nodes.csv",edges,"symbionttolera
nces_init.csv")
    num_hosts=len(hostseq(F))
    num_symbs=len(symbseq(F))
    num_nodes=len(F.nodes())
    #set up hosts and symbs limits
    if nettype=="null":
        h=num_hosts
        s=num_symbs
    else:
        h=730
        s=981
    #sort the degrees
    targets=mydegsort(G,direction)
    targets_symbs=[]
    targets_hosts=[]
    for t in targets:
        if t<=h:
            targets_hosts.append(t) #cutout the symbionts
        else:
            targets_symbs.append(t) #keep the symbs and cut the hosts

    if who=='both':
```

```
        targets=targets
    if who=='hosts':
        targets=targets_hosts
    if who=='symbionts':
        targets=targets_symbs
    #start the removals
    removed_hosts=0
    removed_symbs=0
    while len(targets) > 1:
        G.remove_node(targets[0]) #remove the node from the top of the
targets list
        if targets[0] <=h: #if it's a host add to the removed host lis
t
            removed_hosts=removed_hosts+1
        else: #if not it's a symbiont and should be added to the remov
ed symbiont list
            removed_symbs=removed_symbs+1
        N_g.append(max(map(len, nx.connected_components(G)))) #update
the size of the gian component
        targets.remove(targets[0]) #remove the node from the target li
st

        #now update the isolated nodes
        C=nx.connected_components(G) #recalculate the connected compon
ents
        isolated_hosts=0
        isolated_symbs=0
        for m in C: #look over the components
            if len(m)==1: #if the component is just 1 node
                if m[0]<=h: #dont count the symbiont, just add the hos
t nodes whose ID#s go up to 730
                    isolated_hosts=isolated_hosts+1 #update the isolat
ed host nodes size
                else:
                    isolated_symbs=isolated_symbs+1
        iso_h.append(isolated_hosts) #update isolated and removed list
s
        iso_s.append(isolated_symbs)
        rem_h.append(removed_hosts)
        rem_s.append(removed_symbs)
    #at the end get the total removed lists
    totrem_h = [x+y for x,y in zip(iso_h, rem_h)]
    totrem_s = [x+y for x,y in zip(iso_s, rem_s)]
    totrem_both = [x+y for x,y in zip(totrem_s,totrem_h)]
    numremovals=list(xrange(1,len(totrem_both)+1))
    if len(numremovals)<len(F.nodes()):
        a=len(F.nodes())-len(numremovals)
        numremovals2=numremovals
        for i in xrange(0,a):
            totrem_h.append(totrem_h[len(numremovals)-1])
            totrem_s.append(totrem_s[len(numremovals)-1])
            totrem_both.append(totrem_both[len(numremovals)-1])
            N_g.append(N_g[len(numremovals)-1])
```

```
            numremovals2.append(numremovals[len(numremovals)-1])
    return N_g, iso_h, iso_s, totrem_h, totrem_s, totrem_both, numremo
vals2
```

Remove by tolerance: sort nodes by tolerance values then remove from top of the list.

In [19]:

```
def removebytolerance(G, direction, who, nettype,edges):
    G.remove_nodes_from(nx.isolates(G)) #get rid of isolates already p
resent
    tols=nx.get_node_attributes(G,'tolerance') #get list of tolerances
    #set up the blanks
    N_g = []
    iso_h=[]
    iso_s=[]
    rem_s=[]
    rem_h=[]
    targets_hosts=[]
    targets_symbs=[]
    #sort the tolerances
    targets=sorted(tols,key=tols.__getitem__,reverse=direction)
    F,hedge_list=mygraph("Global_host_nodes.csv",edges,"symbionttolera
nces_init.csv")
    num_hosts=len(hostseq(F))
    num_symbs=len(symbseq(F))
    num_nodes=len(F.nodes())
    if nettype=="null":
        h=num_hosts
        s=num_symbs
    else:
        h=730
        s=981
    for t in targets:
        if t<=h:
            targets_hosts.append(t)
        else:
            targets_symbs.append(t)
    #pick which targets to use
    if who=='both':
        targets=targets
    if who=='symbionts':
        targets=targets_symbs
    if who=='hosts':
        targets=targets_hosts
    #start the removal
    removed_hosts=0
    removed_symbs=0
    while len(targets) > 2:
        G.remove_node(targets[0]) #remove the first target from networ
k
        #update the removed
```

```
        if targets[0] <=h:
            removed_hosts=removed_hosts+1
        else:
            removed_symbs=removed_symbs+1
        N_g.append(max(map(len, nx.connected_components(G)))) #update
the size of the giant component
        targets.remove(targets[0]) #remove the first target from the t
arget list

        #now get the isolated hosts and total isolates
        C=nx.connected_components(G) #recalculate the connected compon
ents
        isolated_hosts=0
        isolated_symbs=0
        for m in C: #look over the components
            if len(m)==1: #if the component is just 1 node
                if m[0]<=h: #dont count the symbiont, just add the hos
t nodes whose ID#s go up to 730
                    isolated_hosts=isolated_hosts+1 #update the isolat
ed host nodes size
                else:
                    isolated_symbs=isolated_symbs+1
        #update the isolates and removed lists
        iso_h.append(isolated_hosts)
        iso_s.append(isolated_symbs)
        rem_h.append(removed_hosts)
        rem_s.append(removed_symbs)
    #at the end get the total removed lists
    totrem_h = [x+y for x,y in zip(iso_h, rem_h)]
    totrem_s = [x+y for x,y in zip(iso_s, rem_s)]
    totrem_both = [x+y for x,y in zip(totrem_s,totrem_h)]
    numremovals=list(xrange(1,len(totrem_both)+1))
    if len(numremovals)<len(F.nodes()):
        a=len(F.nodes())-len(numremovals)
        numremovals2=numremovals
        for i in xrange(0,a):
            totrem_h.append(totrem_h[len(numremovals)-1])
            totrem_s.append(totrem_s[len(numremovals)-1])
            totrem_both.append(totrem_both[len(numremovals)-1])
            N_g.append(N_g[len(numremovals)-1])
            numremovals2.append(numremovals[len(numremovals)-1])
    return N_g, iso_h, iso_s, totrem_h, totrem_s, totrem_both, numremo
vals2
```

Ok, the removal models are coded, so now need a multiple simulation function

In [20]:
```
def MultSimsRemovals_nodes(edges, model, who, direction,nettype):
    sims=100 #set simulation number
    #makea graph to get the lengths for the result arrays
    G,edge_list=mygraph("Global_host_nodes.csv",edges,"symbionttoleran
```

```
ces_init.csv")
    #if who=="symbionts":
       # length=len(symbseq(G))-2 #it's -2 b/c models remove nodes unt
il 2 are left
    #if who=="hosts":
    #    length=len(hostseq(G))-2
   # if who=="both":
    length=len(G.nodes())
    #make results arrays
    results_GC=np.zeros((length,sims))
    results_totrem_h=np.zeros((length,sims))
    results_totrem_s=np.zeros((length,sims))
    results_totrem_both=np.zeros((length,sims))
    results_numremovals=np.zeros((length,sims))

    #pick which removal model to run...
    if model=="degree":
        for i in xrange(0,sims): #for all the sims do the following
            #pick the network type and run with it
            if nettype=="net":
                tols="trial" + str(i+1) + ".csv" #call the right toler
ance file
                G,edge_list=mygraph("Global_host_nodes.csv",edges,tols
) #make network
            else:
                G=get_biparnull_ndc(edges)
            #run the removal model
            N_g, iso_h, iso_s, totrem_h, totrem_s, totrem_both, numrem
ovals=removebydegree(G,direction, who, nettype, edges)
            #update the results arrays
            results_GC[:,i]=N_g
            results_totrem_h[:,i]=totrem_h
            results_totrem_s[:,i]=totrem_s
            results_totrem_both[:,i]=totrem_both
            results_numremovals[:,i]=numremovals

    if model=="random":
        for i in xrange(0,sims): #for all the sims do the following
            #pick the network type
            if nettype=="net":
                tols="trial" + str(i+1) + ".csv" #call the right toler
ance file
                G,edge_list=mygraph("Global_host_nodes.csv",edges,tols
) #make network
            else:
                G=get_biparnull_ndc(edges)
            #run the random node removal model
            N_g, iso_h, iso_s, totrem_h, totrem_s,totrem_both,numremov
als=removeRandomNodes(G, who,nettype,edges)
            #update results arrays
            results_GC[:,i]=N_g
            results_totrem_h[:,i]=totrem_h
            results_totrem_s[:,i]=totrem_s
```

```
            results_totrem_both[:,i]=totrem_both
            results_numremovals[:,i]=numremovals

    if model=="tolerance":
        #pick the network type
        for i in xrange(0,sims): #for all the sims do the following
            if nettype=="net":
                tols="trial" + str(i+1) + ".csv" #call the right toler
ance file
                G,edge_list=mygraph("Global_host_nodes.csv",edges,tols
) #make network
            else:
                G=get_biparnull_ndc(edges)
            #run the tolerance removal model
            N_g, iso_h, iso_s, totrem_h, totrem_s, totrem_both, numrem
ovals=removebytolerance(G, direction, who, nettype,edges)
            #update the results arrays
            results_GC[:,i]=N_g
            results_totrem_h[:,i]=totrem_h
            results_totrem_s[:,i]=totrem_s
            results_totrem_both[:,i]=totrem_both
            results_numremovals[:,i]=numremovals

    return  results_GC, results_totrem_h, results_totrem_s, results_t
otrem_both, results_numremovals
```

Now for the R50 function for nodes

In [21]:

```
def R50_nodes(edges, model, who, direction,nettype):
    results_GC, results_totrem_h, results_totrem_s, results_totrem_bot
h, results_numremovals=MultSimsRemovals_nodes(edges, model, who, direc
tion,nettype)
    #results_GC: number of nodes in giant component
    #results_totrem_h: number of hosts removed
    #results_totrem_s: number of symbionts removed
    #results_totrem_both: number of nodes removed
    #results_numremovals
    #get info about the network
    G,edge_list=mygraph("Global_host_nodes.csv",edges,"symbionttoleran
ces_init.csv")
    numHosts=len(hostseq(G))
    numSymbs=len(symbseq(G))
    totnodes=len(G.nodes())

    #the Giant Component results as fraction of initial size
    GC=results_GC
    GCstart=(GC[0,:])
    GCfrac=GC/GCstart
    meanGCfrac=np.mean(GCfrac,axis=1)
```

```
    #Hosts removed/remaining as percent
    percHostsRemoved=(results_totrem_h/numHosts)*100
    percHostsRemaining=100-percHostsRemoved
    mPHR=np.mean(percHostsRemaining,axis=1)

    #symbionts removed/remaining as percent
    percSymbsRemoved=(results_totrem_s/numSymbs)*100
    percSymbsRemaining=100-percSymbsRemoved
    mPSR=np.mean(percSymbsRemaining,axis=1)

    #all removed/remaining as percent
    percNodesRemoved=(results_totrem_both/totnodes)*100
    percNodesRemaining=100-percNodesRemoved
    mPNR=np.mean(percNodesRemaining,axis=1)

    if who =="hosts":
        total=numHosts
    if who=="both":
        total=totnodes
    if who=="symbionts":
        total=numSymbs

    percNR=(results_numremovals/total)*100
    percNR=np.mean(percNR,axis=1)
    numremovals=len(percNR)

#get the R50 values for Nodes (both), Hosts, and Symbionts separately
by marking which number of nodes removed corresponds ot the 50% thresh
old
    NR_H50=[] #get hosts R50 values
    jtrack=0 #set initial J
    for j in xrange(0,100): #for each simulation do the following
        for i in xrange(1,numremovals): #for each of the removals do t
he following
            if j>jtrack: #so that dont get multiple values per simulat
ion
                if percHostsRemaining[i,j]<=50: #if the pHR of the rem
oval step is <=50
                    if percHostsRemaining[i-1,j]>=50: #and if the pHR
predecessor (previous removal step) was >=50
                        NR_H50.append(i) #the R50 values for this simu
lations is this removal step which
                                         #corresponds to how many nodes hav
e been removed so far
                        jtrack=j #update j

    NR_S50=[] #get the symbionts R50 values
    jtrack=0
    for j in xrange(0,100):
        for i in xrange(1,numremovals):
            if j>jtrack:
                if percSymbsRemaining[i,j]<=50:
```

```
                    if percSymbsRemaining[i-1,j]>=50:
                        NR_S50.append(i)
                        jtrack=j
    NR_N50=[]
    jtrack=0
    for j in xrange(0,100):
        for i in xrange(1,numremovals):
            if j>jtrack:
                if percNodesRemaining[i,j]<=50:
                    if percNodesRemaining[i-1,j]>=50:
                        NR_N50.append(i)
                        jtrack=j
    if who =="hosts":
        totremoved=numHosts
    if who=="both":
        totremoved=totnodes
    if who=="symbionts":
        totremoved=numSymbs

    percNR_SR50=list(np.divide(NR_S50,totremoved,dtype=float))
    percNR_HR50=list(np.divide(NR_H50,totremoved,dtype=float))
    percNR_NR50=list(np.divide(NR_N50,totremoved,dtype=float))

    #If the nodes, hosts, or symbs remaining never went below 50% then
add extra 1's at the end of the R50 list to fill out
    if len(percNR_SR50)<99:
        a=99-len(percNR_SR50)
        for i in xrange(0,a):
            percNR_SR50.append(1)
    if len(percNR_HR50)<99:
        a=99-len(percNR_HR50)
        for i in xrange(0,a):
            percNR_HR50.append(1)
    if len(percNR_NR50)<99:
        a=99-len(percNR_NR50)
        for i in xrange(0,a):
            percNR_NR50.append(1)

    meanR50symbs=np.mean(percNR_SR50)
    meanR50hosts=np.mean(percNR_HR50)
    meanR50total=np.mean(percNR_NR50)
    stdR50symbs=np.std(percNR_SR50)
    stdR50hosts=np.std(percNR_HR50)
    stdR50total=np.std(percNR_NR50)

    return(meanGCfrac,mPHR,mPSR,mPNR,percNR,percNR_HR50,percNR_SR50,pe
rcNR_NR50,meanR50hosts,stdR50hosts,meanR50symbs,stdR50symbs, meanR50to
tal,stdR50total)
```

now run the node removals on the natural nets

[illegible]

In [22]:

```
#degree high hosts
model="degree"
who="hosts"
direction=True
nettype="net"

for i in xrange(0,length):
    meanGCfrac,meanPHR,meanPSR,meanPNR,percNR,percNR_HR50,percNR_SR50,
percNR_NR50,meanR50hosts,stdR50hosts,meanR50symbs,stdR50symbs, meanR50
total,stdR50total=R50_nodes(network_edges[i], model, who, direction,ne
ttype)
    globals()['DHH_mGCfrac_%s' % abbrevs[i]]=meanGCfrac
    globals()['DHH_mpHR_%s' % abbrevs[i]]=meanPHR
    globals()['DHH_mpSR_%s' % abbrevs[i]]=meanPSR
    globals()['DHH_mpNR_%s' % abbrevs[i]]=meanPNR
    globals()['DHH_pLR_%s' % abbrevs[i]]=percNR
    globals()['DHH_R50H_%s' % abbrevs[i]]=percNR_HR50 #misleading beca
use it is the fraction not percent value
    globals()['DHH_R50S_%s' % abbrevs[i]]=percNR_SR50
    globals()['DHH_R50N_%s' % abbrevs[i]]=percNR_NR50
    globals()['DHH_mR50H_%s' % abbrevs[i]]=meanR50hosts
    globals()['DHH_mR50S_%s' % abbrevs[i]]=meanR50symbs
    globals()['DHH_mR50N_%s' % abbrevs[i]]=meanR50total
    globals()['DHH_std50H_%s' % abbrevs[i]]=stdR50hosts
    globals()['DHH_std50S_%s' % abbrevs[i]]=stdR50symbs
    globals()['DHH_std50N_%s' % abbrevs[i]]=stdR50total

#Degree Low Hosts
model="degree"
who="hosts"
direction=False

for i in xrange(0,length):
    meanGCfrac,meanPHR,meanPSR,meanPNR,percNR,percNR_HR50,percNR_SR50,
percNR_NR50,meanR50hosts,stdR50hosts,meanR50symbs,stdR50symbs, meanR50
total,stdR50total=R50_nodes(network_edges[i], model, who, direction,ne
ttype)
    globals()['DLH_mGCfrac_%s' % abbrevs[i]]=meanGCfrac
    globals()['DLH_mpHR_%s' % abbrevs[i]]=meanPHR
    globals()['DLH_mpSR_%s' % abbrevs[i]]=meanPSR
    globals()['DLH_mpNR_%s' % abbrevs[i]]=meanPNR
    globals()['DLH_pLR_%s' % abbrevs[i]]=percNR
    globals()['DLH_R50H_%s' % abbrevs[i]]=percNR_HR50 #misleading beca
use it is the fraction not percent value
    globals()['DLH_R50S_%s' % abbrevs[i]]=percNR_SR50
    globals()['DLH_R50N_%s' % abbrevs[i]]=percNR_NR50
    globals()['DLH_mR50H_%s' % abbrevs[i]]=meanR50hosts
    globals()['DLH_mR50S_%s' % abbrevs[i]]=meanR50symbs
    globals()['DLH_mR50N_%s' % abbrevs[i]]=meanR50total
    globals()['DLH_std50H_%s' % abbrevs[i]]=stdR50hosts
    globals()['DLH_std50S_%s' % abbrevs[i]]=stdR50symbs
    globals()['DLH_std50N_%s' % abbrevs[i]]=stdR50total

#Degree High Symbionts
```

```
model="degree"
who="symbionts"
direction=True

for i in xrange(0,length):
    meanGCfrac,meanPHR,meanPSR,meanPNR,percNR,percNR_HR50,percNR_SR50,
percNR_NR50,meanR50hosts,stdR50hosts,meanR50symbs,stdR50symbs, meanR50
total,stdR50total=R50_nodes(network_edges[i], model, who, direction,ne
ttype)
    globals()['DHS_mGCfrac_%s' % abbrevs[i]]=meanGCfrac
    globals()['DHS_mpHR_%s' % abbrevs[i]]=meanPHR
    globals()['DHS_mpSR_%s' % abbrevs[i]]=meanPSR
    globals()['DHS_mpNR_%s' % abbrevs[i]]=meanPNR
    globals()['DHS_pLR_%s' % abbrevs[i]]=percNR
    globals()['DHS_R50H_%s' % abbrevs[i]]=percNR_HR50 #misleading beca
use it is the fraction not percent value
    globals()['DHS_R50S_%s' % abbrevs[i]]=percNR_SR50
    globals()['DHS_R50N_%s' % abbrevs[i]]=percNR_NR50
    globals()['DHS_mR50H_%s' % abbrevs[i]]=meanR50hosts
    globals()['DHS_mR50S_%s' % abbrevs[i]]=meanR50symbs
    globals()['DHS_mR50N_%s' % abbrevs[i]]=meanR50total
    globals()['DHS_std50H_%s' % abbrevs[i]]=stdR50hosts
    globals()['DHS_std50S_%s' % abbrevs[i]]=stdR50symbs
    globals()['DHS_std50N_%s' % abbrevs[i]]=stdR50total

#Degree Low Symbionts
model="degree"
who="symbionts"
direction=False

for i in xrange(0,length):
    meanGCfrac,meanPHR,meanPSR,meanPNR,percNR,percNR_HR50,percNR_SR50,
percNR_NR50,meanR50hosts,stdR50hosts,meanR50symbs,stdR50symbs, meanR50
total,stdR50total=R50_nodes(network_edges[i], model, who, direction,ne
ttype)
    globals()['DLS_mGCfrac_%s' % abbrevs[i]]=meanGCfrac
    globals()['DLS_mpHR_%s' % abbrevs[i]]=meanPHR
    globals()['DLS_mpSR_%s' % abbrevs[i]]=meanPSR
    globals()['DLS_mpNR_%s' % abbrevs[i]]=meanPNR
    globals()['DLS_pLR_%s' % abbrevs[i]]=percNR
    globals()['DLS_R50H_%s' % abbrevs[i]]=percNR_HR50 #misleading beca
use it is the fraction not percent value
    globals()['DLS_R50S_%s' % abbrevs[i]]=percNR_SR50
    globals()['DLS_R50N_%s' % abbrevs[i]]=percNR_NR50
    globals()['DLS_mR50H_%s' % abbrevs[i]]=meanR50hosts
    globals()['DLS_mR50S_%s' % abbrevs[i]]=meanR50symbs
    globals()['DLS_mR50N_%s' % abbrevs[i]]=meanR50total
    globals()['DLS_std50H_%s' % abbrevs[i]]=stdR50hosts
    globals()['DLS_std50S_%s' % abbrevs[i]]=stdR50symbs
    globals()['DLS_std50N_%s' % abbrevs[i]]=stdR50total

#Degree High Both
model="degree"
```

```
who="both"
direction=True

for i in xrange(0,length):
    meanGCfrac,meanPHR,meanPSR,meanPNR,percNR,percNR_HR50,percNR_SR50,
percNR_NR50,meanR50hosts,stdR50hosts,meanR50symbs,stdR50symbs, meanR50
total,stdR50total=R50_nodes(network_edges[i], model, who, direction,ne
ttype)
    globals()['DHB_mGCfrac_%s' % abbrevs[i]]=meanGCfrac
    globals()['DHB_mpHR_%s' % abbrevs[i]]=meanPHR
    globals()['DHB_mpSR_%s' % abbrevs[i]]=meanPSR
    globals()['DHB_mpNR_%s' % abbrevs[i]]=meanPNR
    globals()['DHB_pLR_%s' % abbrevs[i]]=percNR
    globals()['DHB_R50H_%s' % abbrevs[i]]=percNR_HR50 #misleading beca
use it is the fraction not percent value
    globals()['DHB_R50S_%s' % abbrevs[i]]=percNR_SR50
    globals()['DHB_R50N_%s' % abbrevs[i]]=percNR_NR50
    globals()['DHB_mR50H_%s' % abbrevs[i]]=meanR50hosts
    globals()['DHB_mR50S_%s' % abbrevs[i]]=meanR50symbs
    globals()['DHB_mR50N_%s' % abbrevs[i]]=meanR50total
    globals()['DHB_std50H_%s' % abbrevs[i]]=stdR50hosts
    globals()['DHB_std50S_%s' % abbrevs[i]]=stdR50symbs
    globals()['DHB_std50N_%s' % abbrevs[i]]=stdR50total

#degree low both
model="degree"
who="both"
direction=False

for i in xrange(0,length):
    meanGCfrac,meanPHR,meanPSR,meanPNR,percNR,percNR_HR50,percNR_SR50,
percNR_NR50,meanR50hosts,stdR50hosts,meanR50symbs,stdR50symbs, meanR50
total,stdR50total=R50_nodes(network_edges[i], model, who, direction,ne
ttype)
    globals()['DLB_mGCfrac_%s' % abbrevs[i]]=meanGCfrac
    globals()['DLB_mpHR_%s' % abbrevs[i]]=meanPHR
    globals()['DLB_mpSR_%s' % abbrevs[i]]=meanPSR
    globals()['DLB_mpNR_%s' % abbrevs[i]]=meanPNR
    globals()['DLB_pLR_%s' % abbrevs[i]]=percNR
    globals()['DLB_R50H_%s' % abbrevs[i]]=percNR_HR50 #misleading beca
use it is the fraction not percent value
    globals()['DLB_R50S_%s' % abbrevs[i]]=percNR_SR50
    globals()['DLB_R50N_%s' % abbrevs[i]]=percNR_NR50
    globals()['DLB_mR50H_%s' % abbrevs[i]]=meanR50hosts
    globals()['DLB_mR50S_%s' % abbrevs[i]]=meanR50symbs
    globals()['DLB_mR50N_%s' % abbrevs[i]]=meanR50total
    globals()['DLB_std50H_%s' % abbrevs[i]]=stdR50hosts
    globals()['DLB_std50S_%s' % abbrevs[i]]=stdR50symbs
    globals()['DLB_std50N_%s' % abbrevs[i]]=stdR50total

#ALL OF THE RANDOM NODE REMOVALS
model="random"
who="hosts"
```

```
direction=True
#RANDOM HOSTS
for i in xrange(0,length):
    meanGCfrac,meanPHR,meanPSR,meanPNR,percNR,percNR_HR50,percNR_SR50,
percNR_NR50,meanR50hosts,stdR50hosts,meanR50symbs,stdR50symbs, meanR50
total,stdR50total=R50_nodes(network_edges[i], model, who, direction,ne
ttype)
    globals()['RH_mGCfrac_%s' % abbrevs[i]]=meanGCfrac
    globals()['RH_mpHR_%s' % abbrevs[i]]=meanPHR
    globals()['RH_mpSR_%s' % abbrevs[i]]=meanPSR
    globals()['RH_mpNR_%s' % abbrevs[i]]=meanPNR
    globals()['RH_pLR_%s' % abbrevs[i]]=percNR
    globals()['RH_R50H_%s' % abbrevs[i]]=percNR_HR50 #misleading becau
se it is the fraction not percent value
    globals()['RH_R50S_%s' % abbrevs[i]]=percNR_SR50
    globals()['RH_R50N_%s' % abbrevs[i]]=percNR_NR50
    globals()['RH_mR50H_%s' % abbrevs[i]]=meanR50hosts
    globals()['RH_mR50S_%s' % abbrevs[i]]=meanR50symbs
    globals()['RH_mR50N_%s' % abbrevs[i]]=meanR50total
    globals()['RH_std50H_%s' % abbrevs[i]]=stdR50hosts
    globals()['RH_std50S_%s' % abbrevs[i]]=stdR50symbs
    globals()['RH_std50N_%s' % abbrevs[i]]=stdR50total

#RANDOM SYMBIONTS
who="symbionts"
for i in xrange(0,length):
    meanGCfrac,meanPHR,meanPSR,meanPNR,percNR,percNR_HR50,percNR_SR50,
percNR_NR50,meanR50hosts,stdR50hosts,meanR50symbs,stdR50symbs, meanR50
total,stdR50total=R50_nodes(network_edges[i], model, who, direction,ne
ttype)
    globals()['RS_mGCfrac_%s' % abbrevs[i]]=meanGCfrac
    globals()['RS_mpHR_%s' % abbrevs[i]]=meanPHR
    globals()['RS_mpSR_%s' % abbrevs[i]]=meanPSR
    globals()['RS_mpNR_%s' % abbrevs[i]]=meanPNR
    globals()['RS_pLR_%s' % abbrevs[i]]=percNR
    globals()['RS_R50H_%s' % abbrevs[i]]=percNR_HR50 #misleading becau
se it is the fraction not percent value
    globals()['RS_R50S_%s' % abbrevs[i]]=percNR_SR50
    globals()['RS_R50N_%s' % abbrevs[i]]=percNR_NR50
    globals()['RS_mR50H_%s' % abbrevs[i]]=meanR50hosts
    globals()['RS_mR50S_%s' % abbrevs[i]]=meanR50symbs
    globals()['RS_mR50N_%s' % abbrevs[i]]=meanR50total
    globals()['RS_std50H_%s' % abbrevs[i]]=stdR50hosts
    globals()['RS_std50S_%s' % abbrevs[i]]=stdR50symbs
    globals()['RS_std50N_%s' % abbrevs[i]]=stdR50total

#RANDOM BOTH
who="both"
for i in xrange(0,length):
    meanGCfrac,meanPHR,meanPSR,meanPNR,percNR,percNR_HR50,percNR_SR50,
percNR_NR50,meanR50hosts,stdR50hosts,meanR50symbs,stdR50symbs, meanR50
total,stdR50total=R50_nodes(network_edges[i], model, who, direction,ne
ttype)
```

```
    globals()['RB_mGCfrac_%s' % abbrevs[i]]=meanGCfrac
    globals()['RB_mpHR_%s' % abbrevs[i]]=meanPHR
    globals()['RB_mpSR_%s' % abbrevs[i]]=meanPSR
    globals()['RB_mpNR_%s' % abbrevs[i]]=meanPNR
    globals()['RB_pLR_%s' % abbrevs[i]]=percNR
    globals()['RB_R50H_%s' % abbrevs[i]]=percNR_HR50 #misleading becau
se it is the fraction not percent value
    globals()['RB_R50S_%s' % abbrevs[i]]=percNR_SR50
    globals()['RB_R50N_%s' % abbrevs[i]]=percNR_NR50
    globals()['RB_mR50H_%s' % abbrevs[i]]=meanR50hosts
    globals()['RB_mR50S_%s' % abbrevs[i]]=meanR50symbs
    globals()['RB_mR50N_%s' % abbrevs[i]]=meanR50total
    globals()['RB_std50H_%s' % abbrevs[i]]=stdR50hosts
    globals()['RB_std50S_%s' % abbrevs[i]]=stdR50symbs
    globals()['RB_std50N_%s' % abbrevs[i]]=stdR50total


#Tolerance high host
model="tolerance"
who="hosts"
direction=True

for i in xrange(0,length):
    meanGCfrac,meanPHR,meanPSR,meanPNR,percNR,percNR_HR50,percNR_SR50,
percNR_NR50,meanR50hosts,stdR50hosts,meanR50symbs,stdR50symbs, meanR50
total,stdR50total=R50_nodes(network_edges[i], model, who, direction,ne
ttype)
    globals()['THH_mGCfrac_%s' % abbrevs[i]]=meanGCfrac
    globals()['THH_mpHR_%s' % abbrevs[i]]=meanPHR
    globals()['THH_mpSR_%s' % abbrevs[i]]=meanPSR
    globals()['THH_mpNR_%s' % abbrevs[i]]=meanPNR
    globals()['THH_pLR_%s' % abbrevs[i]]=percNR
    globals()['THH_R50H_%s' % abbrevs[i]]=percNR_HR50 #misleading beca
use it is the fraction not percent value
    globals()['THH_R50S_%s' % abbrevs[i]]=percNR_SR50
    globals()['THH_R50N_%s' % abbrevs[i]]=percNR_NR50
    globals()['THH_mR50H_%s' % abbrevs[i]]=meanR50hosts
    globals()['THH_mR50S_%s' % abbrevs[i]]=meanR50symbs
    globals()['THH_mR50N_%s' % abbrevs[i]]=meanR50total
    globals()['THH_std50H_%s' % abbrevs[i]]=stdR50hosts
    globals()['THH_std50S_%s' % abbrevs[i]]=stdR50symbs
    globals()['THH_std50N_%s' % abbrevs[i]]=stdR50total

#tolerance low host
direction=False
for i in xrange(0,length):
    meanGCfrac,meanPHR,meanPSR,meanPNR,percNR,percNR_HR50,percNR_SR50,
percNR_NR50,meanR50hosts,stdR50hosts,meanR50symbs,stdR50symbs, meanR50
total,stdR50total=R50_nodes(network_edges[i], model, who, direction,ne
ttype)
    globals()['TLH_mGCfrac_%s' % abbrevs[i]]=meanGCfrac
    globals()['TLH_mpHR_%s' % abbrevs[i]]=meanPHR
    globals()['TLH_mpSR_%s' % abbrevs[i]]=meanPSR
```

```
    globals()['TLH_mpNR_%s' % abbrevs[i]]=meanPNR
    globals()['TLH_pLR_%s' % abbrevs[i]]=percNR
    globals()['TLH_R50H_%s' % abbrevs[i]]=percNR_HR50 #misleading beca
use it is the fraction not percent value
    globals()['TLH_R50S_%s' % abbrevs[i]]=percNR_SR50
    globals()['TLH_R50N_%s' % abbrevs[i]]=percNR_NR50
    globals()['TLH_mR50H_%s' % abbrevs[i]]=meanR50hosts
    globals()['TLH_mR50S_%s' % abbrevs[i]]=meanR50symbs
    globals()['TLH_mR50N_%s' % abbrevs[i]]=meanR50total
    globals()['TLH_std50H_%s' % abbrevs[i]]=stdR50hosts
    globals()['TLH_std50S_%s' % abbrevs[i]]=stdR50symbs
    globals()['TLH_std50N_%s' % abbrevs[i]]=stdR50total

#tolerance high symbionts
who="symbionts"
direction=True
for i in xrange(0,length):
    meanGCfrac,meanPHR,meanPSR,meanPNR,percNR,percNR_HR50,percNR_SR50,
percNR_NR50,meanR50hosts,stdR50hosts,meanR50symbs,stdR50symbs, meanR50
total,stdR50total=R50_nodes(network_edges[i], model, who, direction,ne
ttype)
    globals()['THS_mGCfrac_%s' % abbrevs[i]]=meanGCfrac
    globals()['THS_mpHR_%s' % abbrevs[i]]=meanPHR
    globals()['THS_mpSR_%s' % abbrevs[i]]=meanPSR
    globals()['THS_mpNR_%s' % abbrevs[i]]=meanPNR
    globals()['THS_pLR_%s' % abbrevs[i]]=percNR
    globals()['THS_R50H_%s' % abbrevs[i]]=percNR_HR50 #misleading beca
use it is the fraction not percent value
    globals()['THS_R50S_%s' % abbrevs[i]]=percNR_SR50
    globals()['THS_R50N_%s' % abbrevs[i]]=percNR_NR50
    globals()['THS_mR50H_%s' % abbrevs[i]]=meanR50hosts
    globals()['THS_mR50S_%s' % abbrevs[i]]=meanR50symbs
    globals()['THS_mR50N_%s' % abbrevs[i]]=meanR50total
    globals()['THS_std50H_%s' % abbrevs[i]]=stdR50hosts
    globals()['THS_std50S_%s' % abbrevs[i]]=stdR50symbs
    globals()['THS_std50N_%s' % abbrevs[i]]=stdR50total

#tolerance low symbionts
direction=False
for i in xrange(0,length):
    meanGCfrac,meanPHR,meanPSR,meanPNR,percNR,percNR_HR50,percNR_SR50,
percNR_NR50,meanR50hosts,stdR50hosts,meanR50symbs,stdR50symbs, meanR50
total,stdR50total=R50_nodes(network_edges[i], model, who, direction,ne
ttype)
    globals()['TLS_mGCfrac_%s' % abbrevs[i]]=meanGCfrac
    globals()['TLS_mpHR_%s' % abbrevs[i]]=meanPHR
    globals()['TLS_mpSR_%s' % abbrevs[i]]=meanPSR
    globals()['TLS_mpNR_%s' % abbrevs[i]]=meanPNR
    globals()['TLS_pLR_%s' % abbrevs[i]]=percNR
    globals()['TLS_R50H_%s' % abbrevs[i]]=percNR_HR50 #misleading beca
use it is the fraction not percent value
    globals()['TLS_R50S_%s' % abbrevs[i]]=percNR_SR50
    globals()['TLS_R50N_%s' % abbrevs[i]]=percNR_NR50
```

```
    globals()['TLS_mR50H_%s' % abbrevs[i]]=meanR50hosts
    globals()['TLS_mR50S_%s' % abbrevs[i]]=meanR50symbs
    globals()['TLS_mR50N_%s' % abbrevs[i]]=meanR50total
    globals()['TLS_std50H_%s' % abbrevs[i]]=stdR50hosts
    globals()['TLS_std50S_%s' % abbrevs[i]]=stdR50symbs
    globals()['TLS_std50N_%s' % abbrevs[i]]=stdR50total

#tolerance high BOTH
who="both"
direction=True
for i in xrange(0,length):
    meanGCfrac,meanPHR,meanPSR,meanPNR,percNR,percNR_HR50,percNR_SR50,
percNR_NR50,meanR50hosts,stdR50hosts,meanR50symbs,stdR50symbs, meanR50
total,stdR50total=R50_nodes(network_edges[i], model, who, direction,ne
ttype)
    globals()['THB_mGCfrac_%s' % abbrevs[i]]=meanGCfrac
    globals()['THB_mpHR_%s' % abbrevs[i]]=meanPHR
    globals()['THB_mpSR_%s' % abbrevs[i]]=meanPSR
    globals()['THB_mpNR_%s' % abbrevs[i]]=meanPNR
    globals()['THB_pLR_%s' % abbrevs[i]]=percNR
    globals()['THB_R50H_%s' % abbrevs[i]]=percNR_HR50 #misleading beca
use it is the fraction not percent value
    globals()['THB_R50S_%s' % abbrevs[i]]=percNR_SR50
    globals()['THB_R50N_%s' % abbrevs[i]]=percNR_NR50
    globals()['THB_mR50H_%s' % abbrevs[i]]=meanR50hosts
    globals()['THB_mR50S_%s' % abbrevs[i]]=meanR50symbs
    globals()['THB_mR50N_%s' % abbrevs[i]]=meanR50total
    globals()['THB_std50H_%s' % abbrevs[i]]=stdR50hosts
    globals()['THB_std50S_%s' % abbrevs[i]]=stdR50symbs
    globals()['THB_std50N_%s' % abbrevs[i]]=stdR50total

#tolerance low BOTH
direction=False
for i in xrange(0,length):
    meanGCfrac,meanPHR,meanPSR,meanPNR,percNR,percNR_HR50,percNR_SR50,
percNR_NR50,meanR50hosts,stdR50hosts,meanR50symbs,stdR50symbs, meanR50
total,stdR50total=R50_nodes(network_edges[i], model, who, direction,ne
ttype)
    globals()['TLB_mGCfrac_%s' % abbrevs[i]]=meanGCfrac
    globals()['TLB_mpHR_%s' % abbrevs[i]]=meanPHR
    globals()['TLB_mpSR_%s' % abbrevs[i]]=meanPSR
    globals()['TLB_mpNR_%s' % abbrevs[i]]=meanPNR
    globals()['TLB_pLR_%s' % abbrevs[i]]=percNR
    globals()['TLB_R50H_%s' % abbrevs[i]]=percNR_HR50 #misleading beca
use it is the fraction not percent value
    globals()['TLB_R50S_%s' % abbrevs[i]]=percNR_SR50
    globals()['TLB_R50N_%s' % abbrevs[i]]=percNR_NR50
    globals()['TLB_mR50H_%s' % abbrevs[i]]=meanR50hosts
    globals()['TLB_mR50S_%s' % abbrevs[i]]=meanR50symbs
    globals()['TLB_mR50N_%s' % abbrevs[i]]=meanR50total
    globals()['TLB_std50H_%s' % abbrevs[i]]=stdR50hosts
    globals()['TLB_std50S_%s' % abbrevs[i]]=stdR50symbs
    globals()['TLB_std50N_%s' % abbrevs[i]]=stdR50total
```

Start saving things, with the original nets first, and just the means and stdevs

In [23]:

```
#degree high host
#save the means and stds
meanR50H_all=[]
stdR50H_all=[]
meanR50S_all=[]
stdR50S_all=[]
meanR50N_all=[]
stdR50N_all=[]

for i in xrange(0,14):
    meanR50H_all.append(globals()['DHH_mR50H_%s' % abbrevs[i]])
    meanR50S_all.append(globals()['DHH_mR50S_%s' % abbrevs[i]])
    stdR50H_all.append(globals()['DHH_std50H_%s' % abbrevs[i]])
    stdR50S_all.append(globals()['DHH_std50S_%s' % abbrevs[i]])
    meanR50N_all.append(globals()['DHH_mR50N_%s' % abbrevs[i]])
    stdR50N_all.append(globals()['DHH_std50N_%s' % abbrevs[i]])


meanR50H_all=np.array(meanR50H_all)
stdR50H_all=np.array(stdR50H_all)
meanR50S_all=np.array(meanR50S_all)
stdR50S_all=np.array(stdR50S_all)
meanR50N_all=np.array(meanR50N_all)
stdR50N_all=np.array(stdR50N_all)

a=np.column_stack([abbrevs,meanR50H_all,stdR50H_all,meanR50S_all,stdR50S_all,meanR50N_all,stdR50N_all])

df = pd.DataFrame(a)
df.columns=["spatial","meanR50H","stdR50H","meanR50S","stdR50S","meanR50N","stdR50N"]





#degree LOW host
#save the means and stds
meanR50H_all=[]
stdR50H_all=[]
meanR50S_all=[]
stdR50S_all=[]
meanR50N_all=[]
stdR50N_all=[]

for i in xrange(0,14):
    meanR50H_all.append(globals()['DLH_mR50H_%s' % abbrevs[i]])
    meanR50S_all.append(globals()['DLH_mR50S_%s' % abbrevs[i]])
```

```
    stdR50H_all.append(globals()['DLH_std50H_%s' % abbrevs[i]])
    stdR50S_all.append(globals()['DLH_std50S_%s' % abbrevs[i]])
    meanR50N_all.append(globals()['DLH_mR50N_%s' % abbrevs[i]])
    stdR50N_all.append(globals()['DLH_std50N_%s' % abbrevs[i]])


meanR50H_all=np.array(meanR50H_all)
stdR50H_all=np.array(stdR50H_all)
meanR50S_all=np.array(meanR50S_all)
stdR50S_all=np.array(stdR50S_all)
meanR50N_all=np.array(meanR50N_all)
stdR50N_all=np.array(stdR50N_all)

a=np.column_stack([abbrevs,meanR50H_all,stdR50H_all,meanR50S_all,stdR5
0S_all,meanR50N_all,stdR50N_all])

df = pd.DataFrame(a)
df.columns=["spatial","meanR50H","stdR50H","meanR50S","stdR50S","meanR
50N","stdR50N"]




#degree high SYMBIONT
#save the means and stds
meanR50H_all=[]
stdR50H_all=[]
meanR50S_all=[]
stdR50S_all=[]
meanR50N_all=[]
stdR50N_all=[]

for i in xrange(0,14):
    meanR50H_all.append(globals()['DHS_mR50H_%s' % abbrevs[i]])
    meanR50S_all.append(globals()['DHS_mR50S_%s' % abbrevs[i]])
    stdR50H_all.append(globals()['DHS_std50H_%s' % abbrevs[i]])
    stdR50S_all.append(globals()['DHS_std50S_%s' % abbrevs[i]])
    meanR50N_all.append(globals()['DHS_mR50N_%s' % abbrevs[i]])
    stdR50N_all.append(globals()['DHS_std50N_%s' % abbrevs[i]])


meanR50H_all=np.array(meanR50H_all)
stdR50H_all=np.array(stdR50H_all)
meanR50S_all=np.array(meanR50S_all)
stdR50S_all=np.array(stdR50S_all)
meanR50N_all=np.array(meanR50N_all)
stdR50N_all=np.array(stdR50N_all)

a=np.column_stack([abbrevs,meanR50H_all,stdR50H_all,meanR50S_all,stdR5
0S_all,meanR50N_all,stdR50N_all])

df = pd.DataFrame(a)
df.columns=["spatial","meanR50H","stdR50H","meanR50S","stdR50S","meanR
```

```
50N","stdR50N"]




#degree LOW SYMBIONT
#save the means and stds
meanR50H_all=[]
stdR50H_all=[]
meanR50S_all=[]
stdR50S_all=[]
meanR50N_all=[]
stdR50N_all=[]

for i in xrange(0,14):
    meanR50H_all.append(globals()['DLS_mR50H_%s' % abbrevs[i]])
    meanR50S_all.append(globals()['DLS_mR50S_%s' % abbrevs[i]])
    stdR50H_all.append(globals()['DLS_std50H_%s' % abbrevs[i]])
    stdR50S_all.append(globals()['DLS_std50S_%s' % abbrevs[i]])
    meanR50N_all.append(globals()['DLS_mR50N_%s' % abbrevs[i]])
    stdR50N_all.append(globals()['DLS_std50N_%s' % abbrevs[i]])


meanR50H_all=np.array(meanR50H_all)
stdR50H_all=np.array(stdR50H_all)
meanR50S_all=np.array(meanR50S_all)
stdR50S_all=np.array(stdR50S_all)
meanR50N_all=np.array(meanR50N_all)
stdR50N_all=np.array(stdR50N_all)

a=np.column_stack([abbrevs,meanR50H_all,stdR50H_all,meanR50S_all,stdR5
0S_all,meanR50N_all,stdR50N_all])

df = pd.DataFrame(a)
df.columns=["spatial","meanR50H","stdR50H","meanR50S","stdR50S","meanR
50N","stdR50N"]




#degree high BOTH
#save the means and stds
meanR50H_all=[]
stdR50H_all=[]
meanR50S_all=[]
stdR50S_all=[]
meanR50N_all=[]
stdR50N_all=[]

for i in xrange(0,14):
    meanR50H_all.append(globals()['DHB_mR50H_%s' % abbrevs[i]])
    meanR50S_all.append(globals()['DHB_mR50S_%s' % abbrevs[i]])
    stdR50H_all.append(globals()['DHB_std50H_%s' % abbrevs[i]])
```

```
    stdR50S_all.append(globals()['DHB_std50S_%s' % abbrevs[i]])
    meanR50N_all.append(globals()['DHB_mR50N_%s' % abbrevs[i]])
    stdR50N_all.append(globals()['DHB_std50N_%s' % abbrevs[i]])


meanR50H_all=np.array(meanR50H_all)
stdR50H_all=np.array(stdR50H_all)
meanR50S_all=np.array(meanR50S_all)
stdR50S_all=np.array(stdR50S_all)
meanR50N_all=np.array(meanR50N_all)
stdR50N_all=np.array(stdR50N_all)

a=np.column_stack([abbrevs,meanR50H_all,stdR50H_all,meanR50S_all,stdR5
0S_all,meanR50N_all,stdR50N_all])

df = pd.DataFrame(a)
df.columns=["spatial","meanR50H","stdR50H","meanR50S","stdR50S","meanR
50N","stdR50N"]



#degree LOW BOTH
#save the means and stds
meanR50H_all=[]
stdR50H_all=[]
meanR50S_all=[]
stdR50S_all=[]
meanR50N_all=[]
stdR50N_all=[]

for i in xrange(0,14):
    meanR50H_all.append(globals()['DLB_mR50H_%s' % abbrevs[i]])
    meanR50S_all.append(globals()['DLB_mR50S_%s' % abbrevs[i]])
    stdR50H_all.append(globals()['DLB_std50H_%s' % abbrevs[i]])
    stdR50S_all.append(globals()['DLB_std50S_%s' % abbrevs[i]])
    meanR50N_all.append(globals()['DLB_mR50N_%s' % abbrevs[i]])
    stdR50N_all.append(globals()['DLB_std50N_%s' % abbrevs[i]])


meanR50H_all=np.array(meanR50H_all)
stdR50H_all=np.array(stdR50H_all)
meanR50S_all=np.array(meanR50S_all)
stdR50S_all=np.array(stdR50S_all)
meanR50N_all=np.array(meanR50N_all)
stdR50N_all=np.array(stdR50N_all)

a=np.column_stack([abbrevs,meanR50H_all,stdR50H_all,meanR50S_all,stdR5
0S_all,meanR50N_all,stdR50N_all])

df = pd.DataFrame(a)
df.columns=["spatial","meanR50H","stdR50H","meanR50S","stdR50S","meanR
50N","stdR50N"]
```

```
#tolerance high host
#save the means and stds
meanR50H_all=[]
stdR50H_all=[]
meanR50S_all=[]
stdR50S_all=[]
meanR50N_all=[]
stdR50N_all=[]

for i in xrange(0,14):
    meanR50H_all.append(globals()['THH_mR50H_%s' % abbrevs[i]])
    meanR50S_all.append(globals()['THH_mR50S_%s' % abbrevs[i]])
    stdR50H_all.append(globals()['THH_std50H_%s' % abbrevs[i]])
    stdR50S_all.append(globals()['THH_std50S_%s' % abbrevs[i]])
    meanR50N_all.append(globals()['THH_mR50N_%s' % abbrevs[i]])
    stdR50N_all.append(globals()['THH_std50N_%s' % abbrevs[i]])


meanR50H_all=np.array(meanR50H_all)
stdR50H_all=np.array(stdR50H_all)
meanR50S_all=np.array(meanR50S_all)
stdR50S_all=np.array(stdR50S_all)
meanR50N_all=np.array(meanR50N_all)
stdR50N_all=np.array(stdR50N_all)

a=np.column_stack([abbrevs,meanR50H_all,stdR50H_all,meanR50S_all,stdR5
0S_all,meanR50N_all,stdR50N_all])

df = pd.DataFrame(a)
df.columns=["spatial","meanR50H","stdR50H","meanR50S","stdR50S","meanR
50N","stdR50N"]





#TOLERANCE LOW host
#save the means and stds
meanR50H_all=[]
stdR50H_all=[]
meanR50S_all=[]
stdR50S_all=[]
meanR50N_all=[]
stdR50N_all=[]

for i in xrange(0,14):
    meanR50H_all.append(globals()['TLH_mR50H_%s' % abbrevs[i]])
    meanR50S_all.append(globals()['TLH_mR50S_%s' % abbrevs[i]])
```

```
    stdR50H_all.append(globals()['TLH_std50H_%s' % abbrevs[i]])
    stdR50S_all.append(globals()['TLH_std50S_%s' % abbrevs[i]])
    meanR50N_all.append(globals()['TLH_mR50N_%s' % abbrevs[i]])
    stdR50N_all.append(globals()['TLH_std50N_%s' % abbrevs[i]])


meanR50H_all=np.array(meanR50H_all)
stdR50H_all=np.array(stdR50H_all)
meanR50S_all=np.array(meanR50S_all)
stdR50S_all=np.array(stdR50S_all)
meanR50N_all=np.array(meanR50N_all)
stdR50N_all=np.array(stdR50N_all)

a=np.column_stack([abbrevs,meanR50H_all,stdR50H_all,meanR50S_all,stdR5
0S_all,meanR50N_all,stdR50N_all])

df = pd.DataFrame(a)
df.columns=["spatial","meanR50H","stdR50H","meanR50S","stdR50S","meanR
50N","stdR50N"]




#TOLERANCE high SYMBIONT
#save the means and stds
meanR50H_all=[]
stdR50H_all=[]
meanR50S_all=[]
stdR50S_all=[]
meanR50N_all=[]
stdR50N_all=[]

for i in xrange(0,14):
    meanR50H_all.append(globals()['THS_mR50H_%s' % abbrevs[i]])
    meanR50S_all.append(globals()['THS_mR50S_%s' % abbrevs[i]])
    stdR50H_all.append(globals()['THS_std50H_%s' % abbrevs[i]])
    stdR50S_all.append(globals()['THS_std50S_%s' % abbrevs[i]])
    meanR50N_all.append(globals()['THS_mR50N_%s' % abbrevs[i]])
    stdR50N_all.append(globals()['THS_std50N_%s' % abbrevs[i]])


meanR50H_all=np.array(meanR50H_all)
stdR50H_all=np.array(stdR50H_all)
meanR50S_all=np.array(meanR50S_all)
stdR50S_all=np.array(stdR50S_all)
meanR50N_all=np.array(meanR50N_all)
stdR50N_all=np.array(stdR50N_all)

a=np.column_stack([abbrevs,meanR50H_all,stdR50H_all,meanR50S_all,stdR5
0S_all,meanR50N_all,stdR50N_all])

df = pd.DataFrame(a)
```

```
df.columns=["spatial","meanR50H","stdR50H","meanR50S","stdR50S","meanR
50N","stdR50N"]





#TOLERANCE LOW SYMBIONT
#save the means and stds
meanR50H_all=[]
stdR50H_all=[]
meanR50S_all=[]
stdR50S_all=[]
meanR50N_all=[]
stdR50N_all=[]

for i in xrange(0,14):
    meanR50H_all.append(globals()['TLS_mR50H_%s' % abbrevs[i]])
    meanR50S_all.append(globals()['TLS_mR50S_%s' % abbrevs[i]])
    stdR50H_all.append(globals()['TLS_std50H_%s' % abbrevs[i]])
    stdR50S_all.append(globals()['TLS_std50S_%s' % abbrevs[i]])
    meanR50N_all.append(globals()['TLS_mR50N_%s' % abbrevs[i]])
    stdR50N_all.append(globals()['TLS_std50N_%s' % abbrevs[i]])


meanR50H_all=np.array(meanR50H_all)
stdR50H_all=np.array(stdR50H_all)
meanR50S_all=np.array(meanR50S_all)
stdR50S_all=np.array(stdR50S_all)
meanR50N_all=np.array(meanR50N_all)
stdR50N_all=np.array(stdR50N_all)

a=np.column_stack([abbrevs,meanR50H_all,stdR50H_all,meanR50S_all,stdR5
0S_all,meanR50N_all,stdR50N_all])

df = pd.DataFrame(a)
df.columns=["spatial","meanR50H","stdR50H","meanR50S","stdR50S","meanR
50N","stdR50N"]




#TOLERANCE high BOTH
#save the means and stds
meanR50H_all=[]
stdR50H_all=[]
meanR50S_all=[]
stdR50S_all=[]
meanR50N_all=[]
stdR50N_all=[]

for i in xrange(0,14):
```

```
    meanR50H_all.append(globals()['THB_mR50H_%s' % abbrevs[i]])
    meanR50S_all.append(globals()['THB_mR50S_%s' % abbrevs[i]])
    stdR50H_all.append(globals()['THB_std50H_%s' % abbrevs[i]])
    stdR50S_all.append(globals()['THB_std50S_%s' % abbrevs[i]])
    meanR50N_all.append(globals()['THB_mR50N_%s' % abbrevs[i]])
    stdR50N_all.append(globals()['THB_std50N_%s' % abbrevs[i]])


meanR50H_all=np.array(meanR50H_all)
stdR50H_all=np.array(stdR50H_all)
meanR50S_all=np.array(meanR50S_all)
stdR50S_all=np.array(stdR50S_all)
meanR50N_all=np.array(meanR50N_all)
stdR50N_all=np.array(stdR50N_all)

a=np.column_stack([abbrevs,meanR50H_all,stdR50H_all,meanR50S_all,stdR5
0S_all,meanR50N_all,stdR50N_all])

df = pd.DataFrame(a)
df.columns=["spatial","meanR50H","stdR50H","meanR50S","stdR50S","meanR
50N","stdR50N"]





#TOLERANCE LOW BOTH
#save the means and stds
meanR50H_all=[]
stdR50H_all=[]
meanR50S_all=[]
stdR50S_all=[]
meanR50N_all=[]
stdR50N_all=[]

for i in xrange(0,14):
    meanR50H_all.append(globals()['TLB_mR50H_%s' % abbrevs[i]])
    meanR50S_all.append(globals()['TLB_mR50S_%s' % abbrevs[i]])
    stdR50H_all.append(globals()['TLB_std50H_%s' % abbrevs[i]])
    stdR50S_all.append(globals()['TLB_std50S_%s' % abbrevs[i]])
    meanR50N_all.append(globals()['TLB_mR50N_%s' % abbrevs[i]])
    stdR50N_all.append(globals()['TLB_std50N_%s' % abbrevs[i]])


meanR50H_all=np.array(meanR50H_all)
stdR50H_all=np.array(stdR50H_all)
meanR50S_all=np.array(meanR50S_all)
stdR50S_all=np.array(stdR50S_all)
meanR50N_all=np.array(meanR50N_all)
stdR50N_all=np.array(stdR50N_all)

a=np.column_stack([abbrevs,meanR50H_all,stdR50H_all,meanR50S_all,stdR5
0S_all,meanR50N_all,stdR50N_all])
```

```
df = pd.DataFrame(a)
df.columns=["spatial","meanR50H","stdR50H","meanR50S","stdR50S","meanR
50N","stdR50N"]




#random host
#save the means and stds
meanR50H_all=[]
stdR50H_all=[]
meanR50S_all=[]
stdR50S_all=[]
meanR50N_all=[]
stdR50N_all=[]

for i in xrange(0,14):
    meanR50H_all.append(globals()['RH_mR50H_%s' % abbrevs[i]])
    meanR50S_all.append(globals()['RH_mR50S_%s' % abbrevs[i]])
    stdR50H_all.append(globals()['RH_std50H_%s' % abbrevs[i]])
    stdR50S_all.append(globals()['RH_std50S_%s' % abbrevs[i]])
    meanR50N_all.append(globals()['RH_mR50N_%s' % abbrevs[i]])
    stdR50N_all.append(globals()['RH_std50N_%s' % abbrevs[i]])


meanR50H_all=np.array(meanR50H_all)
stdR50H_all=np.array(stdR50H_all)
meanR50S_all=np.array(meanR50S_all)
stdR50S_all=np.array(stdR50S_all)
meanR50N_all=np.array(meanR50N_all)
stdR50N_all=np.array(stdR50N_all)

a=np.column_stack([abbrevs,meanR50H_all,stdR50H_all,meanR50S_all,stdR5
0S_all,meanR50N_all,stdR50N_all])

df = pd.DataFrame(a)
df.columns=["spatial","meanR50H","stdR50H","meanR50S","stdR50S","meanR
50N","stdR50N"]





#RANDOM SYMBIONT
#save the means and stds
meanR50H_all=[]
stdR50H_all=[]
meanR50S_all=[]
stdR50S_all=[]
meanR50N_all=[]
stdR50N_all=[]
```

```
for i in xrange(0,14):
    meanR50H_all.append(globals()['RS_mR50H_%s' % abbrevs[i]])
    meanR50S_all.append(globals()['RS_mR50S_%s' % abbrevs[i]])
    stdR50H_all.append(globals()['RS_std50H_%s' % abbrevs[i]])
    stdR50S_all.append(globals()['RS_std50S_%s' % abbrevs[i]])
    meanR50N_all.append(globals()['RS_mR50N_%s' % abbrevs[i]])
    stdR50N_all.append(globals()['RS_std50N_%s' % abbrevs[i]])


meanR50H_all=np.array(meanR50H_all)
stdR50H_all=np.array(stdR50H_all)
meanR50S_all=np.array(meanR50S_all)
stdR50S_all=np.array(stdR50S_all)
meanR50N_all=np.array(meanR50N_all)
stdR50N_all=np.array(stdR50N_all)

a=np.column_stack([abbrevs,meanR50H_all,stdR50H_all,meanR50S_all,stdR5
0S_all,meanR50N_all,stdR50N_all])

df = pd.DataFrame(a)
df.columns=["spatial","meanR50H","stdR50H","meanR50S","stdR50S","meanR
50N","stdR50N"]





#RANDOM BOTH
#save the means and stds
meanR50H_all=[]
stdR50H_all=[]
meanR50S_all=[]
stdR50S_all=[]
meanR50N_all=[]
stdR50N_all=[]

for i in xrange(0,14):
    meanR50H_all.append(globals()['RB_mR50H_%s' % abbrevs[i]])
    meanR50S_all.append(globals()['RB_mR50S_%s' % abbrevs[i]])
    stdR50H_all.append(globals()['RB_std50H_%s' % abbrevs[i]])
    stdR50S_all.append(globals()['RB_std50S_%s' % abbrevs[i]])
    meanR50N_all.append(globals()['RB_mR50N_%s' % abbrevs[i]])
    stdR50N_all.append(globals()['RB_std50N_%s' % abbrevs[i]])


meanR50H_all=np.array(meanR50H_all)
stdR50H_all=np.array(stdR50H_all)
meanR50S_all=np.array(meanR50S_all)
stdR50S_all=np.array(stdR50S_all)
meanR50N_all=np.array(meanR50N_all)
stdR50N_all=np.array(stdR50N_all)
```

```
a=np.column_stack([abbrevs,meanR50H_all,stdR50H_all,meanR50S_all,stdR5
0S_all,meanR50N_all,stdR50N_all])

df = pd.DataFrame(a)
df.columns=["spatial","meanR50H","stdR50H","meanR50S","stdR50S","meanR
50N","stdR50N"]
```

Save the simulations of r50s

In [271]:

```
#save for the total robustness scenario
for i in xrange(0,14):
    bleach=globals()['bleach_R50N_%s' % abbrevs[i]]
    bleach = [x/100 for x in bleach]
    RL=globals()['RL_R50N_%s' % abbrevs[i]]
    LT_BL=globals()['LT_BL_R50N_%s' % abbrevs[i]]
    LT_SL=globals()['LT_SL_R50N_%s' % abbrevs[i]]
    LT_HL=globals()['LT_HL_R50N_%s' % abbrevs[i]]
    RB=globals()['RB_R50N_%s' % abbrevs[i]]
    TLB=globals()['TLB_R50N_%s' % abbrevs[i]]
    DHB=globals()['DHB_R50N_%s' % abbrevs[i]]
    DLB=globals()['DLB_R50N_%s' % abbrevs[i]]
    a=np.column_stack([bleach,RL,LT_BL,LT_SL,LT_HL,RB,TLB,DHB,DLB])
    df = pd.DataFrame(a)
    df.columns=["bleach","RL","LT_BL","LT_SL","LT_HL","RB","TLB","DHB"
,"DLB"]
    globals()['totrob_%s' % abbrevs[i]]=df
#Use totrob_'abbreviation'.to_csv("path") to save
```

In [273]:

```
#save for the host robustness scenario
for i in xrange(0,14):
    bleach=globals()['bleach_R50H_%s' % abbrevs[i]]
    bleach = [x/100 for x in bleach]
    RL=globals()['RL_R50H_%s' % abbrevs[i]]
    LT_BL=globals()['LT_BL_R50H_%s' % abbrevs[i]]
    LT_SL=globals()['LT_SL_R50H_%s' % abbrevs[i]]
    LT_HL=globals()['LT_HL_R50H_%s' % abbrevs[i]]
    RS=globals()['RS_R50H_%s' % abbrevs[i]]
    TLS=globals()['TLS_R50H_%s' % abbrevs[i]]
    DHS=globals()['DHS_R50H_%s' % abbrevs[i]]
    DLS=globals()['DLS_R50H_%s' % abbrevs[i]]
    a=np.column_stack([bleach,RL,LT_BL,LT_SL,LT_HL,RS,TLS,DHS,DLS])
    df = pd.DataFrame(a)
    df.columns=["bleach","RL","LT_BL","LT_SL","LT_HL","RS","TLS","DHS"
,"DLS"]
    globals()['hostrob_%s' % abbrevs[i]]=df
```

In [274]:
```
#save for the symbiont robustness scenario
for i in xrange(0,14):
    bleach=globals()['bleach_R50S_%s' % abbrevs[i]]
    bleach = [x/100 for x in bleach]
    RL=globals()['RL_R50S_%s' % abbrevs[i]]
    LT_BL=globals()['LT_BL_R50S_%s' % abbrevs[i]]
    LT_SL=globals()['LT_SL_R50S_%s' % abbrevs[i]]
    LT_HL=globals()['LT_HL_R50S_%s' % abbrevs[i]]
    RH=globals()['RH_R50S_%s' % abbrevs[i]]
    TLH=globals()['TLH_R50S_%s' % abbrevs[i]]
    DHH=globals()['DHH_R50S_%s' % abbrevs[i]]
    DLH=globals()['DLH_R50S_%s' % abbrevs[i]]
    a=np.column_stack([bleach,RL,LT_BL,LT_SL,LT_HL,RH,TLH,DHH,DLH])
    df = pd.DataFrame(a)
    df.columns=["bleach","RL","LT_BL","LT_SL","LT_HL","RH","TLH","DHH"
,"DLH"]
    globals()['symbrob_%s' % abbrevs[i]]=df
```

```
In [275]: #actually, lets put it all in one file because it will be easier to wo
rk with in R
for i in xrange(0,14):
    T_bleach=globals()['bleach_R50N_%s' % abbrevs[i]]
    T_bleach = [x/100 for x in bleach]
    T_RL=globals()['RL_R50N_%s' % abbrevs[i]]
    T_LT_BL=globals()['LT_BL_R50N_%s' % abbrevs[i]]
    T_LT_SL=globals()['LT_SL_R50N_%s' % abbrevs[i]]
    T_LT_HL=globals()['LT_HL_R50N_%s' % abbrevs[i]]
    T_RB=globals()['RB_R50N_%s' % abbrevs[i]]
    T_TLB=globals()['TLB_R50N_%s' % abbrevs[i]]
    T_DHB=globals()['DHB_R50N_%s' % abbrevs[i]]
    T_DLB=globals()['DLB_R50N_%s' % abbrevs[i]]

    H_bleach=globals()['bleach_R50H_%s' % abbrevs[i]]
    H_bleach = [x/100 for x in bleach]
    H_RL=globals()['RL_R50H_%s' % abbrevs[i]]
    H_LT_BL=globals()['LT_BL_R50H_%s' % abbrevs[i]]
    H_LT_SL=globals()['LT_SL_R50H_%s' % abbrevs[i]]
    H_LT_HL=globals()['LT_HL_R50H_%s' % abbrevs[i]]
    H_RS=globals()['RS_R50H_%s' % abbrevs[i]]
    H_TLS=globals()['TLS_R50H_%s' % abbrevs[i]]
    H_DHS=globals()['DHS_R50H_%s' % abbrevs[i]]
    H_DLS=globals()['DLS_R50H_%s' % abbrevs[i]]

    S_bleach=globals()['bleach_R50S_%s' % abbrevs[i]]
    S_bleach = [x/100 for x in bleach]
    S_RL=globals()['RL_R50S_%s' % abbrevs[i]]
    S_LT_BL=globals()['LT_BL_R50S_%s' % abbrevs[i]]
    S_LT_SL=globals()['LT_SL_R50S_%s' % abbrevs[i]]
    S_LT_HL=globals()['LT_HL_R50S_%s' % abbrevs[i]]
    S_RH=globals()['RH_R50S_%s' % abbrevs[i]]
    S_TLH=globals()['TLH_R50S_%s' % abbrevs[i]]
    S_DHH=globals()['DHH_R50S_%s' % abbrevs[i]]
    S_DLH=globals()['DLH_R50S_%s' % abbrevs[i]]


    a=np.column_stack([T_bleach,T_RL,T_LT_BL,T_LT_SL,T_LT_HL,T_RB,T_TL
B,T_DHB,T_DLB,S_bleach,S_RL,S_LT_BL,S_LT_SL,S_LT_HL,S_RH,S_TLH,S_DHH,S
_DLH,H_bleach,H_RL,H_LT_BL,H_LT_SL,H_LT_HL,H_RS,H_TLS,H_DHS,H_DLS])
    df = pd.DataFrame(a)
    df.columns=["T_bleach","T_RL","T_LT_BL","T_LT_SL","T_LT_HL","T_RB"
,"T_TLB","T_DHB","T_DLB","S_bleach","S_RL","S_LT_BL","S_LT_SL","S_LT_H
L","S_RH","S_TLH","S_DHH","S_DLH","H_bleach","H_RL","H_LT_BL","H_LT_SL
","H_LT_HL","H_RS","H_TLS","H_DHS","H_DLS"]
    globals()['rob_%s' % abbrevs[i]]=df
```

Ok, now lets plot the Robustness Curves for the Global Network

In [25]:
```
fig,((ax1,ax2,ax3))=plt.subplots(nrows=1,ncols=3,sharex='col',figsize=
(19,7))

#total robustness



ax1.plot(RL_pLR_G,RL_mpNR_G,label='Random Links',color='black',linesty
le='-',linewidth=2)
ax1.plot(bleach_pLR_G[:,1],bleach_mpNR_G,label='Bleaching',color='#1f7
8b4',linestyle='-',linewidth=2)

ax1.plot(LT_BL_pLR_G,LT_BL_mpNR_G,label='Susceptible Link',color='#33a
02c',linestyle='-',linewidth=2)
ax1.plot(LT_HL_pLR_G,LT_HL_mpNR_G,label='Susceptible Host Link',color=
'#e31a1c',linestyle='-',linewidth=2)
ax1.plot(LT_SL_pLR_G,LT_SL_mpNR_G,label='Susceptible Symbiont Link',co
lor='#ff7f00',linestyle='-',linewidth=2)

ax1.plot(RB_pLR_G,RB_mpNR_G,label='Random Nodes',color="darkgrey",line
style='--',linewidth=3)

ax1.plot(DLB_pLR_G,DLB_mpNR_G,label='Low Degree',color="#a6cee3",lines
tyle='--',linewidth=3)

ax1.plot(DHB_pLR_G,DHB_mpNR_G,label='High Degree',color="#cab2d6",line
style='--',linewidth=3)

ax1.plot(TLB_pLR_G,TLB_mpNR_G,label='Susceptible',color="#6a3d9a",line
style='--',linewidth=3)
ax1.set_ylim(0,100)
ax1.set_xlim(0,100)

ax1.set_title("Total Robustness",size=15)
#host robustness

ax2.plot(RL_pLR_G,RL_mpHR_G,label='Random Links',color='black',linesty
le='-',linewidth=2)
ax2.plot(bleach_pLR_G[:,1],bleach_mpHR_G,label='Bleaching',color='#1f7
8b4',linestyle='-',linewidth=2)
ax2.plot(LT_BL_pLR_G,LT_BL_mpHR_G,label='Susceptible Link',color='#33a
02c',linestyle='-',linewidth=2)
ax2.plot(LT_HL_pLR_G,LT_HL_mpHR_G,label='Susceptible Host Link',color=
'#e31a1c',linestyle='-',linewidth=2)
ax2.plot(LT_SL_pLR_G,LT_SL_mpHR_G,label='Susceptible Symbiont Link',co
lor='#ff7f00',linestyle='-',linewidth=2)
ax2.plot(RS_pLR_G,RS_mpHR_G,label='Random Nodes',color="darkgrey",line
style='--',linewidth=3)
ax2.plot(DLS_pLR_G,DLS_mpHR_G,label='Low Degree',color="#a6cee3",lines
tyle='--',linewidth=3)
ax2.plot(DHS_pLR_G,DHS_mpHR_G,label='High Degree',color="#cab2d6",line
style='--',linewidth=3)
ax2.plot(TLS_pLR_G,TLS_mpHR_G,label='Susceptible',color="#6a3d9a",line
```

```
style='--',linewidth=3)
ax2.set_title("Host Robustness",size=15)
ax2.set_ylim(0,100)
ax2.set_xlim(0,100)
#symbiont robustness

ax3.plot(RL_pLR_G,RL_mpSR_G,label='Random Links',color='black',linesty
le='-',linewidth=2)
ax3.plot(bleach_pLR_G[:,1],bleach_mpSR_G,label='Bleaching',color='#1f7
8b4',linestyle='-',linewidth=2)
ax3.plot(LT_BL_pLR_G,LT_BL_mpSR_G,label='Susceptible Link',color='#33a
02c',linestyle='-',linewidth=2)
ax3.plot(LT_HL_pLR_G,LT_HL_mpSR_G,label='Susceptible Host Link',color=
'#e31a1c',linestyle='-',linewidth=2)
ax3.plot(LT_SL_pLR_G,LT_SL_mpSR_G,label='Susceptible Symbiont Link',co
lor='#ff7f00',linestyle='-',linewidth=2)
ax3.plot(RH_pLR_G,RH_mpSR_G,label='Random Nodes',color="darkgrey",line
style='--',linewidth=3)
ax3.plot(DLH_pLR_G,DLH_mpSR_G,label='Low Degree',color="#a6cee3",lines
tyle='--',linewidth=3)
ax3.plot(DHH_pLR_G,DHH_mpSR_G,label='High Degree',color="#cab2d6",line
style='--',linewidth=3)
ax3.plot(TLH_pLR_G,TLH_mpSR_G,label='Susceptible',color="#6a3d9a",line
style='--',linewidth=3)
ax3.set_title("Symbiont Robustness",size=15)
ax3.set_ylim(0,100)
ax3.set_xlim(0,100)

fig.text(0.5, 0.01, 'Percent Removed', ha='center',size=15)
fig.text(0.08, 0.5, 'Percent Remaining', va='center', rotation='vertic
al',size=15)

legend=plt.legend( loc = 'upper center', bbox_to_anchor = (0.5, 1.1),n
col=5,
            bbox_transform = plt.gcf().transFigure ,fontsize=15)
#legend.get_frame().set_facecolor('#cccccc')
#ax.legend(loc='upper center', bbox_to_anchor=(0.5, -0.05),  shadow=Tr
ue, ncol=2)
#plt.tight_layout
#fig.savefig("/Users/saradellwilliams/Dropbox/Williams_Suppmat_obj2/Gl
obalNetworkRobustness_051419.png",transparent=False,bbox_inches='tight
')

#so links are green
#hosts are blue
#symbionts are yellow

#543005
#8c510a
#bf812d
#dfc27d
#f6e8c3
#c7eae5
```

```
#80cdc1
#35978f
#01665e
#003c30
```

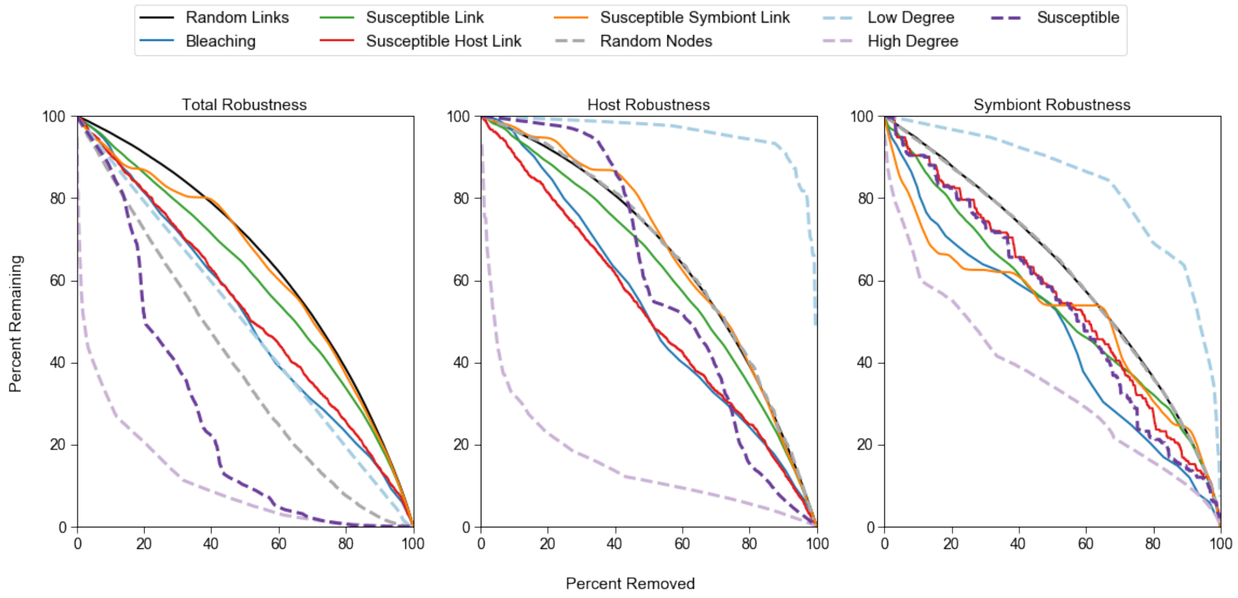

In [39]:
```
fig,((ax1,ax2,ax3,ax4))=plt.subplots(nrows=1,ncols=4,sharex='col',figsize=(15,5))

#global robustness



ax1.plot(RL_pLR_G,RL_mpNR_G,label='Random Links',color='black',linestyle='-',linewidth=2)
ax1.plot(bleach_pLR_G[:,1],bleach_mpNR_G,label='Bleaching',color='#1f78b4',linestyle='-',linewidth=2)

ax1.plot(LT_BL_pLR_G,LT_BL_mpNR_G,label='Susceptible Link',color='#33a02c',linestyle='-',linewidth=2)
ax1.plot(LT_HL_pLR_G,LT_HL_mpNR_G,label='Susceptible Host Link',color='#e31a1c',linestyle='-',linewidth=2)
ax1.plot(LT_SL_pLR_G,LT_SL_mpNR_G,label='Susceptible Symbiont Link',color='#ff7f00',linestyle='-',linewidth=2)

ax1.plot(RB_pLR_G,RB_mpNR_G,label='Random Nodes',color="darkgrey",linestyle='--',linewidth=3)

ax1.plot(DLB_pLR_G,DLB_mpNR_G,label='Low Degree',color="#a6cee3",linestyle='--',linewidth=3)

ax1.plot(DHB_pLR_G,DHB_mpNR_G,label='High Degree',color="#cab2d6",linestyle='--',linewidth=3)

ax1.plot(TLB_pLR_G,TLB_mpNR_G,label='Susceptible',color="#6a3d9a",linestyle='--',linewidth=3)
ax1.set_ylim(0,100)
```

```
ax1.set_xlim(0,100)

ax1.set_title("Global",size=20)

#Pacific robustness



ax2.plot(RL_pLR_P,RL_mpNR_P,label='Random Links',color='black',linesty
le='-',linewidth=2)
ax2.plot(bleach_pLR_P[:,1],bleach_mpNR_P,label='Bleaching',color='#1f7
8b4',linestyle='-',linewidth=2)

ax2.plot(LT_BL_pLR_P,LT_BL_mpNR_P,label='Susceptible Link',color='#33a
02c',linestyle='-',linewidth=2)
ax2.plot(LT_HL_pLR_P,LT_HL_mpNR_P,label='Susceptible Host Link',color=
'#e31a1c',linestyle='-',linewidth=2)
ax2.plot(LT_SL_pLR_P,LT_SL_mpNR_P,label='Susceptible Symbiont Link',co
lor='#ff7f00',linestyle='-',linewidth=2)

ax2.plot(RB_pLR_P,RB_mpNR_P,label='Random Nodes',color="darkgrey",line
style='--',linewidth=3)

ax2.plot(DLB_pLR_P,DLB_mpNR_P,label='Low Degree',color="#a6cee3",lines
tyle='--',linewidth=3)

ax2.plot(DHB_pLR_P,DHB_mpNR_P,label='High Degree',color="#cab2d6",line
style='--',linewidth=3)

ax2.plot(TLB_pLR_P,TLB_mpNR_P,label='Susceptible',color="#6a3d9a",line
style='--',linewidth=3)
ax2.set_ylim(0,100)
ax2.set_xlim(0,100)

ax2.set_title("Pacific",size=20)


#Indian robustness



ax3.plot(RL_pLR_I,RL_mpNR_I,label='Random Links',color='black',linesty
le='-',linewidth=2)
ax3.plot(bleach_pLR_I[:,1],bleach_mpNR_I,label='Bleaching',color='#1f7
8b4',linestyle='-',linewidth=2)

ax3.plot(LT_BL_pLR_I,LT_BL_mpNR_I,label='Susceptible Link',color='#33a
02c',linestyle='-',linewidth=2)
ax3.plot(LT_HL_pLR_I,LT_HL_mpNR_I,label='Susceptible Host Link',color=
'#e31a1c',linestyle='-',linewidth=2)
ax3.plot(LT_SL_pLR_I,LT_SL_mpNR_I,label='Susceptible Symbiont Link',co
lor='#ff7f00',linestyle='-',linewidth=2)
```

```
ax3.plot(RB_pLR_I,RB_mpNR_I,label='Random Nodes',color="darkgrey",line
style='--',linewidth=3)

ax3.plot(DLB_pLR_I,DLB_mpNR_I,label='Low Degree',color="#a6cee3",lines
tyle='--',linewidth=3)

ax3.plot(DHB_pLR_I,DHB_mpNR_I,label='High Degree',color="#cab2d6",line
style='--',linewidth=3)

ax3.plot(TLB_pLR_I,TLB_mpNR_I,label='Susceptible',color="#6a3d9a",line
style='--',linewidth=3)
ax3.set_ylim(0,100)
ax3.set_xlim(0,100)

ax3.set_title("Indian",size=20)

#caribbean robustness



ax4.plot(RL_pLR_C,RL_mpNR_C,label='Random Links',color='black',linesty
le='-',linewidth=2)
ax4.plot(bleach_pLR_C[:,1],bleach_mpNR_C,label='Bleaching',color='#1f7
8b4',linestyle='-',linewidth=2)

ax4.plot(LT_BL_pLR_C,LT_BL_mpNR_C,label='Susceptible Link',color='#33a
02c',linestyle='-',linewidth=2)
ax4.plot(LT_HL_pLR_C,LT_HL_mpNR_C,label='Susceptible Host Link',color=
'#e31a1c',linestyle='-',linewidth=2)
ax4.plot(LT_SL_pLR_C,LT_SL_mpNR_C,label='Susceptible Symbiont Link',co
lor='#ff7f00',linestyle='-',linewidth=2)

ax4.plot(RB_pLR_C,RB_mpNR_C,label='Random Nodes',color="darkgrey",line
style='--',linewidth=3)

ax4.plot(DLB_pLR_C,DLB_mpNR_C,label='Low Degree',color="#a6cee3",lines
tyle='--',linewidth=3)

ax4.plot(DHB_pLR_C,DHB_mpNR_C,label='High Degree',color="#cab2d6",line
style='--',linewidth=3)

ax4.plot(TLB_pLR_C,TLB_mpNR_C,label='Susceptible',color="#6a3d9a",line
style='--',linewidth=3)
ax4.set_ylim(0,100)
ax4.set_xlim(0,100)

ax4.set_title("Caribbean",size=20)

fig.text(0.5, 0.01, 'Percent Removed', ha='center',size=20)
fig.text(0.08, 0.5, 'Percent Remaining', va='center', rotation='vertic
al',size=20)

legend=plt.legend( loc = 'upper center', bbox_to_anchor = (0.5, 1.1),n
```

```
col=5,
            bbox_transform = plt.gcf().transFigure ,fontsize=15)

fig.savefig("/Users/saradellwilliams/Dropbox/Williams_Suppmat_obj2/mainoceansrobustnesscurves_v4.png",transparent=False,bbox_inches='tight')
```

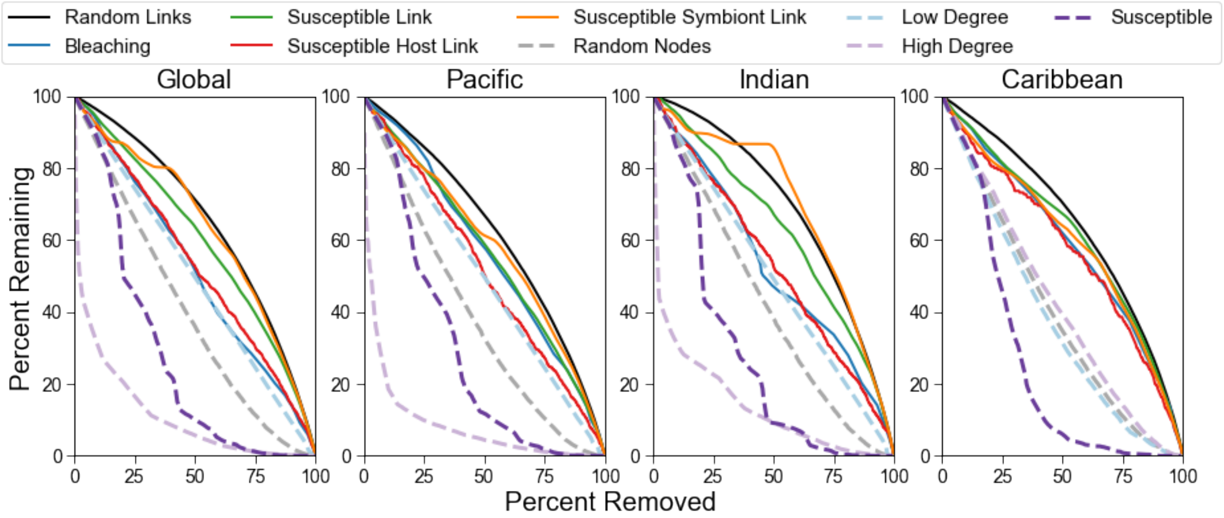


```
In [ ]:
```
