# Supplementary material for: Resistance and robustness of the global coral–symbiont network
Source: Ecology. 2020 Feb 14;101(5):e02990. doi: 10.1002/ecy.2990 (PMC7317464; doi:10.1002/ecy.2990)
Supplement: Supplementary file 4 [file ECY-101-e02990-s004.zip › DataS2/StatisticsAndVisualization_R35.nb.html]

Resistance and Robustness Visualizations and Statistics


Code 

- Show All Code
- Hide All Code
- Download Rmd

# Resistance and Robustness Visualizations and Statistics


#### Note on this code:

R version 3.5.1 (2018-07-02) Platform: x86\_64-apple-darwin15.6.0 (64-bit) Running under: macOS Sierra 10.12.6

Code written by Sara D. Williams

## Resistance Results Visualization

The following code chunk is used to make Figure 3.


```
bleachmodel<-read.csv("Resistance.csv", header=T) #compiled results for Resistance from Bleaching model, see Metadata 2 for more information.
summary(bleachmodel)
```


```
       X                   Spatial.Scale
 Min.   : 1.00   Caribbean        : 5   
 1st Qu.:18.25   Central Caribbean: 5   
 Median :35.50   Central Pacific  : 5   
 Mean   :35.50   Eastern Caribbean: 5   
 3rd Qu.:52.75   Eastern Pacific  : 5   
 Max.   :70.00   Global           : 5   
                 (Other)          :40   
       R               R_std        
 Min.   :0.09428   Min.   :0.00000  
 1st Qu.:0.19074   1st Qu.:0.01897  
 Median :0.25842   Median :0.02477  
 Mean   :0.34151   Mean   :0.02941  
 3rd Qu.:0.50396   3rd Qu.:0.03397  
 Max.   :0.88822   Max.   :0.10159  
                                    
                                 Simulation   Group   
 Network                              :14   Carib:15  
 Random Bipartite Degree Conserved    :14   Ind  :15  
 Random Bipartite Not-Degree Conserved:14   Main :20  
 Random Tolerances                    :14   Pac  :20  
 Shuffled Tolerances                  :14             
                                                      
                                                      
    statlab  
 A      : 7  
 D      : 7  
 C      : 6  
 E      : 6  
 I      : 6  
 F      : 5  
 (Other):33
```


```
theme_set(theme_grey(base_size = 14)) 
#reorder simulations
bleachmodel$Simulation<-factor(bleachmodel$Simulation,levels=c("Network","Shuffled Tolerances","Random Tolerances","Random Bipartite Degree Conserved","Random Bipartite Not-Degree Conserved"))
#plot the global and ocean-basins
Main_oceans<-bleachmodel %>%
  filter(Group=='Main')
#reorder x axis
Main_oceans$Spatial.Scale<-factor(Main_oceans$Spatial.Scale,levels=c("Global","Pacific","Indian","Caribbean"))
main<-ggplot( Main_oceans, aes(x=as.factor(Spatial.Scale), y=R,fill=Simulation)) +
  geom_bar(position=position_dodge(),stat="identity",colour='black',mapping=aes(col="red")) +
  geom_errorbar(aes(ymin=R-R_std, ymax=R+R_std), width=.2,position=position_dodge(.9))+
  scale_fill_brewer(palette="BuGn")+
  coord_cartesian(ylim=c(0,1))+
  theme(panel.background = element_blank())+ 
  theme(panel.grid.major = element_blank(), panel.grid.minor = element_blank(),
        panel.background = element_blank(), axis.line = element_line(colour = "black"))+
  labs(x="Global and Oceans",y="Resistance")+
  geom_text(aes( label=statlab,y=R+R_std+0.1),position = position_dodge(1),vjust =0)
#caribbean subregions
carib<-bleachmodel %>%
  filter(Group=='Carib')
c<-ggplot( carib, aes(x=as.factor(Spatial.Scale), y=R, fill=Simulation)) +
  geom_bar(position=position_dodge(), stat="identity",colour='black') +
  geom_errorbar(aes(ymin=R-R_std, ymax=R+R_std), width=.2,position=position_dodge(.9))+
  scale_fill_brewer(palette="BuGn")+coord_cartesian(ylim=c(0,1))+
  theme(panel.background = element_blank())+ 
  theme(panel.grid.major = element_blank(), panel.grid.minor = element_blank(),
        panel.background = element_blank(), axis.line = element_line(colour = "black"))+
  labs(x="Caribbean Regions",y=" ")+
  geom_text(aes( label=statlab,y=R+R_std+0.1),position = position_dodge(1),vjust = 0)
#Pacific subregions
pac<-bleachmodel %>%
  filter(Group=='Pac')
p<-ggplot( pac, aes(x=as.factor(Spatial.Scale), y=R, fill=Simulation)) +
  geom_bar(position=position_dodge(), stat="identity", colour='black') +
  geom_errorbar(aes(ymin=R-R_std, ymax=R+R_std), width=.2,position=position_dodge(.9))+
  scale_fill_brewer(palette="BuGn")+coord_cartesian(ylim=c(0,1))+
  theme(panel.background = element_blank())+ 
  theme(panel.grid.major = element_blank(), panel.grid.minor = element_blank(),
        panel.background = element_blank(), axis.line = element_line(colour = "black"))+
  labs(x="Pacific Regions",y=" ")+
  geom_text(aes( label=statlab,y=R+R_std+0.1),position = position_dodge(1),vjust=0)
#Indian subregions
ind<-bleachmodel %>%
  filter(Group=='Ind')
i<-ggplot( ind, aes(x=as.factor(Spatial.Scale), y=R, fill=Simulation)) +
  geom_bar(position=position_dodge(), stat="identity", colour='black') +
  geom_errorbar(aes(ymin=R-R_std, ymax=R+R_std), width=.2,position=position_dodge(.9))+
  scale_fill_brewer(palette="BuGn")+coord_cartesian(ylim=c(0,1))+theme(panel.background = element_blank())+ 
  theme(panel.grid.major = element_blank(), panel.grid.minor = element_blank(),
        panel.background = element_blank(), axis.line = element_line(colour = "black"))+
  labs(x="Indian Regions",y="Resistance")+
  geom_text(aes( label=statlab,y=R+R_std+0.1),position = position_dodge(1),vjust = 0)
#Put them all in one plot
ggarrange(main, p, i, c, ncol=2, nrow=2, common.legend = TRUE, legend="bottom")
```

## Robustness

The following code chunk is used to make Figure 4 E-H.


```
robustness<-read.csv("Robustness.csv") #results of robustness analyses, see metadata 2 for more info
summary(robustness)
```


```
    network         mean              std          
 C      : 30   Min.   :0.02041   Min.   :0.000000  
 cc     : 30   1st Qu.:0.53709   1st Qu.:0.008193  
 cp     : 30   Median :0.64939   Median :0.027461  
 ec     : 30   Mean   :0.61124   Mean   :0.037324  
 ep     : 30   3rd Qu.:0.71870   3rd Qu.:0.054356  
 G      : 30   Max.   :1.00000   Max.   :0.220333  
 (Other):240                                       
         model          removed      type    
 bleach     : 42   both     : 56   link:252  
 Degree_high: 42   hosts    : 56   node:168  
 Degree_low : 42   links    :252             
 LT_BH      : 42   symbionts: 56             
 LT_BL      : 42                             
 LT_HL      : 42                             
 (Other)    :168                             
  R50_who       group2      group        statlab   
 both :140   carib :120   carib: 90          :366  
 hosts:140   Global: 30   ind  : 90   A      :  7  
 symbs:140   ind   :120   Main :120   B      :  7  
             pac   :150   pac  :120   C      :  7  
                                      D      :  6  
                                      E      :  6  
                                      (Other): 21  
  connectance          hosts         symbionts     
 Min.   :0.01000   Min.   : 14.0   Min.   : 13.00  
 1st Qu.:0.03700   1st Qu.: 31.0   1st Qu.: 29.00  
 Median :0.06650   Median : 83.5   Median : 40.50  
 Mean   :0.07071   Mean   :144.4   Mean   : 63.21  
 3rd Qu.:0.08500   3rd Qu.:157.0   3rd Qu.: 74.00  
 Max.   :0.23600   Max.   :685.0   Max.   :250.00  
                                                   
     links       
 Min.   :  43.0  
 1st Qu.:  84.0  
 Median : 209.5  
 Mean   : 358.4  
 3rd Qu.: 404.0  
 Max.   :1697.0
```


```
#get rid of the removal models that were tested but not used
data2<-robustness %>%
    filter(model!="LT_BH", model!="LT_HH",model!="LT_SH",model!="Tolerance_high")
  
Global_tot<-data2%>%
  filter(network=="G",removed!="hosts",removed!="symbionts",R50_who=="both")
Global_tot$removed<-factor(Global_tot$removed,levels=c("links"="links","nodes"="both"))
  
global<-ggplot( Global_tot, aes(x=as.factor(model), y=mean,fill=removed))
g_total<-global+
  geom_bar(position=position_dodge(),stat="identity",colour='black',mapping=aes(col="red")) +
  geom_errorbar(aes(ymin=mean-std, ymax=mean+std), width=.2,position=position_dodge(.9))+
  scale_fill_manual(values=c("links" = "#7fc97f", "both" = "#99d8c9", "hosts" = "#a6cee3","symbionts"="#ffff99"), drop = FALSE)+
  #ylim(0,1)+
  scale_y_continuous(limits=c(0,1.05),expand = c(0,0),breaks=seq(0, 1, 0.1))+
  theme(panel.background = element_blank())+ 
  theme(panel.grid.major = element_blank(), panel.grid.minor = element_blank(),panel.background = element_blank(), axis.line = element_line(colour = "black"))+
  labs(x="",y="Robustness, R50")+
  theme(legend.position="none",axis.text.x = element_text(angle = 70, hjust = 1))+
  scale_x_discrete(limits=c("Random_link","bleach","LT_BL","LT_HL","LT_SL","Random_node","Tolerance_low","Degree_low","Degree_high"),labels=c("Random_link"="Random","bleach"="Bleaching","Tolerance_low"="Susceptible","Tolerance_high"="High Tolerance","Degree_high"= "High Degree","Degree_low"="Low Degree","Random_node"="Random Node","LT_BH"="High Avg. Link Tolerance","LT_BL"="Susceptible","LT_HH"="High Host Link Tolerance","LT_HL"="Host Susceptible","LT_SH"="High Symbiont Link Tolerance","LT_SL"="Symbiont Susceptible"))+
  geom_text(aes( label=statlab,y=mean+std+0.05),position = position_dodge(0.9),vjust = 0)
Pacific_tot<-data2%>%
  filter(network=="P",removed!="hosts",removed!="symbionts",R50_who=="both")
Pacific_tot$removed<-factor(Pacific_tot$removed,levels=c("links"="links","nodes"="both"))
  
Pacific<-ggplot( Pacific_tot, aes(x=as.factor(model), y=mean,fill=removed))
p_total<-Pacific+
  geom_bar(position=position_dodge(),stat="identity",colour='black',mapping=aes(col="red")) +
  geom_errorbar(aes(ymin=mean-std, ymax=mean+std), width=.2,position=position_dodge(.9))+
  scale_fill_manual(values=c("links" = "#7fc97f", "both" = "#99d8c9", "hosts" = "#a6cee3","symbionts"="#ffff99"), drop = FALSE)+
  #ylim(0,1)+
  scale_y_continuous(limits=c(0,1.05),expand = c(0,0),breaks=seq(0, 1, 0.1))+
  theme(panel.background = element_blank())+ 
  theme(panel.grid.major = element_blank(), panel.grid.minor = element_blank(),panel.background = element_blank(), axis.line = element_line(colour = "black"))+
  labs(x="",y="")+
  theme(legend.position="none",axis.text.x = element_text(angle = 70, hjust = 1))+
  scale_x_discrete(limits=c("Random_link","bleach","LT_BL","LT_HL","LT_SL","Random_node","Tolerance_low","Degree_low","Degree_high"),labels=c("Random_link"="Random","bleach"="Bleaching","Tolerance_low"="Susceptible","Tolerance_high"="High Tolerance","Degree_high"= "High Degree","Degree_low"="Low Degree","Random_node"="Random Node","LT_BH"="High Avg. Link Tolerance","LT_BL"="Susceptible","LT_HH"="High Host Link Tolerance","LT_HL"="Host Susceptible","LT_SH"="High Symbiont Link Tolerance","LT_SL"="Symbiont Susceptible"))+
  geom_text(aes( label=statlab,y=mean+std+0.05),position = position_dodge(0.9),vjust = 0)
Carib_tot<-data2%>%
  filter(network=="C",removed!="hosts",removed!="symbionts",R50_who=="both")
Carib_tot$removed<-factor(Carib_tot$removed,levels=c("links"="links","nodes"="both"))
Carib<-ggplot( Carib_tot, aes(x=as.factor(model), y=mean,fill=removed))
c_total<-Carib+
  geom_bar(position=position_dodge(),stat="identity",colour='black',mapping=aes(col="red")) +
  geom_errorbar(aes(ymin=mean-std, ymax=mean+std), width=.2,position=position_dodge(.9))+
  scale_fill_manual(values=c("links" = "#7fc97f", "both" = "#99d8c9", "hosts" = "#a6cee3","symbionts"="#ffff99"), drop = FALSE)+
  #ylim(0,1)+
  scale_y_continuous(limits=c(0,1.05),expand = c(0,0),breaks=seq(0, 1, 0.1))+
  theme(panel.background = element_blank())+ 
  theme(panel.grid.major = element_blank(), panel.grid.minor = element_blank(),panel.background = element_blank(), axis.line = element_line(colour = "black"))+
  labs(x="",y="")+
  theme(legend.position="none",axis.text.x = element_text(angle = 70, hjust = 1))+
  scale_x_discrete(limits=c("Random_link","bleach","LT_BL","LT_HL","LT_SL","Random_node","Tolerance_low","Degree_low","Degree_high"),labels=c("Random_link"="Random","bleach"="Bleaching","Tolerance_low"="Susceptible","Tolerance_high"="High Tolerance","Degree_high"= "High Degree","Degree_low"="Low Degree","Random_node"="Random Node","LT_BH"="High Avg. Link Tolerance","LT_BL"="Susceptible","LT_HH"="High Host Link Tolerance","LT_HL"="Host Susceptible","LT_SH"="High Symbiont Link Tolerance","LT_SL"="Symbiont Susceptible"))+
  geom_text(aes( label=statlab,y=mean+std+0.05),position = position_dodge(0.9),vjust = 0)
Ind_tot<-data2%>%
  filter(network=="I",removed!="hosts",removed!="symbionts",R50_who=="both")
Ind_tot$removed<-factor(Carib_tot$removed,levels=c("links"="links","nodes"="both"))
Ind<-ggplot( Ind_tot, aes(x=as.factor(model), y=mean,fill=removed))
i_total<-Ind+
  geom_bar(position=position_dodge(),stat="identity",colour='black',mapping=aes(col="red")) +
  geom_errorbar(aes(ymin=mean-std, ymax=mean+std), width=.2,position=position_dodge(.9))+
  scale_fill_manual(values=c("links" = "#7fc97f", "both" = "#99d8c9", "hosts" = "#a6cee3","symbionts"="#ffff99"), drop = FALSE)+
  #ylim(0,1)+
  scale_y_continuous(limits=c(0,1.05),expand = c(0,0),breaks=seq(0, 1, 0.1))+
  theme(panel.background = element_blank())+ 
  theme(panel.grid.major = element_blank(), panel.grid.minor = element_blank(),panel.background = element_blank(), axis.line = element_line(colour = "black"))+
  labs(x="",y="")+
  theme(legend.position="none",axis.text.x = element_text(angle = 70, hjust = 1))+
  scale_x_discrete(limits=c("Random_link","bleach","LT_BL","LT_HL","LT_SL","Random_node","Tolerance_low","Degree_low","Degree_high"),labels=c("Random_link"="Random","bleach"="Bleaching","Tolerance_low"="Susceptible","Tolerance_high"="High Tolerance","Degree_high"= "High Degree","Degree_low"="Low Degree","Random_node"="Random Node","LT_BH"="High Avg. Link Tolerance","LT_BL"="Susceptible","LT_HH"="High Host Link Tolerance","LT_HL"="Host Susceptible","LT_SH"="High Symbiont Link Tolerance","LT_SL"="Symbiont Susceptible"))+
  geom_text(aes( label=statlab,y=mean+std+0.05),position = position_dodge(0.9),vjust = 0)
#Put them all in one plot
ggarrange(g_total,p_total,i_total,c_total, ncol=4, nrow=1, common.legend = FALSE, legend=NULL)
```


The following code chunk is used to make Appendix S2, Figure S1 D-F.


```
myplot<-function(data2,N){ #data2=robustness results, N=network to plot
  data2<-data2 %>%
    filter(model!="LT_BH", model!="LT_HH",model!="LT_SH",model!="Tolerance_high")
  
  Global_tot<-data2%>%
    filter(network==N,removed!="hosts",removed!="symbionts",R50_who=="both")
  Global_tot$removed<-factor(Global_tot$removed,levels=c("links","both","hosts","symbionts"))
  
  global<-ggplot( Global_tot, aes(x=as.factor(model), y=mean,fill=removed))
  g_total<-global+
    geom_bar(position=position_dodge(),stat="identity",colour='black',mapping=aes(col="red")) +
    geom_errorbar(aes(ymin=mean-std, ymax=mean+std), width=.2,position=position_dodge(.9))+
    scale_fill_manual(values=c("links" = "#7fc97f", "both" = "#99d8c9", "hosts" = "#a6cee3","symbionts"="#ffff99"), 
                      drop = FALSE)+
    
    #ylim(0,1)+
    scale_y_continuous(limits=c(0,1.05),expand = c(0,0),breaks=seq(0, 1, 0.1))+
    theme(panel.background = element_blank())+ 
    theme(panel.grid.major = element_blank(), panel.grid.minor = element_blank(),
          panel.background = element_blank(), axis.line = element_line(colour = "black"))+
    labs(x="",y="Total Robustness")+
    theme(axis.text.x = element_text(angle = 70, hjust = 1))+
    scale_x_discrete(limits=c("Random_link","bleach","LT_BL","LT_HL","LT_SL","Random_node","Tolerance_low","Degree_low","Degree_high"),
                     labels=c("Random_link"="Random","bleach"="Bleaching","Tolerance_low"="Susceptible","Tolerance_high"="High Tolerance","Degree_high"= "High Degree","Degree_low"="Low Degree","Random_node"="Random Node","LT_BH"="High Avg. Link Tolerance","LT_BL"="Susceptible","LT_HH"="High Host Link Tolerance","LT_HL"="Host Susceptible","LT_SH"="High Symbiont Link Tolerance","LT_SL"="Symbiont Susceptible"))+
    geom_text(aes( label=statlab,y=mean+std+0.05),position = position_dodge(0.9),vjust = 0)
  
  Global_h<-data2%>%
    filter(network==N,removed!="hosts",removed!="both",R50_who=="hosts")
  Global_h$removed<-factor(Global_h$removed,levels=c("links","both","hosts","symbionts"))
  global_h<-ggplot( Global_h, aes(x=as.factor(model), y=mean,fill=removed))
  g_hosts<-global_h+
    geom_bar(position=position_dodge(),stat="identity",colour='black',mapping=aes(col="red")) +
    geom_errorbar(aes(ymin=mean-std, ymax=mean+std), width=.2,position=position_dodge(.9))+
    scale_fill_manual(values=c("links" = "#7fc97f", "both" = "#99d8c9", "hosts" = "#a6cee3","symbionts"="#ffff99"), 
                      drop = FALSE)+
    scale_y_continuous(limits=c(0,1.05),expand = c(0,0),breaks=seq(0, 1, 0.1))+
    theme(panel.background = element_blank())+ 
    theme(panel.grid.major = element_blank(), panel.grid.minor = element_blank(),
          panel.background = element_blank(), axis.line = element_line(colour = "black"))+
    labs(x="",y="Host Robustness")+
    theme(axis.text.x = element_text(angle = 70, hjust = 1))+
    scale_x_discrete(limits=c("Random_link","bleach","LT_BL","LT_HL","LT_SL","Random_node","Tolerance_low","Degree_low","Degree_high"),
                     labels=c("Random_link"="Random","bleach"="Bleaching","Tolerance_low"="Susceptible","Tolerance_high"="High Tolerance","Degree_high"= "High Degree","Degree_low"="Low Degree","Random_node"="Random Node","LT_BH"="High Avg. Link Tolerance","LT_BL"="Susceptible","LT_HH"="High Host Link Tolerance","LT_HL"="Host Susceptible","LT_SH"="High Symbiont Link Tolerance","LT_SL"="Symbiont Susceptible"))+
    geom_text(aes( label=statlab,y=mean+std+0.03),position = position_dodge(0.9),vjust =0)
  
  Global_s<-data2%>%
    filter(network==N,removed!="symbionts",removed!="both",R50_who=="symbs")
  Global_s$removed<-factor(Global_s$removed,levels=c("links","both","hosts","symbionts"))
  global_s<-ggplot( Global_s, aes(x=as.factor(model), y=mean,fill=removed))
  g_symbs<-global_s+
    geom_bar(position=position_dodge(),stat="identity",colour='black',mapping=aes(col="red")) +
    geom_errorbar(aes(ymin=mean-std, ymax=mean+std), width=.2,position=position_dodge(.9))+
    scale_fill_manual(values=c("links" = "#7fc97f", "both" = "#99d8c9", "hosts" = "#a6cee3","symbionts"="#ffff99"), 
                      drop = FALSE)+
    scale_y_continuous(limits=c(0,1.05),expand = c(0,0),breaks=seq(0, 1, 0.1))+
    theme(panel.background = element_blank())+ 
    theme(panel.grid.major = element_blank(), panel.grid.minor = element_blank(),
          panel.background = element_blank(), axis.line = element_line(colour = "black"))+
    labs(x="",y="Symbiont Robustness")+
    theme(axis.text.x = element_text(angle = 70, hjust = 1))+
    scale_x_discrete(limits=c("Random_link","bleach","LT_BL","LT_HL","LT_SL","Random_node","Tolerance_low","Degree_low","Degree_high"),
                     labels=c("Random_link"="Random","bleach"="Bleaching","Tolerance_low"="Susceptible","Tolerance_high"="High Tolerance","Degree_high"= "High Degree","Degree_low"="Low Degree","Random_node"="Random Node","LT_BH"="High Avg. Link Tolerance","LT_BL"="Susceptible","LT_HH"="High Host Link Tolerance","LT_HL"="Host Susceptible","LT_SH"="High Symbiont Link Tolerance","LT_SL"="Symbiont Susceptible"))+
    geom_text(aes( label=statlab,y=mean+std+0.03),position = position_dodge(0.9),vjust =0)
  
  theme_set(theme_grey(base_size = 14)) 
  p<-ggarrange(g_total,g_hosts,g_symbs, ncol=3, nrow=1, common.legend = TRUE, legend="bottom")
  return(p)
}
myplot(robustness,"G")
```

## Randomization Tests

See Appendix S2 for description. Randomization tests are often called permutation tests, thus I use “permutation” within the code.


```
mypermutation_twocomp<-function(data,netA,netB,levelA,levelB,nsims){
  #get data
  #levels(data$net_type)<-c("hsrand_dtemp", "net_dtemp", "rand_dtemp","rbdc_dtemp", "rbndc_dtemp")
  #split data for two levels
  net1 <- data%>%
    filter(net_type==netA)
  net2 <- data%>%
    filter(net_type==netB)
  twocomps<-rbind(net1,net2)
  # initialize test
  combined_resistance <- c(net1$resistance,net2$resistance) #combines resistance values into a vector
  combined_labels <- c(net1$net_type,net2$net_type)#combines network type into a vector
  diff_obs <- mean(net1$resistance) - mean(net2$resistance)
  model <- anova(lm(twocomps$resistance ~ twocomps$net_type)) 
  obt.F <- model$"F value"[1]     # Our obtained F  statistic
  obt.p <- model$"Pr(>F)"[1]
  
  # permutation test
  diffs <- rep(NA, nsims)
  fstats<-rep(NA,nsims)
  for (i in 1:nsims) {
    #shuffle the labels
    shuffled_labels <- sample(combined_labels, replace = FALSE)
    twocomps$shuffled_labels<-shuffled_labels
    #shuffle the resistances
    shuffled_resistance<-sample(combined_resistance, replace = FALSE)
    twocomps$shuffled_resistance<-shuffled_resistance
    #get the differences in the means of the shuffled groups
    diffs[i] <- mean(shuffled_resistance[shuffled_labels == levelA]) -  mean(shuffled_resistance[shuffled_labels == levelB])
    #run a new anova
    newmodel <- anova(lm(twocomps$shuffled_resistance ~ twocomps$shuffled_labels)) 
    fstats[i] <- newmodel$"F value"[1]     # get a new F  statistic
  }
  #calculate the two-sided p-value
  # p-value = (number of more extreme differences than diff_obs)/nsims
  pval_diffs<-length(diffs[abs(diffs) >= abs(diff_obs)])/nsims
  pval_fstatdifs<-(length(fstats[abs(fstats) > abs(obt.F)])+1)/(1+nsims)
  return(c(diff_obs,obt.p,obt.F,pval_diffs,pval_fstatdifs))
}
resistance_perms<-function(file,nsims){
  #get data
  resist_data<-read.csv(file)
  #select columns
  selectdata<-resist_data%>%
    select(hsrand_dtemp,net_dtemp,rand_dtemp,rbdc_dtemp,rbndc_dtemp)
  #get into long format
  longdata <- gather(selectdata, key=net_type,value=resistance,factor_key = TRUE)
  #setup storage for permutation results
  resmat<-matrix(NA,nrow=0,ncol=7)
  pvals<-rep()
  #run permutations
  for (j in 1:(length(levels(longdata$net_type))-1)){
    netA<-levels(longdata$net_type)[j]
    for (i in (j+1):length(levels(longdata$net_type))){
      netB<-levels(longdata$net_type)[i]
      levelA<-j
      levelB<-i
      #print(c(netA,netB,levelA,levelB))
      permresults<-mypermutation_twocomp(longdata,netA,netB,levelA,levelB,1000)
      #print(results)
      #pvals<-append(pvals,permresults[5])
      permres<-c(permresults,netA,netB)
      resmat<-rbind(resmat,permres)
  }
}
  all_results<-cbind(resmat,p.adjust(as.numeric(resmat[,5]),"holm")) #adjust p values using the holm correction
  results<-as.data.frame(all_results)
  colnames(results)<-c("diff_obs","obt.p","obt.F","pval_diffs","pval_fstatdifs","net1","net2","p_adjust_holm")
  return(results)
}
#TEST
resistance_perms("TEST_resistance_perms.csv",1000)
```


```
                     diff_obs                obt.p
permres   0.00412371134020573    0.702317494689414
permres.1  -0.436082474226805 6.33757407839271e-88
permres.2  -0.152577319587629 2.49559940772465e-38
permres.3  -0.121649484536083 5.37670669053595e-26
permres.4  -0.440206185567011 3.32885050387969e-86
permres.5  -0.156701030927835 5.16592722639757e-37
permres.6  -0.125773195876289 1.85944491914943e-25
permres.7   0.283505154639176 2.96101092082189e-63
permres.8   0.314432989690722 3.42201761802395e-67
permres.9  0.0309278350515463 0.000594160717217541
                      obt.F pval_diffs
permres   0.146508966043483      0.776
permres.1   1318.0773480663          0
permres.2  268.554789272025          0
permres.3  151.108297535609          0
permres.4  1257.08014938236          0
permres.5  254.297638156382          0
permres.6  146.730745532962          0
permres.7  644.188110026627          0
permres.8  726.876119160036          0
permres.9  12.1964991530209      0.001
                pval_fstatdifs         net1
permres      0.725274725274725 hsrand_dtemp
permres.1 0.000999000999000999 hsrand_dtemp
permres.2 0.000999000999000999 hsrand_dtemp
permres.3 0.000999000999000999 hsrand_dtemp
permres.4 0.000999000999000999    net_dtemp
permres.5 0.000999000999000999    net_dtemp
permres.6 0.000999000999000999    net_dtemp
permres.7 0.000999000999000999   rand_dtemp
permres.8 0.000999000999000999   rand_dtemp
permres.9    0.001998001998002   rbdc_dtemp
                 net2       p_adjust_holm
permres     net_dtemp   0.725274725274725
permres.1  rand_dtemp 0.00999000999000999
permres.2  rbdc_dtemp 0.00999000999000999
permres.3 rbndc_dtemp 0.00999000999000999
permres.4  rand_dtemp 0.00999000999000999
permres.5  rbdc_dtemp 0.00999000999000999
permres.6 rbndc_dtemp 0.00999000999000999
permres.7  rbdc_dtemp 0.00999000999000999
permres.8 rbndc_dtemp 0.00999000999000999
permres.9 rbndc_dtemp 0.00999000999000999
```

## Robustness vs. Connectance

See Appendix S2 for description.


```
#load data
robustness<-read.csv("Robustness.csv")
#check distribution of connectance
plot(density((log(robustness$connectance)))) #It's wonky.
```


```
#make it easy to plot removal model of choice vs connectance with a linear fit
my_RClinreg_plot<-function(choice){
  data<-robustness %>%
    filter(model!="LT_BH", model!="LT_HH",model!="LT_SH",model!="Tolerance_high",R50_who=="both",model==choice)
  p<-ggplotRegression(lm(mean ~ connectance, data = data),choice)
  p+geom_point(y=data$mean,x=data$connectance,aes(color=data$group),size=6)
  
}
#run a Kendall's coefficient of rank correlation test on removal model of choice
my_cortest<-function(choice){
  data<-robustness %>%
    filter(model!="LT_BH", model!="LT_HH",model!="LT_SH",model!="Tolerance_high",R50_who=="both",model==choice)
  ct<-cor.test(data$mean,data$connectance,method="kendall")
  return(ct)
}
#run for different removal models 
my_RClinreg_plot("bleach")
```


```
my_cortest("bleach")
```


```
    Kendall's rank correlation tau

data:  data$mean and data$connectance
T = 67, p-value = 0.01928
alternative hypothesis: true tau is not equal to 0
sample estimates:
      tau 
0.4725275
```


```
my_RClinreg_plot("Random_link")
```


```
my_cortest("Random_link")
```


```
    Kendall's rank correlation tau

data:  data$mean and data$connectance
T = 52, p-value = 0.5183
alternative hypothesis: true tau is not equal to 0
sample estimates:
      tau 
0.1428571
```


```
my_RClinreg_plot("LT_BL")
```


```
my_cortest("LT_BL")
```


```
    Kendall's rank correlation tau

data:  data$mean and data$connectance
T = 55, p-value = 0.3308
alternative hypothesis: true tau is not equal to 0
sample estimates:
      tau 
0.2087912
```


```
my_RClinreg_plot("LT_HL")
```


```
my_cortest("LT_HL")
```


```
    Kendall's rank correlation tau

data:  data$mean and data$connectance
T = 56, p-value = 0.2792
alternative hypothesis: true tau is not equal to 0
sample estimates:
      tau 
0.2307692
```


```
my_RClinreg_plot("LT_SL")
```


```
my_cortest("LT_SL")
```


```
    Kendall's rank correlation tau

data:  data$mean and data$connectance
T = 42, p-value = 0.7472
alternative hypothesis: true tau is not equal to 0
sample estimates:
        tau 
-0.07692308
```


```
my_RClinreg_plot("Degree_high")
```


```
my_cortest("Degree_high")
```


```
    Kendall's rank correlation tau

data:  data$mean and data$connectance
T = 72, p-value = 0.003025
alternative hypothesis: true tau is not equal to 0
sample estimates:
      tau 
0.5824176
```


```
my_RClinreg_plot("Degree_low")
```


```
my_cortest("Degree_low")
```


```
    Kendall's rank correlation tau

data:  data$mean and data$connectance
T = 27, p-value = 0.04718
alternative hypothesis: true tau is not equal to 0
sample estimates:
       tau 
-0.4065934
```


```
my_RClinreg_plot("Random_node")
```


```
my_cortest("Random_node")
```


```
    Kendall's rank correlation tau

data:  data$mean and data$connectance
T = 47, p-value = 0.9145
alternative hypothesis: true tau is not equal to 0
sample estimates:
       tau 
0.03296703
```


```
my_RClinreg_plot("Tolerance_low")
```


```
my_cortest("Tolerance_low")
```


```
    Kendall's rank correlation tau

data:  data$mean and data$connectance
T = 56, p-value = 0.2792
alternative hypothesis: true tau is not equal to 0
sample estimates:
      tau 
0.2307692
```

## Symbiont node thermal tolerances: picking missing tolerances

Code for Appendix S1, Figure S1: Frequency distribution of Symbiodinium thermal tolerance scores adapted from Swain et al. (2017); distribution is colored by tolerance range, red is highly susceptible, orange is medium tolerance, and yellow is high tolerance. Swain et al. (2017) provides a framework for a consensus of Symbiodinium thermotolerance ranks developed from rank-aggregation methods. Their ranking scheme orders Symbiodinium phylotypes from 0-100, but the rank values are not indicative of total magnitude differences in thermotolerance. To determine tolerances of the unlisted symbiont types in our network, rank values were randomly drawn from the high, medium, and low thermal tolerance frequency distributions in the relative proportions of the clades represented in those distributions. Thus, for each simulation of either the bleaching or different removal models described below, the symbiont tolerances varied within a set distribution (Figure 1D).


```
### Exploring data from Swain et al 2016a ####
data<-read.csv("SwainSymbsR.csv")
#frequency distribution --> inset from figure 1 from paper
bins=seq(0,100,by=2)
#split the frequency distribution into 3 subsets defined by swain et al 16
low <- data[which(data$ScoreR_k<=17),]
high <- data[which(data$ScoreR_k>=33),]
middle<- data[which(data$ScoreR_k>17 & data$ScoreR_k<33),]
#High hist
hist(high$ScoreR_k, breaks=bins, col="yellow", ylim=c(0,12), xlab="thermotolerance score", main="Frequency Distributions of Thermotolerance Scores")
#middle hist
hist(middle$ScoreR_k, breaks=bins, col="orange", add=T)
```


```
#low hist
hist(low$ScoreR_k, breaks=bins, col="red", add=T)
```


Code for fitting the above thermal toelrance distributions distributions


```
### Fit the Thermotolerance Distributions ####
#first need the mixtools package
if(!("mixtools" %in% installed.packages())){install.packages("mixtools")}
```


```
Warning in install.packages :
  cannot open URL 'https://cran.rstudio.com/bin/macosx/el-capitan/contrib/3.5/PACKAGES.rds': HTTP status was '404 Not Found'
```


```
trying URL 'https://cran.rstudio.com/bin/macosx/el-capitan/contrib/3.5/mixtools_1.1.0.tgz'
Content type 'application/x-gzip' length 1413097 bytes (1.3 MB)
==================================================
downloaded 1.3 MB
```


```
The downloaded binary packages are in
    /var/folders/9_/h1rwfkm916nc6pcl1dsx6r6h0000gn/T//Rtmpfw2FHQ/downloaded_packages
```


```
library("mixtools")
```


```
mixtools package, version 1.1.0, Released 2017-03-10
This package is based upon work supported by the National Science Foundation under Grant No. SES-0518772.
```


```
#Use the expectation Maximization (EM) Approach
em <- normalmixEM(data$ScoreR_k, arbvar = TRUE,epsilon = 1e-03, k = 3)
```


```
number of iterations= 8
```


```
# Estimated means
em$mu
```


```
[1]  7.993676 34.266710 54.339110
```


```
# Estimated standard deviations
em$sigma
```


```
[1]  2.531190 17.196483  1.094525
```


```
###RESULTS
#> em$mu
#[1]  7.983029 29.408570 51.900339
#> em$sigma
#[1]  2.711480  9.500587 18.006626
#do 10000 simulations ####
runs <- 10000
sims.low <- rnorm(runs,mean=7.983029,sd=2.711480)
sims.middle <-rnorm(runs,mean=29.408570,sd=9.500587)
sims.high<-rnorm(runs,mean=51.900339,sd=18.006626)
#High hist
hist(sims.high, col="yellow")
#middle hist
hist(sims.middle, col="orange", add=T)
```


```
#low hist
hist(sims.low, col="red", add=T)
```


Code for settingup for toelrancea function


```
#VALUES THAT ARE NEEDED #### 
##These are just the numbers of symbs from each clade in each freq level from Swain
swain_A<-8
swain_B<-8
swain_C<-71
swain_D<-19
swain_E<-1
swain_F<-3
low_A<-1
middle_A<-3
high_A<-4
prob_low_A<-low_A/swain_A
prob_middle_A<-middle_A/swain_A
prob_high_A<-high_A/swain_A
low_B<-3
middle_B<-4
high_B<-1
prob_low_B<-low_B/swain_B
prob_middle_B<-middle_B/swain_B
prob_high_B<-high_B/swain_B
low_C<-24
middle_C<-19
high_C<-28
prob_low_C<-low_C/swain_C
prob_middle_C<-middle_C/swain_C
prob_high_C<-high_C/swain_C
low_D<-3
middle_D<-4
high_D<-12
prob_low_D<-low_D/swain_D
prob_middle_D<-middle_D/swain_D
prob_high_D<-high_D/swain_D
low_E<-1
prob_low_E<-1
prob_middle_E<-0
prob_high_E<-0
high_F<-3
prob_low_F<-0
prob_middle_F<-0
prob_high_F<-1
#OK so these are the number of symbs in each clade that dont have tolerances 
net_A<-6
net_B<-13
net_C<-160
net_D<-6
net_E<-0
net_F<-0
```


Function for getting the tolerances


```
get_tols<-function(size,lowprob,midprob,highprob,runs,sims.low,sims.middle,sims.high){
  #make a sample distribution where 1 corresponds to the low thermaltolerance,
  # 2 is the medium and 3 is high. 1:3 are put into the sample distribution 
  #based on the probabilities in the function input that are clade specific
  sampledistr<-sample(x = 1:3, size = size, prob = c(lowprob, midprob, highprob), replace=TRUE)
  tolerance<-numeric(length=size)
  for (i in 1:size) { #for each number in the sampledistr do:
    if (sampledistr[i]==1){
      tolerance[i]<-sample(x=sims.low,size=1) #pull from low
    }
    else if (sampledistr[i]==2){ #pull from middle
      tolerance[i]<-sample(x=sims.middle,size=1)
    }
    else 
      tolerance[i]<-sample(x=sims.high,size=1) #pull from high
  }
  tolerance<-sqrt(tolerance)/10 #take the sqrt because swain's tolerances are not indicative of magnitude and then divide by 10 to get a number from 0-1
  return(tolerance)
}
```


Make 100 simulations worth of tolerance files


```
trials=1 #actually did 100, but don't want to make a ton of new files, so here is 1 example file.
lst=seq(1:trials)
for(i in seq_along(lst)){
  Ctols<-get_tols(net_C,prob_low_C,prob_middle_C,prob_high_C,10000,sims.low,sims.middle,sims.high)
  Atols<-get_tols(net_A,prob_low_A,prob_middle_A,prob_high_A,10000,sims.low,sims.middle,sims.high)
  Btols<-get_tols(net_B,prob_low_B,prob_middle_B,prob_high_B,10000,sims.low,sims.middle,sims.high)
  Dtols<-get_tols(net_D,prob_low_D,prob_middle_D,prob_high_D,10000,sims.low,sims.middle,sims.high)
  Ctols<-as.data.frame(Ctols)
  Atols<-as.data.frame(Atols)
  Btols<-as.data.frame(Btols)
  Dtols<-as.data.frame(Dtols)
  colnames(Ctols) <- c("tolerance")
  colnames(Btols) <- c("tolerance")
  colnames(Dtols) <- c("tolerance")
  colnames(Atols) <- c("tolerance")
  newscores<-rbind(Atols,Btols,Ctols,Dtols)
  name<-paste('trial',i,".csv",sep="")
  
  write.csv(newscores,name,row.names=FALSE)
}
```


Now for the shuffled tolerances:


```
# Now get the toleranes for the Shuffled Symbionts/ Shuffled Random null model ####
#for the random set of tolerances where they are all pulled equally from the 3 distributions
runs=10000
sims.low <- rnorm(runs,mean=7.983029,sd=2.711480)
sims.low<-subset(sims.low,sims.low>=0)
sims.middle <-rnorm(runs,mean=29.408570,sd=9.500587)
sims.middle<-subset(sims.middle,sims.middle>=0)
sims.high<-rnorm(runs,mean=51.900339,sd=18.006626) 
sims.high<-subset(sims.high,sims.high<=100)
sims.high<-subset(sims.high,sims.high>=0)
#so the following function does what the old one did but also specifies
#if using probabilies determined from data or if pulling all values from 
#the distributions with the same probability (1/3)
allthedata<-function(probability,trials,sims.low,sims.middle,sims.high,net_A,net_B,net_C,net_D){
  if (probability==1){
    swain_A<-8
    swain_B<-8
    swain_C<-71
    swain_D<-19
    swain_E<-1
    swain_F<-3
    
    low_A<-1
    middle_A<-3
    high_A<-4
    prob_low_A<-low_A/swain_A
    prob_middle_A<-middle_A/swain_A
    prob_high_A<-high_A/swain_A
    
    low_B<-3
    middle_B<-4
    high_B<-1
    prob_low_B<-low_B/swain_B
    prob_middle_B<-middle_B/swain_B
    prob_high_B<-high_B/swain_B
    
    low_C<-24
    middle_C<-19
    high_C<-28
    prob_low_C<-low_C/swain_C
    prob_middle_C<-middle_C/swain_C
    prob_high_C<-high_C/swain_C
    
    low_D<-3
    middle_D<-4
    high_D<-12
    prob_low_D<-low_D/swain_D
    prob_middle_D<-middle_D/swain_D
    prob_high_D<-high_D/swain_D
    
    low_E<-1
    prob_low_E<-1
    prob_middle_E<-0
    prob_high_E<-0
    
    high_F<-3
    prob_low_F<-0
    prob_middle_F<-0
    prob_high_F<-1
    
  }
  else
    prob_low_C=prob_middle_C=prob_high_C=prob_low_A=prob_middle_A=prob_high_A=prob_low_B=prob_middle_B=prob_high_B=prob_low_D=prob_middle_D=prob_high_D=(1/3)
  
  #net_A<-8
  #net_B<-18
  #net_C<-177
  #net_D<-7
  #net_E<-0
  #net_F<-0
  lst=seq(1:trials)
  for(i in seq_along(lst)){
    Ctols<-get_tols(net_C,prob_low_C,prob_middle_C,prob_high_C,10000,sims.low,sims.middle,sims.high)
    Atols<-get_tols(net_A,prob_low_A,prob_middle_A,prob_high_A,10000,sims.low,sims.middle,sims.high)
    Btols<-get_tols(net_B,prob_low_B,prob_middle_B,prob_high_B,10000,sims.low,sims.middle,sims.high)
    Dtols<-get_tols(net_D,prob_low_D,prob_middle_D,prob_high_D,10000,sims.low,sims.middle,sims.high)
    Ctols<-as.data.frame(Ctols)
    Atols<-as.data.frame(Atols)
    Btols<-as.data.frame(Btols)
    Dtols<-as.data.frame(Dtols)
    colnames(Ctols) <- c("tolerance")
    colnames(Btols) <- c("tolerance")
    colnames(Dtols) <- c("tolerance")
    colnames(Atols) <- c("tolerance")
    newscores<-rbind(Atols,Btols,Ctols,Dtols)
    name<-paste('rtrial',i,".csv",sep="")
    
    write.csv(newscores,name,row.names=FALSE)
  }
}
#size of the clades in the network
net_A<-10
net_B<-20
net_C<-211
net_D<-9
allthedata(0,1,sims.low,sims.middle,sims.high,net_A,net_B,net_C,net_D)
```

# Revised Figures

Figure 4, now without the degree removal models


```
robustness<-read.csv("Robustness.csv") #results of robustness analyses, see metadata 2 for more info
summary(robustness)
```


```
    network         mean              std                   model          removed   
 C      : 30   Min.   :0.02041   Min.   :0.000000   bleach     : 42   both     : 56  
 cc     : 30   1st Qu.:0.53709   1st Qu.:0.008193   Degree_high: 42   hosts    : 56  
 cp     : 30   Median :0.64939   Median :0.027461   Degree_low : 42   links    :252  
 ec     : 30   Mean   :0.61124   Mean   :0.037324   LT_BH      : 42   symbionts: 56  
 ep     : 30   3rd Qu.:0.71870   3rd Qu.:0.054356   LT_BL      : 42                  
 G      : 30   Max.   :1.00000   Max.   :0.220333   LT_HL      : 42                  
 (Other):240                                        (Other)    :168                  
   type      R50_who       group2      group        statlab     connectance     
 link:252   both :140   carib :120   carib: 90          :366   Min.   :0.01000  
 node:168   hosts:140   Global: 30   ind  : 90   A      :  7   1st Qu.:0.03700  
            symbs:140   ind   :120   Main :120   B      :  7   Median :0.06650  
                        pac   :150   pac  :120   C      :  7   Mean   :0.07071  
                                                 D      :  6   3rd Qu.:0.08500  
                                                 E      :  6   Max.   :0.23600  
                                                 (Other): 21                    
     hosts         symbionts          links       
 Min.   : 14.0   Min.   : 13.00   Min.   :  43.0  
 1st Qu.: 31.0   1st Qu.: 29.00   1st Qu.:  84.0  
 Median : 83.5   Median : 40.50   Median : 209.5  
 Mean   :144.4   Mean   : 63.21   Mean   : 358.4  
 3rd Qu.:157.0   3rd Qu.: 74.00   3rd Qu.: 404.0  
 Max.   :685.0   Max.   :250.00   Max.   :1697.0
```


```
#get rid of the removal models that were tested but not used
data2<-robustness %>%
    filter(model!="LT_BH", model!="LT_HH",model!="LT_SH",model!="Tolerance_high",model!="Degree_high",model!="Degree_low")%>%
  droplevels()
levels(data2$model)
```


```
[1] "bleach"        "LT_BL"         "LT_HL"         "LT_SL"         "Random_link"  
[6] "Random_node"   "Tolerance_low"
```


```
Global_tot<-data2%>%
  filter(network=="G",removed!="hosts",removed!="symbionts",R50_who=="both")
Global_tot$removed<-factor(Global_tot$removed,levels=c("links"="links","nodes"="both"))
  
global<-ggplot( Global_tot, aes(x=as.factor(model), y=mean,fill=removed))
g_total<-global+
  geom_bar(position=position_dodge(),stat="identity",colour='black',mapping=aes(col="red")) +
  geom_errorbar(aes(ymin=mean-std, ymax=mean+std), width=.2,position=position_dodge(.9))+
  scale_fill_manual(values=c("links" = "#7fc97f", "both" = "#99d8c9", "hosts" = "#a6cee3","symbionts"="#ffff99"), drop = FALSE)+
  #ylim(0,1)+
  scale_y_continuous(limits=c(0,1.05),expand = c(0,0),breaks=seq(0, 1, 0.1))+
  theme(panel.background = element_blank())+ 
  theme(panel.grid.major = element_blank(), panel.grid.minor = element_blank(),panel.background = element_blank(), axis.line = element_line(colour = "black"))+
  labs(x="",y="Robustness, R50")+
  theme(legend.position="none",axis.text.x = element_text(angle = 70, hjust = 1))+
  scale_x_discrete(limits=c("Random_link","bleach","LT_BL","LT_HL","LT_SL","Random_node","Tolerance_low"),labels=c("Random_link"="Random","bleach"="Bleaching","Tolerance_low"="Susceptible","Tolerance_high"="High Tolerance","Degree_high"= "High Degree","Degree_low"="Low Degree","Random_node"="Random Node","LT_BH"="High Avg. Link Tolerance","LT_BL"="Susceptible","LT_HH"="High Host Link Tolerance","LT_HL"="Host Susceptible","LT_SH"="High Symbiont Link Tolerance","LT_SL"="Symbiont Susceptible"))+
  geom_text(aes( label=statlab,y=mean+std+0.05),position = position_dodge(0.9),vjust = 0)
Pacific_tot<-data2%>%
  filter(network=="P",removed!="hosts",removed!="symbionts",R50_who=="both")
Pacific_tot$removed<-factor(Pacific_tot$removed,levels=c("links"="links","nodes"="both"))
  
Pacific<-ggplot( Pacific_tot, aes(x=as.factor(model), y=mean,fill=removed))
p_total<-Pacific+
  geom_bar(position=position_dodge(),stat="identity",colour='black',mapping=aes(col="red")) +
  geom_errorbar(aes(ymin=mean-std, ymax=mean+std), width=.2,position=position_dodge(.9))+
  scale_fill_manual(values=c("links" = "#7fc97f", "both" = "#99d8c9", "hosts" = "#a6cee3","symbionts"="#ffff99"), drop = FALSE)+
  #ylim(0,1)+
  scale_y_continuous(limits=c(0,1.05),expand = c(0,0),breaks=seq(0, 1, 0.1))+
  theme(panel.background = element_blank())+ 
  theme(panel.grid.major = element_blank(), panel.grid.minor = element_blank(),panel.background = element_blank(), axis.line = element_line(colour = "black"))+
  labs(x="",y="")+
  theme(legend.position="none",axis.text.x = element_text(angle = 70, hjust = 1))+
  scale_x_discrete(limits=c("Random_link","bleach","LT_BL","LT_HL","LT_SL","Random_node","Tolerance_low"),labels=c("Random_link"="Random","bleach"="Bleaching","Tolerance_low"="Susceptible","Tolerance_high"="High Tolerance","Degree_high"= "High Degree","Degree_low"="Low Degree","Random_node"="Random Node","LT_BH"="High Avg. Link Tolerance","LT_BL"="Susceptible","LT_HH"="High Host Link Tolerance","LT_HL"="Host Susceptible","LT_SH"="High Symbiont Link Tolerance","LT_SL"="Symbiont Susceptible"))+
  geom_text(aes( label=statlab,y=mean+std+0.05),position = position_dodge(0.9),vjust = 0)
Carib_tot<-data2%>%
  filter(network=="C",removed!="hosts",removed!="symbionts",R50_who=="both")
Carib_tot$removed<-factor(Carib_tot$removed,levels=c("links"="links","nodes"="both"))
Carib<-ggplot( Carib_tot, aes(x=as.factor(model), y=mean,fill=removed))
c_total<-Carib+
  geom_bar(position=position_dodge(),stat="identity",colour='black',mapping=aes(col="red")) +
  geom_errorbar(aes(ymin=mean-std, ymax=mean+std), width=.2,position=position_dodge(.9))+
  scale_fill_manual(values=c("links" = "#7fc97f", "both" = "#99d8c9", "hosts" = "#a6cee3","symbionts"="#ffff99"), drop = FALSE)+
  #ylim(0,1)+
  scale_y_continuous(limits=c(0,1.05),expand = c(0,0),breaks=seq(0, 1, 0.1))+
  theme(panel.background = element_blank())+ 
  theme(panel.grid.major = element_blank(), panel.grid.minor = element_blank(),panel.background = element_blank(), axis.line = element_line(colour = "black"))+
  labs(x="",y="")+
  theme(legend.position="none",axis.text.x = element_text(angle = 70, hjust = 1))+
  scale_x_discrete(limits=c("Random_link","bleach","LT_BL","LT_HL","LT_SL","Random_node","Tolerance_low"),labels=c("Random_link"="Random","bleach"="Bleaching","Tolerance_low"="Susceptible","Tolerance_high"="High Tolerance","Degree_high"= "High Degree","Degree_low"="Low Degree","Random_node"="Random Node","LT_BH"="High Avg. Link Tolerance","LT_BL"="Susceptible","LT_HH"="High Host Link Tolerance","LT_HL"="Host Susceptible","LT_SH"="High Symbiont Link Tolerance","LT_SL"="Symbiont Susceptible"))+
  geom_text(aes( label=statlab,y=mean+std+0.05),position = position_dodge(0.9),vjust = 0)
Ind_tot<-data2%>%
  filter(network=="I",removed!="hosts",removed!="symbionts",R50_who=="both")
Ind_tot$removed<-factor(Carib_tot$removed,levels=c("links"="links","nodes"="both"))
Ind<-ggplot( Ind_tot, aes(x=as.factor(model), y=mean,fill=removed))
i_total<-Ind+
  geom_bar(position=position_dodge(),stat="identity",colour='black',mapping=aes(col="red")) +
  geom_errorbar(aes(ymin=mean-std, ymax=mean+std), width=.2,position=position_dodge(.9))+
  scale_fill_manual(values=c("links" = "#7fc97f", "both" = "#99d8c9", "hosts" = "#a6cee3","symbionts"="#ffff99"), drop = FALSE)+
  #ylim(0,1)+
  scale_y_continuous(limits=c(0,1.05),expand = c(0,0),breaks=seq(0, 1, 0.1))+
  theme(panel.background = element_blank())+ 
  theme(panel.grid.major = element_blank(), panel.grid.minor = element_blank(),panel.background = element_blank(), axis.line = element_line(colour = "black"))+
  labs(x="",y="")+
  theme(legend.position="none",axis.text.x = element_text(angle = 70, hjust = 1))+
  scale_x_discrete(limits=c("Random_link","bleach","LT_BL","LT_HL","LT_SL","Random_node","Tolerance_low"),labels=c("Random_link"="Random","bleach"="Bleaching","Tolerance_low"="Susceptible","Tolerance_high"="High Tolerance","Degree_high"= "High Degree","Degree_low"="Low Degree","Random_node"="Random Node","LT_BH"="High Avg. Link Tolerance","LT_BL"="Susceptible","LT_HH"="High Host Link Tolerance","LT_HL"="Host Susceptible","LT_SH"="High Symbiont Link Tolerance","LT_SL"="Symbiont Susceptible"))+
  geom_text(aes( label=statlab,y=mean+std+0.05),position = position_dodge(0.9),vjust = 0)
#Put them all in one plot
ggarrange(g_total,p_total,i_total,c_total, ncol=4, nrow=1, common.legend = FALSE, legend=NULL)
```

LS0tCnRpdGxlOiAiUmVzaXN0YW5jZSBhbmQgUm9idXN0bmVzcyBWaXN1YWxpemF0aW9ucyBhbmQgU3RhdGlzdGljcyIKb3V0cHV0OgogIGh0bWxfbm90ZWJvb2s6IGRlZmF1bHQKICBwZGZfZG9jdW1lbnQ6IGRlZmF1bHQKICB3b3JkX2RvY3VtZW50OiBkZWZhdWx0Ci0tLQpgYGB7ciBzZXR1cCwgaW5jbHVkZT1GQUxTRX0Ka25pdHI6Om9wdHNfY2h1bmskc2V0KGVjaG8gPSBUUlVFKQprbml0cjo6b3B0c19jaHVuayRzZXQoZmlnLndpZHRoPTEyLCBmaWcuaGVpZ2h0PTgpIAojbG9hZCBwYWNrYWdlcyBuZWVkZWQKbGlicmFyeSh0aWR5dmVyc2UpCmxpYnJhcnkoZ2dwdWJyKQpsaWJyYXJ5KHZlZ2FuKQpsaWJyYXJ5KGNvaW4pCnNldHdkKCJ+L0Ryb3Bib3gvTXlfUHVibGljYXRpb25zL0dsb2JhbEJsZWFjaGluZ01vZGVsUmFuZFIvRWNvbG9neVN1Ym1pc3Npb24vU00vRGF0YVMyIikgI3NldCB5b3VyIGNvcnJlY3Qgd29ya2luZyBkaXJlY3RvcnkKYGBgCiMjIyMgTm90ZSBvbiB0aGlzIGNvZGU6ClIgdmVyc2lvbiAzLjUuMSAoMjAxOC0wNy0wMikKUGxhdGZvcm06IHg4Nl82NC1hcHBsZS1kYXJ3aW4xNS42LjAgKDY0LWJpdCkKUnVubmluZyB1bmRlcjogbWFjT1MgU2llcnJhIDEwLjEyLjYKCkNvZGUgd3JpdHRlbiBieSBTYXJhIEQuIFdpbGxpYW1zCgojIyBSZXNpc3RhbmNlIFJlc3VsdHMgVmlzdWFsaXphdGlvbgoKVGhlIGZvbGxvd2luZyBjb2RlIGNodW5rIGlzIHVzZWQgdG8gbWFrZSBGaWd1cmUgMy4KCmBgYHtyfQpibGVhY2htb2RlbDwtcmVhZC5jc3YoIlJlc2lzdGFuY2UuY3N2IiwgaGVhZGVyPVQpICNjb21waWxlZCByZXN1bHRzIGZvciBSZXNpc3RhbmNlIGZyb20gQmxlYWNoaW5nIG1vZGVsLCBzZWUgTWV0YWRhdGEgMiBmb3IgbW9yZSBpbmZvcm1hdGlvbi4Kc3VtbWFyeShibGVhY2htb2RlbCkKdGhlbWVfc2V0KHRoZW1lX2dyZXkoYmFzZV9zaXplID0gMTQpKSAKI3Jlb3JkZXIgc2ltdWxhdGlvbnMKYmxlYWNobW9kZWwkU2ltdWxhdGlvbjwtZmFjdG9yKGJsZWFjaG1vZGVsJFNpbXVsYXRpb24sbGV2ZWxzPWMoIk5ldHdvcmsiLCJTaHVmZmxlZCBUb2xlcmFuY2VzIiwiUmFuZG9tIFRvbGVyYW5jZXMiLCJSYW5kb20gQmlwYXJ0aXRlIERlZ3JlZSBDb25zZXJ2ZWQiLCJSYW5kb20gQmlwYXJ0aXRlIE5vdC1EZWdyZWUgQ29uc2VydmVkIikpCgojcGxvdCB0aGUgZ2xvYmFsIGFuZCBvY2Vhbi1iYXNpbnMKTWFpbl9vY2VhbnM8LWJsZWFjaG1vZGVsICU+JQogIGZpbHRlcihHcm91cD09J01haW4nKQojcmVvcmRlciB4IGF4aXMKTWFpbl9vY2VhbnMkU3BhdGlhbC5TY2FsZTwtZmFjdG9yKE1haW5fb2NlYW5zJFNwYXRpYWwuU2NhbGUsbGV2ZWxzPWMoIkdsb2JhbCIsIlBhY2lmaWMiLCJJbmRpYW4iLCJDYXJpYmJlYW4iKSkKbWFpbjwtZ2dwbG90KCBNYWluX29jZWFucywgYWVzKHg9YXMuZmFjdG9yKFNwYXRpYWwuU2NhbGUpLCB5PVIsZmlsbD1TaW11bGF0aW9uKSkgKwogIGdlb21fYmFyKHBvc2l0aW9uPXBvc2l0aW9uX2RvZGdlKCksc3RhdD0iaWRlbnRpdHkiLGNvbG91cj0nYmxhY2snLG1hcHBpbmc9YWVzKGNvbD0icmVkIikpICsKICBnZW9tX2Vycm9yYmFyKGFlcyh5bWluPVItUl9zdGQsIHltYXg9UitSX3N0ZCksIHdpZHRoPS4yLHBvc2l0aW9uPXBvc2l0aW9uX2RvZGdlKC45KSkrCiAgc2NhbGVfZmlsbF9icmV3ZXIocGFsZXR0ZT0iQnVHbiIpKwogIGNvb3JkX2NhcnRlc2lhbih5bGltPWMoMCwxKSkrCiAgdGhlbWUocGFuZWwuYmFja2dyb3VuZCA9IGVsZW1lbnRfYmxhbmsoKSkrIAogIHRoZW1lKHBhbmVsLmdyaWQubWFqb3IgPSBlbGVtZW50X2JsYW5rKCksIHBhbmVsLmdyaWQubWlub3IgPSBlbGVtZW50X2JsYW5rKCksCiAgICAgICAgcGFuZWwuYmFja2dyb3VuZCA9IGVsZW1lbnRfYmxhbmsoKSwgYXhpcy5saW5lID0gZWxlbWVudF9saW5lKGNvbG91ciA9ICJibGFjayIpKSsKICBsYWJzKHg9Ikdsb2JhbCBhbmQgT2NlYW5zIix5PSJSZXNpc3RhbmNlIikrCiAgZ2VvbV90ZXh0KGFlcyggbGFiZWw9c3RhdGxhYix5PVIrUl9zdGQrMC4xKSxwb3NpdGlvbiA9IHBvc2l0aW9uX2RvZGdlKDEpLHZqdXN0ID0wKQoKI2NhcmliYmVhbiBzdWJyZWdpb25zCmNhcmliPC1ibGVhY2htb2RlbCAlPiUKICBmaWx0ZXIoR3JvdXA9PSdDYXJpYicpCmM8LWdncGxvdCggY2FyaWIsIGFlcyh4PWFzLmZhY3RvcihTcGF0aWFsLlNjYWxlKSwgeT1SLCBmaWxsPVNpbXVsYXRpb24pKSArCiAgZ2VvbV9iYXIocG9zaXRpb249cG9zaXRpb25fZG9kZ2UoKSwgc3RhdD0iaWRlbnRpdHkiLGNvbG91cj0nYmxhY2snKSArCiAgZ2VvbV9lcnJvcmJhcihhZXMoeW1pbj1SLVJfc3RkLCB5bWF4PVIrUl9zdGQpLCB3aWR0aD0uMixwb3NpdGlvbj1wb3NpdGlvbl9kb2RnZSguOSkpKwogIHNjYWxlX2ZpbGxfYnJld2VyKHBhbGV0dGU9IkJ1R24iKStjb29yZF9jYXJ0ZXNpYW4oeWxpbT1jKDAsMSkpKwogIHRoZW1lKHBhbmVsLmJhY2tncm91bmQgPSBlbGVtZW50X2JsYW5rKCkpKyAKICB0aGVtZShwYW5lbC5ncmlkLm1ham9yID0gZWxlbWVudF9ibGFuaygpLCBwYW5lbC5ncmlkLm1pbm9yID0gZWxlbWVudF9ibGFuaygpLAogICAgICAgIHBhbmVsLmJhY2tncm91bmQgPSBlbGVtZW50X2JsYW5rKCksIGF4aXMubGluZSA9IGVsZW1lbnRfbGluZShjb2xvdXIgPSAiYmxhY2siKSkrCiAgbGFicyh4PSJDYXJpYmJlYW4gUmVnaW9ucyIseT0iICIpKwogIGdlb21fdGV4dChhZXMoIGxhYmVsPXN0YXRsYWIseT1SK1Jfc3RkKzAuMSkscG9zaXRpb24gPSBwb3NpdGlvbl9kb2RnZSgxKSx2anVzdCA9IDApCgojUGFjaWZpYyBzdWJyZWdpb25zCnBhYzwtYmxlYWNobW9kZWwgJT4lCiAgZmlsdGVyKEdyb3VwPT0nUGFjJykKcDwtZ2dwbG90KCBwYWMsIGFlcyh4PWFzLmZhY3RvcihTcGF0aWFsLlNjYWxlKSwgeT1SLCBmaWxsPVNpbXVsYXRpb24pKSArCiAgZ2VvbV9iYXIocG9zaXRpb249cG9zaXRpb25fZG9kZ2UoKSwgc3RhdD0iaWRlbnRpdHkiLCBjb2xvdXI9J2JsYWNrJykgKwogIGdlb21fZXJyb3JiYXIoYWVzKHltaW49Ui1SX3N0ZCwgeW1heD1SK1Jfc3RkKSwgd2lkdGg9LjIscG9zaXRpb249cG9zaXRpb25fZG9kZ2UoLjkpKSsKICBzY2FsZV9maWxsX2JyZXdlcihwYWxldHRlPSJCdUduIikrY29vcmRfY2FydGVzaWFuKHlsaW09YygwLDEpKSsKICB0aGVtZShwYW5lbC5iYWNrZ3JvdW5kID0gZWxlbWVudF9ibGFuaygpKSsgCiAgdGhlbWUocGFuZWwuZ3JpZC5tYWpvciA9IGVsZW1lbnRfYmxhbmsoKSwgcGFuZWwuZ3JpZC5taW5vciA9IGVsZW1lbnRfYmxhbmsoKSwKICAgICAgICBwYW5lbC5iYWNrZ3JvdW5kID0gZWxlbWVudF9ibGFuaygpLCBheGlzLmxpbmUgPSBlbGVtZW50X2xpbmUoY29sb3VyID0gImJsYWNrIikpKwogIGxhYnMoeD0iUGFjaWZpYyBSZWdpb25zIix5PSIgIikrCiAgZ2VvbV90ZXh0KGFlcyggbGFiZWw9c3RhdGxhYix5PVIrUl9zdGQrMC4xKSxwb3NpdGlvbiA9IHBvc2l0aW9uX2RvZGdlKDEpLHZqdXN0PTApCgojSW5kaWFuIHN1YnJlZ2lvbnMKaW5kPC1ibGVhY2htb2RlbCAlPiUKICBmaWx0ZXIoR3JvdXA9PSdJbmQnKQppPC1nZ3Bsb3QoIGluZCwgYWVzKHg9YXMuZmFjdG9yKFNwYXRpYWwuU2NhbGUpLCB5PVIsIGZpbGw9U2ltdWxhdGlvbikpICsKICBnZW9tX2Jhcihwb3NpdGlvbj1wb3NpdGlvbl9kb2RnZSgpLCBzdGF0PSJpZGVudGl0eSIsIGNvbG91cj0nYmxhY2snKSArCiAgZ2VvbV9lcnJvcmJhcihhZXMoeW1pbj1SLVJfc3RkLCB5bWF4PVIrUl9zdGQpLCB3aWR0aD0uMixwb3NpdGlvbj1wb3NpdGlvbl9kb2RnZSguOSkpKwogIHNjYWxlX2ZpbGxfYnJld2VyKHBhbGV0dGU9IkJ1R24iKStjb29yZF9jYXJ0ZXNpYW4oeWxpbT1jKDAsMSkpK3RoZW1lKHBhbmVsLmJhY2tncm91bmQgPSBlbGVtZW50X2JsYW5rKCkpKyAKICB0aGVtZShwYW5lbC5ncmlkLm1ham9yID0gZWxlbWVudF9ibGFuaygpLCBwYW5lbC5ncmlkLm1pbm9yID0gZWxlbWVudF9ibGFuaygpLAogICAgICAgIHBhbmVsLmJhY2tncm91bmQgPSBlbGVtZW50X2JsYW5rKCksIGF4aXMubGluZSA9IGVsZW1lbnRfbGluZShjb2xvdXIgPSAiYmxhY2siKSkrCiAgbGFicyh4PSJJbmRpYW4gUmVnaW9ucyIseT0iUmVzaXN0YW5jZSIpKwogIGdlb21fdGV4dChhZXMoIGxhYmVsPXN0YXRsYWIseT1SK1Jfc3RkKzAuMSkscG9zaXRpb24gPSBwb3NpdGlvbl9kb2RnZSgxKSx2anVzdCA9IDApCgojUHV0IHRoZW0gYWxsIGluIG9uZSBwbG90CmdnYXJyYW5nZShtYWluLCBwLCBpLCBjLCBuY29sPTIsIG5yb3c9MiwgY29tbW9uLmxlZ2VuZCA9IFRSVUUsIGxlZ2VuZD0iYm90dG9tIikKCmBgYAoKIyMgUm9idXN0bmVzcwoKVGhlIGZvbGxvd2luZyBjb2RlIGNodW5rIGlzIHVzZWQgdG8gbWFrZSBGaWd1cmUgNCBFLUguCgpgYGB7cn0Kcm9idXN0bmVzczwtcmVhZC5jc3YoIlJvYnVzdG5lc3MuY3N2IikgI3Jlc3VsdHMgb2Ygcm9idXN0bmVzcyBhbmFseXNlcywgc2VlIG1ldGFkYXRhIDIgZm9yIG1vcmUgaW5mbwpzdW1tYXJ5KHJvYnVzdG5lc3MpCiNnZXQgcmlkIG9mIHRoZSByZW1vdmFsIG1vZGVscyB0aGF0IHdlcmUgdGVzdGVkIGJ1dCBub3QgdXNlZApkYXRhMjwtcm9idXN0bmVzcyAlPiUKICAgIGZpbHRlcihtb2RlbCE9IkxUX0JIIiwgbW9kZWwhPSJMVF9ISCIsbW9kZWwhPSJMVF9TSCIsbW9kZWwhPSJUb2xlcmFuY2VfaGlnaCIpCiAgCkdsb2JhbF90b3Q8LWRhdGEyJT4lCiAgZmlsdGVyKG5ldHdvcms9PSJHIixyZW1vdmVkIT0iaG9zdHMiLHJlbW92ZWQhPSJzeW1iaW9udHMiLFI1MF93aG89PSJib3RoIikKR2xvYmFsX3RvdCRyZW1vdmVkPC1mYWN0b3IoR2xvYmFsX3RvdCRyZW1vdmVkLGxldmVscz1jKCJsaW5rcyI9ImxpbmtzIiwibm9kZXMiPSJib3RoIikpCiAgCmdsb2JhbDwtZ2dwbG90KCBHbG9iYWxfdG90LCBhZXMoeD1hcy5mYWN0b3IobW9kZWwpLCB5PW1lYW4sZmlsbD1yZW1vdmVkKSkKZ190b3RhbDwtZ2xvYmFsKwogIGdlb21fYmFyKHBvc2l0aW9uPXBvc2l0aW9uX2RvZGdlKCksc3RhdD0iaWRlbnRpdHkiLGNvbG91cj0nYmxhY2snLG1hcHBpbmc9YWVzKGNvbD0icmVkIikpICsKICBnZW9tX2Vycm9yYmFyKGFlcyh5bWluPW1lYW4tc3RkLCB5bWF4PW1lYW4rc3RkKSwgd2lkdGg9LjIscG9zaXRpb249cG9zaXRpb25fZG9kZ2UoLjkpKSsKICBzY2FsZV9maWxsX21hbnVhbCh2YWx1ZXM9YygibGlua3MiID0gIiM3ZmM5N2YiLCAiYm90aCIgPSAiIzk5ZDhjOSIsICJob3N0cyIgPSAiI2E2Y2VlMyIsInN5bWJpb250cyI9IiNmZmZmOTkiKSwgZHJvcCA9IEZBTFNFKSsKICAjeWxpbSgwLDEpKwogIHNjYWxlX3lfY29udGludW91cyhsaW1pdHM9YygwLDEuMDUpLGV4cGFuZCA9IGMoMCwwKSxicmVha3M9c2VxKDAsIDEsIDAuMSkpKwogIHRoZW1lKHBhbmVsLmJhY2tncm91bmQgPSBlbGVtZW50X2JsYW5rKCkpKyAKICB0aGVtZShwYW5lbC5ncmlkLm1ham9yID0gZWxlbWVudF9ibGFuaygpLCBwYW5lbC5ncmlkLm1pbm9yID0gZWxlbWVudF9ibGFuaygpLHBhbmVsLmJhY2tncm91bmQgPSBlbGVtZW50X2JsYW5rKCksIGF4aXMubGluZSA9IGVsZW1lbnRfbGluZShjb2xvdXIgPSAiYmxhY2siKSkrCiAgbGFicyh4PSIiLHk9IlJvYnVzdG5lc3MsIFI1MCIpKwogIHRoZW1lKGxlZ2VuZC5wb3NpdGlvbj0ibm9uZSIsYXhpcy50ZXh0LnggPSBlbGVtZW50X3RleHQoYW5nbGUgPSA3MCwgaGp1c3QgPSAxKSkrCiAgc2NhbGVfeF9kaXNjcmV0ZShsaW1pdHM9YygiUmFuZG9tX2xpbmsiLCJibGVhY2giLCJMVF9CTCIsIkxUX0hMIiwiTFRfU0wiLCJSYW5kb21fbm9kZSIsIlRvbGVyYW5jZV9sb3ciLCJEZWdyZWVfbG93IiwiRGVncmVlX2hpZ2giKSxsYWJlbHM9YygiUmFuZG9tX2xpbmsiPSJSYW5kb20iLCJibGVhY2giPSJCbGVhY2hpbmciLCJUb2xlcmFuY2VfbG93Ij0iU3VzY2VwdGlibGUiLCJUb2xlcmFuY2VfaGlnaCI9IkhpZ2ggVG9sZXJhbmNlIiwiRGVncmVlX2hpZ2giPSAiSGlnaCBEZWdyZWUiLCJEZWdyZWVfbG93Ij0iTG93IERlZ3JlZSIsIlJhbmRvbV9ub2RlIj0iUmFuZG9tIE5vZGUiLCJMVF9CSCI9IkhpZ2ggQXZnLiBMaW5rIFRvbGVyYW5jZSIsIkxUX0JMIj0iU3VzY2VwdGlibGUiLCJMVF9ISCI9IkhpZ2ggSG9zdCBMaW5rIFRvbGVyYW5jZSIsIkxUX0hMIj0iSG9zdCBTdXNjZXB0aWJsZSIsIkxUX1NIIj0iSGlnaCBTeW1iaW9udCBMaW5rIFRvbGVyYW5jZSIsIkxUX1NMIj0iU3ltYmlvbnQgU3VzY2VwdGlibGUiKSkrCiAgZ2VvbV90ZXh0KGFlcyggbGFiZWw9c3RhdGxhYix5PW1lYW4rc3RkKzAuMDUpLHBvc2l0aW9uID0gcG9zaXRpb25fZG9kZ2UoMC45KSx2anVzdCA9IDApCgpQYWNpZmljX3RvdDwtZGF0YTIlPiUKICBmaWx0ZXIobmV0d29yaz09IlAiLHJlbW92ZWQhPSJob3N0cyIscmVtb3ZlZCE9InN5bWJpb250cyIsUjUwX3dobz09ImJvdGgiKQpQYWNpZmljX3RvdCRyZW1vdmVkPC1mYWN0b3IoUGFjaWZpY190b3QkcmVtb3ZlZCxsZXZlbHM9YygibGlua3MiPSJsaW5rcyIsIm5vZGVzIj0iYm90aCIpKQogIApQYWNpZmljPC1nZ3Bsb3QoIFBhY2lmaWNfdG90LCBhZXMoeD1hcy5mYWN0b3IobW9kZWwpLCB5PW1lYW4sZmlsbD1yZW1vdmVkKSkKcF90b3RhbDwtUGFjaWZpYysKICBnZW9tX2Jhcihwb3NpdGlvbj1wb3NpdGlvbl9kb2RnZSgpLHN0YXQ9ImlkZW50aXR5Iixjb2xvdXI9J2JsYWNrJyxtYXBwaW5nPWFlcyhjb2w9InJlZCIpKSArCiAgZ2VvbV9lcnJvcmJhcihhZXMoeW1pbj1tZWFuLXN0ZCwgeW1heD1tZWFuK3N0ZCksIHdpZHRoPS4yLHBvc2l0aW9uPXBvc2l0aW9uX2RvZGdlKC45KSkrCiAgc2NhbGVfZmlsbF9tYW51YWwodmFsdWVzPWMoImxpbmtzIiA9ICIjN2ZjOTdmIiwgImJvdGgiID0gIiM5OWQ4YzkiLCAiaG9zdHMiID0gIiNhNmNlZTMiLCJzeW1iaW9udHMiPSIjZmZmZjk5IiksIGRyb3AgPSBGQUxTRSkrCiAgI3lsaW0oMCwxKSsKICBzY2FsZV95X2NvbnRpbnVvdXMobGltaXRzPWMoMCwxLjA1KSxleHBhbmQgPSBjKDAsMCksYnJlYWtzPXNlcSgwLCAxLCAwLjEpKSsKICB0aGVtZShwYW5lbC5iYWNrZ3JvdW5kID0gZWxlbWVudF9ibGFuaygpKSsgCiAgdGhlbWUocGFuZWwuZ3JpZC5tYWpvciA9IGVsZW1lbnRfYmxhbmsoKSwgcGFuZWwuZ3JpZC5taW5vciA9IGVsZW1lbnRfYmxhbmsoKSxwYW5lbC5iYWNrZ3JvdW5kID0gZWxlbWVudF9ibGFuaygpLCBheGlzLmxpbmUgPSBlbGVtZW50X2xpbmUoY29sb3VyID0gImJsYWNrIikpKwogIGxhYnMoeD0iIix5PSIiKSsKICB0aGVtZShsZWdlbmQucG9zaXRpb249Im5vbmUiLGF4aXMudGV4dC54ID0gZWxlbWVudF90ZXh0KGFuZ2xlID0gNzAsIGhqdXN0ID0gMSkpKwogIHNjYWxlX3hfZGlzY3JldGUobGltaXRzPWMoIlJhbmRvbV9saW5rIiwiYmxlYWNoIiwiTFRfQkwiLCJMVF9ITCIsIkxUX1NMIiwiUmFuZG9tX25vZGUiLCJUb2xlcmFuY2VfbG93IiwiRGVncmVlX2xvdyIsIkRlZ3JlZV9oaWdoIiksbGFiZWxzPWMoIlJhbmRvbV9saW5rIj0iUmFuZG9tIiwiYmxlYWNoIj0iQmxlYWNoaW5nIiwiVG9sZXJhbmNlX2xvdyI9IlN1c2NlcHRpYmxlIiwiVG9sZXJhbmNlX2hpZ2giPSJIaWdoIFRvbGVyYW5jZSIsIkRlZ3JlZV9oaWdoIj0gIkhpZ2ggRGVncmVlIiwiRGVncmVlX2xvdyI9IkxvdyBEZWdyZWUiLCJSYW5kb21fbm9kZSI9IlJhbmRvbSBOb2RlIiwiTFRfQkgiPSJIaWdoIEF2Zy4gTGluayBUb2xlcmFuY2UiLCJMVF9CTCI9IlN1c2NlcHRpYmxlIiwiTFRfSEgiPSJIaWdoIEhvc3QgTGluayBUb2xlcmFuY2UiLCJMVF9ITCI9Ikhvc3QgU3VzY2VwdGlibGUiLCJMVF9TSCI9IkhpZ2ggU3ltYmlvbnQgTGluayBUb2xlcmFuY2UiLCJMVF9TTCI9IlN5bWJpb250IFN1c2NlcHRpYmxlIikpKwogIGdlb21fdGV4dChhZXMoIGxhYmVsPXN0YXRsYWIseT1tZWFuK3N0ZCswLjA1KSxwb3NpdGlvbiA9IHBvc2l0aW9uX2RvZGdlKDAuOSksdmp1c3QgPSAwKQoKQ2FyaWJfdG90PC1kYXRhMiU+JQogIGZpbHRlcihuZXR3b3JrPT0iQyIscmVtb3ZlZCE9Imhvc3RzIixyZW1vdmVkIT0ic3ltYmlvbnRzIixSNTBfd2hvPT0iYm90aCIpCkNhcmliX3RvdCRyZW1vdmVkPC1mYWN0b3IoQ2FyaWJfdG90JHJlbW92ZWQsbGV2ZWxzPWMoImxpbmtzIj0ibGlua3MiLCJub2RlcyI9ImJvdGgiKSkKCkNhcmliPC1nZ3Bsb3QoIENhcmliX3RvdCwgYWVzKHg9YXMuZmFjdG9yKG1vZGVsKSwgeT1tZWFuLGZpbGw9cmVtb3ZlZCkpCmNfdG90YWw8LUNhcmliKwogIGdlb21fYmFyKHBvc2l0aW9uPXBvc2l0aW9uX2RvZGdlKCksc3RhdD0iaWRlbnRpdHkiLGNvbG91cj0nYmxhY2snLG1hcHBpbmc9YWVzKGNvbD0icmVkIikpICsKICBnZW9tX2Vycm9yYmFyKGFlcyh5bWluPW1lYW4tc3RkLCB5bWF4PW1lYW4rc3RkKSwgd2lkdGg9LjIscG9zaXRpb249cG9zaXRpb25fZG9kZ2UoLjkpKSsKICBzY2FsZV9maWxsX21hbnVhbCh2YWx1ZXM9YygibGlua3MiID0gIiM3ZmM5N2YiLCAiYm90aCIgPSAiIzk5ZDhjOSIsICJob3N0cyIgPSAiI2E2Y2VlMyIsInN5bWJpb250cyI9IiNmZmZmOTkiKSwgZHJvcCA9IEZBTFNFKSsKICAjeWxpbSgwLDEpKwogIHNjYWxlX3lfY29udGludW91cyhsaW1pdHM9YygwLDEuMDUpLGV4cGFuZCA9IGMoMCwwKSxicmVha3M9c2VxKDAsIDEsIDAuMSkpKwogIHRoZW1lKHBhbmVsLmJhY2tncm91bmQgPSBlbGVtZW50X2JsYW5rKCkpKyAKICB0aGVtZShwYW5lbC5ncmlkLm1ham9yID0gZWxlbWVudF9ibGFuaygpLCBwYW5lbC5ncmlkLm1pbm9yID0gZWxlbWVudF9ibGFuaygpLHBhbmVsLmJhY2tncm91bmQgPSBlbGVtZW50X2JsYW5rKCksIGF4aXMubGluZSA9IGVsZW1lbnRfbGluZShjb2xvdXIgPSAiYmxhY2siKSkrCiAgbGFicyh4PSIiLHk9IiIpKwogIHRoZW1lKGxlZ2VuZC5wb3NpdGlvbj0ibm9uZSIsYXhpcy50ZXh0LnggPSBlbGVtZW50X3RleHQoYW5nbGUgPSA3MCwgaGp1c3QgPSAxKSkrCiAgc2NhbGVfeF9kaXNjcmV0ZShsaW1pdHM9YygiUmFuZG9tX2xpbmsiLCJibGVhY2giLCJMVF9CTCIsIkxUX0hMIiwiTFRfU0wiLCJSYW5kb21fbm9kZSIsIlRvbGVyYW5jZV9sb3ciLCJEZWdyZWVfbG93IiwiRGVncmVlX2hpZ2giKSxsYWJlbHM9YygiUmFuZG9tX2xpbmsiPSJSYW5kb20iLCJibGVhY2giPSJCbGVhY2hpbmciLCJUb2xlcmFuY2VfbG93Ij0iU3VzY2VwdGlibGUiLCJUb2xlcmFuY2VfaGlnaCI9IkhpZ2ggVG9sZXJhbmNlIiwiRGVncmVlX2hpZ2giPSAiSGlnaCBEZWdyZWUiLCJEZWdyZWVfbG93Ij0iTG93IERlZ3JlZSIsIlJhbmRvbV9ub2RlIj0iUmFuZG9tIE5vZGUiLCJMVF9CSCI9IkhpZ2ggQXZnLiBMaW5rIFRvbGVyYW5jZSIsIkxUX0JMIj0iU3VzY2VwdGlibGUiLCJMVF9ISCI9IkhpZ2ggSG9zdCBMaW5rIFRvbGVyYW5jZSIsIkxUX0hMIj0iSG9zdCBTdXNjZXB0aWJsZSIsIkxUX1NIIj0iSGlnaCBTeW1iaW9udCBMaW5rIFRvbGVyYW5jZSIsIkxUX1NMIj0iU3ltYmlvbnQgU3VzY2VwdGlibGUiKSkrCiAgZ2VvbV90ZXh0KGFlcyggbGFiZWw9c3RhdGxhYix5PW1lYW4rc3RkKzAuMDUpLHBvc2l0aW9uID0gcG9zaXRpb25fZG9kZ2UoMC45KSx2anVzdCA9IDApCgpJbmRfdG90PC1kYXRhMiU+JQogIGZpbHRlcihuZXR3b3JrPT0iSSIscmVtb3ZlZCE9Imhvc3RzIixyZW1vdmVkIT0ic3ltYmlvbnRzIixSNTBfd2hvPT0iYm90aCIpCkluZF90b3QkcmVtb3ZlZDwtZmFjdG9yKENhcmliX3RvdCRyZW1vdmVkLGxldmVscz1jKCJsaW5rcyI9ImxpbmtzIiwibm9kZXMiPSJib3RoIikpCgpJbmQ8LWdncGxvdCggSW5kX3RvdCwgYWVzKHg9YXMuZmFjdG9yKG1vZGVsKSwgeT1tZWFuLGZpbGw9cmVtb3ZlZCkpCmlfdG90YWw8LUluZCsKICBnZW9tX2Jhcihwb3NpdGlvbj1wb3NpdGlvbl9kb2RnZSgpLHN0YXQ9ImlkZW50aXR5Iixjb2xvdXI9J2JsYWNrJyxtYXBwaW5nPWFlcyhjb2w9InJlZCIpKSArCiAgZ2VvbV9lcnJvcmJhcihhZXMoeW1pbj1tZWFuLXN0ZCwgeW1heD1tZWFuK3N0ZCksIHdpZHRoPS4yLHBvc2l0aW9uPXBvc2l0aW9uX2RvZGdlKC45KSkrCiAgc2NhbGVfZmlsbF9tYW51YWwodmFsdWVzPWMoImxpbmtzIiA9ICIjN2ZjOTdmIiwgImJvdGgiID0gIiM5OWQ4YzkiLCAiaG9zdHMiID0gIiNhNmNlZTMiLCJzeW1iaW9udHMiPSIjZmZmZjk5IiksIGRyb3AgPSBGQUxTRSkrCiAgI3lsaW0oMCwxKSsKICBzY2FsZV95X2NvbnRpbnVvdXMobGltaXRzPWMoMCwxLjA1KSxleHBhbmQgPSBjKDAsMCksYnJlYWtzPXNlcSgwLCAxLCAwLjEpKSsKICB0aGVtZShwYW5lbC5iYWNrZ3JvdW5kID0gZWxlbWVudF9ibGFuaygpKSsgCiAgdGhlbWUocGFuZWwuZ3JpZC5tYWpvciA9IGVsZW1lbnRfYmxhbmsoKSwgcGFuZWwuZ3JpZC5taW5vciA9IGVsZW1lbnRfYmxhbmsoKSxwYW5lbC5iYWNrZ3JvdW5kID0gZWxlbWVudF9ibGFuaygpLCBheGlzLmxpbmUgPSBlbGVtZW50X2xpbmUoY29sb3VyID0gImJsYWNrIikpKwogIGxhYnMoeD0iIix5PSIiKSsKICB0aGVtZShsZWdlbmQucG9zaXRpb249Im5vbmUiLGF4aXMudGV4dC54ID0gZWxlbWVudF90ZXh0KGFuZ2xlID0gNzAsIGhqdXN0ID0gMSkpKwogIHNjYWxlX3hfZGlzY3JldGUobGltaXRzPWMoIlJhbmRvbV9saW5rIiwiYmxlYWNoIiwiTFRfQkwiLCJMVF9ITCIsIkxUX1NMIiwiUmFuZG9tX25vZGUiLCJUb2xlcmFuY2VfbG93IiwiRGVncmVlX2xvdyIsIkRlZ3JlZV9oaWdoIiksbGFiZWxzPWMoIlJhbmRvbV9saW5rIj0iUmFuZG9tIiwiYmxlYWNoIj0iQmxlYWNoaW5nIiwiVG9sZXJhbmNlX2xvdyI9IlN1c2NlcHRpYmxlIiwiVG9sZXJhbmNlX2hpZ2giPSJIaWdoIFRvbGVyYW5jZSIsIkRlZ3JlZV9oaWdoIj0gIkhpZ2ggRGVncmVlIiwiRGVncmVlX2xvdyI9IkxvdyBEZWdyZWUiLCJSYW5kb21fbm9kZSI9IlJhbmRvbSBOb2RlIiwiTFRfQkgiPSJIaWdoIEF2Zy4gTGluayBUb2xlcmFuY2UiLCJMVF9CTCI9IlN1c2NlcHRpYmxlIiwiTFRfSEgiPSJIaWdoIEhvc3QgTGluayBUb2xlcmFuY2UiLCJMVF9ITCI9Ikhvc3QgU3VzY2VwdGlibGUiLCJMVF9TSCI9IkhpZ2ggU3ltYmlvbnQgTGluayBUb2xlcmFuY2UiLCJMVF9TTCI9IlN5bWJpb250IFN1c2NlcHRpYmxlIikpKwogIGdlb21fdGV4dChhZXMoIGxhYmVsPXN0YXRsYWIseT1tZWFuK3N0ZCswLjA1KSxwb3NpdGlvbiA9IHBvc2l0aW9uX2RvZGdlKDAuOSksdmp1c3QgPSAwKQoKI1B1dCB0aGVtIGFsbCBpbiBvbmUgcGxvdApnZ2FycmFuZ2UoZ190b3RhbCxwX3RvdGFsLGlfdG90YWwsY190b3RhbCwgbmNvbD00LCBucm93PTEsIGNvbW1vbi5sZWdlbmQgPSBGQUxTRSwgbGVnZW5kPU5VTEwpCmBgYAoKVGhlIGZvbGxvd2luZyBjb2RlIGNodW5rIGlzIHVzZWQgdG8gbWFrZSBBcHBlbmRpeCBTMiwgRmlndXJlIFMxIEQtRi4KCmBgYHtyfQpteXBsb3Q8LWZ1bmN0aW9uKGRhdGEyLE4peyAjZGF0YTI9cm9idXN0bmVzcyByZXN1bHRzLCBOPW5ldHdvcmsgdG8gcGxvdAogIGRhdGEyPC1kYXRhMiAlPiUKICAgIGZpbHRlcihtb2RlbCE9IkxUX0JIIiwgbW9kZWwhPSJMVF9ISCIsbW9kZWwhPSJMVF9TSCIsbW9kZWwhPSJUb2xlcmFuY2VfaGlnaCIpCiAgCiAgR2xvYmFsX3RvdDwtZGF0YTIlPiUKICAgIGZpbHRlcihuZXR3b3JrPT1OLHJlbW92ZWQhPSJob3N0cyIscmVtb3ZlZCE9InN5bWJpb250cyIsUjUwX3dobz09ImJvdGgiKQogIEdsb2JhbF90b3QkcmVtb3ZlZDwtZmFjdG9yKEdsb2JhbF90b3QkcmVtb3ZlZCxsZXZlbHM9YygibGlua3MiLCJib3RoIiwiaG9zdHMiLCJzeW1iaW9udHMiKSkKICAKICBnbG9iYWw8LWdncGxvdCggR2xvYmFsX3RvdCwgYWVzKHg9YXMuZmFjdG9yKG1vZGVsKSwgeT1tZWFuLGZpbGw9cmVtb3ZlZCkpCiAgZ190b3RhbDwtZ2xvYmFsKwogICAgZ2VvbV9iYXIocG9zaXRpb249cG9zaXRpb25fZG9kZ2UoKSxzdGF0PSJpZGVudGl0eSIsY29sb3VyPSdibGFjaycsbWFwcGluZz1hZXMoY29sPSJyZWQiKSkgKwogICAgZ2VvbV9lcnJvcmJhcihhZXMoeW1pbj1tZWFuLXN0ZCwgeW1heD1tZWFuK3N0ZCksIHdpZHRoPS4yLHBvc2l0aW9uPXBvc2l0aW9uX2RvZGdlKC45KSkrCiAgICBzY2FsZV9maWxsX21hbnVhbCh2YWx1ZXM9YygibGlua3MiID0gIiM3ZmM5N2YiLCAiYm90aCIgPSAiIzk5ZDhjOSIsICJob3N0cyIgPSAiI2E2Y2VlMyIsInN5bWJpb250cyI9IiNmZmZmOTkiKSwgCiAgICAgICAgICAgICAgICAgICAgICBkcm9wID0gRkFMU0UpKwogICAgCiAgICAjeWxpbSgwLDEpKwogICAgc2NhbGVfeV9jb250aW51b3VzKGxpbWl0cz1jKDAsMS4wNSksZXhwYW5kID0gYygwLDApLGJyZWFrcz1zZXEoMCwgMSwgMC4xKSkrCiAgICB0aGVtZShwYW5lbC5iYWNrZ3JvdW5kID0gZWxlbWVudF9ibGFuaygpKSsgCiAgICB0aGVtZShwYW5lbC5ncmlkLm1ham9yID0gZWxlbWVudF9ibGFuaygpLCBwYW5lbC5ncmlkLm1pbm9yID0gZWxlbWVudF9ibGFuaygpLAogICAgICAgICAgcGFuZWwuYmFja2dyb3VuZCA9IGVsZW1lbnRfYmxhbmsoKSwgYXhpcy5saW5lID0gZWxlbWVudF9saW5lKGNvbG91ciA9ICJibGFjayIpKSsKICAgIGxhYnMoeD0iIix5PSJUb3RhbCBSb2J1c3RuZXNzIikrCiAgICB0aGVtZShheGlzLnRleHQueCA9IGVsZW1lbnRfdGV4dChhbmdsZSA9IDcwLCBoanVzdCA9IDEpKSsKICAgIHNjYWxlX3hfZGlzY3JldGUobGltaXRzPWMoIlJhbmRvbV9saW5rIiwiYmxlYWNoIiwiTFRfQkwiLCJMVF9ITCIsIkxUX1NMIiwiUmFuZG9tX25vZGUiLCJUb2xlcmFuY2VfbG93IiwiRGVncmVlX2xvdyIsIkRlZ3JlZV9oaWdoIiksCiAgICAgICAgICAgICAgICAgICAgIGxhYmVscz1jKCJSYW5kb21fbGluayI9IlJhbmRvbSIsImJsZWFjaCI9IkJsZWFjaGluZyIsIlRvbGVyYW5jZV9sb3ciPSJTdXNjZXB0aWJsZSIsIlRvbGVyYW5jZV9oaWdoIj0iSGlnaCBUb2xlcmFuY2UiLCJEZWdyZWVfaGlnaCI9ICJIaWdoIERlZ3JlZSIsIkRlZ3JlZV9sb3ciPSJMb3cgRGVncmVlIiwiUmFuZG9tX25vZGUiPSJSYW5kb20gTm9kZSIsIkxUX0JIIj0iSGlnaCBBdmcuIExpbmsgVG9sZXJhbmNlIiwiTFRfQkwiPSJTdXNjZXB0aWJsZSIsIkxUX0hIIj0iSGlnaCBIb3N0IExpbmsgVG9sZXJhbmNlIiwiTFRfSEwiPSJIb3N0IFN1c2NlcHRpYmxlIiwiTFRfU0giPSJIaWdoIFN5bWJpb250IExpbmsgVG9sZXJhbmNlIiwiTFRfU0wiPSJTeW1iaW9udCBTdXNjZXB0aWJsZSIpKSsKICAgIGdlb21fdGV4dChhZXMoIGxhYmVsPXN0YXRsYWIseT1tZWFuK3N0ZCswLjA1KSxwb3NpdGlvbiA9IHBvc2l0aW9uX2RvZGdlKDAuOSksdmp1c3QgPSAwKQogIAogIEdsb2JhbF9oPC1kYXRhMiU+JQogICAgZmlsdGVyKG5ldHdvcms9PU4scmVtb3ZlZCE9Imhvc3RzIixyZW1vdmVkIT0iYm90aCIsUjUwX3dobz09Imhvc3RzIikKICBHbG9iYWxfaCRyZW1vdmVkPC1mYWN0b3IoR2xvYmFsX2gkcmVtb3ZlZCxsZXZlbHM9YygibGlua3MiLCJib3RoIiwiaG9zdHMiLCJzeW1iaW9udHMiKSkKICBnbG9iYWxfaDwtZ2dwbG90KCBHbG9iYWxfaCwgYWVzKHg9YXMuZmFjdG9yKG1vZGVsKSwgeT1tZWFuLGZpbGw9cmVtb3ZlZCkpCiAgZ19ob3N0czwtZ2xvYmFsX2grCiAgICBnZW9tX2Jhcihwb3NpdGlvbj1wb3NpdGlvbl9kb2RnZSgpLHN0YXQ9ImlkZW50aXR5Iixjb2xvdXI9J2JsYWNrJyxtYXBwaW5nPWFlcyhjb2w9InJlZCIpKSArCiAgICBnZW9tX2Vycm9yYmFyKGFlcyh5bWluPW1lYW4tc3RkLCB5bWF4PW1lYW4rc3RkKSwgd2lkdGg9LjIscG9zaXRpb249cG9zaXRpb25fZG9kZ2UoLjkpKSsKICAgIHNjYWxlX2ZpbGxfbWFudWFsKHZhbHVlcz1jKCJsaW5rcyIgPSAiIzdmYzk3ZiIsICJib3RoIiA9ICIjOTlkOGM5IiwgImhvc3RzIiA9ICIjYTZjZWUzIiwic3ltYmlvbnRzIj0iI2ZmZmY5OSIpLCAKICAgICAgICAgICAgICAgICAgICAgIGRyb3AgPSBGQUxTRSkrCiAgICBzY2FsZV95X2NvbnRpbnVvdXMobGltaXRzPWMoMCwxLjA1KSxleHBhbmQgPSBjKDAsMCksYnJlYWtzPXNlcSgwLCAxLCAwLjEpKSsKICAgIHRoZW1lKHBhbmVsLmJhY2tncm91bmQgPSBlbGVtZW50X2JsYW5rKCkpKyAKICAgIHRoZW1lKHBhbmVsLmdyaWQubWFqb3IgPSBlbGVtZW50X2JsYW5rKCksIHBhbmVsLmdyaWQubWlub3IgPSBlbGVtZW50X2JsYW5rKCksCiAgICAgICAgICBwYW5lbC5iYWNrZ3JvdW5kID0gZWxlbWVudF9ibGFuaygpLCBheGlzLmxpbmUgPSBlbGVtZW50X2xpbmUoY29sb3VyID0gImJsYWNrIikpKwogICAgbGFicyh4PSIiLHk9Ikhvc3QgUm9idXN0bmVzcyIpKwogICAgdGhlbWUoYXhpcy50ZXh0LnggPSBlbGVtZW50X3RleHQoYW5nbGUgPSA3MCwgaGp1c3QgPSAxKSkrCiAgICBzY2FsZV94X2Rpc2NyZXRlKGxpbWl0cz1jKCJSYW5kb21fbGluayIsImJsZWFjaCIsIkxUX0JMIiwiTFRfSEwiLCJMVF9TTCIsIlJhbmRvbV9ub2RlIiwiVG9sZXJhbmNlX2xvdyIsIkRlZ3JlZV9sb3ciLCJEZWdyZWVfaGlnaCIpLAogICAgICAgICAgICAgICAgICAgICBsYWJlbHM9YygiUmFuZG9tX2xpbmsiPSJSYW5kb20iLCJibGVhY2giPSJCbGVhY2hpbmciLCJUb2xlcmFuY2VfbG93Ij0iU3VzY2VwdGlibGUiLCJUb2xlcmFuY2VfaGlnaCI9IkhpZ2ggVG9sZXJhbmNlIiwiRGVncmVlX2hpZ2giPSAiSGlnaCBEZWdyZWUiLCJEZWdyZWVfbG93Ij0iTG93IERlZ3JlZSIsIlJhbmRvbV9ub2RlIj0iUmFuZG9tIE5vZGUiLCJMVF9CSCI9IkhpZ2ggQXZnLiBMaW5rIFRvbGVyYW5jZSIsIkxUX0JMIj0iU3VzY2VwdGlibGUiLCJMVF9ISCI9IkhpZ2ggSG9zdCBMaW5rIFRvbGVyYW5jZSIsIkxUX0hMIj0iSG9zdCBTdXNjZXB0aWJsZSIsIkxUX1NIIj0iSGlnaCBTeW1iaW9udCBMaW5rIFRvbGVyYW5jZSIsIkxUX1NMIj0iU3ltYmlvbnQgU3VzY2VwdGlibGUiKSkrCiAgICBnZW9tX3RleHQoYWVzKCBsYWJlbD1zdGF0bGFiLHk9bWVhbitzdGQrMC4wMykscG9zaXRpb24gPSBwb3NpdGlvbl9kb2RnZSgwLjkpLHZqdXN0ID0wKQogIAogIEdsb2JhbF9zPC1kYXRhMiU+JQogICAgZmlsdGVyKG5ldHdvcms9PU4scmVtb3ZlZCE9InN5bWJpb250cyIscmVtb3ZlZCE9ImJvdGgiLFI1MF93aG89PSJzeW1icyIpCiAgR2xvYmFsX3MkcmVtb3ZlZDwtZmFjdG9yKEdsb2JhbF9zJHJlbW92ZWQsbGV2ZWxzPWMoImxpbmtzIiwiYm90aCIsImhvc3RzIiwic3ltYmlvbnRzIikpCiAgZ2xvYmFsX3M8LWdncGxvdCggR2xvYmFsX3MsIGFlcyh4PWFzLmZhY3Rvcihtb2RlbCksIHk9bWVhbixmaWxsPXJlbW92ZWQpKQogIGdfc3ltYnM8LWdsb2JhbF9zKwogICAgZ2VvbV9iYXIocG9zaXRpb249cG9zaXRpb25fZG9kZ2UoKSxzdGF0PSJpZGVudGl0eSIsY29sb3VyPSdibGFjaycsbWFwcGluZz1hZXMoY29sPSJyZWQiKSkgKwogICAgZ2VvbV9lcnJvcmJhcihhZXMoeW1pbj1tZWFuLXN0ZCwgeW1heD1tZWFuK3N0ZCksIHdpZHRoPS4yLHBvc2l0aW9uPXBvc2l0aW9uX2RvZGdlKC45KSkrCiAgICBzY2FsZV9maWxsX21hbnVhbCh2YWx1ZXM9YygibGlua3MiID0gIiM3ZmM5N2YiLCAiYm90aCIgPSAiIzk5ZDhjOSIsICJob3N0cyIgPSAiI2E2Y2VlMyIsInN5bWJpb250cyI9IiNmZmZmOTkiKSwgCiAgICAgICAgICAgICAgICAgICAgICBkcm9wID0gRkFMU0UpKwogICAgc2NhbGVfeV9jb250aW51b3VzKGxpbWl0cz1jKDAsMS4wNSksZXhwYW5kID0gYygwLDApLGJyZWFrcz1zZXEoMCwgMSwgMC4xKSkrCiAgICB0aGVtZShwYW5lbC5iYWNrZ3JvdW5kID0gZWxlbWVudF9ibGFuaygpKSsgCiAgICB0aGVtZShwYW5lbC5ncmlkLm1ham9yID0gZWxlbWVudF9ibGFuaygpLCBwYW5lbC5ncmlkLm1pbm9yID0gZWxlbWVudF9ibGFuaygpLAogICAgICAgICAgcGFuZWwuYmFja2dyb3VuZCA9IGVsZW1lbnRfYmxhbmsoKSwgYXhpcy5saW5lID0gZWxlbWVudF9saW5lKGNvbG91ciA9ICJibGFjayIpKSsKICAgIGxhYnMoeD0iIix5PSJTeW1iaW9udCBSb2J1c3RuZXNzIikrCiAgICB0aGVtZShheGlzLnRleHQueCA9IGVsZW1lbnRfdGV4dChhbmdsZSA9IDcwLCBoanVzdCA9IDEpKSsKICAgIHNjYWxlX3hfZGlzY3JldGUobGltaXRzPWMoIlJhbmRvbV9saW5rIiwiYmxlYWNoIiwiTFRfQkwiLCJMVF9ITCIsIkxUX1NMIiwiUmFuZG9tX25vZGUiLCJUb2xlcmFuY2VfbG93IiwiRGVncmVlX2xvdyIsIkRlZ3JlZV9oaWdoIiksCiAgICAgICAgICAgICAgICAgICAgIGxhYmVscz1jKCJSYW5kb21fbGluayI9IlJhbmRvbSIsImJsZWFjaCI9IkJsZWFjaGluZyIsIlRvbGVyYW5jZV9sb3ciPSJTdXNjZXB0aWJsZSIsIlRvbGVyYW5jZV9oaWdoIj0iSGlnaCBUb2xlcmFuY2UiLCJEZWdyZWVfaGlnaCI9ICJIaWdoIERlZ3JlZSIsIkRlZ3JlZV9sb3ciPSJMb3cgRGVncmVlIiwiUmFuZG9tX25vZGUiPSJSYW5kb20gTm9kZSIsIkxUX0JIIj0iSGlnaCBBdmcuIExpbmsgVG9sZXJhbmNlIiwiTFRfQkwiPSJTdXNjZXB0aWJsZSIsIkxUX0hIIj0iSGlnaCBIb3N0IExpbmsgVG9sZXJhbmNlIiwiTFRfSEwiPSJIb3N0IFN1c2NlcHRpYmxlIiwiTFRfU0giPSJIaWdoIFN5bWJpb250IExpbmsgVG9sZXJhbmNlIiwiTFRfU0wiPSJTeW1iaW9udCBTdXNjZXB0aWJsZSIpKSsKICAgIGdlb21fdGV4dChhZXMoIGxhYmVsPXN0YXRsYWIseT1tZWFuK3N0ZCswLjAzKSxwb3NpdGlvbiA9IHBvc2l0aW9uX2RvZGdlKDAuOSksdmp1c3QgPTApCiAgCiAgdGhlbWVfc2V0KHRoZW1lX2dyZXkoYmFzZV9zaXplID0gMTQpKSAKICBwPC1nZ2FycmFuZ2UoZ190b3RhbCxnX2hvc3RzLGdfc3ltYnMsIG5jb2w9MywgbnJvdz0xLCBjb21tb24ubGVnZW5kID0gVFJVRSwgbGVnZW5kPSJib3R0b20iKQogIHJldHVybihwKQp9CgpteXBsb3Qocm9idXN0bmVzcywiRyIpCgpgYGAKCiMjIFJhbmRvbWl6YXRpb24gVGVzdHMKClNlZSBBcHBlbmRpeCBTMiBmb3IgZGVzY3JpcHRpb24uIFJhbmRvbWl6YXRpb24gdGVzdHMgYXJlIG9mdGVuIGNhbGxlZCBwZXJtdXRhdGlvbiB0ZXN0cywgdGh1cyBJIHVzZSAicGVybXV0YXRpb24iIHdpdGhpbiB0aGUgY29kZS4KCmBgYHtyfQoKbXlwZXJtdXRhdGlvbl90d29jb21wPC1mdW5jdGlvbihkYXRhLG5ldEEsbmV0QixsZXZlbEEsbGV2ZWxCLG5zaW1zKXsKICAjZ2V0IGRhdGEKICAjbGV2ZWxzKGRhdGEkbmV0X3R5cGUpPC1jKCJoc3JhbmRfZHRlbXAiLCAibmV0X2R0ZW1wIiwgInJhbmRfZHRlbXAiLCJyYmRjX2R0ZW1wIiwgInJibmRjX2R0ZW1wIikKICAjc3BsaXQgZGF0YSBmb3IgdHdvIGxldmVscwogIG5ldDEgPC0gZGF0YSU+JQogICAgZmlsdGVyKG5ldF90eXBlPT1uZXRBKQogIG5ldDIgPC0gZGF0YSU+JQogICAgZmlsdGVyKG5ldF90eXBlPT1uZXRCKQogIHR3b2NvbXBzPC1yYmluZChuZXQxLG5ldDIpCgogICMgaW5pdGlhbGl6ZSB0ZXN0CiAgY29tYmluZWRfcmVzaXN0YW5jZSA8LSBjKG5ldDEkcmVzaXN0YW5jZSxuZXQyJHJlc2lzdGFuY2UpICNjb21iaW5lcyByZXNpc3RhbmNlIHZhbHVlcyBpbnRvIGEgdmVjdG9yCiAgY29tYmluZWRfbGFiZWxzIDwtIGMobmV0MSRuZXRfdHlwZSxuZXQyJG5ldF90eXBlKSNjb21iaW5lcyBuZXR3b3JrIHR5cGUgaW50byBhIHZlY3RvcgogIGRpZmZfb2JzIDwtIG1lYW4obmV0MSRyZXNpc3RhbmNlKSAtIG1lYW4obmV0MiRyZXNpc3RhbmNlKQogIG1vZGVsIDwtIGFub3ZhKGxtKHR3b2NvbXBzJHJlc2lzdGFuY2UgfiB0d29jb21wcyRuZXRfdHlwZSkpIAogIG9idC5GIDwtIG1vZGVsJCJGIHZhbHVlIlsxXSAgICAgIyBPdXIgb2J0YWluZWQgRiAgc3RhdGlzdGljCiAgb2J0LnAgPC0gbW9kZWwkIlByKD5GKSJbMV0KICAKICAjIHBlcm11dGF0aW9uIHRlc3QKICBkaWZmcyA8LSByZXAoTkEsIG5zaW1zKQogIGZzdGF0czwtcmVwKE5BLG5zaW1zKQogIGZvciAoaSBpbiAxOm5zaW1zKSB7CiAgICAjc2h1ZmZsZSB0aGUgbGFiZWxzCiAgICBzaHVmZmxlZF9sYWJlbHMgPC0gc2FtcGxlKGNvbWJpbmVkX2xhYmVscywgcmVwbGFjZSA9IEZBTFNFKQogICAgdHdvY29tcHMkc2h1ZmZsZWRfbGFiZWxzPC1zaHVmZmxlZF9sYWJlbHMKICAgICNzaHVmZmxlIHRoZSByZXNpc3RhbmNlcwogICAgc2h1ZmZsZWRfcmVzaXN0YW5jZTwtc2FtcGxlKGNvbWJpbmVkX3Jlc2lzdGFuY2UsIHJlcGxhY2UgPSBGQUxTRSkKICAgIHR3b2NvbXBzJHNodWZmbGVkX3Jlc2lzdGFuY2U8LXNodWZmbGVkX3Jlc2lzdGFuY2UKICAgICNnZXQgdGhlIGRpZmZlcmVuY2VzIGluIHRoZSBtZWFucyBvZiB0aGUgc2h1ZmZsZWQgZ3JvdXBzCiAgICBkaWZmc1tpXSA8LSBtZWFuKHNodWZmbGVkX3Jlc2lzdGFuY2Vbc2h1ZmZsZWRfbGFiZWxzID09IGxldmVsQV0pIC0gIG1lYW4oc2h1ZmZsZWRfcmVzaXN0YW5jZVtzaHVmZmxlZF9sYWJlbHMgPT0gbGV2ZWxCXSkKICAgICNydW4gYSBuZXcgYW5vdmEKICAgIG5ld21vZGVsIDwtIGFub3ZhKGxtKHR3b2NvbXBzJHNodWZmbGVkX3Jlc2lzdGFuY2UgfiB0d29jb21wcyRzaHVmZmxlZF9sYWJlbHMpKSAKICAgIGZzdGF0c1tpXSA8LSBuZXdtb2RlbCQiRiB2YWx1ZSJbMV0gICAgICMgZ2V0IGEgbmV3IEYgIHN0YXRpc3RpYwogIH0KCiAgI2NhbGN1bGF0ZSB0aGUgdHdvLXNpZGVkIHAtdmFsdWUKICAjIHAtdmFsdWUgPSAobnVtYmVyIG9mIG1vcmUgZXh0cmVtZSBkaWZmZXJlbmNlcyB0aGFuIGRpZmZfb2JzKS9uc2ltcwogIHB2YWxfZGlmZnM8LWxlbmd0aChkaWZmc1thYnMoZGlmZnMpID49IGFicyhkaWZmX29icyldKS9uc2ltcwogIHB2YWxfZnN0YXRkaWZzPC0obGVuZ3RoKGZzdGF0c1thYnMoZnN0YXRzKSA+IGFicyhvYnQuRildKSsxKS8oMStuc2ltcykKICByZXR1cm4oYyhkaWZmX29icyxvYnQucCxvYnQuRixwdmFsX2RpZmZzLHB2YWxfZnN0YXRkaWZzKSkKfQoKcmVzaXN0YW5jZV9wZXJtczwtZnVuY3Rpb24oZmlsZSxuc2ltcyl7CiAgI2dldCBkYXRhCiAgcmVzaXN0X2RhdGE8LXJlYWQuY3N2KGZpbGUpCiAgI3NlbGVjdCBjb2x1bW5zCiAgc2VsZWN0ZGF0YTwtcmVzaXN0X2RhdGElPiUKICAgIHNlbGVjdChoc3JhbmRfZHRlbXAsbmV0X2R0ZW1wLHJhbmRfZHRlbXAscmJkY19kdGVtcCxyYm5kY19kdGVtcCkKICAjZ2V0IGludG8gbG9uZyBmb3JtYXQKICBsb25nZGF0YSA8LSBnYXRoZXIoc2VsZWN0ZGF0YSwga2V5PW5ldF90eXBlLHZhbHVlPXJlc2lzdGFuY2UsZmFjdG9yX2tleSA9IFRSVUUpCiAgI3NldHVwIHN0b3JhZ2UgZm9yIHBlcm11dGF0aW9uIHJlc3VsdHMKICByZXNtYXQ8LW1hdHJpeChOQSxucm93PTAsbmNvbD03KQogIHB2YWxzPC1yZXAoKQogICNydW4gcGVybXV0YXRpb25zCiAgZm9yIChqIGluIDE6KGxlbmd0aChsZXZlbHMobG9uZ2RhdGEkbmV0X3R5cGUpKS0xKSl7CiAgICBuZXRBPC1sZXZlbHMobG9uZ2RhdGEkbmV0X3R5cGUpW2pdCiAgICBmb3IgKGkgaW4gKGorMSk6bGVuZ3RoKGxldmVscyhsb25nZGF0YSRuZXRfdHlwZSkpKXsKICAgICAgbmV0QjwtbGV2ZWxzKGxvbmdkYXRhJG5ldF90eXBlKVtpXQogICAgICBsZXZlbEE8LWoKICAgICAgbGV2ZWxCPC1pCiAgICAgICNwcmludChjKG5ldEEsbmV0QixsZXZlbEEsbGV2ZWxCKSkKICAgICAgcGVybXJlc3VsdHM8LW15cGVybXV0YXRpb25fdHdvY29tcChsb25nZGF0YSxuZXRBLG5ldEIsbGV2ZWxBLGxldmVsQiwxMDAwKQogICAgICAjcHJpbnQocmVzdWx0cykKICAgICAgI3B2YWxzPC1hcHBlbmQocHZhbHMscGVybXJlc3VsdHNbNV0pCiAgICAgIHBlcm1yZXM8LWMocGVybXJlc3VsdHMsbmV0QSxuZXRCKQogICAgICByZXNtYXQ8LXJiaW5kKHJlc21hdCxwZXJtcmVzKQogIH0KfQogIGFsbF9yZXN1bHRzPC1jYmluZChyZXNtYXQscC5hZGp1c3QoYXMubnVtZXJpYyhyZXNtYXRbLDVdKSwiaG9sbSIpKSAjYWRqdXN0IHAgdmFsdWVzIHVzaW5nIHRoZSBob2xtIGNvcnJlY3Rpb24KICByZXN1bHRzPC1hcy5kYXRhLmZyYW1lKGFsbF9yZXN1bHRzKQogIGNvbG5hbWVzKHJlc3VsdHMpPC1jKCJkaWZmX29icyIsIm9idC5wIiwib2J0LkYiLCJwdmFsX2RpZmZzIiwicHZhbF9mc3RhdGRpZnMiLCJuZXQxIiwibmV0MiIsInBfYWRqdXN0X2hvbG0iKQogIHJldHVybihyZXN1bHRzKQp9CgojVEVTVApyZXNpc3RhbmNlX3Blcm1zKCJURVNUX3Jlc2lzdGFuY2VfcGVybXMuY3N2IiwxMDAwKQpgYGAKCiMjIFJvYnVzdG5lc3MgdnMuIENvbm5lY3RhbmNlCgpTZWUgQXBwZW5kaXggUzIgZm9yIGRlc2NyaXB0aW9uLgoKYGBge3J9CiNsb2FkIGRhdGEKcm9idXN0bmVzczwtcmVhZC5jc3YoIlJvYnVzdG5lc3MuY3N2IikKI2NoZWNrIGRpc3RyaWJ1dGlvbiBvZiBjb25uZWN0YW5jZQpwbG90KGRlbnNpdHkoKGxvZyhyb2J1c3RuZXNzJGNvbm5lY3RhbmNlKSkpKSAjSXQncyB3b25reS4KCiNtYWtlIGl0IGVhc3kgdG8gcGxvdCByZW1vdmFsIG1vZGVsIG9mIGNob2ljZSB2cyBjb25uZWN0YW5jZSB3aXRoIGEgbGluZWFyIGZpdApteV9SQ2xpbnJlZ19wbG90PC1mdW5jdGlvbihjaG9pY2UpewogIGRhdGE8LXJvYnVzdG5lc3MgJT4lCiAgICBmaWx0ZXIobW9kZWwhPSJMVF9CSCIsIG1vZGVsIT0iTFRfSEgiLG1vZGVsIT0iTFRfU0giLG1vZGVsIT0iVG9sZXJhbmNlX2hpZ2giLFI1MF93aG89PSJib3RoIixtb2RlbD09Y2hvaWNlKQoKICBwPC1nZ3Bsb3RSZWdyZXNzaW9uKGxtKG1lYW4gfiBjb25uZWN0YW5jZSwgZGF0YSA9IGRhdGEpLGNob2ljZSkKICBwK2dlb21fcG9pbnQoeT1kYXRhJG1lYW4seD1kYXRhJGNvbm5lY3RhbmNlLGFlcyhjb2xvcj1kYXRhJGdyb3VwKSxzaXplPTYpCiAgCn0KCiNydW4gYSBLZW5kYWxsJ3MgY29lZmZpY2llbnQgb2YgcmFuayBjb3JyZWxhdGlvbiB0ZXN0IG9uIHJlbW92YWwgbW9kZWwgb2YgY2hvaWNlCm15X2NvcnRlc3Q8LWZ1bmN0aW9uKGNob2ljZSl7CiAgZGF0YTwtcm9idXN0bmVzcyAlPiUKICAgIGZpbHRlcihtb2RlbCE9IkxUX0JIIiwgbW9kZWwhPSJMVF9ISCIsbW9kZWwhPSJMVF9TSCIsbW9kZWwhPSJUb2xlcmFuY2VfaGlnaCIsUjUwX3dobz09ImJvdGgiLG1vZGVsPT1jaG9pY2UpCiAgY3Q8LWNvci50ZXN0KGRhdGEkbWVhbixkYXRhJGNvbm5lY3RhbmNlLG1ldGhvZD0ia2VuZGFsbCIpCiAgcmV0dXJuKGN0KQp9CiNydW4gZm9yIGRpZmZlcmVudCByZW1vdmFsIG1vZGVscyAKbXlfUkNsaW5yZWdfcGxvdCgiYmxlYWNoIikKbXlfY29ydGVzdCgiYmxlYWNoIikgCm15X1JDbGlucmVnX3Bsb3QoIlJhbmRvbV9saW5rIikKbXlfY29ydGVzdCgiUmFuZG9tX2xpbmsiKQpteV9SQ2xpbnJlZ19wbG90KCJMVF9CTCIpCm15X2NvcnRlc3QoIkxUX0JMIikKbXlfUkNsaW5yZWdfcGxvdCgiTFRfSEwiKQpteV9jb3J0ZXN0KCJMVF9ITCIpCm15X1JDbGlucmVnX3Bsb3QoIkxUX1NMIikKbXlfY29ydGVzdCgiTFRfU0wiKQpteV9SQ2xpbnJlZ19wbG90KCJEZWdyZWVfaGlnaCIpCm15X2NvcnRlc3QoIkRlZ3JlZV9oaWdoIikKbXlfUkNsaW5yZWdfcGxvdCgiRGVncmVlX2xvdyIpCm15X2NvcnRlc3QoIkRlZ3JlZV9sb3ciKQpteV9SQ2xpbnJlZ19wbG90KCJSYW5kb21fbm9kZSIpCm15X2NvcnRlc3QoIlJhbmRvbV9ub2RlIikKbXlfUkNsaW5yZWdfcGxvdCgiVG9sZXJhbmNlX2xvdyIpCm15X2NvcnRlc3QoIlRvbGVyYW5jZV9sb3ciKQoKYGBgCgojIyBTeW1iaW9udCBub2RlIHRoZXJtYWwgdG9sZXJhbmNlczogcGlja2luZyBtaXNzaW5nIHRvbGVyYW5jZXMKCkNvZGUgZm9yIEFwcGVuZGl4IFMxLCBGaWd1cmUgUzE6IEZyZXF1ZW5jeSBkaXN0cmlidXRpb24gb2YgU3ltYmlvZGluaXVtIHRoZXJtYWwgdG9sZXJhbmNlIHNjb3JlcyBhZGFwdGVkIGZyb20gU3dhaW4gZXQgYWwuICgyMDE3KTsgZGlzdHJpYnV0aW9uIGlzIGNvbG9yZWQgYnkgdG9sZXJhbmNlIHJhbmdlLCByZWQgaXMgaGlnaGx5IHN1c2NlcHRpYmxlLCBvcmFuZ2UgaXMgbWVkaXVtIHRvbGVyYW5jZSwgYW5kIHllbGxvdyBpcyBoaWdoIHRvbGVyYW5jZS4gU3dhaW4gZXQgYWwuICgyMDE3KSBwcm92aWRlcyBhIGZyYW1ld29yayBmb3IgYSBjb25zZW5zdXMgb2YgU3ltYmlvZGluaXVtIHRoZXJtb3RvbGVyYW5jZSByYW5rcyBkZXZlbG9wZWQgZnJvbSByYW5rLWFnZ3JlZ2F0aW9uIG1ldGhvZHMuIFRoZWlyIHJhbmtpbmcgc2NoZW1lIG9yZGVycyBTeW1iaW9kaW5pdW0gcGh5bG90eXBlcyBmcm9tIDAtMTAwLCBidXQgdGhlIHJhbmsgdmFsdWVzIGFyZSBub3QgaW5kaWNhdGl2ZSBvZiB0b3RhbCBtYWduaXR1ZGUgZGlmZmVyZW5jZXMgaW4gdGhlcm1vdG9sZXJhbmNlLiBUbyBkZXRlcm1pbmUgdG9sZXJhbmNlcyBvZiB0aGUgdW5saXN0ZWQgc3ltYmlvbnQgdHlwZXMgaW4gb3VyIG5ldHdvcmssIHJhbmsgdmFsdWVzIHdlcmUgcmFuZG9tbHkgZHJhd24gZnJvbSB0aGUgaGlnaCwgbWVkaXVtLCBhbmQgbG93IHRoZXJtYWwgdG9sZXJhbmNlIGZyZXF1ZW5jeSBkaXN0cmlidXRpb25zIGluIHRoZSByZWxhdGl2ZSBwcm9wb3J0aW9ucyBvZiB0aGUgY2xhZGVzIHJlcHJlc2VudGVkIGluIHRob3NlIGRpc3RyaWJ1dGlvbnMuIFRodXMsIGZvciBlYWNoIHNpbXVsYXRpb24gb2YgZWl0aGVyIHRoZSBibGVhY2hpbmcgb3IgZGlmZmVyZW50IHJlbW92YWwgbW9kZWxzIGRlc2NyaWJlZCBiZWxvdywgdGhlIHN5bWJpb250IHRvbGVyYW5jZXMgdmFyaWVkIHdpdGhpbiBhIHNldCBkaXN0cmlidXRpb24gKEZpZ3VyZSAxRCkuCmBgYHtyfQojIyMgRXhwbG9yaW5nIGRhdGEgZnJvbSBTd2FpbiBldCBhbCAyMDE2YSAjIyMjCmRhdGE8LXJlYWQuY3N2KCJTd2FpblN5bWJzUi5jc3YiKQojZnJlcXVlbmN5IGRpc3RyaWJ1dGlvbiAtLT4gaW5zZXQgZnJvbSBmaWd1cmUgMSBmcm9tIHBhcGVyCmJpbnM9c2VxKDAsMTAwLGJ5PTIpCiNzcGxpdCB0aGUgZnJlcXVlbmN5IGRpc3RyaWJ1dGlvbiBpbnRvIDMgc3Vic2V0cyBkZWZpbmVkIGJ5IHN3YWluIGV0IGFsIDE2CmxvdyA8LSBkYXRhW3doaWNoKGRhdGEkU2NvcmVSX2s8PTE3KSxdCmhpZ2ggPC0gZGF0YVt3aGljaChkYXRhJFNjb3JlUl9rPj0zMyksXQptaWRkbGU8LSBkYXRhW3doaWNoKGRhdGEkU2NvcmVSX2s+MTcgJiBkYXRhJFNjb3JlUl9rPDMzKSxdCiNIaWdoIGhpc3QKaGlzdChoaWdoJFNjb3JlUl9rLCBicmVha3M9YmlucywgY29sPSJ5ZWxsb3ciLCB5bGltPWMoMCwxMiksIHhsYWI9InRoZXJtb3RvbGVyYW5jZSBzY29yZSIsIG1haW49IkZyZXF1ZW5jeSBEaXN0cmlidXRpb25zIG9mIFRoZXJtb3RvbGVyYW5jZSBTY29yZXMiKQojbWlkZGxlIGhpc3QKaGlzdChtaWRkbGUkU2NvcmVSX2ssIGJyZWFrcz1iaW5zLCBjb2w9Im9yYW5nZSIsIGFkZD1UKQojbG93IGhpc3QKaGlzdChsb3ckU2NvcmVSX2ssIGJyZWFrcz1iaW5zLCBjb2w9InJlZCIsIGFkZD1UKQoKYGBgCkNvZGUgZm9yIGZpdHRpbmcgdGhlIGFib3ZlIHRoZXJtYWwgdG9lbHJhbmNlIGRpc3RyaWJ1dGlvbnMgZGlzdHJpYnV0aW9ucwpgYGB7cn0KIyMjIEZpdCB0aGUgVGhlcm1vdG9sZXJhbmNlIERpc3RyaWJ1dGlvbnMgIyMjIwojZmlyc3QgbmVlZCB0aGUgbWl4dG9vbHMgcGFja2FnZQppZighKCJtaXh0b29scyIgJWluJSBpbnN0YWxsZWQucGFja2FnZXMoKSkpe2luc3RhbGwucGFja2FnZXMoIm1peHRvb2xzIil9CmxpYnJhcnkoIm1peHRvb2xzIikKI1VzZSB0aGUgZXhwZWN0YXRpb24gTWF4aW1pemF0aW9uIChFTSkgQXBwcm9hY2gKZW0gPC0gbm9ybWFsbWl4RU0oZGF0YSRTY29yZVJfaywgYXJidmFyID0gVFJVRSxlcHNpbG9uID0gMWUtMDMsIGsgPSAzKQojIEVzdGltYXRlZCBtZWFucwplbSRtdQojIEVzdGltYXRlZCBzdGFuZGFyZCBkZXZpYXRpb25zCmVtJHNpZ21hCiMjI1JFU1VMVFMKIz4gZW0kbXUKI1sxXSAgNy45ODMwMjkgMjkuNDA4NTcwIDUxLjkwMDMzOQojPiBlbSRzaWdtYQojWzFdICAyLjcxMTQ4MCAgOS41MDA1ODcgMTguMDA2NjI2CgojZG8gMTAwMDAgc2ltdWxhdGlvbnMgIyMjIwpydW5zIDwtIDEwMDAwCnNpbXMubG93IDwtIHJub3JtKHJ1bnMsbWVhbj03Ljk4MzAyOSxzZD0yLjcxMTQ4MCkKc2ltcy5taWRkbGUgPC1ybm9ybShydW5zLG1lYW49MjkuNDA4NTcwLHNkPTkuNTAwNTg3KQpzaW1zLmhpZ2g8LXJub3JtKHJ1bnMsbWVhbj01MS45MDAzMzksc2Q9MTguMDA2NjI2KQojSGlnaCBoaXN0Cmhpc3Qoc2ltcy5oaWdoLCBjb2w9InllbGxvdyIpCiNtaWRkbGUgaGlzdApoaXN0KHNpbXMubWlkZGxlLCBjb2w9Im9yYW5nZSIsIGFkZD1UKQojbG93IGhpc3QKaGlzdChzaW1zLmxvdywgY29sPSJyZWQiLCBhZGQ9VCkKCmBgYApDb2RlIGZvciBzZXR0aW5ndXAgZm9yIHRvZWxyYW5jZWEgZnVuY3Rpb24KYGBge3J9CiNWQUxVRVMgVEhBVCBBUkUgTkVFREVEICMjIyMgCiMjVGhlc2UgYXJlIGp1c3QgdGhlIG51bWJlcnMgb2Ygc3ltYnMgZnJvbSBlYWNoIGNsYWRlIGluIGVhY2ggZnJlcSBsZXZlbCBmcm9tIFN3YWluCnN3YWluX0E8LTgKc3dhaW5fQjwtOApzd2Fpbl9DPC03MQpzd2Fpbl9EPC0xOQpzd2Fpbl9FPC0xCnN3YWluX0Y8LTMKCmxvd19BPC0xCm1pZGRsZV9BPC0zCmhpZ2hfQTwtNApwcm9iX2xvd19BPC1sb3dfQS9zd2Fpbl9BCnByb2JfbWlkZGxlX0E8LW1pZGRsZV9BL3N3YWluX0EKcHJvYl9oaWdoX0E8LWhpZ2hfQS9zd2Fpbl9BCgpsb3dfQjwtMwptaWRkbGVfQjwtNApoaWdoX0I8LTEKcHJvYl9sb3dfQjwtbG93X0Ivc3dhaW5fQgpwcm9iX21pZGRsZV9CPC1taWRkbGVfQi9zd2Fpbl9CCnByb2JfaGlnaF9CPC1oaWdoX0Ivc3dhaW5fQgoKbG93X0M8LTI0Cm1pZGRsZV9DPC0xOQpoaWdoX0M8LTI4CnByb2JfbG93X0M8LWxvd19DL3N3YWluX0MKcHJvYl9taWRkbGVfQzwtbWlkZGxlX0Mvc3dhaW5fQwpwcm9iX2hpZ2hfQzwtaGlnaF9DL3N3YWluX0MKCmxvd19EPC0zCm1pZGRsZV9EPC00CmhpZ2hfRDwtMTIKcHJvYl9sb3dfRDwtbG93X0Qvc3dhaW5fRApwcm9iX21pZGRsZV9EPC1taWRkbGVfRC9zd2Fpbl9ECnByb2JfaGlnaF9EPC1oaWdoX0Qvc3dhaW5fRAoKbG93X0U8LTEKcHJvYl9sb3dfRTwtMQpwcm9iX21pZGRsZV9FPC0wCnByb2JfaGlnaF9FPC0wCgpoaWdoX0Y8LTMKcHJvYl9sb3dfRjwtMApwcm9iX21pZGRsZV9GPC0wCnByb2JfaGlnaF9GPC0xCiNPSyBzbyB0aGVzZSBhcmUgdGhlIG51bWJlciBvZiBzeW1icyBpbiBlYWNoIGNsYWRlIHRoYXQgZG9udCBoYXZlIHRvbGVyYW5jZXMgCm5ldF9BPC02Cm5ldF9CPC0xMwpuZXRfQzwtMTYwCm5ldF9EPC02Cm5ldF9FPC0wCm5ldF9GPC0wCmBgYAoKRnVuY3Rpb24gZm9yIGdldHRpbmcgdGhlIHRvbGVyYW5jZXMKYGBge3J9CmdldF90b2xzPC1mdW5jdGlvbihzaXplLGxvd3Byb2IsbWlkcHJvYixoaWdocHJvYixydW5zLHNpbXMubG93LHNpbXMubWlkZGxlLHNpbXMuaGlnaCl7CiAgI21ha2UgYSBzYW1wbGUgZGlzdHJpYnV0aW9uIHdoZXJlIDEgY29ycmVzcG9uZHMgdG8gdGhlIGxvdyB0aGVybWFsdG9sZXJhbmNlLAogICMgMiBpcyB0aGUgbWVkaXVtIGFuZCAzIGlzIGhpZ2guIDE6MyBhcmUgcHV0IGludG8gdGhlIHNhbXBsZSBkaXN0cmlidXRpb24gCiAgI2Jhc2VkIG9uIHRoZSBwcm9iYWJpbGl0aWVzIGluIHRoZSBmdW5jdGlvbiBpbnB1dCB0aGF0IGFyZSBjbGFkZSBzcGVjaWZpYwogIHNhbXBsZWRpc3RyPC1zYW1wbGUoeCA9IDE6Mywgc2l6ZSA9IHNpemUsIHByb2IgPSBjKGxvd3Byb2IsIG1pZHByb2IsIGhpZ2hwcm9iKSwgcmVwbGFjZT1UUlVFKQogIHRvbGVyYW5jZTwtbnVtZXJpYyhsZW5ndGg9c2l6ZSkKICBmb3IgKGkgaW4gMTpzaXplKSB7ICNmb3IgZWFjaCBudW1iZXIgaW4gdGhlIHNhbXBsZWRpc3RyIGRvOgogICAgaWYgKHNhbXBsZWRpc3RyW2ldPT0xKXsKICAgICAgdG9sZXJhbmNlW2ldPC1zYW1wbGUoeD1zaW1zLmxvdyxzaXplPTEpICNwdWxsIGZyb20gbG93CiAgICB9CiAgICBlbHNlIGlmIChzYW1wbGVkaXN0cltpXT09Mil7ICNwdWxsIGZyb20gbWlkZGxlCiAgICAgIHRvbGVyYW5jZVtpXTwtc2FtcGxlKHg9c2ltcy5taWRkbGUsc2l6ZT0xKQogICAgfQogICAgZWxzZSAKICAgICAgdG9sZXJhbmNlW2ldPC1zYW1wbGUoeD1zaW1zLmhpZ2gsc2l6ZT0xKSAjcHVsbCBmcm9tIGhpZ2gKICB9CiAgdG9sZXJhbmNlPC1zcXJ0KHRvbGVyYW5jZSkvMTAgI3Rha2UgdGhlIHNxcnQgYmVjYXVzZSBzd2FpbidzIHRvbGVyYW5jZXMgYXJlIG5vdCBpbmRpY2F0aXZlIG9mIG1hZ25pdHVkZSBhbmQgdGhlbiBkaXZpZGUgYnkgMTAgdG8gZ2V0IGEgbnVtYmVyIGZyb20gMC0xCiAgcmV0dXJuKHRvbGVyYW5jZSkKfQpgYGAKCk1ha2UgMTAwIHNpbXVsYXRpb25zIHdvcnRoIG9mIHRvbGVyYW5jZSBmaWxlcwpgYGB7cn0KdHJpYWxzPTEgI2FjdHVhbGx5IGRpZCAxMDAsIGJ1dCBkb24ndCB3YW50IHRvIG1ha2UgYSB0b24gb2YgbmV3IGZpbGVzLCBzbyBoZXJlIGlzIDEgZXhhbXBsZSBmaWxlLgpsc3Q9c2VxKDE6dHJpYWxzKQpmb3IoaSBpbiBzZXFfYWxvbmcobHN0KSl7CiAgQ3RvbHM8LWdldF90b2xzKG5ldF9DLHByb2JfbG93X0MscHJvYl9taWRkbGVfQyxwcm9iX2hpZ2hfQywxMDAwMCxzaW1zLmxvdyxzaW1zLm1pZGRsZSxzaW1zLmhpZ2gpCiAgQXRvbHM8LWdldF90b2xzKG5ldF9BLHByb2JfbG93X0EscHJvYl9taWRkbGVfQSxwcm9iX2hpZ2hfQSwxMDAwMCxzaW1zLmxvdyxzaW1zLm1pZGRsZSxzaW1zLmhpZ2gpCiAgQnRvbHM8LWdldF90b2xzKG5ldF9CLHByb2JfbG93X0IscHJvYl9taWRkbGVfQixwcm9iX2hpZ2hfQiwxMDAwMCxzaW1zLmxvdyxzaW1zLm1pZGRsZSxzaW1zLmhpZ2gpCiAgRHRvbHM8LWdldF90b2xzKG5ldF9ELHByb2JfbG93X0QscHJvYl9taWRkbGVfRCxwcm9iX2hpZ2hfRCwxMDAwMCxzaW1zLmxvdyxzaW1zLm1pZGRsZSxzaW1zLmhpZ2gpCiAgQ3RvbHM8LWFzLmRhdGEuZnJhbWUoQ3RvbHMpCiAgQXRvbHM8LWFzLmRhdGEuZnJhbWUoQXRvbHMpCiAgQnRvbHM8LWFzLmRhdGEuZnJhbWUoQnRvbHMpCiAgRHRvbHM8LWFzLmRhdGEuZnJhbWUoRHRvbHMpCiAgY29sbmFtZXMoQ3RvbHMpIDwtIGMoInRvbGVyYW5jZSIpCiAgY29sbmFtZXMoQnRvbHMpIDwtIGMoInRvbGVyYW5jZSIpCiAgY29sbmFtZXMoRHRvbHMpIDwtIGMoInRvbGVyYW5jZSIpCiAgY29sbmFtZXMoQXRvbHMpIDwtIGMoInRvbGVyYW5jZSIpCiAgbmV3c2NvcmVzPC1yYmluZChBdG9scyxCdG9scyxDdG9scyxEdG9scykKICBuYW1lPC1wYXN0ZSgndHJpYWwnLGksIi5jc3YiLHNlcD0iIikKICAKICB3cml0ZS5jc3YobmV3c2NvcmVzLG5hbWUscm93Lm5hbWVzPUZBTFNFKQp9CgpgYGAKCk5vdyBmb3IgdGhlIHNodWZmbGVkIHRvbGVyYW5jZXM6CmBgYHtyfQojIE5vdyBnZXQgdGhlIHRvbGVyYW5lcyBmb3IgdGhlIFNodWZmbGVkIFN5bWJpb250cy8gU2h1ZmZsZWQgUmFuZG9tIG51bGwgbW9kZWwgIyMjIwojZm9yIHRoZSByYW5kb20gc2V0IG9mIHRvbGVyYW5jZXMgd2hlcmUgdGhleSBhcmUgYWxsIHB1bGxlZCBlcXVhbGx5IGZyb20gdGhlIDMgZGlzdHJpYnV0aW9ucwpydW5zPTEwMDAwCnNpbXMubG93IDwtIHJub3JtKHJ1bnMsbWVhbj03Ljk4MzAyOSxzZD0yLjcxMTQ4MCkKc2ltcy5sb3c8LXN1YnNldChzaW1zLmxvdyxzaW1zLmxvdz49MCkKc2ltcy5taWRkbGUgPC1ybm9ybShydW5zLG1lYW49MjkuNDA4NTcwLHNkPTkuNTAwNTg3KQpzaW1zLm1pZGRsZTwtc3Vic2V0KHNpbXMubWlkZGxlLHNpbXMubWlkZGxlPj0wKQpzaW1zLmhpZ2g8LXJub3JtKHJ1bnMsbWVhbj01MS45MDAzMzksc2Q9MTguMDA2NjI2KSAKc2ltcy5oaWdoPC1zdWJzZXQoc2ltcy5oaWdoLHNpbXMuaGlnaDw9MTAwKQpzaW1zLmhpZ2g8LXN1YnNldChzaW1zLmhpZ2gsc2ltcy5oaWdoPj0wKQoKI3NvIHRoZSBmb2xsb3dpbmcgZnVuY3Rpb24gZG9lcyB3aGF0IHRoZSBvbGQgb25lIGRpZCBidXQgYWxzbyBzcGVjaWZpZXMKI2lmIHVzaW5nIHByb2JhYmlsaWVzIGRldGVybWluZWQgZnJvbSBkYXRhIG9yIGlmIHB1bGxpbmcgYWxsIHZhbHVlcyBmcm9tIAojdGhlIGRpc3RyaWJ1dGlvbnMgd2l0aCB0aGUgc2FtZSBwcm9iYWJpbGl0eSAoMS8zKQphbGx0aGVkYXRhPC1mdW5jdGlvbihwcm9iYWJpbGl0eSx0cmlhbHMsc2ltcy5sb3csc2ltcy5taWRkbGUsc2ltcy5oaWdoLG5ldF9BLG5ldF9CLG5ldF9DLG5ldF9EKXsKICBpZiAocHJvYmFiaWxpdHk9PTEpewogICAgc3dhaW5fQTwtOAogICAgc3dhaW5fQjwtOAogICAgc3dhaW5fQzwtNzEKICAgIHN3YWluX0Q8LTE5CiAgICBzd2Fpbl9FPC0xCiAgICBzd2Fpbl9GPC0zCiAgICAKICAgIGxvd19BPC0xCiAgICBtaWRkbGVfQTwtMwogICAgaGlnaF9BPC00CiAgICBwcm9iX2xvd19BPC1sb3dfQS9zd2Fpbl9BCiAgICBwcm9iX21pZGRsZV9BPC1taWRkbGVfQS9zd2Fpbl9BCiAgICBwcm9iX2hpZ2hfQTwtaGlnaF9BL3N3YWluX0EKICAgIAogICAgbG93X0I8LTMKICAgIG1pZGRsZV9CPC00CiAgICBoaWdoX0I8LTEKICAgIHByb2JfbG93X0I8LWxvd19CL3N3YWluX0IKICAgIHByb2JfbWlkZGxlX0I8LW1pZGRsZV9CL3N3YWluX0IKICAgIHByb2JfaGlnaF9CPC1oaWdoX0Ivc3dhaW5fQgogICAgCiAgICBsb3dfQzwtMjQKICAgIG1pZGRsZV9DPC0xOQogICAgaGlnaF9DPC0yOAogICAgcHJvYl9sb3dfQzwtbG93X0Mvc3dhaW5fQwogICAgcHJvYl9taWRkbGVfQzwtbWlkZGxlX0Mvc3dhaW5fQwogICAgcHJvYl9oaWdoX0M8LWhpZ2hfQy9zd2Fpbl9DCiAgICAKICAgIGxvd19EPC0zCiAgICBtaWRkbGVfRDwtNAogICAgaGlnaF9EPC0xMgogICAgcHJvYl9sb3dfRDwtbG93X0Qvc3dhaW5fRAogICAgcHJvYl9taWRkbGVfRDwtbWlkZGxlX0Qvc3dhaW5fRAogICAgcHJvYl9oaWdoX0Q8LWhpZ2hfRC9zd2Fpbl9ECiAgICAKICAgIGxvd19FPC0xCiAgICBwcm9iX2xvd19FPC0xCiAgICBwcm9iX21pZGRsZV9FPC0wCiAgICBwcm9iX2hpZ2hfRTwtMAogICAgCiAgICBoaWdoX0Y8LTMKICAgIHByb2JfbG93X0Y8LTAKICAgIHByb2JfbWlkZGxlX0Y8LTAKICAgIHByb2JfaGlnaF9GPC0xCiAgICAKICB9CiAgZWxzZQogICAgcHJvYl9sb3dfQz1wcm9iX21pZGRsZV9DPXByb2JfaGlnaF9DPXByb2JfbG93X0E9cHJvYl9taWRkbGVfQT1wcm9iX2hpZ2hfQT1wcm9iX2xvd19CPXByb2JfbWlkZGxlX0I9cHJvYl9oaWdoX0I9cHJvYl9sb3dfRD1wcm9iX21pZGRsZV9EPXByb2JfaGlnaF9EPSgxLzMpCiAgCiAgI25ldF9BPC04CiAgI25ldF9CPC0xOAogICNuZXRfQzwtMTc3CiAgI25ldF9EPC03CiAgI25ldF9FPC0wCiAgI25ldF9GPC0wCiAgbHN0PXNlcSgxOnRyaWFscykKICBmb3IoaSBpbiBzZXFfYWxvbmcobHN0KSl7CiAgICBDdG9sczwtZ2V0X3RvbHMobmV0X0MscHJvYl9sb3dfQyxwcm9iX21pZGRsZV9DLHByb2JfaGlnaF9DLDEwMDAwLHNpbXMubG93LHNpbXMubWlkZGxlLHNpbXMuaGlnaCkKICAgIEF0b2xzPC1nZXRfdG9scyhuZXRfQSxwcm9iX2xvd19BLHByb2JfbWlkZGxlX0EscHJvYl9oaWdoX0EsMTAwMDAsc2ltcy5sb3csc2ltcy5taWRkbGUsc2ltcy5oaWdoKQogICAgQnRvbHM8LWdldF90b2xzKG5ldF9CLHByb2JfbG93X0IscHJvYl9taWRkbGVfQixwcm9iX2hpZ2hfQiwxMDAwMCxzaW1zLmxvdyxzaW1zLm1pZGRsZSxzaW1zLmhpZ2gpCiAgICBEdG9sczwtZ2V0X3RvbHMobmV0X0QscHJvYl9sb3dfRCxwcm9iX21pZGRsZV9ELHByb2JfaGlnaF9ELDEwMDAwLHNpbXMubG93LHNpbXMubWlkZGxlLHNpbXMuaGlnaCkKICAgIEN0b2xzPC1hcy5kYXRhLmZyYW1lKEN0b2xzKQogICAgQXRvbHM8LWFzLmRhdGEuZnJhbWUoQXRvbHMpCiAgICBCdG9sczwtYXMuZGF0YS5mcmFtZShCdG9scykKICAgIER0b2xzPC1hcy5kYXRhLmZyYW1lKER0b2xzKQogICAgY29sbmFtZXMoQ3RvbHMpIDwtIGMoInRvbGVyYW5jZSIpCiAgICBjb2xuYW1lcyhCdG9scykgPC0gYygidG9sZXJhbmNlIikKICAgIGNvbG5hbWVzKER0b2xzKSA8LSBjKCJ0b2xlcmFuY2UiKQogICAgY29sbmFtZXMoQXRvbHMpIDwtIGMoInRvbGVyYW5jZSIpCiAgICBuZXdzY29yZXM8LXJiaW5kKEF0b2xzLEJ0b2xzLEN0b2xzLER0b2xzKQogICAgbmFtZTwtcGFzdGUoJ3J0cmlhbCcsaSwiLmNzdiIsc2VwPSIiKQogICAgCiAgICB3cml0ZS5jc3YobmV3c2NvcmVzLG5hbWUscm93Lm5hbWVzPUZBTFNFKQogIH0KfQojc2l6ZSBvZiB0aGUgY2xhZGVzIGluIHRoZSBuZXR3b3JrCm5ldF9BPC0xMApuZXRfQjwtMjAKbmV0X0M8LTIxMQpuZXRfRDwtOQphbGx0aGVkYXRhKDAsMSxzaW1zLmxvdyxzaW1zLm1pZGRsZSxzaW1zLmhpZ2gsbmV0X0EsbmV0X0IsbmV0X0MsbmV0X0QpCmBgYAoKIyBSZXZpc2VkIEZpZ3VyZXMKCkZpZ3VyZSA0LCBub3cgd2l0aG91dCB0aGUgZGVncmVlIHJlbW92YWwgbW9kZWxzCmBgYHtyLGZpZy5oZWlnaHQ9MixmaWcud2lkdGg9NC41fQpyb2J1c3RuZXNzPC1yZWFkLmNzdigiUm9idXN0bmVzcy5jc3YiKSAjcmVzdWx0cyBvZiByb2J1c3RuZXNzIGFuYWx5c2VzLCBzZWUgbWV0YWRhdGEgMiBmb3IgbW9yZSBpbmZvCnN1bW1hcnkocm9idXN0bmVzcykKI2dldCByaWQgb2YgdGhlIHJlbW92YWwgbW9kZWxzIHRoYXQgd2VyZSB0ZXN0ZWQgYnV0IG5vdCB1c2VkCmRhdGEyPC1yb2J1c3RuZXNzICU+JQogICAgZmlsdGVyKG1vZGVsIT0iTFRfQkgiLCBtb2RlbCE9IkxUX0hIIixtb2RlbCE9IkxUX1NIIixtb2RlbCE9IlRvbGVyYW5jZV9oaWdoIixtb2RlbCE9IkRlZ3JlZV9oaWdoIixtb2RlbCE9IkRlZ3JlZV9sb3ciKSU+JQogIGRyb3BsZXZlbHMoKQoKbGV2ZWxzKGRhdGEyJG1vZGVsKQpHbG9iYWxfdG90PC1kYXRhMiU+JQogIGZpbHRlcihuZXR3b3JrPT0iRyIscmVtb3ZlZCE9Imhvc3RzIixyZW1vdmVkIT0ic3ltYmlvbnRzIixSNTBfd2hvPT0iYm90aCIpCkdsb2JhbF90b3QkcmVtb3ZlZDwtZmFjdG9yKEdsb2JhbF90b3QkcmVtb3ZlZCxsZXZlbHM9YygibGlua3MiPSJsaW5rcyIsIm5vZGVzIj0iYm90aCIpKQogIApnbG9iYWw8LWdncGxvdCggR2xvYmFsX3RvdCwgYWVzKHg9YXMuZmFjdG9yKG1vZGVsKSwgeT1tZWFuLGZpbGw9cmVtb3ZlZCkpCmdfdG90YWw8LWdsb2JhbCsKICBnZW9tX2Jhcihwb3NpdGlvbj1wb3NpdGlvbl9kb2RnZSgpLHN0YXQ9ImlkZW50aXR5Iixjb2xvdXI9J2JsYWNrJyxtYXBwaW5nPWFlcyhjb2w9InJlZCIpKSArCiAgZ2VvbV9lcnJvcmJhcihhZXMoeW1pbj1tZWFuLXN0ZCwgeW1heD1tZWFuK3N0ZCksIHdpZHRoPS4yLHBvc2l0aW9uPXBvc2l0aW9uX2RvZGdlKC45KSkrCiAgc2NhbGVfZmlsbF9tYW51YWwodmFsdWVzPWMoImxpbmtzIiA9ICIjN2ZjOTdmIiwgImJvdGgiID0gIiM5OWQ4YzkiLCAiaG9zdHMiID0gIiNhNmNlZTMiLCJzeW1iaW9udHMiPSIjZmZmZjk5IiksIGRyb3AgPSBGQUxTRSkrCiAgI3lsaW0oMCwxKSsKICBzY2FsZV95X2NvbnRpbnVvdXMobGltaXRzPWMoMCwxLjA1KSxleHBhbmQgPSBjKDAsMCksYnJlYWtzPXNlcSgwLCAxLCAwLjEpKSsKICB0aGVtZShwYW5lbC5iYWNrZ3JvdW5kID0gZWxlbWVudF9ibGFuaygpKSsgCiAgdGhlbWUocGFuZWwuZ3JpZC5tYWpvciA9IGVsZW1lbnRfYmxhbmsoKSwgcGFuZWwuZ3JpZC5taW5vciA9IGVsZW1lbnRfYmxhbmsoKSxwYW5lbC5iYWNrZ3JvdW5kID0gZWxlbWVudF9ibGFuaygpLCBheGlzLmxpbmUgPSBlbGVtZW50X2xpbmUoY29sb3VyID0gImJsYWNrIikpKwogIGxhYnMoeD0iIix5PSJSb2J1c3RuZXNzLCBSNTAiKSsKICB0aGVtZShsZWdlbmQucG9zaXRpb249Im5vbmUiLGF4aXMudGV4dC54ID0gZWxlbWVudF90ZXh0KGFuZ2xlID0gNzAsIGhqdXN0ID0gMSkpKwogIHNjYWxlX3hfZGlzY3JldGUobGltaXRzPWMoIlJhbmRvbV9saW5rIiwiYmxlYWNoIiwiTFRfQkwiLCJMVF9ITCIsIkxUX1NMIiwiUmFuZG9tX25vZGUiLCJUb2xlcmFuY2VfbG93IiksbGFiZWxzPWMoIlJhbmRvbV9saW5rIj0iUmFuZG9tIiwiYmxlYWNoIj0iQmxlYWNoaW5nIiwiVG9sZXJhbmNlX2xvdyI9IlN1c2NlcHRpYmxlIiwiVG9sZXJhbmNlX2hpZ2giPSJIaWdoIFRvbGVyYW5jZSIsIkRlZ3JlZV9oaWdoIj0gIkhpZ2ggRGVncmVlIiwiRGVncmVlX2xvdyI9IkxvdyBEZWdyZWUiLCJSYW5kb21fbm9kZSI9IlJhbmRvbSBOb2RlIiwiTFRfQkgiPSJIaWdoIEF2Zy4gTGluayBUb2xlcmFuY2UiLCJMVF9CTCI9IlN1c2NlcHRpYmxlIiwiTFRfSEgiPSJIaWdoIEhvc3QgTGluayBUb2xlcmFuY2UiLCJMVF9ITCI9Ikhvc3QgU3VzY2VwdGlibGUiLCJMVF9TSCI9IkhpZ2ggU3ltYmlvbnQgTGluayBUb2xlcmFuY2UiLCJMVF9TTCI9IlN5bWJpb250IFN1c2NlcHRpYmxlIikpKwogIGdlb21fdGV4dChhZXMoIGxhYmVsPXN0YXRsYWIseT1tZWFuK3N0ZCswLjA1KSxwb3NpdGlvbiA9IHBvc2l0aW9uX2RvZGdlKDAuOSksdmp1c3QgPSAwKQoKUGFjaWZpY190b3Q8LWRhdGEyJT4lCiAgZmlsdGVyKG5ldHdvcms9PSJQIixyZW1vdmVkIT0iaG9zdHMiLHJlbW92ZWQhPSJzeW1iaW9udHMiLFI1MF93aG89PSJib3RoIikKUGFjaWZpY190b3QkcmVtb3ZlZDwtZmFjdG9yKFBhY2lmaWNfdG90JHJlbW92ZWQsbGV2ZWxzPWMoImxpbmtzIj0ibGlua3MiLCJub2RlcyI9ImJvdGgiKSkKICAKUGFjaWZpYzwtZ2dwbG90KCBQYWNpZmljX3RvdCwgYWVzKHg9YXMuZmFjdG9yKG1vZGVsKSwgeT1tZWFuLGZpbGw9cmVtb3ZlZCkpCnBfdG90YWw8LVBhY2lmaWMrCiAgZ2VvbV9iYXIocG9zaXRpb249cG9zaXRpb25fZG9kZ2UoKSxzdGF0PSJpZGVudGl0eSIsY29sb3VyPSdibGFjaycsbWFwcGluZz1hZXMoY29sPSJyZWQiKSkgKwogIGdlb21fZXJyb3JiYXIoYWVzKHltaW49bWVhbi1zdGQsIHltYXg9bWVhbitzdGQpLCB3aWR0aD0uMixwb3NpdGlvbj1wb3NpdGlvbl9kb2RnZSguOSkpKwogIHNjYWxlX2ZpbGxfbWFudWFsKHZhbHVlcz1jKCJsaW5rcyIgPSAiIzdmYzk3ZiIsICJib3RoIiA9ICIjOTlkOGM5IiwgImhvc3RzIiA9ICIjYTZjZWUzIiwic3ltYmlvbnRzIj0iI2ZmZmY5OSIpLCBkcm9wID0gRkFMU0UpKwogICN5bGltKDAsMSkrCiAgc2NhbGVfeV9jb250aW51b3VzKGxpbWl0cz1jKDAsMS4wNSksZXhwYW5kID0gYygwLDApLGJyZWFrcz1zZXEoMCwgMSwgMC4xKSkrCiAgdGhlbWUocGFuZWwuYmFja2dyb3VuZCA9IGVsZW1lbnRfYmxhbmsoKSkrIAogIHRoZW1lKHBhbmVsLmdyaWQubWFqb3IgPSBlbGVtZW50X2JsYW5rKCksIHBhbmVsLmdyaWQubWlub3IgPSBlbGVtZW50X2JsYW5rKCkscGFuZWwuYmFja2dyb3VuZCA9IGVsZW1lbnRfYmxhbmsoKSwgYXhpcy5saW5lID0gZWxlbWVudF9saW5lKGNvbG91ciA9ICJibGFjayIpKSsKICBsYWJzKHg9IiIseT0iIikrCiAgdGhlbWUobGVnZW5kLnBvc2l0aW9uPSJub25lIixheGlzLnRleHQueCA9IGVsZW1lbnRfdGV4dChhbmdsZSA9IDcwLCBoanVzdCA9IDEpKSsKICBzY2FsZV94X2Rpc2NyZXRlKGxpbWl0cz1jKCJSYW5kb21fbGluayIsImJsZWFjaCIsIkxUX0JMIiwiTFRfSEwiLCJMVF9TTCIsIlJhbmRvbV9ub2RlIiwiVG9sZXJhbmNlX2xvdyIpLGxhYmVscz1jKCJSYW5kb21fbGluayI9IlJhbmRvbSIsImJsZWFjaCI9IkJsZWFjaGluZyIsIlRvbGVyYW5jZV9sb3ciPSJTdXNjZXB0aWJsZSIsIlRvbGVyYW5jZV9oaWdoIj0iSGlnaCBUb2xlcmFuY2UiLCJEZWdyZWVfaGlnaCI9ICJIaWdoIERlZ3JlZSIsIkRlZ3JlZV9sb3ciPSJMb3cgRGVncmVlIiwiUmFuZG9tX25vZGUiPSJSYW5kb20gTm9kZSIsIkxUX0JIIj0iSGlnaCBBdmcuIExpbmsgVG9sZXJhbmNlIiwiTFRfQkwiPSJTdXNjZXB0aWJsZSIsIkxUX0hIIj0iSGlnaCBIb3N0IExpbmsgVG9sZXJhbmNlIiwiTFRfSEwiPSJIb3N0IFN1c2NlcHRpYmxlIiwiTFRfU0giPSJIaWdoIFN5bWJpb250IExpbmsgVG9sZXJhbmNlIiwiTFRfU0wiPSJTeW1iaW9udCBTdXNjZXB0aWJsZSIpKSsKICBnZW9tX3RleHQoYWVzKCBsYWJlbD1zdGF0bGFiLHk9bWVhbitzdGQrMC4wNSkscG9zaXRpb24gPSBwb3NpdGlvbl9kb2RnZSgwLjkpLHZqdXN0ID0gMCkKCkNhcmliX3RvdDwtZGF0YTIlPiUKICBmaWx0ZXIobmV0d29yaz09IkMiLHJlbW92ZWQhPSJob3N0cyIscmVtb3ZlZCE9InN5bWJpb250cyIsUjUwX3dobz09ImJvdGgiKQpDYXJpYl90b3QkcmVtb3ZlZDwtZmFjdG9yKENhcmliX3RvdCRyZW1vdmVkLGxldmVscz1jKCJsaW5rcyI9ImxpbmtzIiwibm9kZXMiPSJib3RoIikpCgpDYXJpYjwtZ2dwbG90KCBDYXJpYl90b3QsIGFlcyh4PWFzLmZhY3Rvcihtb2RlbCksIHk9bWVhbixmaWxsPXJlbW92ZWQpKQpjX3RvdGFsPC1DYXJpYisKICBnZW9tX2Jhcihwb3NpdGlvbj1wb3NpdGlvbl9kb2RnZSgpLHN0YXQ9ImlkZW50aXR5Iixjb2xvdXI9J2JsYWNrJyxtYXBwaW5nPWFlcyhjb2w9InJlZCIpKSArCiAgZ2VvbV9lcnJvcmJhcihhZXMoeW1pbj1tZWFuLXN0ZCwgeW1heD1tZWFuK3N0ZCksIHdpZHRoPS4yLHBvc2l0aW9uPXBvc2l0aW9uX2RvZGdlKC45KSkrCiAgc2NhbGVfZmlsbF9tYW51YWwodmFsdWVzPWMoImxpbmtzIiA9ICIjN2ZjOTdmIiwgImJvdGgiID0gIiM5OWQ4YzkiLCAiaG9zdHMiID0gIiNhNmNlZTMiLCJzeW1iaW9udHMiPSIjZmZmZjk5IiksIGRyb3AgPSBGQUxTRSkrCiAgI3lsaW0oMCwxKSsKICBzY2FsZV95X2NvbnRpbnVvdXMobGltaXRzPWMoMCwxLjA1KSxleHBhbmQgPSBjKDAsMCksYnJlYWtzPXNlcSgwLCAxLCAwLjEpKSsKICB0aGVtZShwYW5lbC5iYWNrZ3JvdW5kID0gZWxlbWVudF9ibGFuaygpKSsgCiAgdGhlbWUocGFuZWwuZ3JpZC5tYWpvciA9IGVsZW1lbnRfYmxhbmsoKSwgcGFuZWwuZ3JpZC5taW5vciA9IGVsZW1lbnRfYmxhbmsoKSxwYW5lbC5iYWNrZ3JvdW5kID0gZWxlbWVudF9ibGFuaygpLCBheGlzLmxpbmUgPSBlbGVtZW50X2xpbmUoY29sb3VyID0gImJsYWNrIikpKwogIGxhYnMoeD0iIix5PSIiKSsKICB0aGVtZShsZWdlbmQucG9zaXRpb249Im5vbmUiLGF4aXMudGV4dC54ID0gZWxlbWVudF90ZXh0KGFuZ2xlID0gNzAsIGhqdXN0ID0gMSkpKwogIHNjYWxlX3hfZGlzY3JldGUobGltaXRzPWMoIlJhbmRvbV9saW5rIiwiYmxlYWNoIiwiTFRfQkwiLCJMVF9ITCIsIkxUX1NMIiwiUmFuZG9tX25vZGUiLCJUb2xlcmFuY2VfbG93IiksbGFiZWxzPWMoIlJhbmRvbV9saW5rIj0iUmFuZG9tIiwiYmxlYWNoIj0iQmxlYWNoaW5nIiwiVG9sZXJhbmNlX2xvdyI9IlN1c2NlcHRpYmxlIiwiVG9sZXJhbmNlX2hpZ2giPSJIaWdoIFRvbGVyYW5jZSIsIkRlZ3JlZV9oaWdoIj0gIkhpZ2ggRGVncmVlIiwiRGVncmVlX2xvdyI9IkxvdyBEZWdyZWUiLCJSYW5kb21fbm9kZSI9IlJhbmRvbSBOb2RlIiwiTFRfQkgiPSJIaWdoIEF2Zy4gTGluayBUb2xlcmFuY2UiLCJMVF9CTCI9IlN1c2NlcHRpYmxlIiwiTFRfSEgiPSJIaWdoIEhvc3QgTGluayBUb2xlcmFuY2UiLCJMVF9ITCI9Ikhvc3QgU3VzY2VwdGlibGUiLCJMVF9TSCI9IkhpZ2ggU3ltYmlvbnQgTGluayBUb2xlcmFuY2UiLCJMVF9TTCI9IlN5bWJpb250IFN1c2NlcHRpYmxlIikpKwogIGdlb21fdGV4dChhZXMoIGxhYmVsPXN0YXRsYWIseT1tZWFuK3N0ZCswLjA1KSxwb3NpdGlvbiA9IHBvc2l0aW9uX2RvZGdlKDAuOSksdmp1c3QgPSAwKQoKSW5kX3RvdDwtZGF0YTIlPiUKICBmaWx0ZXIobmV0d29yaz09IkkiLHJlbW92ZWQhPSJob3N0cyIscmVtb3ZlZCE9InN5bWJpb250cyIsUjUwX3dobz09ImJvdGgiKQpJbmRfdG90JHJlbW92ZWQ8LWZhY3RvcihDYXJpYl90b3QkcmVtb3ZlZCxsZXZlbHM9YygibGlua3MiPSJsaW5rcyIsIm5vZGVzIj0iYm90aCIpKQoKSW5kPC1nZ3Bsb3QoIEluZF90b3QsIGFlcyh4PWFzLmZhY3Rvcihtb2RlbCksIHk9bWVhbixmaWxsPXJlbW92ZWQpKQppX3RvdGFsPC1JbmQrCiAgZ2VvbV9iYXIocG9zaXRpb249cG9zaXRpb25fZG9kZ2UoKSxzdGF0PSJpZGVudGl0eSIsY29sb3VyPSdibGFjaycsbWFwcGluZz1hZXMoY29sPSJyZWQiKSkgKwogIGdlb21fZXJyb3JiYXIoYWVzKHltaW49bWVhbi1zdGQsIHltYXg9bWVhbitzdGQpLCB3aWR0aD0uMixwb3NpdGlvbj1wb3NpdGlvbl9kb2RnZSguOSkpKwogIHNjYWxlX2ZpbGxfbWFudWFsKHZhbHVlcz1jKCJsaW5rcyIgPSAiIzdmYzk3ZiIsICJib3RoIiA9ICIjOTlkOGM5IiwgImhvc3RzIiA9ICIjYTZjZWUzIiwic3ltYmlvbnRzIj0iI2ZmZmY5OSIpLCBkcm9wID0gRkFMU0UpKwogICN5bGltKDAsMSkrCiAgc2NhbGVfeV9jb250aW51b3VzKGxpbWl0cz1jKDAsMS4wNSksZXhwYW5kID0gYygwLDApLGJyZWFrcz1zZXEoMCwgMSwgMC4xKSkrCiAgdGhlbWUocGFuZWwuYmFja2dyb3VuZCA9IGVsZW1lbnRfYmxhbmsoKSkrIAogIHRoZW1lKHBhbmVsLmdyaWQubWFqb3IgPSBlbGVtZW50X2JsYW5rKCksIHBhbmVsLmdyaWQubWlub3IgPSBlbGVtZW50X2JsYW5rKCkscGFuZWwuYmFja2dyb3VuZCA9IGVsZW1lbnRfYmxhbmsoKSwgYXhpcy5saW5lID0gZWxlbWVudF9saW5lKGNvbG91ciA9ICJibGFjayIpKSsKICBsYWJzKHg9IiIseT0iIikrCiAgdGhlbWUobGVnZW5kLnBvc2l0aW9uPSJub25lIixheGlzLnRleHQueCA9IGVsZW1lbnRfdGV4dChhbmdsZSA9IDcwLCBoanVzdCA9IDEpKSsKICBzY2FsZV94X2Rpc2NyZXRlKGxpbWl0cz1jKCJSYW5kb21fbGluayIsImJsZWFjaCIsIkxUX0JMIiwiTFRfSEwiLCJMVF9TTCIsIlJhbmRvbV9ub2RlIiwiVG9sZXJhbmNlX2xvdyIpLGxhYmVscz1jKCJSYW5kb21fbGluayI9IlJhbmRvbSIsImJsZWFjaCI9IkJsZWFjaGluZyIsIlRvbGVyYW5jZV9sb3ciPSJTdXNjZXB0aWJsZSIsIlRvbGVyYW5jZV9oaWdoIj0iSGlnaCBUb2xlcmFuY2UiLCJEZWdyZWVfaGlnaCI9ICJIaWdoIERlZ3JlZSIsIkRlZ3JlZV9sb3ciPSJMb3cgRGVncmVlIiwiUmFuZG9tX25vZGUiPSJSYW5kb20gTm9kZSIsIkxUX0JIIj0iSGlnaCBBdmcuIExpbmsgVG9sZXJhbmNlIiwiTFRfQkwiPSJTdXNjZXB0aWJsZSIsIkxUX0hIIj0iSGlnaCBIb3N0IExpbmsgVG9sZXJhbmNlIiwiTFRfSEwiPSJIb3N0IFN1c2NlcHRpYmxlIiwiTFRfU0giPSJIaWdoIFN5bWJpb250IExpbmsgVG9sZXJhbmNlIiwiTFRfU0wiPSJTeW1iaW9udCBTdXNjZXB0aWJsZSIpKSsKICBnZW9tX3RleHQoYWVzKCBsYWJlbD1zdGF0bGFiLHk9bWVhbitzdGQrMC4wNSkscG9zaXRpb24gPSBwb3NpdGlvbl9kb2RnZSgwLjkpLHZqdXN0ID0gMCkKCiNQdXQgdGhlbSBhbGwgaW4gb25lIHBsb3QKZ2dhcnJhbmdlKGdfdG90YWwscF90b3RhbCxpX3RvdGFsLGNfdG90YWwsIG5jb2w9NCwgbnJvdz0xLCBjb21tb24ubGVnZW5kID0gRkFMU0UsIGxlZ2VuZD1OVUxMKQpgYGAKCgo=
