# Supplementary material for: Resistance and robustness of the global coral–symbiont network
Source: Ecology. 2020 Feb 14;101(5):e02990. doi: 10.1002/ecy.2990 (PMC7317464; doi:10.1002/ecy.2990)
Supplement: Supplementary file 5 [file ECY-101-e02990-s005.pdf]

Williams, S.D. and M.R. Patterson. 2020. Resistance and robustness of the global coral–symbiont network. Ecology.

Data S1

Data S1 contains files needed to make the networks in the resistance and robustness code (Data S2).

Author

Sara D. Williams
Northeastern University, Marine Science Center
430 Nahant Rd, Nahant MA 01908
Williams.sar@husky.neu.edu

File list

`Global_edges.csv`: All edges in the global network of coral-symbiont associations. Delete first row with column names before calling in code. Each following row represents one link.

Symbiont Index – index number of symbiont node

Host Index – index number of host node

T_MMM_05_degC – mean monthly maximum sea surface temperature for subregion of the host node

`Global_hostnodes.csv`: All host (coral species) nods in global network of coral-symbiont associations. Each row is a node.

Node_id – index number of node

Ocean – Ocean-basin location of node

Host_Sci_Name – scientific name of node

Type – host (0) or symbiont (1)

Genetic – Host family

Host_tol – thermal tolerance value

`Symbnodes_notols.csv`: All symbiont nodes in the global network that did not have assigned thermal tolerances. See Data S2 for code for missing tolerance values.

ID – index number of node

Phylotype – designated ITS2 type of symbiont node

Type – host (0) or symbiont (1)

Clade – clade of node

`Symbnodes_tols.csv`: All symbiont nodes in the global network that did have assigned thermal tolerances.

ID – index number of node

Phylotype – designated ITS2 type of symbiont node

Type – host (0) or symbiont (1)

Clade – clade of node

Tolerance – thermal tolerance value

`GeoSymbioScleractinians.csv`: File of unfiltered data used from the GeoSymbio database to create the Global Network. See Franklin et al. (2012) for description. Each row is a sampled association.

`GlobalNetwork.csv`: Filtered data from GeoSymbioScleractinians.csv used to make the networks. Each row is a different known association and a link.

Clade – Symbiont clade

Type – Designated symbiont ITS2 type

Host_Family – Coral host family

Host_Genus – Coral host Genus

Host_Sci_Name – Coral host scientific name

Ocean – Ocean-basin scale location of sampling location of association

Region – Subregion scale location of sampling location of association

Host_tol – Host node thermal tolerance

Symb_ID – symbiont node ID

Host_ID – host node ID

T_MMM_05 – Mean monthly maximum sea surface tolerance in 2005 of the subregion location

Files for Host-Specific Exploratory Model:

`HostSpecific_Globaledges.csv`: All edges in the host-specific network of coral-symbiont associations. Delete first row with column names before calling in code. Each following row represents one link.

`HostSpecific_symbnodes.csv`: All new symbiont nodes for the host-specific network

Files for the generalized global network:

`Generalized_global_edges.csv`: All edges in the generalized network of coral-symbiont associations. First column is the symbiont ID and second is the host ID

`Generalized_global_hostnodes.csv`: All host nodes with ID's for the generalized network.

Files for additional spatial scales are available on the GitHub repository for this paper, DOI: 10.5281/zenodo.3595582*. They can also be filtered from the* `GlobalNetwork.csv`*.*
