# Supplementary material for: Resistance and robustness of the global coral–symbiont network
Source: Ecology. 2020 Feb 14;101(5):e02990. doi: 10.1002/ecy.2990 (PMC7317464; doi:10.1002/ecy.2990)
Supplement: Supplementary file 6 [file ECY-101-e02990-s006.pdf]

**Williams, S.D., and M.R. Patterson. 2020. Resistance and robustness of the global coral–symbiont network. Ecology.**

---

## Data S2

**Data S2 contains code and data files needed to run the network bleaching model, null network models, and robustness removal models. All code was written in Python 2.7.16 using a jupyter notebook or R version 3.5.1 using R studio. The code is also available via a GitHub repository, DOI: 10.5281/zenodo.3595582.**

---

## Author

Sara D. Williams
Northeastern University, Marine Science Center
430 Nahant Rd, Nahant MA 01908
Williams.sar@husky.neu.edu

---

## File list

`Resistance.csv`: Results of bleaching model simulations and resistance calculations.

First column – Row number (ignore)

Spatial.Scale – Location/Spatial scale of network

R – Mean resistance value from 100 simulations of bleaching model

R_std – Standard deviation of resistance values from 100 simulations of bleaching model

Simulation – Which network type, natural network or null network models (Network = natural network, Shuffled Tolerances, Random Bipartite Degree Conserved, Random Bipartite Not-Degree Conserved)

Group – Used for plotting convenience

Statlab – statistical significance label from randomization test results

`Robustness.csv`: Results of the robustness analyses

Network – Which location/spatial scale (abbreviated names)

Mean – Mean R50 value from 100 simulations

Std – Standard deviation of R50 value from 100 simulations

Model – Which removal model (Bleach = bleaching model, LT_BH = average link tolerance high to low, LT_BL=susceptible links, LT_HL= susceptible host links, LT_SL=susceptible symbiont links, Random_link=random links, Degree_high= high to low degree, Degree_low=low to high degree, Random_node=random nodes, Tolerance_low=susceptible nodes)

Removed – links, both type of nodes, hosts, or symbionts

Type – link or node removal model

R50_who – Which robustness scenario (both=total, hosts=host, symbs=symbiont)

Group2 – Used for plotting convenience

Group – Used for plotting convenience

Statlab – statistical significance label from randomization test results

Connectance – connectance of network

Hosts – number of hosts in network

Symbionts – number of symbionts in network

Links – number of links in network

`TEST_resistance_perms.csv`: For testing the statistics code in the R markdown file.

Net_dtemp – resistance values from each bleaching model simulation of the natural network

hsrand_dtemp – resistance values from each bleaching model simulation of the shuffled tolerances null network

rand_dtemp – resistance values from each bleaching model simulation of the random tolerances null network

rbndc_dtemp– resistance values from each bleaching model simulation of the RBNDC null network

rbdc_dtemp– resistance values from each bleaching model simulation of the RBDC null network

`Resistance_RandomizationTest_results.csv`: Results of randomization tests of significance for the resistance results, details in Appendix S2.

Spatial Scale – Which network (abbreviations for Global, ocean-basins, or subregions)

Dif_means – Difference in observed means

Obt_p – Obtained P value

Obt_f – Obtained F statistic

Pval –P value based on difference in F statistic; (number of permutations that resulted in a F.stat > obtained F.stat + 1)/(1+number of permutations)

Type1 –which network is being compared to type2 (natural or null models)

Type2—which network is being compared to type1 (natural or null models

p_adjust_holm – holm adjusted p value

`Robustness_RandomizationTest_results.csv`: Results of randomization tests of significance for the robustness results, details in Appendix S2.

Spatial Scale – Which network (abbreviations for Global, ocean-basins, or subregions)

RobustnessType – Total, host, or symbiont robustness scenario

Dif_means – Difference in observed means

Obt_p – Obtained P value

Obt_f – Obtained F statistic

Pval –P value based on difference in F statistic; (number of permutations that resulted in a F.stat > obtained F.stat + 1)/(1+number of permutations)

Type1 –which network is being compared to type2 (natural or null models)

Type2—which network is being compared to type1 (natural or null models

p_adjust_holm – holm adjusted p value

`SwainSymbsR.csv`: Swain et al. (2017) thermal tolerance ranks that are adapted for the symbiont node thermal tolerances. See Swain et al. (2017) for file description.

`BleachingModel_Resistance_Py27.ipynb`: Jupyter notebook for simulating the bleaching model (Eq. 1), calculating resistance, degree distribution fitting, the host-specific network, and the generalized network.

`BleachingModel_Resistance_Py27.pdf` – PDF of Jupyter notebook above.

`RemovalModels_Robustness_Py27.ipynb`: Jupyter notebook for the removal models and robustness analyses

`RemovalModels_Robustness_Py27.pdf`: PDF of Jupyter notebook above.

:

`StatisticsAndVisualization_R35.rmd`: Rmarkdown for visualizing the model results, statistics described in Appendix S2, and code for symbiont node tolerances.

`StatisticsAndVisualization_R35.nb.html`: html of Rmarkdown above.

---
